# *Staphylococcus aureus* Transcriptome Architecture: From Laboratory to Infection-Mimicking Conditions

**DOI:** 10.1371/journal.pgen.1005962

**Published:** 2016-04-01

**Authors:** Ulrike Mäder, Pierre Nicolas, Maren Depke, Jan Pané-Farré, Michel Debarbouille, Magdalena M. van der Kooi-Pol, Cyprien Guérin, Sandra Dérozier, Aurelia Hiron, Hanne Jarmer, Aurélie Leduc, Stephan Michalik, Ewoud Reilman, Marc Schaffer, Frank Schmidt, Philippe Bessières, Philippe Noirot, Michael Hecker, Tarek Msadek, Uwe Völker, Jan Maarten van Dijl

**Affiliations:** 1 Interfaculty Institute for Genetics and Functional Genomics, University Medicine Greifswald, Greifswald, Germany; 2 MaIAGE, INRA, Université Paris-Saclay, Jouy-en-Josas, France; 3 Institute for Microbiology, Ernst-Moritz-Arndt-University Greifswald, Greifswald, Germany; 4 Biology of Gram-Positive Pathogens, Department of Microbiology, Institut Pasteur and CNRS ERL 3526, Paris, France; 5 Department of Medical Microbiology, University of Groningen, University Medical Center Groningen, Groningen, The Netherlands; 6 Center for Biological Sequence Analysis, Department of Systems Biology, Technical University of Denmark, Kongens Lyngby, Denmark; 7 Institut Micalis, INRA and AgroParisTech, Jouy-en-Josas, France; Indiana University, UNITED STATES

## Abstract

*Staphylococcus aureus* is a major pathogen that colonizes about 20% of the human population. Intriguingly, this Gram-positive bacterium can survive and thrive under a wide range of different conditions, both inside and outside the human body. Here, we investigated the transcriptional adaptation of *S*. *aureus* HG001, a derivative of strain NCTC 8325, across experimental conditions ranging from optimal growth *in vitro* to intracellular growth in host cells. These data establish an extensive repertoire of transcription units and non-coding RNAs, a classification of 1412 promoters according to their dependence on the RNA polymerase sigma factors SigA or SigB, and allow identification of new potential targets for several known transcription factors. In particular, this study revealed a relatively low abundance of antisense RNAs in *S*. *aureus*, where they overlap only 6% of the coding genes, and only 19 antisense RNAs not co-transcribed with other genes were found. Promoter analysis and comparison with *Bacillus subtilis* links the small number of antisense RNAs to a less profound impact of alternative sigma factors in *S*. *aureus*. Furthermore, we revealed that Rho-dependent transcription termination suppresses pervasive antisense transcription, presumably originating from abundant spurious transcription initiation in this A+T-rich genome, which would otherwise affect expression of the overlapped genes. In summary, our study provides genome-wide information on transcriptional regulation and non-coding RNAs in *S*. *aureus* as well as new insights into the biological function of Rho and the implications of spurious transcription in bacteria.

## Author Summary

The major human pathogen *Staphylococcus aureus* can survive under a wide range of conditions, both inside and outside the human body. The goal of this study was to determine how *S*. *aureus* adapts to such different conditions and, additionally, we wanted to identify general factors governing the staphylococcal transcriptome architecture. Therefore, we performed a precise analysis of all RNA transcripts of *S*. *aureus* across experimental conditions ranging from *in vitro* growth in different media to internalization by eukaryotic host cells. We systematically mapped all transcription units, annotated non-coding RNAs, and assigned promoters controlled by particular RNA polymerase sigma factors and transcription factors. By a comparison with data available for the related Gram-positive bacterium *Bacillus subtilis*, we made key observations concerning the abundance and origin of antisense RNAs. Intriguingly, these findings support the view that many antisense RNAs in a bacterium like *B*. *subtilis* could be byproducts of spurious promoter recognition by condition-specific alternative sigma factors. We also report that the transcription termination factor Rho prevents widespread antisense transcription, presumably caused by pervasive transcription initiation in the A+T-rich genome of *S*. *aureus*. Altogether our study presents new perspectives on the biological significance of antisense and pervasive transcription in bacteria.

## Introduction

The Gram-positive bacterium *Staphylococcus aureus* causes human infections that range from superficial skin infections to life-threatening diseases such as pneumonia, endocarditis, osteomyelitis, bacteremia and sepsis [1]. This major human pathogen is also a common component of skin and mucosal flora and many clinical cases arise from auto-infection [2]. In the healthy population the most important niche of the bacterium seems to be the anterior nares, with a proportion of approximately 20% permanent carriers [3]. *S*. *aureus´* host range is not limited to humans; it also infects many animals [4] and frequently causes food-borne disease due to its presence on raw meat [5]. A growing concern is the emergence of antibiotic-resistant strains, such as methicillin-resistant *S*. *aureus* (MRSA) [6,7]. The versatile nature of *S*. *aureus* relies on a wide range of virulence factors, whose expression is coordinated by a complex gene regulatory network. They facilitate the escape from host immune responses and adaptation to diverse environmental conditions (reviewed in [8,9]).

Physiological adaptation of a bacterium is coordinated largely at the transcriptional level where molecules such as RNA polymerase sigma factors, transcription factors, and regulatory RNAs, are involved in a variety of mechanisms to modulate mRNA synthesis, processing and degradation. Genome-wide transcriptome studies analyzing bacterial transcription globally and in a quantitative manner across various environmental conditions have provided deep insights into the bacterial transcriptome architecture [10–13]. In particular, by revealing the repertoire of non-coding RNAs, they raised the interest in the regulatory roles of small non-coding RNAs and antisense RNAs [14,15]. A tiling array transcriptome study of the Gram-positive model bacterium *Bacillus subtilis* exposed to a wide range of nutritional and environmental conditions established one of the most comprehensive repertoires of transcription units in a prokaryote [13]. It also evaluated the global contribution of a bacterium’s alternative sigma factors to transcriptional regulation and proposed the hypothesis that a large proportion of *B*. *subtilis* antisense RNAs could be attributed to transcription initiated by alternative sigma factors and to imperfect control of transcription termination. This raised the possibility that many antisense transcripts may not have a functional role but are spurious transcripts generated by imperfect transcription termination and unintended transcription initiation, the latter being presumably less deleterious and more frequent when linked to alternative condition-dependent sigma factors [13]. In line with this hypothesis, other studies also proposed a possible preponderant role of transcriptional noise in antisense transcription based on the weak conservation of promoters associated with these RNAs between *Escherichia coli* and *Salmonella enterica* [16] and in another group of the Gammaproteobacteria [17]. However, the extent of spurious transcription in bacterial genomes and its implications remain a matter of debate [18,19].

The availability of large-scale transcriptome data for a particular organism has also proven a very useful resource, complementing sequence-based genome annotation, for the respective research community. Indeed, the information on the genetic regulatory network of an organism provided by the characterization of the wild-type global transcriptome across a wide range of conditions is complementary to more classical studies analyzing particular mutants. The greatest asset of such an approach is its unbiased nature, which does not focus on preselected regulatory circuits and conditions. Simultaneously, it allows comprehensive and precise mapping of all transcriptome parts such as transcription units, non-coding RNA species, and transcription start sites. Taking the aforementioned *B*. *subtilis* data set as an example, the approach provides at least three useful pieces of information that jointly contribute to the discrimination between direct and indirect regulatory effects. First, the observed co-expression patterns can complement results obtained in more targeted experiments [20]. Second, the data facilitate detection of new regulatory RNA molecules [21]. Third, the detailed information on transcription units and transcription start sites facilitates the search for potential regulator binding sites [22]. This is particularly useful for the genome-wide identification of sigma factor regulons, since the bipartite degenerate motifs recognized by the sigma factors lie directly upstream of the transcription start sites. An unsupervised algorithm to identify and map these motifs by combining information from the DNA sequences and the condition-dependent activities of the detected promoters was developed recently [13].

Like *B*. *subtilis*, *S*. *aureus* is a low G+C Gram-positive bacterium but has a smaller genome (~2.8 Mbp) than *B*. *subtilis* (~4.2Mbp) and a markedly different partitioning of its promoter space since only three alternative sigma factors are encoded by its genome. In contrast, the *B*. *subtilis* genome encodes 17 alternative sigma factors, 14 of which are known to be activated in response to environmental conditions or during developmental processes (http://www.subtiwiki.uni-goettingen.de;[23]). In *S*. *aureus*, aside from SigB which is the major and by far the most investigated alternative sigma factor, SigH and SigS have been described [24,25]. Several transcriptomic and proteomic studies have mapped the SigB regulon and revealed that both structure and function of this regulon seem to differ between *B*. *subtilis* and *S*. *aureus* [26–31]. In *S*. *aureus*, SigB is a member of a complex network of regulators controlling expression of multiple virulence factors [32]. The role of SigB during infection was analyzed *in vivo* using different *S*. *aureus* strains and infection models [33–37], and a common theme seems to emerge in which SigB may contribute to host cell invasion and intracellular persistence of *S*. *aureus* [38–41]. Along with transcription initiation, transcription termination is an essential determinant of transcriptome architecture. Two pathways of transcription termination are known of which several aspects remain incompletely understood [42]. Intrinsic termination, universal to all bacteria, depends directly on the properties of the transcribed RNA sequence and does not require factors outside the elongation complex. The second pathway involves the protein Rho and coexists with intrinsic termination in the vast majority of bacteria [43]. Rho is an ATP-dependent helicase/translocase that loads onto the nascent RNA, moves 5’-3’ faster than the RNA polymerase, and provokes termination after hitting the elongation complex [44]. Unlike in *E*. *coli*, Rho is dispensable in *B*. *subtilis* and *S*. *aureus* [45,46] and many intrinsic transcription terminators are identified in their genomes [47,48].

In the present study we have analyzed the condition-dependent transcriptome of *S*. *aureus* HG001 by strand-specific tiling array hybridizations. This strain was derived from *S*. *aureus* NCTC 8325, a model strain widely used in genetic and regulatory studies, but in HG001, in contrast to its parent, the SigB-activating phosphatase RsbU is active [49]. The investigated experimental conditions ranged from optimal *in vitro* growth to interaction with host cells. The data thus obtained were analyzed *in silico*, including the systematic mapping of transcription units, annotation of non-coding RNAs, the classification of promoters according to their dependency on SigA and SigB, and the prediction of potentially new transcription factor target sites. In particular, a Fur-dependent sRNA postulated to represent the *S*. *aureus* functional analog of FsrA/RyhB was identified. Our findings contribute to a better understanding of the pathogenicity of this organism, in particular the complex regulation of genes encoding virulence factors and the adaptation of *S*. *aureus* to infection-mimicking conditions. Antisense RNAs were of particular interest because of the small number of alternative sigma factors present in *S*. *aureus*. This class of RNAs was indeed found to be relatively rare in *S*. *aureus*, overlapping only 6% of the annotated coding genes. Global analysis of the role of Rho-dependent termination in *S*. *aureus* revealed a remarkable overall increase in antisense transcription in the absence of Rho, which had an impact on expression of overlapping genes. Nonetheless, the lack of Rho triggered only slight changes to the growth behavior compared to the respective wild-type strain.

## Results

### Overall gene expression patterns of *S*. *aureus* under various physiological conditions

The transcriptome of *S*. *aureus* HG001 [49] was analyzed using 156 RNA samples taken under 44 different experimental conditions. Specifically, cells were grown in rich medium (***TSB***), minimal medium (***CDM***), cell culture media (***RPMI***, ***pMEM***), and in human plasma (***plasma***) (Figure A in S1 Data). Samples were taken during exponential growth phase (***exp***) as well as 2 hours (***t2***) and 4 hours (***t4***) after entry into stationary phase. In addition, *S*. *aureus* was grown in the presence of the cationic antimicrobial peptide colistin (***colist***) and sub-inhibitory concentrations of the following clinically most relevant antibiotics: flucloxacillin (***fluc***), vancomycin (***vanc***), ciprofloxacin (***cipro***), clindamycin (***clind***), erythromycin (***ery***), linezolid (***line***), and trimethoprim-sulfamethoxazole (***T/Smx***). Cell culture infection experiments were performed with the human bronchial epithelial cell line S9 and the human monocyte cell line THP-1. Staphylococci internalized by eukaryotic host cells were isolated from samples taken 2/ 2.5 hours (***intTHP1-2h*** and ***intS9-2h***) and 6/ 6.5 hours (***intTHP1-6h*** and ***intS9-6h***) post infection. The following additional samples were taken: i) non-adherent bacteria at multiplicity of infection (MOI) of 25 or 50 retrieved from the supernatant of S9 cells after 1 hour of infection (***nonad25-1h*** and ***nonad50-1h***); ii) bacteria after 1, 2.5, and 6.5 hours of incubation in the infection medium at 37°C and 5% CO_2_ without agitation (***CO***_***2***_***-1h***, ***CO***_***2***_***-2h*** and ***CO***_***2***_***-6h***); iii) bacteria after 2.5 hours of anaerobic incubation in pMEM medium at 37°C (***anaer-2h***); and iv) bacteria grown in RPMI medium with fetal calf serum (FCS) (***RPMI/FCS***), the pre-culture condition for the THP-1 experiments.

**Fig 1:**
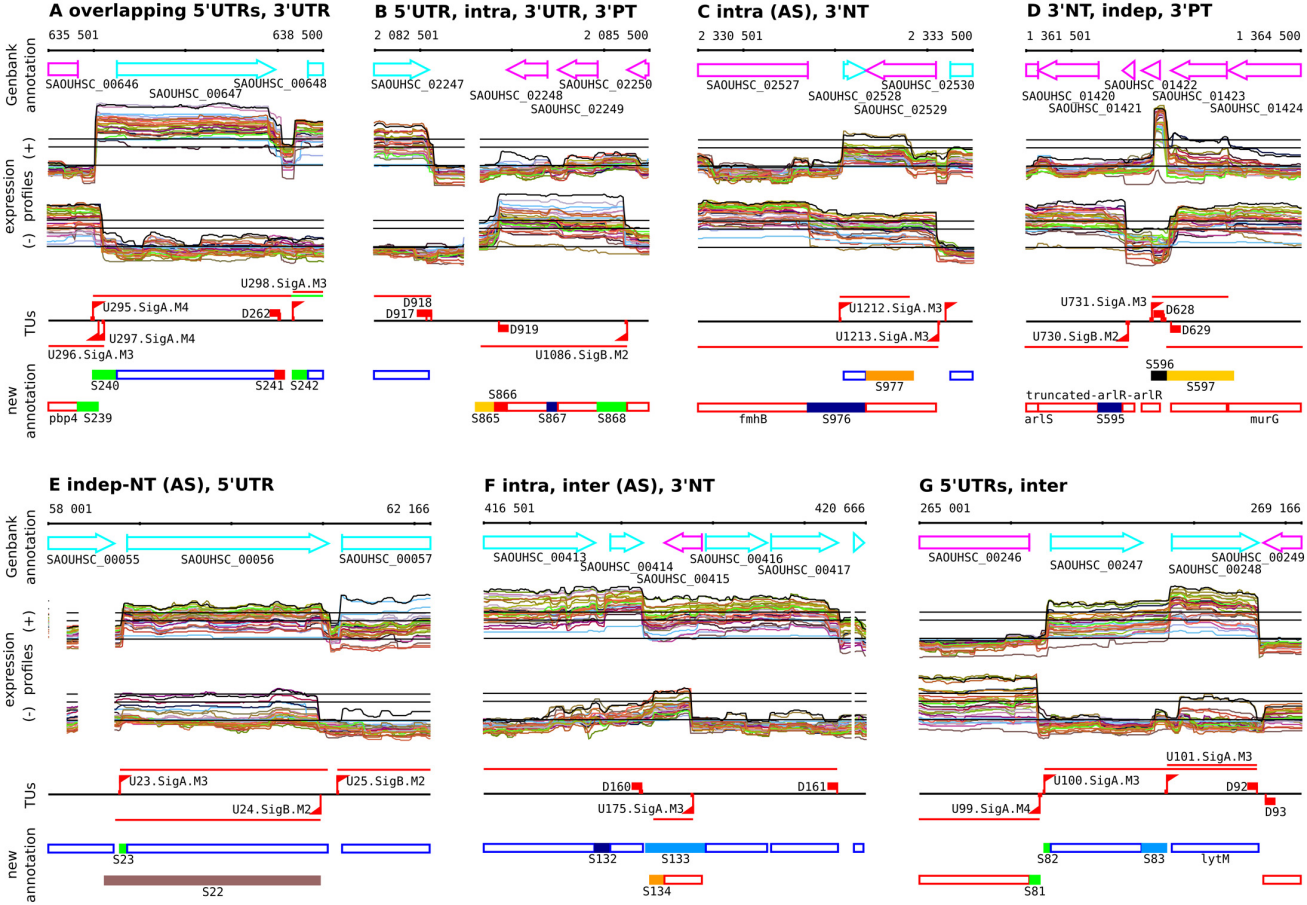
Transcriptional landscape reconstruction leads to a new annotation of the *S*. *aureus* HG001 genome. Panels **(A-G)** show examples of the different categories of transcription segments outside annotated CDSs and RNA genes. Each panel shows from top-to-bottom (i) the original GenBank annotation, (ii) a selection of 30 representative expression profiles (horizontal black lines show for each strand the chromosome median, and the associated 5-fold and 10-fold cut-offs) colored according to the position of the hybridization in 3D PCA, (iii) the detected up-shifts, the associated transcription units, and the down-shift positions, (iv) the new annotation with unannotated expressed segments colored according to the classification based on the transcriptional context. The different categories of terminal regions are **5’UTR** (green boxes) and three classes of 3’ regions: **3’UTR** (red) ending with a defined termination site, **3’NT** (orange) without defined termination site, and **3’PT** (old yellow) downstream a site of partial termination. Two categories of intergenic regions are distinguished: **intra** (dark blue) for strictly intracistronic regions, and **inter** (light blue) for regions where the downstream gene can be transcribed from its own promoter. Finally, depending of the presence or absence of a defined termination site, independent segments decompose into two categories: **indep** (black) and **indep-NT** (brown). Transcription segments overlapping (≥100bp or ≥50%) GenBank annotated genes on the opposite strand are referred to as antisense (**AS**).

The *S*. *aureus* Expression Data Browser at http://genome.jouy.inra.fr/aeb/ provides the condition-dependent transcription profiles, mapping of transcription units and the new annotation of transcribed segments along the chromosome. Transcription profiles for selected genomic regions are presented in Fig 1. For each annotated coding sequence (CDS) and newly discovered RNA feature (see next section) expression values for all samples were calculated (S1 Table). Using a stringent signal threshold of 5-fold higher than the chromosome median [13], a high proportion (2524/2836; 89%) of annotated CDSs was expressed under at least one biological condition. Of these, 110 CDSs (3.9% of all CDSs) were found to be always highly expressed, i.e. rank in the 30% most highly expressed CDSs under all the conditions tested (S2 Table).

**Fig 2:**
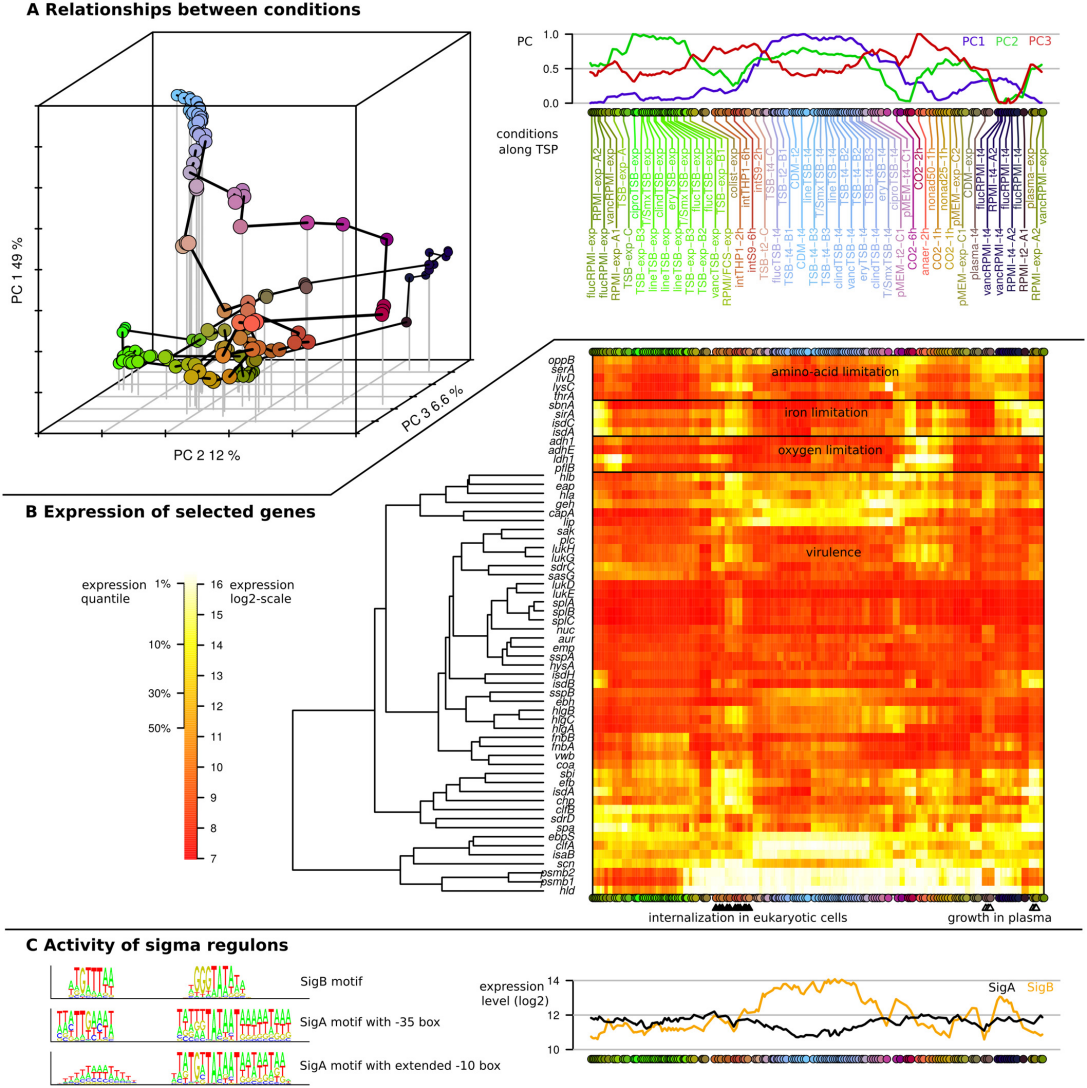
The diversity of *S*. *aureus* HG001 wild-type expression profiles across 156 RNA samples from 44 laboratory and infection-related experimental conditions. **(A)** The left subpanel shows a 3D representation of the relationships between the 156 RNA samples. This projection obtained by Principal Component Analysis accounts for ~67% of the total variance in the initial space of the expression levels of 4028 chromosomal regions (annotated genes and new segments). A different color was associated to each RNA sample according to its position in the 3D space, each axis being associated with a different color component (R/G/B). The right sub-panel displays the 156 RNA samples along a single horizontal axis corresponding to the shortest tour also represented by a black polygonal path in the 3D representation. Below the horizontal axis, a unique identifier of the biological condition is reported vertically (when consecutive RNA samples arise from the same biological condition only the first biological replicate is labeled). Above the horizontal axis, three curves indicate the coordinates of the RNA samples on the three first axes of the PCA. **(B)** Heatmap representation of the expression profiles of selected genes: reference genes for amino-acid, iron and oxygen limitation; genes known to be involved in virulence ordered by hierarchical clustering. On the left-hand side, the scale bar provides the correspondence between colors and quantile-normalized expression levels. **(C)** Activity of the SigA and SigB regulons across RNA samples computed as the average expression level of SigA-dependent and SigB-dependent promoters identified by our analysis. On the left-hand side the consensus motifs (shown as sequence logos) defined by our analysis indicate the characteristics of the different types of sigma-factor binding sites.

To elucidate the main physiological adaptations caused by the biological conditions of our study, we conducted a Principal Component Analysis (PCA). This approach allowed assessing the general relationships between the 156 RNA samples in terms of expression profiles composed of aggregated values for all annotated genes and newly discovered RNA features (Fig 2A, Text E in S1 Data). The percentage of total variance captured by the successive axes revealed a major first axis (48.9%) followed by minor axes of smoothly decreasing importance such that 67.3% of the total variance is captured with 3 axes, but 13 axes are needed to reach 90%. To account for most of the complexity of our data set (91.6% of the total variance), we examined axes 1 to 15 (Fig 2A, Figure B in S1 Data). The first PCA axis separates the conditions predominantly according to the growth phase. While transition to stationary phase is certainly associated with massive changes in gene expression, the strong impact of the growth phase on total data variability was also expected from the study design, which included many samples from exponential and stationary phase. The interpretation of the other axes was based on how the position of the samples on an axis correlated with the expression of each gene and which genes contributed most to the definition of the axis (correlations and loadings in S3 Table). We also compared how well the position on the axes summarized the whole expression profile of a given RNA sample through root mean squared errors (rmse) (Figure B in S1 Data). Genes coding for several amino acid biosynthetic pathways displayed strong negative correlation coefficients with position on axis 2, suggesting that it reflects amino acid availability. Strikingly, the RPMI stationary phase samples (“RPMI-t4”) were characterized by very low positions on this axis. Indeed, time-resolved measurements of extracellular metabolites of *S*. *aureus* strains during growth in RPMI medium revealed that amino acids are largely taken up during the exponential growth phase [50]. In the case of axis 3, positive correlation with genes induced in response to anaerobiosis (*srrA*) and negative correlation with genes induced by oxidative stress (*sufS*, *mecA2*) was observed, so that this axis might reflect the difference between aerobic and anaerobic conditions. This interpretation is supported by the position of the anaerobic growth condition (anaer-2h) on the axis 3. *S*. *aureus* grown in the presence of sub-inhibitory concentrations of several clinically relevant antibiotics exhibited overall minor gene expression changes (S4 Table).

In addition to the PCA representation we displayed the expression profiles of selected reference genes for limitations in amino acids, iron or oxygen and of 47 virulence factors comprising adhesins, immunomodulatory proteins, toxins, and secreted enzymes [51,52] (Fig 2B). Of note, PCA and expression profiles of reference genes clearly showed that the eukaryotic cell culture medium RPMI resembles the conditions *S*. *aureus* is faced with in human plasma (plasma-exp and plasma-t4), as illustrated by high expression levels of iron-regulated genes during growth in both conditions. The virulence factor genes showed a great diversity of expression profiles and all of them were expressed under at least one of the growth conditions (Fig 2B, S1 Fig). After internalization by human THP-1 macrophages or S9 bronchial epithelial cells (intTHP1 and intS9) high expression of genes involved in amino acid or iron acquisition and of many virulence-associated genes were observed (S4 Table). Overall, the variance in the data set reflects environmental conditions encountered by *S*. *aureus* during colonization and infection such as changing amino acid, iron or oxygen availability.

### Identification and classification of RNA features

In each of the 156 tiling array profiles, we mapped the transcribed regions (TRs) as well as the positions of abrupt signal increases and decreases (called up-shifts and down-shifts). The 1523 RNA 5’-ends (S5 Table) derived from the high-confidence up-shift positions are indicative of promoter sequences [13]. Downstream of these putative promoters, we delineated 1418 transcription units (TUs). According to this list of TUs, approximately one-fifth (22%) of the previously annotated CDSs can be transcribed from more than one promoter under the conditions tested (S6 Table). The analysis also identified 1261 high-confidence signal down-shift sites (S7 Table) corresponding to the RNA 3’-ends, which were further examined in connection with dissecting the role of Rho (see below).

The TRs outside of annotated CDSs and RNA genes were assigned to 1192 RNA segments (S1 to S1192) according to their structural relationship with neighboring genes (Text F in S1 Data). Our classification illustrated in Fig 1 indicates in particular the positions within TUs: 5’ of the coding region (5’UTR), intergenic (intra, inter), or 3’ of the coding region (3’UTR, 3’PT, 3’NT). Substantial complexity arises from incomplete transcription termination: the suffixes PT and NT indicate 3’UTRs following partial termination (PT) and 3’UTRs associated with lack of a termination site and slow decrease in signal intensity (NT); segments referred to as “inter” result from continuation of transcription into a downstream region located between two TUs. For the RNA features transcribed from their own promoter (independently of annotated genes), two types of segments (Indep, Indep-NT) are also distinguished depending on the presence of a defined termination site. The complete results are available in S6 Table and summarized in Table 1. A comparison of the expression levels of the different categories of RNA segments is shown in Figure C in S1 Data.

**Table 1:** Summary of the new transcription segments.

	L < 150 bp					L ≥ 150 bp				
Segment type^a^	Total (#)	AS^b^ (#)	pCDSs^c^ (#)	Length^d^ (bp)	Beaume^e^ (#)	Total (#)	AS^b^ (#)	pCDSs^c^ (#)	Length^d^ (bp)	Beaume^e^ (#)
3'UTR	144	7	0	14,718	6	69	9	4	17,740	16
3'NT	1	0	0	93	0	9	8	0	7,250	1
3'PT	6	1	0	518	1	20	14	0	12,209	4
3'ND	8	1	0	784	0	5	1	0	1,827	0
5'UTR	272	9	0	27,047	11	180	22	3	49,292	33
indep	13	3	0	1,625	9	39	9	9	12,282	22
indep-NT	1	0	0	99	0	8	7	0	8,012	2
inter	90	3	0	9,141	4	104	26	2	41,994	6
intra	118	2	0	12,184	1	105	23	2	31,071	5
Total	653	26	0	66,209	32	539	119	20	181,677	89

^a^ The different segment categories are described in the main text and Fig 1; 3’ND corresponds to 3’ regions for which no category was determined due to incomplete data in regions of repeated sequences.

^b^ Segments overlapping GenBank annotated genes on the antisense strand (≥100bp or ≥50%).

^c^ Segments containing putative unannotated CDSs predicted by SHOW (confidence probability ≥0.9).

^d^ Cumulated length.

^e^ Segments overlapping segments already reported in *S*. *aureus* N315 by Beaume *et al*. [62].

Our study identified nearly all (48/50) of the (predicted) non-coding RNAs of *S*. *aureus* NCTC 8325 from the Rfam database [53], including the generic RNAs 6S RNA, 4.5S RNA, tmRNA, RNase P, 33 *cis*-acting 5’-UTRs, and 11 small regulatory RNAs (sRNAs) including RNAIII [54] and SprD [55]. In addition, a multitude of non-coding RNAs of *S*. *aureus* has been predicted and/or experimentally identified by different global approaches, mostly for *S*. *aureus* N315 [56–62]. The RNA-seq based study by Beaume *et al*. [62] confirmed almost all non-coding RNAs from earlier studies [63]. We therefore referred to previously known non-coding RNAs of *S*. *aureus* by mapping the 186 intergenic transcripts, including 25 antisense RNAs (ASRNAs), of N315 reported by Beaume *et al*. [62] onto the NCTC 8325 genome (Text G in S1 Data). For 112 of 132 uniquely mapping transcripts our study detected a counterpart in the NCTC 8325 transcriptome (S6 Table). The remaining 20 transcripts were not detected because their expression levels were either below the threshold value defining transcribed regions or these transcripts were absent from *S*. *aureus* NCTC 8325, at least under the conditions tested.

The 22 Indep or Indep-NT segments (Table B in S1 Data) which are not antisense RNAs or associated with predicted new CDSs and do not represent a *cis*-acting 5’-regulatory region (Table C in S1 Data) can be regarded as potential *trans*-encoded small regulatory RNAs (sRNAs). Of these, three are generic RNAs (tmRNA, 6S RNA and 4.5S RNA) and 11 correspond to intergenic transcripts of N315 [62] classified as *bona fide* sRNAs. Searching against all experimentally confirmed sRNAs of *S*. *aureus* [63] (Text H in S1 Data) revealed that seven potential sRNAs (S35, S204, S414, S736, S774, S808, and S1077) were not identified by previous studies. Two of them (S736 and S808) exhibited high expression under all conditions and S1077 specifically during growth in RPMI medium; the expression of the other four genes was lower (Figure D in S1 Data). Interestingly, expression of S414 reached its highest level during growth of *S*. *aureus* in human plasma, and S414 and S774 appeared further down-regulated in the stationary phase. Expression of these seven potential sRNAs was also examined by Northern blot analysis. For six of them (i.e. S35, S204, S736, S774, S808, and S1077), the presence of a transcript with the predicted size was confirmed for exponentially growing and stationary phase cells in three cultivation media (TSB, RPMI, pMEM; Figure E in S1 Data). The remaining sRNA, S414, exhibited very low expression levels under all conditions tested in our study except for growth in human plasma. The identification of these new sRNA candidates will likely facilitate the identification of new components of the regulatory network of *S*. *aureus* since recent studies revealed important roles of sRNAs in the regulation of metabolism and virulence (reviewed in [64]).

ASRNAs accounted for 145 of the 1192 RNA features identified by our study (Table 1, S6 Table) and occurred at a rate of 51 per Mbp. With respect to the coding genes, 5.6% (159/2836) of all GenBank annotated genes were overlapped by ASRNAs under the set of growth conditions applied and these genes showed a trend towards lower expression levels compared to all genes (Figure C in S1 Data). A relatively small fraction (13%, 19/145) of the ASRNAs belonged to the group of RNA features exhibiting their own promoter, independent of annotated genes (categories Indep and Indep-NT). In *B*. *subtilis*, using similar criteria to define ASRNAs but with a higher number of different biological conditions, the rate of ASRNAs was 100 per Mbp, the proportion of overlapped genes reached 13%, and RNAs classified as Indep and Indep-NT accounted for 21% (88/423) of the ASRNAs [13]. ASRNAs appeared thus less frequent and less often initiated from their own promoter in *S*. *aureus* than in *B*. *subtilis*.

### Promoters controlled by the alternative sigma factor SigB

**Fig 3:**
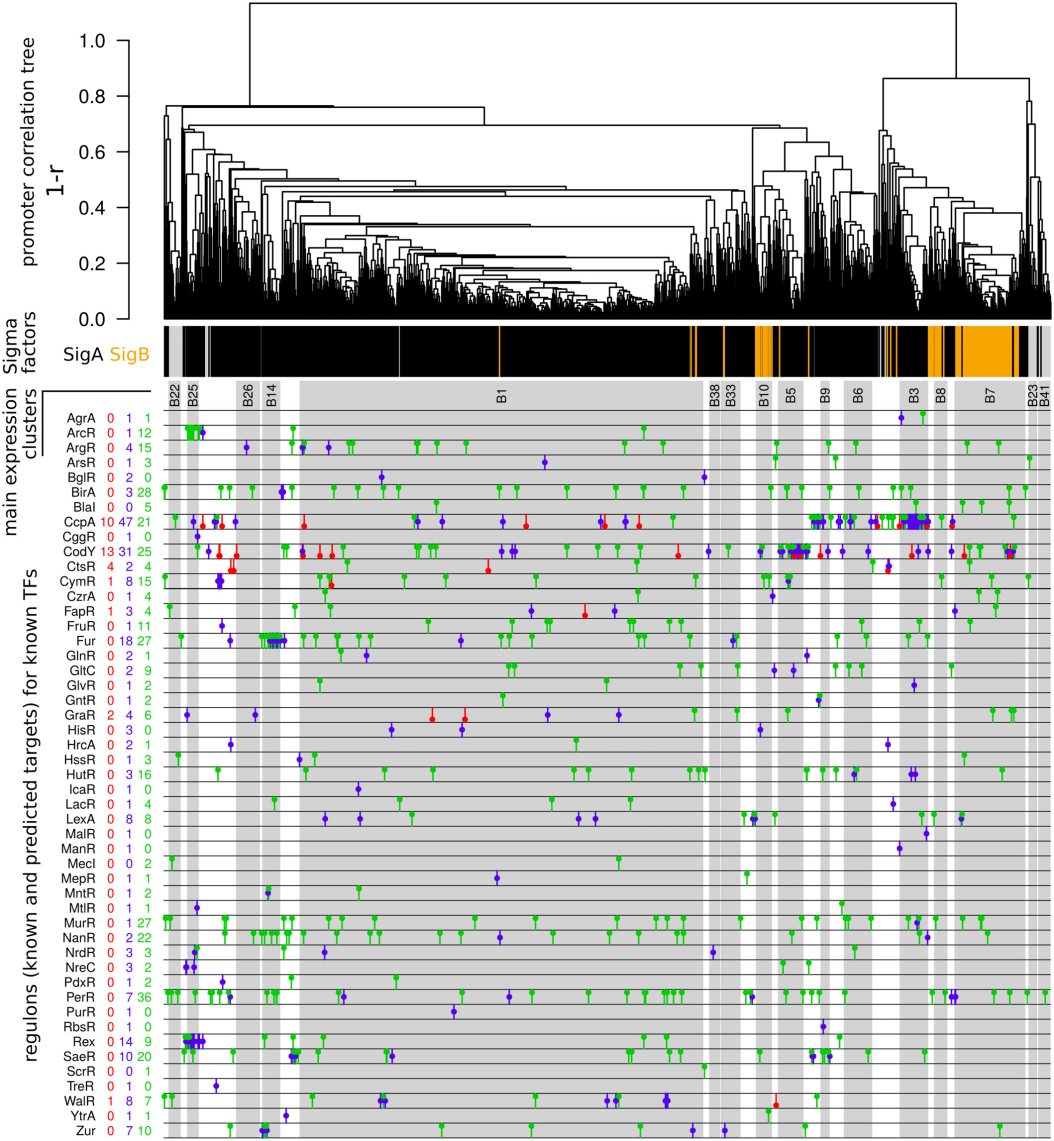
Promoter tree, sigma-factor and TFBS predictions. From top to bottom, the figure includes the following elements: A promoter tree built by hierarchical clustering of promoter activities across RNA samples based on pairwise correlations. The classification of up-shifts according to the type of sigma-factor binding sites identified (black bars for SigA, orange bars for SigB, gray bars for lack of sigma-factor binding site identification). The clusters of size ≥15 promoters obtained when splitting the tree at an average Pearson correlation coefficient 0.6. The TFBSs identified by MAST search. Here, the different transcription factors are listed on the left-hand side of the plot along with the counts for three different categories of up-shifts. The color codes used for counts and symbols are: blue for sites predicted by MAST search and included in the training (RegPrecise) set, red for sites in the training set but not identified by our MAST search, green for sites predicted by MAST search but not listed in the training set thus representing newly identified potential TFBSs.

In previous transcriptomic and proteomic studies [26–31], the assignment of genes to the SigB regulon was based on the comparison of expression levels in SigB-proficient strains versus strains lacking (active) SigB, which entails that not all of the proposed target genes are directly influenced by SigB. In addition, some regulon members might not be expressed in the particular growth conditions considered in these studies. By using an approach of promoter classification for genome-wide *de novo* identification of sigma factor regulons [13], a binding site for SigA or SigB could be assigned to most (1412, 93%) of the 1523 *S*. *aureus* up-shifts identified in our study (Fig 3, detailed information including position and sequence of the sigma factor binding sites is reported in S5 Table). With respect to the other two alternative sigma factors of *S*. *aureus*, SigH and SigS, no potential binding sites could be assigned. This was not unexpected since the *sigH* gene is cryptic in the NCTC 8325 strain under standard conditions [65] and target genes of SigS have not yet been identified [25] suggesting that the *S*. *aureus* SigS regulon may be particularly small and/or active only in a narrow range of physiological conditions.

A total of 145 promoters were classified as SigB-dependent. Their average activity profile is shown in Fig 2C. It appears that induction ratios of SigB-controlled genes in *S*. *aureus* are rather moderate (~10-fold) when compared to *B*. *subtilis* (~60-fold) [13], mainly due to higher basal activity. The conditions of SigB activation seem also much less specific since the average activity of the SigB promoters is higher than the average activity of SigA promoters in 49% of our samples (9% in *B*. *subtilis*). Furthermore, unlike in *B*. *subtilis*, the predicted SigB promoters form distinct clusters in the promoter tree summarizing the pairwise correlations of promoter activities (Fig 3), which underscores a substantial diversity of activity profiles. While, in agreement with former studies [66,30], the general pattern is an induction of SigB promoters in stationary samples (cluster B7), some SigB promoters are more specific for RPMI medium (cluster B10) and others are not induced in this medium (cluster B8) (Figure F in S1 Data). Analysis of the promoter sequences of the three main SigB-related clusters showed characteristic features for the smaller clusters B8 and B10, namely a lesser degree of conservation of the -35 region (B8) or -10 region (B10) and occurrence of a conserved AA motif upstream of the -10 region (B8 and B10) (Text I and Figure G in S1 Data). On average, activity was highest during stationary phase in TSB and CDM, but also occurred in the other growth media except for human plasma and not always associated with stationary phase, such as in CDM exponential phase samples (Fig 2C). A recent proteome study reported the activation of SigB following internalization of *S*. *aureus* by S9 cells [40]. In our data we see a weak induction at the early time point after internalization (2 and 2.5 hours, respectively, post-infection) by S9 epithelial cells and THP-1 macrophages which disappeared at the second time point (6 or 6.5 hours post-infection). It is also interesting to note that, in the promoter correlation tree, we find promoters predicted as SigA-dependent even in the clusters most enriched in SigB promoters (B7, B8, B10), pointing to the potential indirect regulation of these genes by regulators downstream of SigB. Alternatively, this observation might also suggest that similar expression profiles can be generated by different regulatory mechanisms.

Based on the repertoire of transcription units defined by our study, 249 annotated protein-coding genes were found to be preceded by a SigB binding site, out of which 163 (65%) were considered as SigB-regulated by (one of) the previous studies (Table D in S1 Data). Thus, our study led to the discovery of 86 new SigB-controlled genes in *S*. *aureus*, which are involved in diverse biological functions. Among the predicted SigB regulon members were also the known SigB-dependent sRNA RsaA (S210/S211) [58] involved in virulence factor regulation through repression of the transcription factor MgrA [67] and a newly identified Indep-NT ASRNA (S22) that covers SAOUHSC_00056 encoding a putative AraC family regulator.

Considering the ASRNAs, 12% (18/145) were identified as transcribed from SigB-dependent promoters, and the percentage dropped to 5% (1/19) for ASRNAs belonging to categories Indep and Indep-NT. In contrast, 75% (109/145) of the ASRNAs were identified as transcribed from SigA-dependent promoters. The remaining fraction consisted of ASRNAs that lacked an identified promoter. Interestingly, the proportion of ASRNAs identified as SigB-dependent in *S*. *aureus* is clearly lower than the total contribution of alternative sigma factors to antisense transcription in *B*. *subtilis*. In this organism 50% (213/423) of the ASRNAs were predicted to be transcribed by one of the alternative sigma factors and the percentage raised to 68% (60/88) for the ASRNAs classified as Indep or Indep-NT. Many of the ASRNAs in *B*. *subtilis* are transcribed from sporulation sigma factors that do not have counterparts in *S*. *aureus*. However, even SigB-generated ASRNAs occurred at lower rates in *S*. *aureus* than in *B*. *subtilis* (6.7 Mbp^-1^ versus 12 Mbp^-1^), and this was much more pronounced for the subset of ASRNAs belonging to the categories Indep and Indep-NT (0.35 Mbp^-1^ in *S*. *aureus* vs. 3.8 Mbp^-1^ in *B*. *subtilis*). In contrast, SigA-dependent ASRNAs tended to occur at similar rates in the two organisms with 39 Mbp^-1^ in *S*. *aureus* vs. 57 Mbp^-1^ in *B*. *subtilis* and 5.7 Mbp^-1^ vs. 4.7 Mbp^-1^ for the categories Indep and Indep-NT. The comparatively low frequency of ASRNAs in *S*. *aureus* appears thus as the combined outcome of the small number of alternative sigma factors and of the small contribution of SigB to antisense transcription in this organism.

### Transcription factor binding site analysis to predict new regulon members

Coordinated regulation of promoter activities is summarized in the promoter correlation tree and the associated clustering: 1242 up-shifts (82%) belonged to 16 activity clusters with ≥15 members defined by a cutoff on average Pearson correlation set to 0.6 (Fig 3). The largest cluster (B1) alone gathered 46% of all the up-shifts. We used the known information on transcription factor (TF) regulons to better understand the contribution of TFs other than SigB to the regulation underlying these correlations. In particular we relied on the experimentally identified and manually curated inferred binding sites of 47 TFs from the RegPrecise database [68] for the identification of additional potential TF binding sites (TFBSs). This analysis allowed identification of 623 known or predicted TFBSs associated to a total of 470 up-shifts (S8 and S9 Tables). These sites were unevenly distributed in the correlation tree (Fig 3). In particular, SigA-dependent promoters outside the largest activity cluster (B1) were markedly more frequently associated with identified TFBSs (46%) than SigA-dependent promoters inside this cluster (23%) or SigB-dependent promoters (19%). Furthermore, each of the four smaller clusters of SigA-dependent promoters exhibiting the highest within-cluster correlation (indicative of a low diversity of expression profiles) was found associated with a particular TF: B3 (50 promoters) with the master regulator of carbon catabolite repression CcpA, B5 (45 promoters) with the global nutritional regulator of stationary phase adaptation and virulence CodY, B14 (32 promoters) with the ferric-uptake regulator Fur, and B25 (21 promoters) with the redox-sensing transcriptional repressor Rex. Average activity profiles for these four groups of promoters are shown in Figure F in S1 Data.

For a number of regulons the MAST search allowed new predictions with strongly correlated expression profiles, in particular for Fur, Rex, CodY, and CcpA (Fig 3). For the Rex repressor involved in anaerobic gene regulation, 9 promoters were newly identified, of which 2 clustered with the 14 known Rex-controlled promoters in B25. The potential new Rex targets associated with these promoters are two predicted membrane proteins of unknown function (SAOUHSC_00146 and SAOUHSC_01133). As a second example we looked at the Fur regulon for which 9 promoters with newly predicted binding sites were found in cluster B14 together with the majority of known Fur-dependent promoters. Fur is a global regulator of iron homeostasis, which generally represses genes for siderophore biosynthesis and iron transport under iron-sufficient conditions; in *S*. *aureus*, several iron uptake systems are known to be controlled by Fur [69,70]. Correspondingly, of 21 annotated genes associated with the 9 newly predicted Fur-regulated promoters in cluster B14, 10 genes are implicated in iron/trace metal transport (*fhuD1*, *opp-1* operon, *mntABC*) or iron release from heme (*isdI*). Of these, the *mntABC* operon encoding a manganese transporter is a potential new member of the *S*. *aureus* Fur regulon. For the other genes, Fur-dependent regulation has already been reported but with different localization of the Fur box (Text J in S1 Data).

TFBS prediction also revealed a potentially Fur-dependent non-coding RNA (S596) with a size of ~180 nt that was also confirmed by Northern blot analysis (Figure E in S1 Data) located on the opposite strand of a short annotated CDS (SAOUHSC_01422, 66 amino acids) of unknown function (Figure H in S1 Data). Of note, annotation of SAOUHSC_01422 might be incorrect since short CDSs are notoriously difficult to identify. Indeed, an amino-acid level similarity search with blastp retrieves significant hits only with other hypothetical proteins of *S*. *aureus* and a few other *Staphylococcus* species. According to the SHOW CDS prediction software [71] the confidence probability associated with this putative CDS is also very low (value of 0.18). For these reasons we assume that S596 may be a *trans*-encoded sRNA rather than a *cis*-encoded ASRNA. Indeed, the occurrence and role of Fur-dependent sRNAs was reported for several bacterial species (reviewed in [72]). Target prediction for S596 using CopraRNA [73] revealed enrichment of genes encoding iron-containing proteins and of genes with functions in cofactor metabolism. Potential targets of this sRNA also included *arlRS* encoding a virulence-associated two-component system (Table E in S1 Data).

### Termination of TUs identified in the *S*. *aureus* wild-type is predominantly Rho-independent

Specific sequence elements, typically encoding a hairpin structure followed by a U-rich tract, direct dissociation of the RNA polymerase from the DNA template at intrinsic termination sites. Applying a 30 bp distance cut-off, these elements were searched using the TransTermHP software [48]. They were found for 67% of the 1261 identified high-confidence down-shifts (Figure I in S1 Data, S7 Table) suggesting that intrinsic, also known as Rho-independent, termination determines the 3’-end of a majority of the TUs with defined termination sites in *S*. *aureus*. Most down-shift sites were also found associated with predicted intrinsic terminators in *B*. *subtilis*, where the transcription termination factor Rho was shown to be involved in termination of a particular class of TUs lacking a defined 3’-end. These TUs were characterized by slowly decreasing expression, apparently due to the lack of an efficient intrinsic terminator (categories 3’PT and 3’NT comprising 3’-extended mRNAs, and category Indep-NT) [13], and they often overlapped the antisense strand of annotated genes. In *S*. *aureus*, only 19 TUs lacked a defined termination site (3’NT and Indep-NT, Table 1), which is strikingly less than the 120 TUs found in *B*. *subtilis* (6.7 per Mbp^-1^ vs. 28.5 Mbp^-1^). The 3’PT and AS segments were also about twice less frequent than in *B*. *subtilis* (26 vs. 78, 9.2 vs. 18.4 Mbp^-1^ for 3’PT; 145 vs. 423, 51.4 vs. 100.3 Mbp^-1^ for AS).

Intrigued by the very limited impact of *rho*-deletion on the growth of *S*. *aureus* [46] and by the low abundance of TUs that we could anticipate being targets of Rho-dependent termination, we decided to examine the role of Rho in this organism by comparatively profiling the transcriptomes of the wild-type and its isogenic *rho*-deletion mutant. Chromosomal regions with Rho-dependent changes in transcript levels were mapped by comparing the normalized tiling array transcription profiles of the Δ*rho* mutant and the parental strain HG001 harvested during exponential growth and four hours after entry into stationary phase in TSB and RPMI medium. In both media, growth of the *S*. *aureus* Δ*rho* mutant was almost identical to that of the parental strain, except reaching a slightly lower final OD (Figure J in S1 Data). The Expression Data Browser at http://genome.jouy.inra.fr/aeb/ also provides the condition-dependent transcription profiles of the Δ*rho* mutant.

**Fig 4:**
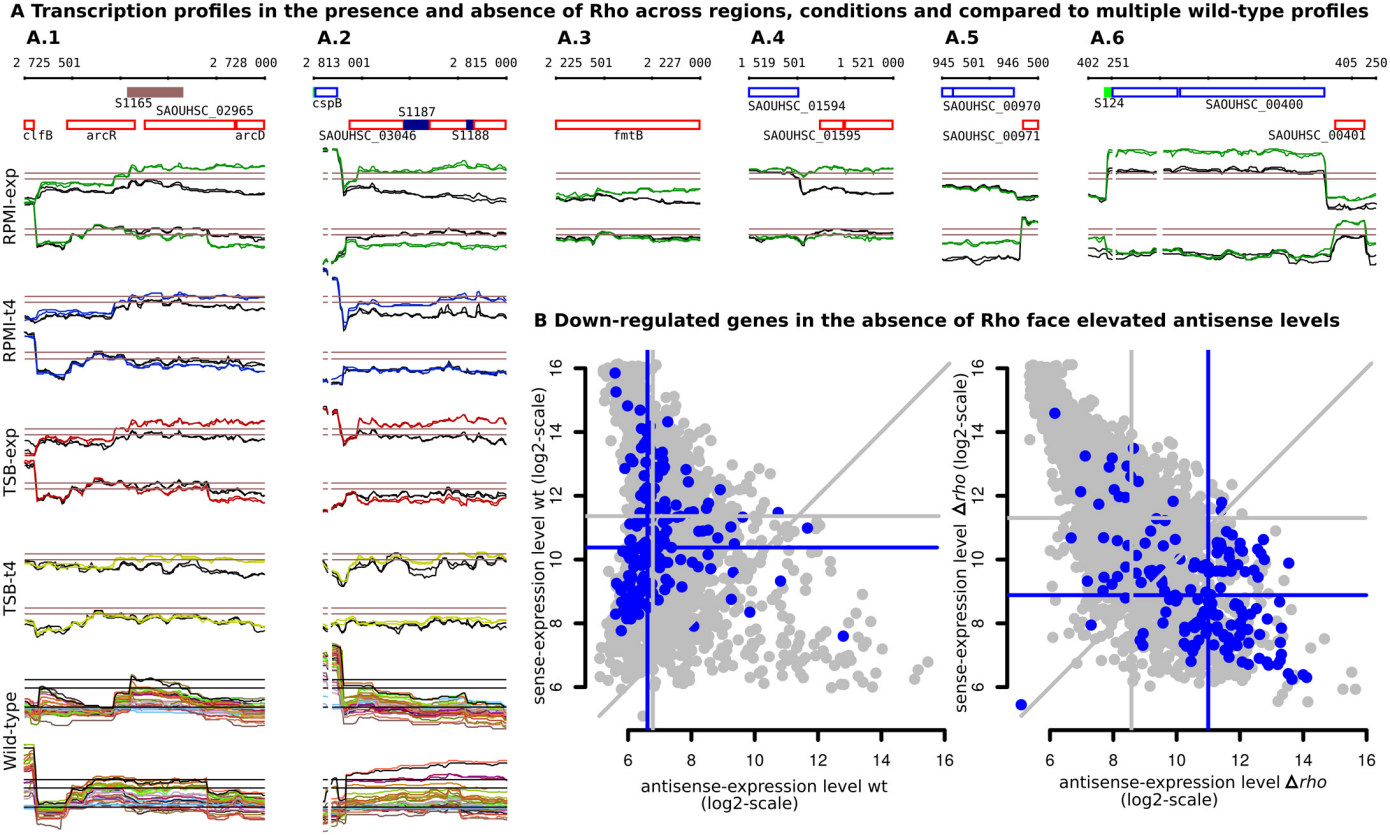
Context and impact of elevated antisense expression levels in the Δ*rho* mutant. **(A)** Transcription profiles for selected regions showing different effects of *rho* deletion, from left to right: 1) flattening of the downstream drift expression patterns typical of regions lacking defined termination sites (3’PT and 3’NT); 2) & 3) expression of regions for which no promoters are detected in the wild-type; 4) & 5) transcriptional read-through at defined termination sites; 6) higher transcript levels of coding genes. For the first two regions we show the transcription profiles for the four growth conditions examined, with wild-type profiles in black and Δ*rho* mutant profiles in condition-specific colors, as well as the 30 representative wild-type profiles. As seen in these examples, the impact of *rho* deletion tends to be stronger in RPMI than in TSB medium and in exponential growth than in stationary phase. Some degree of decrease of sense transcript levels, which may be caused by elevated antisense levels, is seen in these examples. **(B)** Sense versus antisense transcription levels in the wild-type and in the Δ*rho* mutant for exponential growth in RPMI. Each annotated gene is represented by a point. There is a strong negative correlation between sense and antisense expression in the Δ*rho* mutant (Pearson correlation coefficient r = -0.73) that is also visible but much weaker in the wild-type (r = -0.30). Indeed, antisense levels tend to increase genome-wide in the Δ*rho* mutant, except for the antisense strand of the most highly expressed genes. The most down-regulated genes (expression level in the Δ*rho* mutant is ≤50% of the wild-type) are highlighted in blue; they face antisense transcripts with particularly elevated levels in the Δ*rho* mutant. Horizontal and vertical lines indicate the medians (global in gray, most down-regulated genes in blue).

As anticipated, when examining the TUs belonging to the 3’NT and Indep-NT classes, it was observed that most of them (12/19) possessed 3’-extensions in the Δ*rho* mutant relative to the parental strain (Fig 4A.1), supporting the assumption that termination of this class of TUs depends on Rho as previously shown for *B*. *subtilis*. Of the remaining 7 TUs, 3 were not expressed in wild-type cells under the selected growth conditions and one exhibited higher expression levels in the mutant thus not allowing conclusions on the contribution of read-through to the observed 3’-extension.

Nevertheless, extension of TUs lacking an intrinsic terminator (3’NT and Indep-NT) accounted for only a very small fraction of the increase in transcript levels detected in absence of Rho and we therefore decided to analyze all chromosomal regions where normalized transcript levels were 4-fold higher in the Δ*rho* mutant than in the parental strain in at least one of the four conditions (Text D in S1 Data) (Table 2 and S10 Table). These individual regions were up to 38 Kbp long. Globally, the fraction of the chromosome up-regulated was largest during exponential growth (RPMI-exp: 1.51 Mbp; TSB-exp: 0.91 Mbp) and much smaller during stationary phase (RPMI-t4: 0.54 Mbp; TSB-t4: 0.16 Mbp). Despite these differences in magnitude, the regions subjected to up-regulation were remarkably consistent between conditions: out of the 1.65 Mb (distributed in 416 regions) identified in at least one of the four conditions, as much as 92% (1.51 Mb in 358 regions) were upregulated in RPMI-exp (Table 2). Importantly and expectedly, the predominant effect of Rho deficiency was clearly up-regulation of transcript levels; using similar criteria as for up-regulation we detected only 0.16 Mbp (in 195 regions) exhibiting down-regulation. We also noticed that the magnitude of the effect of the absence of Rho tended to be consistent with the expression level of *rho* in the *S*. *aureus* wild-type, which is higher during exponential growth than in stationary phase, particularly in TSB medium (Figure K in S1 Data). Indeed, the expression level of *rho* probably reflects its importance that may itself well be a direct function of the global transcriptional activity.

**Table 2:** Characteristics of regions up-regulated in the Δ*rho* mutant across four experimental conditions.

Characteristics^a^	RPMI exp	RPMI t4	TSB exp	TSB t4	All^b^
Total number (#)	358	192	235	141	416
Cum. Length (bp)^c^	1,514,754	539,446	906,070	164,919	1,645,632
Mean Length (bp)	4,231	2,801	3,856	1,170	3,956
Max. Length (bp)^d^	38,791	22,468	38,528	7,603	38,791
GB U new segments (%)^e^	9.2%	8.3%	9.0%	28.3%	11.1%
AS to GB (%)^f^	77.0%	77.9%	79.9%	62.4%	75.7%
Gene in 5’ (#)^g^	37	22	25	23	-
Term. in 5’ (#)^h^	53	6	11	23	-
NT in 5’ (#)^i^	6	6	7	3	-
Gene U Term. U NT (#)^j^	90	34	41	42	-
Gene U Term. U NT (bp)^k^	407,778	125,445	176,173	51,835	-
Probes x4 (%)^l^	69.3%	52.4%	59.6%	44.1%	-

^a^ Characteristics of regions where a four-fold up-regulation was detected in the Δ*rho* mutant and extended on both sides to include adjacent regions with two-fold up-regulation.

^b^ Regions defined from positions up-regulated in at least one of the four experimental conditions (RPMI exp, RPMI t4, TSB exp, TSB t4).

^c^ Cumulated length.

^d^ Maximum length.

^e^ Fraction of regions overlapping GenBank annotated genes and new expression segments detected in the wild-type.

^f^ Fraction of regions antisense to GenBank annotations.

^g^ Number of regions of which the first 200 bp overlap with a gene up-regulated in the Δ*rho* mutant (annotated gene or new segment of categories 5’, indep, and indep-NT).

^h^ Number of regions with a Rho-dependent termination site located around their 5’-end.

^i^ Number of regions of which the 5’-end (±500 bp) overlap with a 3’NT or Indep-NT segment detected in the wild type.

^j^ and ^k^ Number and cumulated length of regions counted in at least one the three aforementioned categories (^g^, ^h^, ^i^).

^l^ Fraction of positions exhibiting four-fold up-regulation.

It was expected that most of the up-regulated regions in the Δ*rho* mutant would result from extensions beyond the transcript 3’-ends due to read-through of Rho-dependent terminators. However, our analysis (Text D in S1 Data) revealed that the number of up-regulated regions due to read-throughs was indeed rather limited (S7 and S10 Tables). For instance, in the condition of exponential growth in RPMI, where the effect of Rho deletion was most pronounced, only 59 out of the 358 up-regulated regions were linked to read-through transcription (53) or lack of an intrinsic terminator (6) (Table 2). Together, in the three conditions with the strongest effect of Rho deletion, we detected only 60 termination sites at which read-through transcription can be assumed to contribute significantly to higher downstream transcript levels in the mutant. Complete read-through occurred at only three termination sites. In most cases only a limited level of read-through in the absence of Rho was observed (Fig 4A.4 & 4A.5). Of note, these partial read-throughs were also observed at sites where intrinsic terminators are predicted.

Besides read-through transcription or lack of an intrinsic terminator, another source of up-regulated regions in the Δ*rho* mutant was the higher expression of coding genes sometimes followed by (often long) downstream extensions (Fig 4A.6). These altered expression levels are likely to be indirect regulatory effects caused by Rho deficiency. Altogether, 180 genes were strongly (more than 4-fold) up-regulated in the mutant in at least one of the four growth conditions (S11 Table). The highest number of up-regulated genes (148) was observed during exponential growth in RPMI, but no more than 45 of the 358 up-regulated regions could be considered as caused by the higher expression of coding genes (Table 2). Taken together, read-through transcription (including extensions of TUs lacking a defined 3’-end in the wild-type) or higher expression of coding genes could not account for more than one third of the up-regulated regions (Table 2).

### Rho minimizes the effects of pervasive antisense transcription initiation

The largest group of Rho-dependent transcripts was indeed formed by antisense transcripts only detectable in the absence of Rho that could not be linked to the TUs identified in the wild-type (Fig 4A.2 & 4A.3). Globally, of the 1.65 Mbp covered by the 416 regions up-regulated in the Δ*rho* mutant, only 11% corresponded to annotated genes or RNA segments detected in the wild-type, whereas 76% mapped to the antisense strand of annotated genes (Table 2). As much as 52% of the 2.37 Mbp of annotated genes was overlapped on the antisense strand. Often, long genomic regions were covered by antisense transcripts in the Δ*rho* mutant as, for example, the complete prophages phi11 and phi12 that are each approximately 40 kbp long. In these regions, the increase in antisense transcript levels in the Δ*rho* mutant was most pronounced during exponential growth in RPMI and TSB, conditions of low prophage gene expression.

Antisense transcripts only detectable in the Δ*rho* mutant correspond presumably to very short and/or unstable transcripts in the wild-type, which might be terminated with the help of Rho shortly after initiation from promoters that are thus normally cryptic. However, Rho inactivation did not induce additional strong defined up-shift sites comparable to typical promoters, as indicated by visual inspection of the transcription profiles and a systematic search for up-shifts in the mutant with the criteria applied for the initial mapping of the wild-type promoters. Instead, the patterns of transcription in the Δ*rho* mutant seem shaped by pervasive low-level promoter activity. This raised the interesting question whether the action of Rho and/or this pervasive promoter activity is really more pronounced on the antisense strand or simply masked by the higher levels of normal transcriptional activity on the sense strand. During exponential growth in RPMI, where antisense up-regulation was the strongest, the median log2-ratio between the Δ*rho* mutant and the wild-type was -0.13 (no global up-regulation) for the 292 annotated genes (sense level) with log2 expression signal ≤8 in the wild-type, as compared to 1.11 (global up-regulation) for the 2453 antisense transcripts of annotated genes with log2 expression signal ≤8. Therefore, the absence of Rho seems to impact less on sense strands than antisense strands even after accounting for the potential masking effect of the coding transcripts (Figure L in S1 Data).

*S*. *aureus* has a genome markedly more A+T-rich (67.2%) than *B*. *subtilis* (56.5%) and *E*. *coli* (49.2%). We therefore wanted to examine to what extent different genome compositions may lead to a different rate of random occurrence of the TATAAT hexamer that corresponds to the canonical core of the -10 box for the housekeeping sigma factor (SigA in *S*. *aureus* and *B*. *subtilis*, Sig70 in *E*. *coli*) in the three genomes (GenBank: CP000253, AL009126.3, U00096.3). The TATAAT hexamer was indeed much more frequent (0.905 Kbp^-1^) in the antisense strand of protein-coding genes in the *S*. *aureus* genome than in the *B*. *subtilis* (0.332 Kbp^-1^) or *E*. *coli* genome (0.091 Kbp^-1^). In each of these three genomes the TATAAT hexamer was also less frequent on the sense strand than on the antisense strand (e.g., 0.905 Kbp^-1^ vs. 0.479 Kbp^-1^ in *S*. *aureus*). This possibly reflects, at least partly, the fact that the hexamer TATAAT cannot start at the second position of a codon within a gene since it contains the TAA stop codon. The extent of differential abundances of the TATAAT hexamer between and within genomes seems thus consistent with the idea that nucleotide composition may contribute to shape the patterns of pervasive transcription initiation counteracted by Rho.

Antisense transcription can change the expression of the overlapped genes. Therefore, we investigated the effect of elevated antisense transcription on sense transcript levels under Rho-deficient conditions. Indeed, of the 167 annotated genes most down-regulated (log2-ratio≤-1, q-value≤0.05) in the Δ*rho* mutant during exponential growth in RPMI medium (S11 Table), 153 (92%) were overlapped by an up-regulated region on the opposite strand. Strikingly, down-regulation was detected mostly at loci where the level of antisense transcription is high compared to the sense level. In fact, the antisense strands reached higher levels than sense strands in the Δ*rho* mutant for 73% of the 167 most down-regulated genes (Fig 4B), and in most cases (59% of 167) the antisense levels in the mutant appeared even higher than the sense levels in the wild-type (see medians in Fig 4B). We also analyzed more globally the levels of sense and antisense transcripts of all annotated genes in the wild-type and the Δ*rho* mutant. Indeed, antisense transcription increased predominantly in the regions of low sense expression leading to a strong negative correlation between sense and antisense expression (Pearson correlation coefficient r = -0.73) that was much weaker in the wild-type (r = -0.30), but also statistically significant (p-value<10^−15^) (Fig 4B). In particular the most highly expressed genes showed the lowest levels of antisense transcription in the Δ*rho* mutant. This observation suggests that down-regulation of sense expression by antisense expression is mirrored by down-regulation of antisense by sense expression, the impact of which increases with the sense expression level.

### Northern Blot analysis of antisense transcripts detected in the Δ*rho* mutant

**Fig 5:**
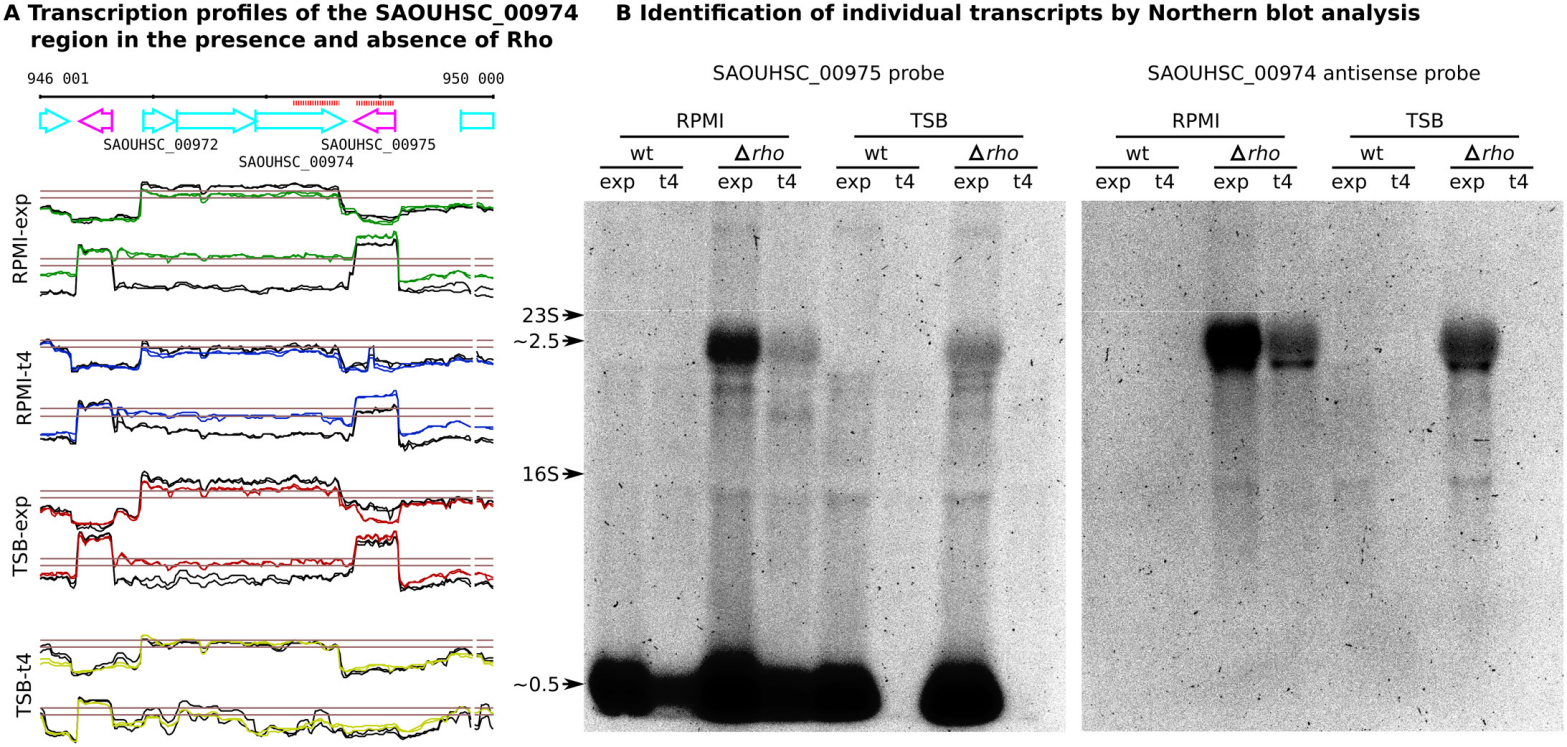
Northern blot analysis of a Δ*rho* mutant-specific antisense transcript facing a tri-cistronic transcription unit starting with SAOUHSC_00972. **(A)** Transcription profiles of the genomic region assayed. *S*. *aureus* wild-type (black lines) and Δ*rho* mutant (colored lines) were grown in RPMI and TSB medium. **(B)** Northern blot analysis of the same RNA samples (5 μg per lane) using RNA probes (indicated by red bars) directed against SAOUHSC_00975 and the antisense strand of SAOUHSC_00974. The SAOUHSC_00975 probe detected the 0.5-kb mRNA in both strains and the longer read-through transcript in the Δ*rho* mutant. The antisense specific probe detected only the 2.5-kb transcript specific to the mutant samples.

In principle, the increased transcript levels observed outside of annotated genes in the Δ*rho* mutant based on the tiling array signals may originate from long RNA molecules or from overlapping small molecules resulting from degradation of longer transcripts [74]. To discriminate between these two hypotheses, we performed Northern blot analyses for six antisense-generating transcribed regions that appeared longer or only present in the Δ*rho* mutant, mainly as a result of read-through transcription at TU ends. For all six antisense transcripts, the analysis confirmed the presence of large-size RNA molecules that were specific for the mutant samples (Fig 5, Figure M in S1 Data). In three cases, the size of the detected band suggested the presence of an intact transcript covering the complete region identified to be upregulated in the Δ*rho* mutant. In a fourth case (3’PT transcript S931), the obtained size matched the position of a marked drop in the tiling array signal. For a ~9 Kb long transcribed region downstream of S234 and a ~11 Kb long region downstream of S596, Northern blot analysis detected transcripts with different sizes, all larger than 2 Kb.

The example in Fig 5 shows the analysis of a read-through transcript downstream of SAOUHSC_00975 that faces a tricistronic TU (SAOUHSC_00972 to SAOUHSC_00974) on the opposite strand using probes with specificity for SAOUHSC_00975 and for the antisense strand of SAOUHSC_00974, i.e. the 3’-extension detected in the absence of Rho. With the first probe, the SAOUHSC_00975 mRNA with a size of ~0.5 Kb (not expressed in stationary phase TSB samples in agreement with the tiling data) and the longer read-through transcript with a size of ~2.5 Kb appearing in the Δ*rho* mutant were detected. The second probe, specific to the antisense region, showed only the longer transcript.

## Discussion

### Comprehensive analysis of the *S*. *aureus* transcriptome

Using a genomic tiling array approach, the transcriptome of *S*. *aureus* HG001 was systematically analyzed under a broad range of experimental conditions to allow for an extensive mapping of TUs, annotation of non-coding RNAs and further insights into the transcriptional regulatory network of *S*. *aureus*. Almost 90% of the annotated genes were expressed in at least one growth condition but only 110 CDSs (3.9%) were always found to be highly expressed (S2 Table), which is indicative of the richness of our data set. The 110 always highly expressed CDSs encompass genes encoding ribosomal proteins and translation factors (18 genes), enzymes of central carbon metabolism (14), proteins involved in cell division and cell wall metabolism (10), RNA polymerase subunits (*rpoA*, *rpoB*, *rpoC*, *sigB*), and half (55/110) belong to the set of 351 genes of *S*. *aureus* NCTC 8325 identified as essential for survival and growth *in vitro* [75] (S2 Table). They also comprise transcriptional regulators (*perR*, *rex*, *icaR*), and proteins involved in oxidative stress management (*katA*, *ahpC*, *ahpF*) whose generally high expression might provide a molecular basis for the rather high level of oxidative stress resistance of *S*. *aureus*, a reflection of the frequent encounter of oxidative stress in the interaction with its host/ natural environments.

The data established a detailed description of the transcriptional architecture of the bacterium. In particular, new RNA segments were classified according to their position in the transcript relative to neighbor genes and transcript ends. Most of the 3’-ends were associated with a predicted intrinsic terminator. Transcription units were delineated and their promoters assigned to sigma factor regulons.

### The *S*. *aureus* SigB regulon

SigA or SigB binding sites could be assigned to 1412 of the 1523 transcription signal up-shifts detected, which led to the identification of 145 SigB-dependent promoters. In *B*. *subtilis*, 2935 promoters were classified according to their dependence on different sigma factors [13] and 170 (6% versus 10% in *S*. *aureus*) were assigned to SigB, which controls the general stress regulon. A relatively small fraction (about 12%) of the genes supposed to be controlled by SigB in *S*. *aureus* [29–31] have homologous counterparts in the *B*. *subtilis* SigB regulon. Besides roughly 30% of genes with unknown function, the *S*. *aureus* SigB regulon contains genes involved in cell envelope composition, membrane transport processes, intermediary metabolism, and virulence. Our study identified 86 new protein-coding genes in the SigB regulon that are of particular interest for further investigation.

Activation of SigB involves the same primary partner switching mechanism in *S*. *aureus* and *B*. *subtilis* where SigB is released from its inhibitory complex with the anti-sigma factor RsbW by the competitive binding of the unphosphorylated form of the antagonist protein RsbV to RsbW [76]. However, the two regulatory cascades leading to RsbV dephosphorylation in conditions of energy stress (via phosphatase RsbP) or environmental stress (via phosphatase RsbU) in *B*. *subtilis* are not conserved in *S*. *aureus* whose genome encodes neither RsbP nor the stressosome that controls the activity of RsbU [77]. How and if the activity of SigB is regulated in *S*. *aureus* is still not well understood. RsbU may be constitutively active making SigB activity sensitive to the availability of core RNA polymerase [78]. Our data show fundamental differences in the expression patterns of SigB-dependent TUs in *S*. *aureus* compared to *B*. *subtilis*. Unlike in *B*. *subtilis*, the induction of SigB-dependent promoters in *S*. *aureus* is not restricted to a narrow range of stress conditions but is instead generally associated with the stationary phase and perhaps a general redistribution of RNA-polymerase activity. Indeed, the average expression level downstream of SigB-dependent promoters was higher than for SigA-dependent promoters in half of the conditions of our study. In addition, the *S*. *aureus* SigB regulon exhibits a substantial basal activity in non-induced conditions not seen in *B*. *subtilis* where SigB-dependent promoters are strictly turned off in the absence of stress. It was also interesting to note that while genes of the SigB regulon grouped in a single specific expression cluster in *B*. *subtilis*, this was not the case in *S*. *aureus* where SigB-regulated genes exhibited a substantial diversity of expression patterns, some of which very similar to genes transcribed from SigA-dependent promoters. Altogether, these differences are certainly associated with distinct physiological roles of SigB in the two species as also suggested by the widely different gene content.

### Identification of a potential Fur-dependent sRNA

In addition to searching for so far unknown SigB controlled genes and new insights into the regulatory role of SigB, we exploited our tiling array data set with respect to the prediction of new transcription factor target genes including the iron-responsive regulator Fur. This analysis identified an sRNA (S596) supposed to be controlled by the Fur repressor, which is apparently a functional analog of FsrA/RyhB. It is predicted to repress, amongst others, the expression of genes encoding iron-sulfur-cluster containing proteins (*citB*, *fdhA*, *addB*, *ilvD*, and *miaB*) and heme biosynthesis enzymes (*ctaA* and *hemE*) as well as of tricarboxylic acid (TCA) cycle genes *citZ* (encoding citrate synthase) and *sdhCA* (encoding succinate dehydrogenase subunits). Iron-responsive sRNAs exist in many bacteria including *B*. *subtilis* [79] and typically mediate an iron-sparing response including the down-regulation of TCA cycle genes as first described for *E*. *coli* RyhB [80]. In *S*. *aureus*, Fur-mediated regulation was also shown to involve changes in the levels of central carbon metabolism proteins; three TCA cycle enzymes (aconitase CitB, succinate dehydrogenase subunit SdhA, and fumarase CitG) were observed in lower amounts under conditions of iron starvation [81]. In addition, iron-responsive sRNAs are involved in regulatory processes beyond iron homeostasis, particularly in the regulation of virulence-associated genes in Gram-negative pathogenic bacteria (reviewed in [72]).

### Expression patterns during *in vitro* infection conditions

Our data highlight the diversity of expression patterns for virulence related genes across conditions. This observation reflects the functional heterogeneity of this pool of genes that encompasses biological functions such as cell adhesion, toxin production, and escape of the immune system. However, for the proteins classified as virulence factors, which often have multiple roles in pathogenesis, the knowledge of the exact function and regulation of their encoding genes is not always complete. Indeed, a complex regulatory network mediates the adaptation of *S*. *aureus* to the host during colonization and infection. In particular, expression of virulence factor genes is tightly controlled by multiple regulatory systems involving two-component systems (SaeRS, Agr, ArlRS, LytRS, SrrAB), transcription factors (SarA family regulators, Rot, MgrA, CodY), the alternative sigma factor SigB and sRNAs (RNAIII, SprD, RsaA) [82,64]. Within this regulatory network, the SaeRS system controls the expression of numerous genes, such as those encoding adhesins and toxins [83,84] and its impact on virulence gene expression during *in vivo* infection has recently been characterized [85]. The host signals known to activate the SaeRS system include H_2_O_2_ and sub-inhibitory concentrations of α-defensins [86].

The global view on the condition-dependent expression of virulence related genes pointed to interesting observations. A noticeably high number of virulence factor genes (37/47), in particular SaeRS-dependent genes, were upregulated after internalization by human THP-1 macrophages and S9 bronchial epithelial cells, including *eap*, *fnbA* and *fnbB* (Fig 2B, S4 Table). Extracellular adherence protein (Eap) and fibronectin-binding proteins (FnbA, FnbB) are major determinants facilitating invasion of host cells [87]. The *eap* gene was among the genes most highly expressed in internalized staphylococci. Several genes involved in evasion of host immune responses, in particular complement-mediated phagocytosis [88], were most highly expressed during growth of *S*. *aureus* in human plasma: *spa* encoding the immunoglobulin binding Protein A and four SaeRS-dependent genes (*efb*, *sbi*, *chp*, and *scn*). The Extracellular fibrinogen-binding protein (Efb) interacts with complement protein C3b and attracts fibrinogen to the surface of *S*. *aureus*, thus masking C3b and opsonizing antibodies from recognition by phagocytic receptors [89]. It was shown to block phagocytosis in plasma and in human whole blood. The secreted proteins Sbi, CHIPS, and SCIN are also required for survival of *S*. *aureus* in human blood [90,91]. The finding that *efb*, *sbi*, *chp*, and *scn* showed a partially different expression pattern compared to other SaeRS-dependent genes reflects the expression of virulence factors in adaptation to the host environment, suggesting the involvement of additional regulators.

A recent cell culture infection study revealed the intracellular replication of *S*. *aureus* HG001 inside eukaryotic cells, using the human lung epithelial cell lines A549 and S9 and the kidney cell line HEK 293 [92]. In agreement with this result, overall gene expression patterns observed in the present study suggest that the physiological state of *S*. *aureus* internalized by human THP-1 macrophages or S9 bronchial epithelial cells (analyzed up to 6.5 hours after infection) is comparable to growing rather than stationary phase cells. More specifically, our data revealed that *S*. *aureus* is challenged with reduced amino acid availability and iron limitation upon internalization by these eukaryotic cells. Furthermore, the PCA results (rmse and correlation coefficients for axis 7, see Figure B in S1 Data and S3 Table) pointed to the upregulation of transporter genes as already observed during internalization of *S*. *aureus* by A549 cells [93], in particular for glycerol, glucose-6-phosphate and phosphate (the latter specifically after internalization by S9 cells).

From the expression of reference genes (moderate level of induction of *ldh1*, Fig 2B) it also appears that *S*. *aureus* cells are faced with reduced oxygen availability upon internalization by THP-1 macrophages. In line with this observation, the proteome study by Surmann *et al*. [92] reported elevated levels of fermentation enzymes and the CydAB terminal oxidase in *S*. *aureus* HG001 internalized by eukaryotic cells. These responses indicative of a microaerobic environment inside host cells were, however, different among bacteria internalized by different cell lines; protein levels were significantly less increased during adaptation to S9 cells as compared to A549 and HEK cells.

### Responses to antibiotics at sub-inhibitory concentrations

Previous studies suggested that antibiotics at sub-lethal concentrations can drive the development of resistant phenotypes [94]. In this context, it is interesting that the wide range of clinically relevant antibiotics that we tested (clindamycin, erythromycin, linezolid, TMP-SMX, vancomycin, ciprofloxacin, and flucloxacillin) evoked only very few significant transcriptional responses at sub-inhibitory levels (S4 Table). In fact, the *norA* gene, which confers low-level resistance to fluoroquinolones [95,96] was even found to be down-regulated by ciprofloxacin. The latter is in line with previous findings where *S*. *aureus* was challenged with a lethal dose of ciprofloxacin [97]. Furthermore, none of the tested antibiotics caused enhanced transcription of genes for known drug efflux pumps of *S*. *aureus*, including *norA*, *norB*, *norC*, *mepA*, *mdeA*, *sepA*, *sdrM* and *lmrS*. These transporters were previously found to be upregulated in response to antibiotic challenges, and some were shown to play a role in drug resistance [98].

Nevertheless, the fluoroquinolone ciprofloxacin and the β-lactam antibiotic flucloxacillin did induce some changes in gene expression (Figure N in S1 Data, S4 Table). Of note, the DNA-gyrase inhibitor ciprofloxacin induced transcription of various phage-encoded genes, which is consistent with previous observations where *S*. *aureus* was challenged with lethal concentrations of this antibiotic [97,99]. In contrast, in response to sub-inhibitory concentrations of flucloxacillin, phage 11 genes were down-regulated during stationary phase, and under the same condition, the transcription of several metabolic operons was significantly increased. In the presence of a sub-lethal ciprofloxacin dose, all structural genes of phage 11 were highly induced during exponential growth, which suggests the activation of this lysogenic phage. In addition, part of the genes of phage 12 and of the *hlb*-converting phage 13 was induced under this condition. The induction of phages is apparently linked to the DNA damage-induced SOS response [99], since increased transcription of *recA* and *lexA* was also observed. In *E*. *coli*, activated RecA promotes the autoproteolysis of LexA, resulting in derepression of DNA-repair functions and inactivation of the phage lambda repressor cI [100]. Our findings show for the first time that also sub-inhibitory concentrations of this antibiotic trigger the SOS response, leading to prophage induction. In fact, because phages are effective facilitators of horizontal gene transfer [101–103], induction of prophages *via* the SOS response might be one of the mechanism by which sub-lethal doses of antibiotics contribute to the evolution of resistances [94].

### Limited antisense transcription in the *S*. *aureus* wild-type

ASRNAs are less frequent and less often initiated from their own promoter (categories Indep and Indep-NT) in *S*. *aureus* than in *B*. *subtilis* where many ASRNAs were highly expressed only under specific conditions, such as sporulation or physical stresses. A detailed analysis of the composition of the two ASRNA repertoires revealed the origin of the difference: The smaller contribution of alternative sigma factors is the reason for the lower amount of ASRNAs and in particular ASRNAs belonging to the Indep and Indep-NT categories. Importantly, the lower number of ASRNAs detected in *S*. *aureus* cannot be interpreted as only a simple consequence of the higher number of alternative sigma factors in *B*. *subtilis* or the larger number of experimental conditions tested. Indeed, although the SigB regulon was expressed at high level in many of our experimental conditions, the rate of SigB-dependent ASRNAs per Mbp was markedly lower in *S*. *aureus* than in *B*. *subtilis*, whereas the rate of SigA-dependent ASRNAs was quite comparable. Consequently, the small number of 19 “independent” ASRNAs in *S*. *aureus* can be considered consistent with our initial interpretation that many *B*. *subtilis* ASRNAs may be byproducts of an extensive use of alternative sigma factors for condition-specific promoter recognition prone to generate spurious (non-functional) transcripts without major negative impact on fitness [13]. This potential source of ASRNAs is in fact largely absent in *S*. *aureus* where SigB and SigH (which is not active in the strain used [65]) are the only relevant alternative sigma factors and control of SigB activity appears less stringent than in *B*. *subtilis*.

### Role of the termination factor Rho in suppressing antisense transcription

The global, *i*.*e*. genome-wide, cellular function of Rho has been almost exclusively studied in *E*. *coli* [104–106] in which it accounts for 20–50% of the termination sites [105] and has also been found involved in distinct regulatory mechanisms [107,108]. In *S*. *aureus*, we identified only 19 TUs with heterogeneous and often antisense 3’-extensions due to the complete absence of a defined termination site. Our results confirmed the Rho-dependence of this particular class of TUs already identified in *B*. *subtilis* [13]. Beyond these, a very limited number of TUs exhibited Rho-dependent termination resulting in read-through transcription and extensions beyond the transcript 3’-ends in the absence of Rho. Termination of protein-coding genes in *S*. *aureus* is therefore mostly independent of Rho.

Nevertheless, a massive up-regulation of expression on the antisense strand of the protein-coding genes was found in an isogenic mutant deficient for the termination factor Rho, which substantiates the role of Rho in suppressing pervasive antisense transcription initiation. Previous studies in *B*. *subtilis* and *E*. *coli* had already revealed that Rho plays a major role in suppressing or limiting antisense transcription but Rho-dependent transcripts initiated from promoters undetected in control conditions represented only a limited number of loci [105,13]. Indeed, in these studies Rho was shown to predominantly act on a subset of TUs possessing Rho-dependent terminators. Furthermore, in *B*. *subtilis*, where Rho is also dispensable [45], the impact of Rho deletion appeared comparatively limited to a much smaller number of loci (93 chromosomal regions comprising 367 genes expressed only in absence of Rho) and resulted mainly from 3’-extensions of TUs with respect to the parental strain [13]. In *E*. *coli*, where *rho* is an essential gene, Rho-dependent transcripts were mapped by treatment with the Rho inhibitor bicyclomycin. Antisense transcription suppressed by Rho was identified for 1555 genes (34% of all genes) and was shown to arise mostly (~60%) from continuation of transcription at the end of genes into oppositely oriented downstream genes, but a substantial fraction (~40%) was also generated by transcription from antisense promoters within genes, i.e. by extension of *bona fide* antisense RNAs [105].

The pattern of antisense transcription observed in *S*. *aureus* in absence of Rho is therefore very different from what was reported from *E*. *coli* and *B*. *subtilis*. Our comparison of the frequency of canonical promoter -10 box hexamer TATAAT between *S*. *aureus*, *B*. *subtilis* and *E*. *coli* suggests that pervasive antisense transcription from cryptic low level promoters may be directly linked to the higher A+T composition of the *S*. *aureus* genome. The precise mode of action of Rho has not been studied in Gram-positive bacteria, but what is known from Gram-negative bacteria suggests that Rho is particularly well suited to terminate transcription of ASRNAs since the absence of ribosomes may facilitate its loading and contact with the elongation complex. Interestingly, pervasive transcription initiation certainly also occurs on the sense strand [17] and probably also leads to untranslated transcripts. Nevertheless, we showed that *rho* deletion had less impact on the expression of the sense strand. The higher abundance of the TATAAT hexamer on the antisense strand could at least partly explain why Rho-dependent termination seems to target mostly antisense transcripts in *S*. *aureus*.

### Effects of antisense transcription in the absence of Rho

In our experiments, considerably elevated antisense transcription was not associated with a strong growth inhibitory effect. In general, antisense transcription can have deleterious effects by changing the expression of the overlapped genes and by diverting cellular resources. With regard to the first aspect, a role in regulating the expression of their opposite genes has been established for a number of individual ASRNAs and can involve different mechanisms: transcription interference, transcription attenuation, modulation of mRNA degradation and ribosome binding (for review, see [15,109]). Peters *et al*. [105] reported for *E*. *coli* that the increase in antisense transcription caused by the inhibition of Rho did not affect sense transcription. In contrast, our study revealed an effect of elevated antisense transcription on sense transcript levels in Rho-deficient conditions in *S*. *aureus*. Moreover, the mutant antisense levels of down-regulated genes were found to be mostly higher than the sense levels detected in the wild-type (Fig 4). This observation would be in line with a stoichiometric mechanism of destabilization of the sense transcripts by pairing with their antisense transcripts and degradation of the double-stranded products [74]. We also noticed that the most highly expressed genes showed the lowest level of antisense transcription. This second observation could result from the same mechanism, i.e. low level of antisense caused by degradation of the antisense after pairing with the sense transcript.

Importantly, these results disclosing the context in which antisense transcription impacts on sense transcription directly suggest an explanation for the observation that *rho* deletion has no clear effect on the growth rate despite its role in control of pervasive antisense transcription: The genes whose expression tends to be higher (in particular housekeeping genes) are less impacted by antisense transcription.

### Concluding remarks

Our results obtained on wild-type and Rho-deficient *S*. *aureus* and the direct comparison to those obtained with the same technology on *B*. *subtilis* reveal additional pieces of information on the biological interpretation of pervasive antisense transcripts. Indeed, the data tend to indicate that the pattern of pervasive antisense transcription may be a simple by-product of genome and transcriptome characteristics such as: A+T-composition, multiplicity of promoter motifs recognized by sigma factors, and balance between the evolutionary forces of mutation, drift and negative selection whose stringency is presumably lower for condition-dependent promoters. From such a perspective, pervasive antisense transcription would not be the product of an evolution for a biological function either at the individual level (e.g. *cis*-regulation of sense gene) or at the global level. Furthermore, the data also disambiguate the notions of biological effect and biological function, since we show that pervasive transcription although certainly spurious would have an effect on sense transcription if not suppressed by specific mechanisms such as Rho-dependent termination.

In the context of *S*. *aureus* research, the present study has provided a detailed inventory of transcription units and non-coding RNAs of *S*. *aureus* HG001, along with a classification of SigA- and SigB-dependent promoters, and targets for major transcription factors. It is anticipated that this compendium, which can be queried through an online genome browser, will serve as a major lead for future studies on this important pathogen’s very diverse lifestyles inside and around the human host.

## Materials and Methods

### Growth conditions, cell culture infection models and mutant construction

*S*. *aureus* was grown with shaking at 37°C in the various cultivation media and in human plasma. Samples were collected by centrifugation with half volume of killing buffer (20 mM Tris pH 7.5, 5 mM MgCl_2_, 20 mM NaN_3_). Pellets were frozen in liquid nitrogen and stored at -80°C until RNA preparation. The details of the medium compositions are provided in the supplemental material (Text A in S1 Data). Briefly, CDM (chemically defined medium) contains inorganic salts, glucose, citrate, FeCl_3_, trace elements, vitamins and 2 mM of all 20 proteinogenic amino acids [110]. The cell culture media RPMI 1640 and MEM are synthetic media commonly used for the cultivation of eukaryotic cells that are also used for the pre-cultivation of bacteria in infection studies. The adapted cell culture medium pMEM supporting growth of *S*. *aureus* consists of HEPES-buffered MEM and contains amino acids in concentrations of ≥ 2 mM, except for asparagine (0.1 mM) and methionine (1 mM) [111].

Sub-inhibitory antibiotic concentrations were as follows: flucloxacillin 0.02 μg/mL, vancomycin 0.63 μg/mL, ciprofloxacin 0.10 μg/mL, clindamycin 0.01 μg/mL, erythromycin 0.05 μg/mL, linezolid 0.10 μg/mL, and trimethoprim-sulfamethoxazole 0.75 μg/mL. In these experiments, *S*. *aureus* was pre-cultured in TSB till early-exponential phase (OD_600_ of 0.5), after which the culture was diluted 10-fold using TSB supplemented with the respective antibiotics.

For the experiments with S9 cells (details in Text A & B in S1 Data), exponentially growing bacteria were diluted with MEM supplemented with 4% FCS thereby adjusting a MOI of 25. After adding the bacteria to the host cell layer, cell culture plates were incubated for 1 hour at 37°C and 5% CO_2_ in a humidified incubator. During this time bacteria were internalized by the S9 cells. After 1 hour of infection, lysostaphin was added to kill remaining extracellular bacteria. After 1.5 and 5.5 hours of incubation (2.5 and 6.5 hours post infection) samples were taken and internalized staphylococci were isolated for RNA preparation. For the experiments with THP-1 cells, bacteria were pre-grown in RPMI medium with FCS, added to the host cells and incubated at 37°C and 5% CO_2_ in a humidified incubator.

The Δ*rho* mutant was constructed according to the procedure described by Arnaud *et al*. [112] (Text C in S1 Data).

### RNA preparation and tiling array hybridization

Total RNA was prepared by acid-phenol extraction after mechanical cell disruption as described previously [13]. The tiling array used in this study contains 383,452 probes covering both strands of the *S*. *aureus* NCTC 8325 genome with a tiling step of 18 nucleotides [113]. Synthesis and hybridization of labeled cDNA were carried out at Roche NimbleGen (Madison, WI) using the strand-specific method described before [114]. All experiments were performed in triplicate with RNA isolated from independent cultures.

Tiling array data have been deposited in the NCBI’s Gene Expression Omnibus (GEO) database (accession numbers GSE70040-43).

### Northern blot analysis

Northern blot analysis was carried out as described previously [115]. The digoxigenin-labeled RNA probes were synthesized by *in vitro* transcription (IVT) with T7 RNA polymerase and gene-specific PCR products as template. Primer sequences are listed in Table A in S1 Data. 5 μg of total RNA per lane were separated on 1.2% agarose gels. Chemiluminescence signals were detected using a ChemoCam Imager (Intas Science Image Instruments, Göttingen, Germany). For sRNA analysis 2% agarose gels were used and fluorescent detection of biotin labeled probes was performed with the Odyssey CLx Imager according to the instructions of the manufacturer (LI-COR Biosciences, Lincoln, NE, USA).

### Tiling array data analysis

Detection of new transcribed segments, 5’-ends (signal up-shifts), 3’-ends (signal down-shifts), delineation of TUs, and computation of gene expression values, were performed exactly as described for *B*. *subtilis* [13]; the main steps of these analyses are summarized below. For each hybridization, the log2 of the raw signal intensity along the genome was analyzed with HMMtiling [116] to obtain a smoothed trajectory and the positions of predicted breakpoints, accounting for random noise and differences in probe affinity as characterized by hybridization of genomic DNA [117]. The repertoires of up-shifts and down-shifts corresponding to probable transcript 5’-ends and 3’-ends were established by combining high-confidence breakpoints (credibility cut-off 0.99) from the different hybridizations. The precision in the mapping of up-shifts was determined previously [13] by a comparison of the up-shift positions detected in *B*. *subtilis* with transcription start sites mapped by a differential RNA-seq approach [118], which revealed that the resolution is within the range of the tiling step with a tendency to have up-shifts slightly upstream (median of 12 bp in *B*. *subtilis*) of the actual transcription start site. New transcripts (i.e. unannotated in GenBank: CP000253) were detected where the lower boundary of the 95% equal-tailed credibility interval for the smoothed trajectory reached 10-fold the chromosome median. These segments were further split according to breakpoints to avoid aggregation of regions with different transcription levels and extended on their 5’ and 3’ extremities according to a more permissive expression cut-off (5-fold the chromosome median) to avoid over-segmentation in regions with expression levels close to the 10-fold cut-off. Promoter activity was measured as the smoothed signal downstream the position of the corresponding up-shift and a promoter tree was built by hierarchical clustering based on estimated pairwise correlation coefficients. Downstream each up-shift, a transcription unit (TU) was defined as the maximum continuous region where the expression level remains at least 5-fold higher than the chromosome median before dropping below this level or encountering another up-shift in at least one RNA sample. Aggregated log2-scale expression values for annotated genes and newly detected segments were obtained as the median of the smoothed signal for probes lying entirely within the considered region. For most analyses gene-level values were quantile-normalized to make them more comparable between hybridizations.

A detailed description of the methods used to analyze the Δ*rho*-mutant data is available in Text D in S1 Data. These included probe-level normalization and differential expression analysis to detect up-regulated and down-regulated regions. Briefly, normalization of whole-genome transcription profiles was performed by fitting a quantile-normalization transformation on the aggregated expression values computed for the repertoire of expression segments and applying this transformation on the smoothed probe-level data genome-wide. Contiguous probes exhibiting up- or down-regulation at the specified fold-change and false discovery rate cut-offs were reported as differentially expressed regions.

### Sequence analysis of transcript 5’-ends and 3’-ends

The classification of promoters based on the presence of sigma factor binding site motifs around the up-shifts (-60bp, +40bp) was performed with the software ‘treemm’ [13] that also takes into account expression profiles across hybridizations. This analysis allowed positioning of the sigma factor binding sites with bp-level resolution. Other transcription factor binding sites (TFBS) were searched with MAST v4.9.0 [119] using position weight matrices built from known binding sites. For this purpose, we retrieved from the RegPrecise database v2.1 [68] the manually curated binding sites across *Staphylococcaceae* for the transcription factors listed for *S*. *aureus* N315. For WalR and GraR, not present in RegPrecise, we used the binding sites listed in [113,120,121]. The resulting position weight matrices were used as queries in MAST searches against the database of the sequences around the up-shifts (-100bp, +50bp; E-value cut-off 1). In keeping with the orientations of binding sites listed for *S*. *aureus* in RegPrecise we considered only the occurrences with the same orientation as the up-shifts, except for WalR for which we took both strands into account. We compared the position of signal down-shifts to predictions of intrinsic terminators available at http://transterm.cbcb.umd.edu/tt/ that were made from the sequence alone with the software TranstermHP [48].

### Data availability

The *S*. *aureus* Expression Data Browser at http://genome.jouy.inra.fr/aeb/ can be accessed through the study website http://genome.jouy.inra.fr/aeb/supplementary_data.html. The website also provides links to access the data sets deposited in GEO (records GSE70040-43).

## Supporting Information

S1 Data This file contains Supplementary Methods and Results (Text A—J); Supplementary Figures A—N; and Supplementary Tables A—E. (PDF)

S1 Fig Barplots of expression profiles of 47 virulence-associated genes and selected reference genes for limitations in amino acids, iron or oxygen. Gene expression levels (normalized log2 intensities) are displayed for the following conditions: exponential growth and stationary phase in different cultivation media and human plasma (blue bars); growth in the presence of sub-inhibitory concentrations of various antibiotics in TSB (red bars) and RPMI medium (orange bars); internalization of *S*. *aureus* by S9 bronchial epithelial cells or THP-1 macrophages and related conditions, i.e. 2.5 hours of anaerobic incubation in pMEM medium at 37°C, 1 hour of incubation in the infection medium at 37°C and 5% CO_2_ without agitation, and non-adherent bacteria retrieved from the supernatant of S9 cells after 1 hour of infection (green bars).
(PDF)

S1 Table Expression levels for all annotated genes and new RNA features in the 156 RNA samples. (XLSX)

S2 Table Least and most expressed genes in all conditions. (XLSX)

S3 Table Correlation coefficients and loadings associated with PCA axes 1–15. (XLSX)

S4 Table Differential expression analysis of infection-mimicking and antibiotic conditions. (XLSX)

S5 Table List of promoter up-shifts with cluster information and TU definition. (XLSX)

S6 Table Information summary for each annotated gene and new RNA feature. (XLSX)

S7 Table List of high confidence down-shifts and effects of *rho* deletion at termination sites. (XLSX)

S8 Table List of promoter up-shifts with transcription factor binding site information. (XLSX)

S9 Table Transcription factor regulons including previously known and newly identified potential target genes. (XLSX)

S10 Table List of up-regulated regions in the Δ*rho* mutant. (XLSX)

S11 Table List of genes showing differential expression in the Δ*rho* mutant compared to the wild-type. (XLSX)
