# Supplementary material for: Staphylococcus aureus Transcriptome Architecture: From Laboratory to Infection-Mimicking Conditions
Source: PLoS Genet. 2016 Apr 1;12(4):e1005962. doi: 10.1371/journal.pgen.1005962 (PMC4818034; doi:10.1371/journal.pgen.1005962)
Supplement: S1 Data — (PDF) [file pgen.1005962.s001.pdf]

S1 Data for

Staphylococcus aureus transcriptome architecture: from laboratory to infection-mimicking conditions

Ulrike Mäder[1¶], Pierre Nicolas[2¶], Maren Depke[1], Jan Pané-Farré[3], Michel Debarbouille[4], Magdalena M. van der Kooi-Pol[5], Cyprien Guérin[2], Sandra Dérozier[2], Aurelia Hiron[4], Hanne Jarmer[6], Aurélie Leduc[2], Stephan Michalik[1], Ewoud Reilman[5], Marc Schaffer[1], Frank Schmidt[1], Philippe Bessières[2], Philippe Noirot[7], Michael Hecker[3], Tarek Msadek[4], Uwe Völker[1*] and Jan Maarten van Dijl[5*]

[1] Interfaculty Institute for Genetics and Functional Genomics, University Medicine Greifswald, Greifswald, Germany.

[2] MaIAGE, INRA, Université Paris-Saclay, Jouy-en-Josas, France.

[3] Institute for Microbiology, Ernst-Moritz-Arndt-University Greifswald, Greifswald, Germany.

[4] Biology of Gram-Positive Pathogens, Department of Microbiology, Institut Pasteur and CNRS ERL 3526, Paris, France.

[5] Department of Medical Microbiology, University of Groningen, University Medical Center Groningen, Groningen, The Netherlands.

[6] Center for Biological Sequence Analysis, Department of Systems Biology, Technical University of Denmark, Kongens Lyngby, Denmark.

[7] Institut Micalis, UMR1319/INRA-AgroParisTech, INRA, Jouy-en-Josas, France.

* Corresponding authors

E-mail: voelker@uni-greifswald.de (UV); j.m.van.dijl01@umcg.nl (JMvD)

[¶] These authors contributed equally to this work.

S1 Data Index

Supplementary Text

Text A	Growth conditions
Text B	Preparation of internalized *S. aureus* from host cells, control sample harvest, and RNA preparation from internalized and control samples
Text C	Construction of the ∆*rho* mutant
Text D	Analysis of the ∆*rho*-mutant data
Text E	Principal Component Analysis (PCA) and computation of the associated root mean squared errors
Text F	Curation and classification of RNA features
Text G	Mapping the known non-coding RNAs of strain N315 onto the NCTC 8325 genome and comparison with the RNA features identified by this study
Text H	Identification of new potential *trans*-encoded small regulatory RNAs (sRNAs)
Text I	Analysis of SigB promoter sequences
Text J	Newly predicted Fur-regulated promoters of iron/trace metal transporter genes

Supplementary Figures

Figure A	Growth of *S. aureus* HG001 in different cultivation media and human plasma
Figure B	PCA axes 1 to 15: rmse‘s and coordinates of the biological conditions
Figure C	Distribution of median and maximum expression levels for different categories of RNA features
Figure D	Expression patterns of newly identified potential sRNAs
Figure E	Northern blot analysis of newly identified potential sRNAs
Figure F	Activity profiles of promoter clusters associated with SigB (B7, 8, and 10) and main transcription factor regulons (CcpA, CodY, Fur, and Rex)
Figure G	Consensus motifs of SigB-promoters of promoter clusters B7, B8, and B10
Figure H	Region of the *S. aureus* chromosome covering the potential sRNA S596
Figure I	Distance between transcription down-shifts and predicted intrinsic transcription terminators
Figure J	Growth of *S. aureus* HG001 and its isogenic ∆*rho* mutant
Figure K	Expression levels of *rho* under the different biological conditions

Figure L Comparison of the impact of *rho*-deletion on the expression levels of the sense and antisense strands

Figure M Northern blot analysis of five antisense-generating transcribed regions

Figure N Voronoi-treemap representation of transcriptome changes by sub-inhibitory concentrations of ciprofloxacin and flucloxacillin

Supplementary Tables

Table A Oligonucleotides used for preparation of Northern blot probes

Table B Comparison of potential *trans*-encoded small regulatory RNAs with sRNAs identified by previous studies

Table C Known 5’ *cis*-acting regions of *S. aureus*

Table D Results from previous transcriptome studies in comparison with the SigB regulon revealed by the promoter classification

Table E Target prediction for the potential sRNA S596

Text A Growth conditions

pMEM. The medium pMEM was adapted from the cell culture medium MEM in order to enable bacterial growth with distinct exponential and stationary growth phases. Therefore, the MEM medium was buffered with HEPES for aerobic cultivation and supplemented with 2 mM each of 13 amino acids. The composition of pMEM is listed in the subsequent table.

component	stock concentration	final concentration	volume per 1 l of medium
10x MEM[a]	10x	1x	100 ml
100x NEAA[b,c]	100x	1x	10 ml
200 mM L-glutamine[b,c]	200 mM	4 mM	20 ml
1 M HEPES[c]	1 M	10 mM	10 ml
amino acid mix 1 (Ala, Leu, Ile, Val)[d,e]	50 mM each	2 mM each	40 ml
amino acid mix 2 (Asp, Glu)[d,e]	50 mM each	2 mM each	40 ml
amino acid mix 3 (Ser, Thr, Cys)[d,e], pH 10.3	50 mM each	2 mM each	40 ml
amino acid mix 4 (Pro, His, Phe)[d,e]	50 mM each	2 mM each	40 ml
25 mM tryptophan[d,e]	25 mM	2 mM	80 ml
A. dest.[e]			620 ml
adjust pH to 7.4			

[a] Gibco/Life Technologies; Thermo Fisher Scientific Inc., Waltham, MA, USA
[b] for cell culture
[c] e.g. PAN-Biotech GmbH, Aidenbach, Germany
[d] Sigma-Aldrich Co. LLC., St. Louis, MO, USA; purity > 98%
[e] J. T. Baker, Avantor Performance Materials B. V., Deventer, The Netherlands; MS-grade

RPMI. The following RPMI medium from Gibco was used: 11835 RPMI 1640, no phenol red.

Colistin was added to TSB medium at 50 µg ml^{-1} and cells were grown until an $OD_{600\ nm}$ of 1.

S9 cell culture infection experiment. *S. aureus* HG001 and HG001 pMV158 (expressing GFP) was pre-cultivated over-night in dilution series in pMEM and pMEM/20 µg/ml tetracyclin, respectively. A main culture of pMEM was inoculated from an exponentially growing over-night-culture and cultivated until an optical density at 600 nm (OD_{600}) of 0.4 was reached. Then, an infection mix consisting of bacterial culture and cell culture medium eMEM was assembled. Bacterial culture volume was adjusted to allow infecting confluent S9 cell culture dishes at a multiplicity of infection (MOI) of 25 under consideration of pre-determined host cell numbers per cell culture dish and bacterial colony forming unit (cfu) per ml at OD_{600} of 0.4. Proper buffering conditions for incubation in 5% CO_2 atmosphere were reached by addition of 29 µl of 7.5% sodium hydrogen carbonate per ml of bacterial culture volume in the infection mix. After removal of old cell culture medium, the infection mix was added to the S9 cell culture dishes and incubated at 37°C/5% CO_2 for 1 h. Control samples of non-adherent

bacteria were collected after this first incubation step. The remaining infection mix was replaced by eMEM/10 µg/ml lysostaphin (AMBI PRODUCTS LLC, Lawrence, NY, USA) which killed any extracellular *S. aureus*. After further incubation at 37°C/5% CO_2 for 1.5 h and 5.5 h the samples of the 2.5 h and 6.5 h point in time, respectively, were harvested. In parallel to the cell culture infection, further control samples were generated by incubating the same infection mix in cell culture dishes without host cells 37°C/5% CO_2 for 1 h, 2.5 h, and 6.5 h. For each condition, three biological replicates were sampled.

THP-1 cell culture infection experiment. *S. aureus* HG001 was (pre-)cultured in RPMI medium and used to infect THP-1 macrophages as previously described (Miller *et al.*, 2011). Briefly, THP-1 macrophages were infected with *S. aureus* HG001 at an MOI of 1:50. The macrophages were then incubated with bacteria for 2 h or 6 h. As a control (t = 0 h), *S. aureus* HG001 cells precultured in RPMI were sampled, just before the incubation with THP-1 macrophages. For each condition, three biological replicates were sampled.

Text B Preparation of internalized *S. aureus* from host cells, control sample harvest, and RNA preparation from internalized and control samples

Preparation of internalized staphylococci and harvest of control samples. At the 2.5 h and 6.5 h point in time after host cell infection, the lysostaphin-containing medium was replaced by 1 ml of isotonic killing buffer (20 mM Tris pH 7.5, 5 mM $MgCl_2$, 20 mM NaN_3, 150 mM NaCl). The infected S9 cells were scraped from the culture dish, resuspended and transferred to a 1.5-ml tube for further processing while maintaining the integrity of the majority of host cells. All following steps were performed on ice or at 4°C. Cells were pelleted (600 x g for 5 min) and fixed in ice-cold acetone/ethanol (50% v/v) for 4 min as described by Garzoni et al. (2007) followed by a centrifugation step at 20000 x g for 3 min. The eukaryotic part of the cell pellet was lysed in RLT buffer (Qiagen, Hilden, Germany) and homogenized twice using QIAshredder (Qiagen, Hilden, Germany) and centrifugation at 20000 x g for 2 min. Staphylococci were not lysed during this process although they lost their viability. Therefore, the resulting pellet contained staphylococcal cells and eukaryotic cell debris. Pellets from several infected cell culture dishes processed in parallel were combined and washed once with RLT buffer and four times with TE buffer (10 mM Tris/HCl pH 8, 1 mM EDTA pH 8) to remove residual contaminations by host cell debris. Staphylococcal cell pellets were flash-frozen in liquid nitrogen and stored at −70°C until cell disruption.

Different *S. aureus* control samples (exponential growth phase in pMEM at OD_{600} of 0.4; staphylococci after 1 h, 2.5 h, and 6.5 h of incubation in the infection medium in 5% CO_2-atmosphere at 37°C without agitation, i.e. in cell culture dishes with serum-containing

medium but without presence of host cells; non-adherent staphylococci after 1 h of co-incubation with eukaryotic cells and their products at 37°C/5% CO_2; staphylococci after 2.5 h of anaerobic incubation in pMEM at 37°C) were harvested on ice with addition of at least half a volume of killing buffer (20 mM Tris pH 7.5, 5 mM $MgCl_2$, 20 mM NaN_3). Pellets were flash-frozen in liquid nitrogen and stored at −70°C until cell disruption.

Bacterial cell disruption. The bacterial cell pellet was resuspended on ice in 60 µl killing TE buffer (10 mM Tris/HCl pH 8, 1 mM EDTA pH 8, 20 mM NaN_3) and transferred together with 0.5 ml TRIZOL (Invitrogen, Karlsruhe, Germany) to a liquid nitrogen pre-cooled Teflon vessel. Cells were disintegrated mechanically at 2600 rpm for 2 min in a bead mill (Mikrodismembrator S, B. Braun Biotech International GmbH, Melsungen, Germany; now part of Sartorius AG, Göttingen, Germany). Another 0.5 ml of TRIZOL was added, and the frozen lysate was allowed to thaw. In total, the liquid lysate was incubated at room temperature for 10 min and afterwards flash-frozen in liquid nitrogen and stored −70°C until RNA preparation.

RNA preparation. The RNA preparation with TRIZOL was performed according to the manufacturer's instructions with minor modifications: Briefly, the lysates from bead mill disruption were thawed at room temperature. Chloroform was added (200 µl chloroform / 1 ml TRIZOL), samples were shaken vigorously for 15 s and incubated at room temperature for 5 min. Organic and aqueous phase were separated by centrifugation (12000 x g, 15 min, 4°C), and RNA was precipitated from the aqueous phase with 500 µl isopropanol (2-propanol) / 1 ml TRIZOL overnight at −20°C. After two washes with −20°C pre-cooled 80% ethanol the RNA was dried at room temperature and solved in nuclease-free water (Ambion Inc., Austin, TX, USA, now part of Applied Biosystems, Foster City, CA, USA).

DNase treatment was only possible for control samples because the RNA yield of internalized staphylococci was near the minimum amount needed for tiling array hybridization. Control-RNA-samples were DNase treated and afterwards purified using an RNA Clean-Up and Concentration Kit (Norgen Biotek Corp., Thorold, ON, Canada). The RNA concentration was determined photometrically (NanoDrop ND-1000, NanoDrop Technologies, Wilmington, DE, USA), and the quality was checked with an Agilent 2100 Bioanalyzer (Agilent Technologies, Santa Clara, CA, USA).

Text C Construction of the Δ*rho* mutant

An in-frame deletion mutant of the *rho* gene was constructed in strain HG001 as follows. DNA fragments corresponding to the chromosomal DNA regions located directly upstream

(555 bp) and downstream (598 bp) of the *rho* gene were generated by PCR using oligonucleotide pairs OMD428/OMD429 and OMD430/OMD431, respectively. Oligonucleotides OMD429 and OMD430 were designed with a 24 base complementary overlap, allowing the two generated DNA fragments to be seamlessly fused by strand overlap extension PCR (SOE-PCR; Horton *et al.*, 1989). The purified DNA fragments were mixed in equal amounts and used as a DNA matrix for SOE-PCRs with oligonucleotides OMD428 and OMD431. The full-length DNA fragment was then purified on an agarose gel using the QIAquick Extraction Kit (Qiagen), digested with the appropriate enzymes (BamHI and NcoI), and cloned between the corresponding restriction sites of the thermosensitive shuttle plasmid pMAD (Arnaud *et al.*, 2004). Nucleotide sequence of the construct was confirmed by DNA sequencing and the resulting plasmid passaged through *S. aureus* strain RN4220 and introduced into *S. aureus* HG001. Integration and excision of the pMAD derivatives and deletion of the chromosomal region of interest was carried out as previously described (Arnaud *et al.*, 2004). The *rho* gene deletion was verified by PCR using oligonucleotides OMD435 and OMD436.

OMD428: GGAGGATCCGGGCCAGTAATGTCAGTTATC
OMD429: AGATGTACGTTCTCTTTCAGGCATATAG
OMD430: ATGCCTGAAAGAGAACGTACATCTGAAAGTACTAAAACGGGTCGACCT
OMD431: CCACCATGGGCTTCTTATTCGTGCGATGTT
OMD435: GGTGGTCCTGGTAAACCAGAAGGACTTG
OMD436: CCGGAATGGTAAGTTTCATACATTGAGAG

Text D Analysis of the Δ*rho*-mutant data

Data normalization. The deletion of *rho* induces an increase of the expression outside Genbank annotated regions and new transcription segments detected in the wild-type that impacts a large fraction of the chromosome. After careful examination of the data, we realized that traditional normalization methods such as median or quantile normalization were giving misleading results. In fact, the up-regulation was global enough to significantly shift up-wards the median of the signal along the chromosome. Therefore, the systematic analysis of the up-regulated regions needed a specific normalization procedure. We reasoned that a better option would be to subject to quantile-normalization at first only the signal in the 4028 (Genbank annotation plus our new segments) expression regions characterized in the wild-type and then to apply the sample-specific quantile-normalizing transformation fitted on the coding regions to the whole chromosome. Quantile normalization function was obtained with “limma” R package (Bolstad *et al.*, 2003; Ritchie *et al.*, 2015) and applied to the whole chromosome using the interpolation method implemented in the function

"approxfun" of R package "stats". In practice the tiling array expression signal (log2-scale) as reconstructed with HMMtiling (Nicolas *et al.*, 2009) from the 16 RNA samples (4 growth conditions (RPMI-exp, RPMI-t4, TSB-exp, TSB-t4) x 2 strains (wild-type vs. *rho*-deletion mutant) x 2 biological replicates) were normalized such as to match a common distribution of the aggregated expression levels for the coding regions. The normalized data can be browsed via our website http://genome.jouy.inra.fr/aeb/ and served for all our analyses of this data set.

Building the repertoire of up-regulated regions. Identification of the upregulated regions relied on a differential expression analysis carried out at the probe level on normalized expression profiles along the chromosome. For each probe taken individually, we fitted a linear model (function "lm" in R package "stats") onto the expression signal obtained across the 16 RNA samples. This model incorporated coefficients to capture the differences between the 4 growth conditions as well as the specific effect of the genetic background (*rho*-deletion mutant vs. wild-type) in each condition. In order to account for the number of probes tested, the p-values quantifying the level of statistical support for a non-null effect of the *rho*-deletion in each growth condition were transformed into q-values (R package "fdrtool", Strimmer, 2008). Up-regulated regions were then defined with a two-step procedure: (i) delineation of continuous stretches of probes with an estimated log2 up-regulation coefficient above 2 (4-fold up-regulation) and q-value below 0.05 (thereby controlling the False Discovery Rate at the 5% level) (ii) extension of the 5' and 3' extremities of these regions to encompass also the contiguous probes with log2 up-regulation coefficient above 1 (2-fold up-regulation) and q-value below 0.10. The percentage of probes satisfying simultaneously the 4-fold up-regulation and q-value≤0.05 cut-offs in all regions detected varied between conditions from 44.1% to 69.3% (see Table 2 of the main manuscript).

Mapping of Rho-dependent terminators and classification of the up-regulated regions. To identify Rho-dependent terminators, the levels of the expression signal downstream each of the 1261 high confidence transcription down-shifts identified in the wild-type (156 RNA samples) were compared between the *rho*-mutant and the wild-type in each of the 4 growth conditions tested with the *rho*-deletion mutant. When the expression level upstream the downshift was already higher in the *rho*-mutant we further subtracted the difference measured upstream of the downshift to the difference measured downstream of the downshift. The values are reported in S7 Table. For each condition we then established a list of Rho-dependent terminators as those downshifts for which the expression signal downstream the downshift was at least twice higher in the *rho*-mutant than in the wild-type (after the aforementioned correction when applicable).

In order to dissect the molecular mechanisms that give rise to the up-regulated regions in the *rho*-mutant, we tried to link each up-regulated regions to one of these three possible causal events: (i) inactivation of Rho-dependent terminator, (ii) up-regulation of a coding gene, (iii) presence of segment without defined termination site in the wild-type (NT-segments, described in *B. subtilis* as terminated by the action of Rho). The results are detailed in S9 Table and summarized in Table 2 of the main manuscript. The different cut-offs that served to establish these links are found in the legends of these two tables.

Analysis of the impact of anti-sense upregulation onto sense strand expression. To assess the relationships between sense and anti-sense expression, we computed aggregated expression values for the sense strand and antisense strand of each of the 4028 expression features (Genbank annotated genes and our new segments) across the 16 samples of the *rho*-deletion data set and subjected these values to the same normalizing transformation as the whole chromosome profiles (data presented in S10 Table). Then, we applied to the gene-aggregated sense strand data the same linear model and false discovery rate control procedure as described above for the probe level analysis that served to delineate up-regulated regions (detailed results in S10 Table). In Fig 4B (main manuscript) we represent the relationships between sense expression levels and anti-sense expression levels in the wild-type and *rho*-deletion mutant backgrounds and we highlight those genes whose sense expression level exhibit a statistically significant (q-value≤0.05) two-fold down-regulation. In Figure L we examine separately how the sense and antisense expression levels are impacted by the deletion of *rho*.

Text E Principal Component Analysis (PCA) and computation of the associated root mean squared errors

PCA. The quantile-normalized gene-aggregated expression data (in log2-scale) from the 156 RNA samples have been subjected to PCA. Each RNA sample was seen as a point (156 observations) in space of dimensions equal to the total number of annotated genes and new segments monitored by the tiling array (4028 variables). PCA served to obtain an optimal projection of the observation in spaces of lower dimensions. The variables (individual gene expression values) were centered but not normalized to preserve the differences between genes in terms of amplitude of variation. In practice, the PCA was performed with “prcomp” function of R package “stats” (with options retx=T, center=T, scale=F).

Then we examined: the percentage of the total inertia captured by the successive axes, from 48.9% with 1 axis to 100% with 155 axes (67.3% with 3 axes, 91.7% with 15 axes); the coordinates of the RNA samples in the low-dimension projection spaces (Fig 2A of the main

manuscript, Figure B lower panel); the contribution of the variables (genes and segments) to the definition of each axis (loadings in S3 Table); the correlation between each variable (gene expression level) and each axis (the coordinate of the RNA sample the axis), correlation coefficients are shown in S3 Table. Of note, loadings and correlation coefficients provide complementary rather than redundant information since. A high loading indicates that the variable is important for the definition of the axis (and therefore large amplitude of the variations of this variable along the axis) but it is not necessarily associated with a high coefficient of correlation since the variable may also be linked to other axes. Conversely a high correlation coefficient of a variable with an axis means that the variable is well explained by this axis alone but it can be associated with a low loading when the amplitude of variations for this variable is small compared to other variables. To make loadings easier to interpret, the variable loadings reported in S3 Table are the original loadings (returned in the “rotation” matrix of the “prcomp” object) multiplied by the span of the interval between the two extreme positions occupied by samples on the axis, such as their values correspond to amplitudes explained by the axis (and they can be compared between axes).

Rmse. Each PCA axis is typically associated with condition-specific changes in expression profiles as apparent from the coordinates of the RNA samples on the axes (Fig 2A, Figure B, lower panel). For instance, sample coordinates on axis 1 pull apart stationary phase samples from exponential growth phase samples indicating that axis 1 contributes to capture change in expression profiles that are linked to growth phase.

However it is often not easy to understand, from sample coordinates alone, which is the subset of conditions that each PCA axis contributes to capture. For a more in-depth and quantitative analysis of the relationships between axes and conditions, we also adopted another -less familiar- point of view. Namely, we investigated how the coordinates of a sample on the first k PCA axes allowed reconstructing the centered values of the variables (gene expression levels) with a precision that increases with k. This prediction is obtained by multiplying the matrix of loadings associated with axes (1, …, k) with the vector of coordinates on axes (1, …, k).

Based on this relationship, we computed the root mean squared error (rmse) of the prediction of the expression profile (4028 expression values of the RNA sample) that one could make from the knowledge of the coordinates of the RNA sample on an increasing number of PCA axes (from 0 to 15). These sample-wise rmse’s across samples and PCA dimension (k) can easily be represented and readily point to the PCA axes that contribute to capture each condition-specific profiles (Figure B, upper panel). For instance, the red surface area shows how much rmse’s decreased by taking into account axis 1 in the prediction. From this representation it is clear that axis 1 is important to explain both stationary phase samples

and exponential growth phase samples as anticipated, after the observation mentioned above that sample coordinates on axis 1 correlate with growth phase. In general, the information provided by this analysis proved highly complementary to the analysis of sample coordinates. As an illustration, it is clear that axis 5 contributes much more than axis 4 to capture the specificities of expression profiles obtained in presence of colistin. This was not obvious from the coordinates on axes 4 and 5, since this particular growth condition occupies an extreme position both on axis 4 and on axis 5. Rmse's also provide useful information on which are the conditions associated with expression profiles that are well (or poorly) captured by the PCA, as a function of the number of axes taken into account.

Text F Curation and classification of RNA features

The transcribed regions (TRs) outside of annotated CDSs and RNA genes were decomposed into segments according to the repertoires of transcript ends as described before (Nicolas *et al.*, 2012) and outlined in the main text. After automatic and manual curation, 1192 RNA features (S1 to S1192) were established and classified according to their structural relationship with neighboring genes (S6 Table; Expression Data Browser at http://genome.jouy.inra.fr/aeb/). The different segment categories are described in the main text.

The following color code is used in the figures and the *S. aureus* Expression Data Browser:

- green 5'UTR
- red 3'UTR
- old yellow 3'PT
- orange 3'NT
- dark red 3'ND
- dark blue Intra
- light blue Inter
- black Indep
- brown Indep-NT

Text G Mapping the known non-coding RNAs of strain N315 onto the NCTC 8325 genome and comparison with the RNA features identified by this study

In *S. aureus*, a multitude of regulatory RNAs has been predicted and/or experimentally identified by different approaches including *in silico* predictions (Pichon and Felden, 2005; Geissmann *et al.*, 2009; Marchais *et al.*, 2009), Affymetrix microarrays covering intergenic

regions of *S. aureus* N315 (Roberts *et al.*, 2006), cloning/sequencing of small sized cDNAs (Abu-Qatouseh *et al.*, 2010), and deep sequencing approaches (Bohn *et al.*, 2010; Beaume *et al.*, 2010). Altogether, about 90 of these were confirmed by alternative methods such as Northern Blot analysis or RT-qPCR (reviewed in Felden *et al.*, 2011). With the exception of the study by Abu-Qatouseh *et al.* (2010) analyzing a clinical isolate, strain N315 was chosen for the identification of regulatory RNAs. Expression of *in silico* predicted RNAs was analyzed in different strains (Pichon and Felden, 2005; Geissmann *et al.*, 2009) including in one study *S. aureus* RN6390, a derivative of NCTC 8325-4 (NCTC 8325 cured of the three prophages). However, to the best of our knowledge no systematic identification of RNA features outside annotated CDSs has been performed in the model strain NCTC 8325 so far.

According to Felden *et al.* (2011), the RNA-seq based study by Beaume *et al.* (2010) confirmed almost all non-coding RNAs from earlier studies. We mapped the 186 intergenic transcripts of N315 (BA000018) reported by Beaume *et al.* (2010) onto the NCTC 8325 genome (CP000253). For this task we used blastn algorithm and required a statistically significant hit (E-value≤0.001) with at least 80% of sequence identity and accounting for at least 80% of the length of the query, *i.e.* the Teg regions listed in table S1 of Beaume *et al.* (2010). We then compared the 132 uniquely mapping transcripts with the 1192 RNA features identified by our tiling array study. The results are summarized in S6 Table and Table B. Overall, for 112 of the 132 intergenic transcripts our study detected a counterpart in the NCTC 8325 transcriptome.

Text H Identification of new potential *trans*-encoded small regulatory RNAs (sRNAs)

Our analysis identified 22 potential sRNAs (Table B): Indep or Indep-NT segments which are not ASRNAs or associated with predicted new CDSs and do not represent a *cis*-acting 5'-regulatory region (Table C). Of these, three are generic RNAs (tmRNA, 6S RNA and 4.5S RNA) and 11 correspond to intergenic transcripts of N315 (Beaume *et al.*, 2010) classified as *bona fide* sRNAs. In order to analyze if the remaining eight sRNAs correspond to sRNAs identified by previous studies we mapped the experimentally confirmed sRNAs listed in table S1 of Felden *et al.* (2011) which do not correspond to an intergenic transcript of N315 (Beaume *et al.*, 2010) by BLAST search against the NCTC 8325 genome (CP000253). The sRNA sequences were taken from Bohn *et al.* (2010) (Supplementary Data S1) and Abu-Qatouseh *et al.* (2010) (coordinates given in table 1). The analysis revealed that seven potential sRNAs identified by our study (S35, S204, S414, S736, S774, S808, and S1077) were not identified by any of the previous studies.

Text I Analysis of SigB promoter sequences

Pairwise correlations between promoter activities were summarized in a promoter correlation tree. Associated clustering revealed that 1242 up-shifts (82% of all up-shifts identified) belonged to 16 activity clusters with ≥15 members defined by a cutoff on average Pearson correlation set to 0.6 (see main text). The 145 promoters classified as SigB-dependent by our study form three distinct clusters in the promoter tree: B7 (92 SigB promoters), B8 (12 SigB promoters) and B10 (21 SigB promoters).

In order to compare the sequences of the SigB-dependent promoters (-35 and -10 regions) in the individual promoter clusters, all predicted SigB promoters were manually aligned aiming at a maximal consensus for the GTTTAA (-35) and GGGTAT (-10) hexanucleotides. To accommodate for different spacer lengths, gaps were introduced after the last "A" of the -35 region. A position weight matrix (PWM) was calculated for the -35 and -10 regions of the SigB-promoters in order to estimate the probability for each promoter element. Elements (M) of the PWMs were calculated as follows: $M_{k,j} = \ln(M_{k,j}/b_k)$. The elements were transformed using a background model b (with b = 0.1645 for C or G and 0.3355 for A or T). The probability of a sequence given the PWM was calculated by addition of the relevant probabilities at each position. The analysis revealed that the -35 region of cluster B8 SigB-promoters is less well conserved as compared to clusters B7 and B10. The -10 region of cluster B10 SigB-promoters is less well conserved as compared to clusters B7 and B8.

The frequency of nucleotides within the SigB promoters was visualized as Sequencelogo with the Weblogo tool (http://weblogo.berkeley.edu/logo.cgi) using default parameters (Figure G). The visualization revealed for the -10 region of cluster B10 promoters that in particular conservation of the third "G" is rather weak. Furthermore, for SigB promoters of promoter clusters B8 and B10 a "WW" motif with a preference for "A" over "T" can be observed at positions -4 to -3 relative to the GGGTAT (-10) region. The "WW" motif may function as an extended -10 region in order to accommodate for the less well conserved -35 (B8) and -10 (B10) regions observed for this group of SigB promoters.

Text J Newly predicted Fur-regulated promoters of iron/trace metal transporter genes

Transcription factor binding sites were searched with MAST v4.9.0 using position weight matrices built from known binding sites (see main text). For the ferric uptake regulator Fur, 9 promoters with predicted binding sites were found in cluster B14 (see Text I) together with the majority of known Fur-dependent promoters. Of the 21 annotated genes associated with the 9 promoters, 10 genes are implicated in iron/trace metal transport (*fhuD1*/SAOUHSC_02246, *opp-1* operon, *mntABC*) or iron release from heme

(*isdI*/SAOUHSC_00130). Contrary to previous predictions (Skaar *et al.*, 2004; Sheldon and Heinrichs, 2012), the identified Fur binding site mediating iron-dependent regulation of the *opp-1* genes encoding a transporter for cobalt and nickel (Remy *et al.*, 2013) is located upstream of the co-transcribed *dapF* gene (SAOUHSC_02770). Similarly, the Fur binding site implicated in regulation of the heme oxygenase gene *isdI* is located in the promoter region of the upstream gene of unknown function (SAOUHSC_00131). The MntABC manganese transporter, a potential new member of the *S. aureus* Fur regulon, is known to be controlled by the manganese-responsive MntR repressor. Modulation of manganese homeostasis by iron involving Fur indirectly has already been reported for *B. subtilis* and *Sinorhizobium meliloti* (Helmann, 2014; Platero *et al.*, 2004). It is therefore interesting to note that our data suggests direct regulation by Fur in addition to MntR.

Supplementary Figures

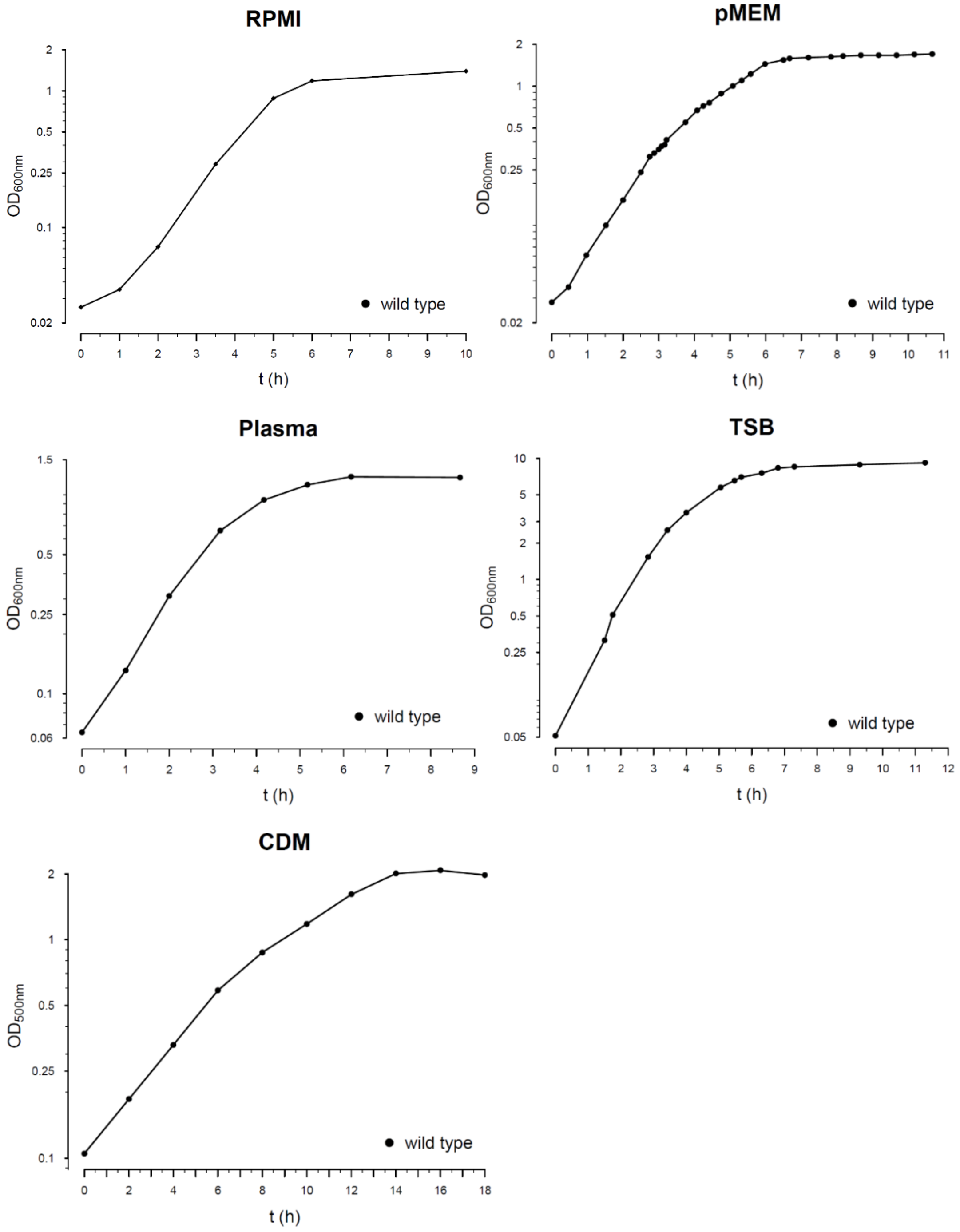

Figure A. Growth of *S. aureus* HG001 in different cultivation media and human plasma.

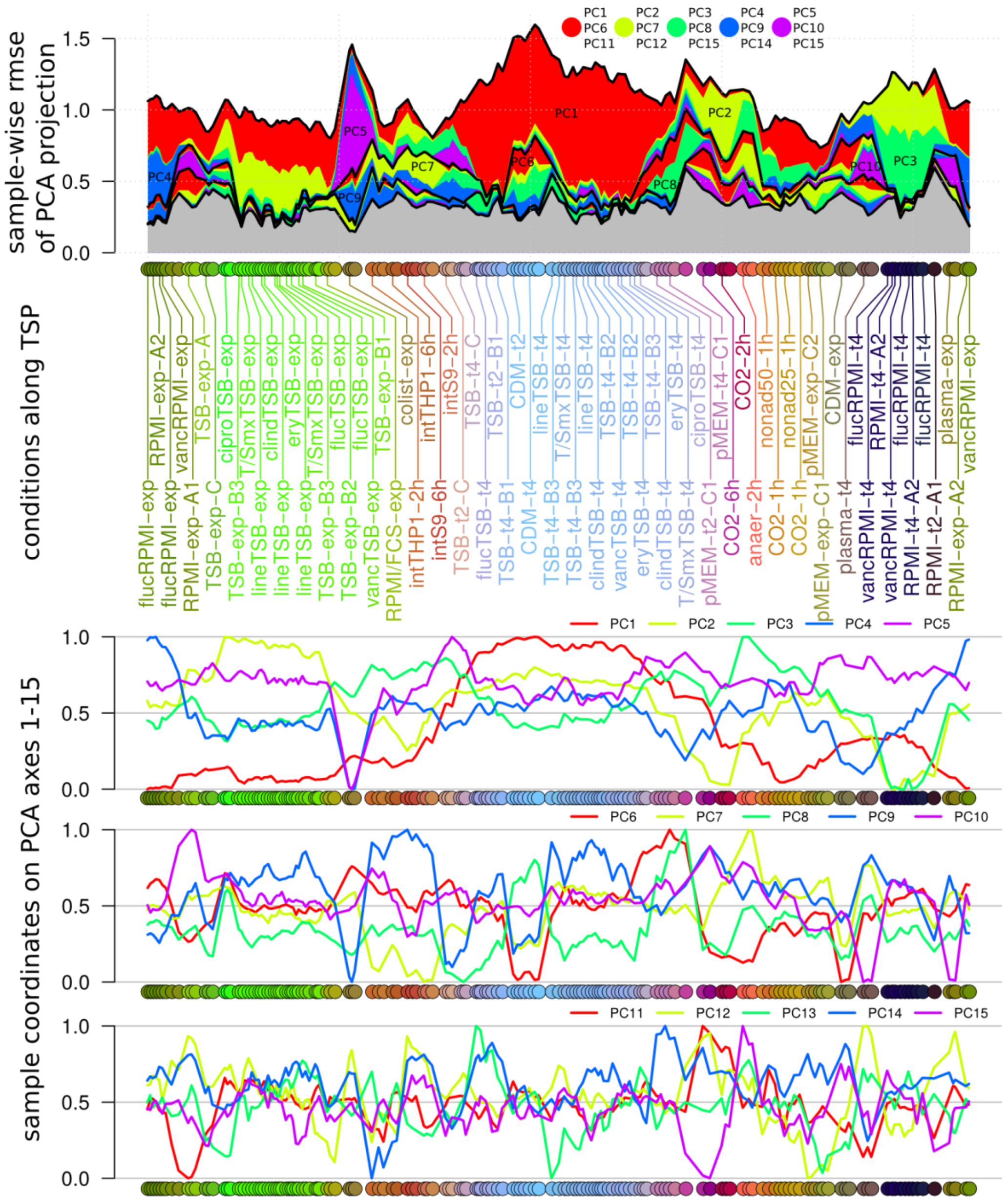

Figure B. PCA axes 1 to 15: rmse's and coordinates of the biological conditions. Upper panel: root mean squared error (rmse) between the gene expression levels as predicted by the coordinates of the sample in the PCA projection and measured gene expression levels. Rmse decreases gradually (predictions becomes more accurate) when the dimension of the PCA projection increases. PCA axes 1-15 are represented by staked colored surfaces whose widths indicate the decrease in rmse associated to each axis. To make the identification of the axis corresponding to each significant surface area easier we recycled 5 different colors 3 times to represent the 15 axes (separating by black lines each 3 group of axes). Middle panel: samples ordered along the TSP. A unique identifier of the biological condition is reported vertically (when consecutive RNA samples arise from the same biological condition only the first biological replicate is labeled). Lower panel: coordinates of samples on the PCA axes 1-15. Here coordinates were rescaled (and shifted) for each axis such as to span exactly the (0,1) interval.

In addition to the first three axes (see main text), other axes of lesser importance in terms of fraction of total variance were also clearly linked to particular biological conditions and/or gene functions. In particular, as pointed out by the analysis of the rmse's, the next two axes contribute to description of exponential growth in RPMI (axis 4) and the effects of colistin (axis 5), whereas axes 7 and 10 seem to capture the modification of the *S. aureus* transcriptome after internalization by eukaryotic cells and in human plasma, respectively.

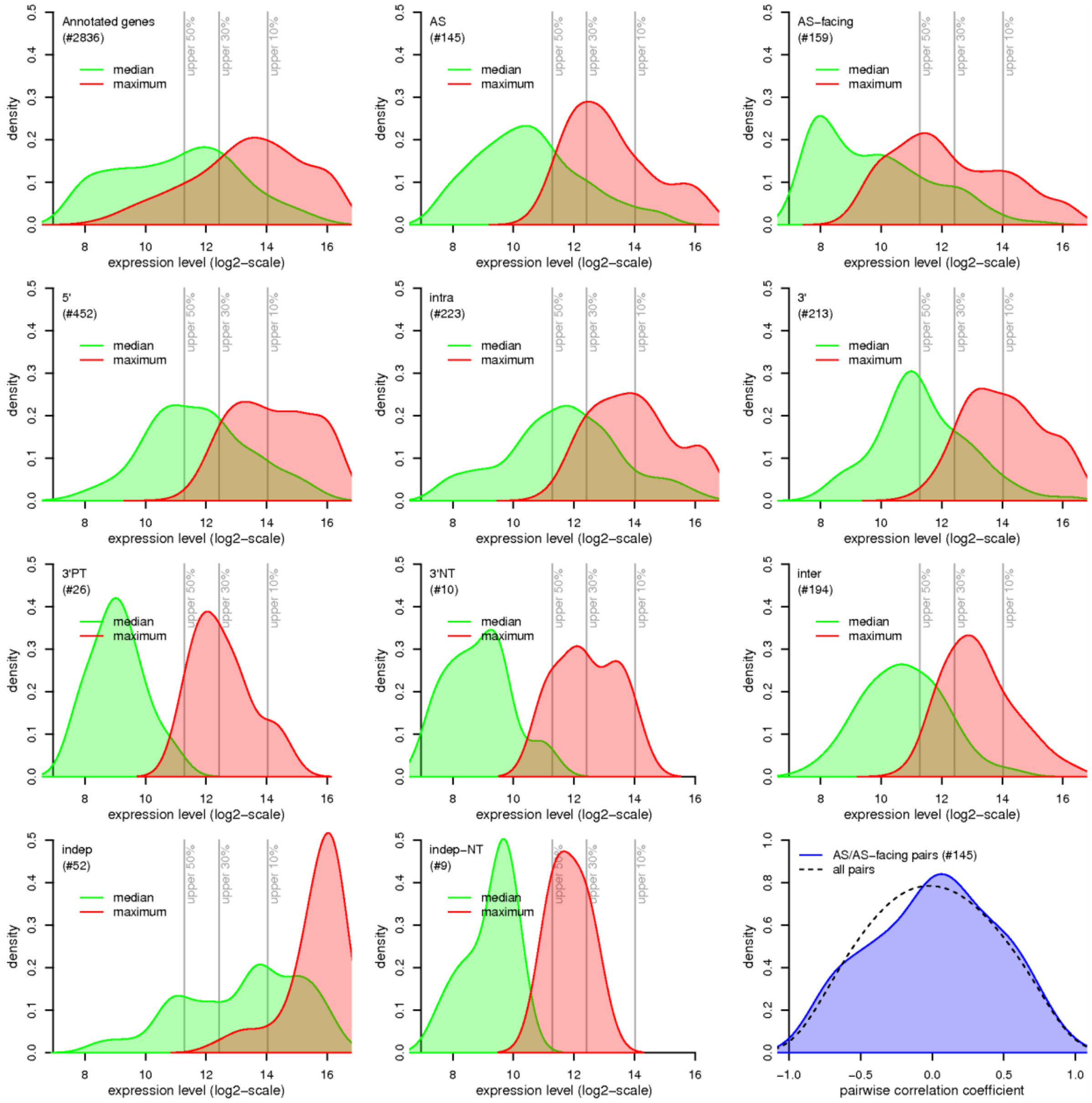

Figure C. Distribution of median and maximum expression levels for different categories of RNA features. For each segment, the median and maximum expression was computed on the quantile-normalized data across the 156 RNA samples available for the wild-type. Then, for each category of segments, non-parametric density estimation was carried out using a Gaussian kernel and bandwidth 0.5 (green for the median, red for the maximum). The number of segments in each category is written between parentheses. Three vertical lines indicate the upper expression quantiles corresponding to 50%, 30%, and 10%, of all the segments in any given RNA sample. As expected from the definitions of the different categories, the distributions of expression levels tend to be similar between the categories “5’”, “intra”, and “3’”. In comparison, the categories “3’PT” and “3’NT” tend to exhibit lower expression levels and “inter” is intermediate. It is also noticeable that expression is much higher for the category “indep” than “indep-NT”. A salient observation is that annotated genes facing ASRNA segments (AS-facing, upper right plot) tend to display lower expression levels than other genes (upper left plot). However there is no detectable trend towards global negative correlation, across conditions, between sense and antisense levels (lower right plot,

bandwidth 0.15). Several factors could contribute to explain the lower expression of genes facing ASRNA segments: (i) it may be that ASRNAs are counter-selected when facing important (and thus highly expressed) genes; (ii) it may also be that ASRNAs destabilize or decrease transcription of the overlapped transcripts; (iii) reciprocally, highly expressed genes may destabilize (or decrease transcription of) ASRNAs. Intriguingly genes facing ASRNA segments were not found to exhibit a lower expression level in *B. subtilis* (Nicolas *et al.*, 2012). This is possibly linked to the highly condition-dependent manner in which many ASRNAs detected in *B. subtilis* are transcribed due to their control by alternative Sigma factors.

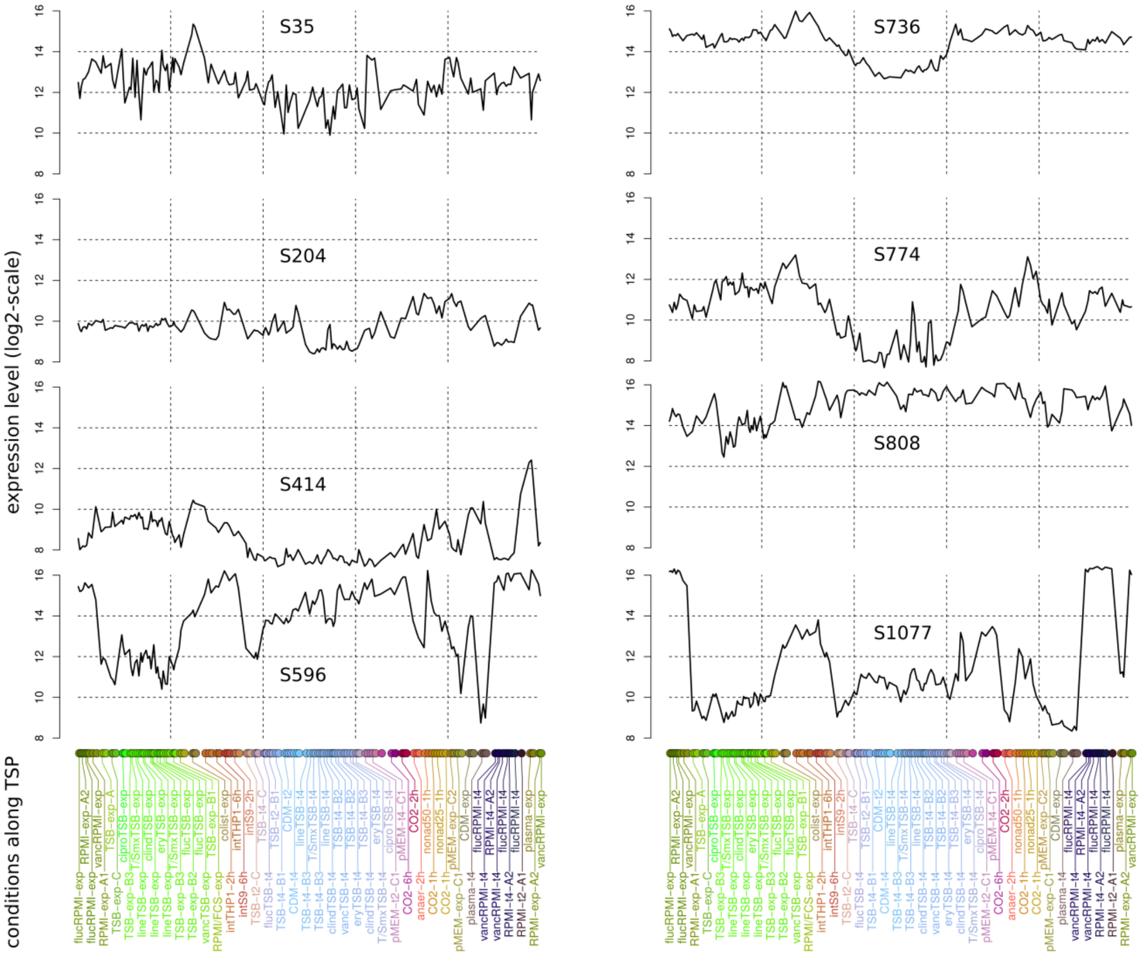

Figure D. Expression patterns of 8 newly identified potential sRNAs. Of note, S596 is antisense of an annotated CDS (SAOUHSC_1422) but was considered an sRNA rather than a typical antisense RNA (for details see main text).

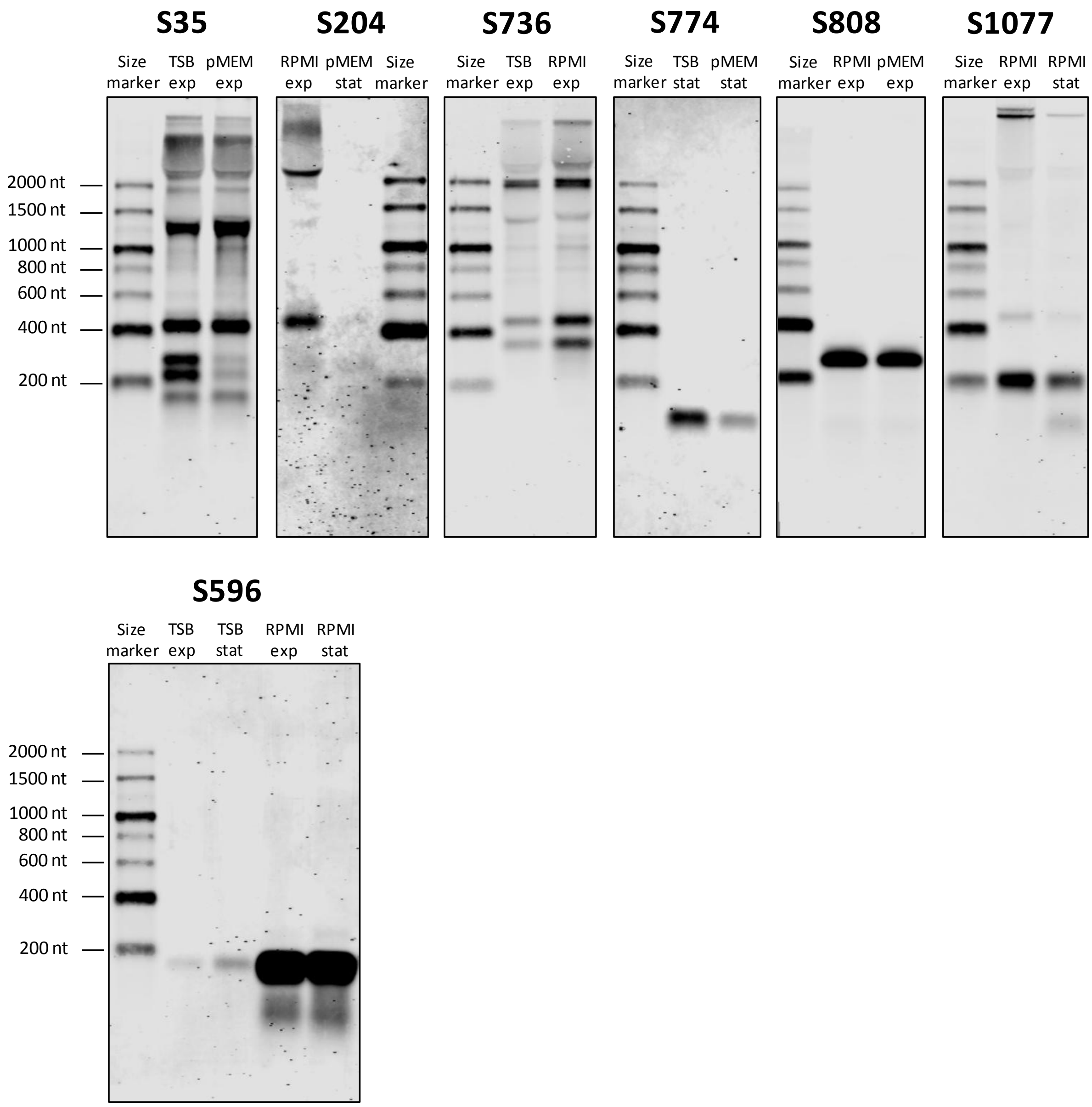

Figure E. Northern blot analysis of newly identified potential sRNAs. RNA samples were derived from exponentially growing and stationary phase cells in three different cultivation media (TSB, RPMI, pMEM). For each sRNA candidate two conditions were selected based on their expression profiles (see Figure D) or the induction pattern of the corresponding up-shift. For S596, which was identified as a probable Fur target, both RPMI and TSB samples were analyzed. Of note, based on our present results, RPMI is representative for iron-limited growth conditions. Northern blot analysis confirmed the presence of RNA molecules matching the respective sizes of the RNA segments identified by the tiling array approach (315 nt for S35, 482 nt for S204, 434 nt for S736, 94 nt for S774, 254 nt S808, 213 nt for S1077, and 176 nt S596). For S35, S736 and S1077 additional bands were detected which can be interpreted in light of their chromosomal contexts accessible via the *S. aureus* Expression Data Browser.

- The situation for S35 is complex, because this segment, except for its 3'-end, lies within a largely repeated region (~900 nt long). Since signals in repeated regions were discarded, tiling array analysis provided no direct information on the 5'-end of S35. In addition to the

transcript of ~300 nt, three bands were visible (~180 nt, ~220 nt, and 400 nt), which are compatible with transcripts generated from the S35 region, but could in principle also arise from the homologous loci. The larger transcripts (>1 kb) can be explained by the homology of S35 with these other regions in the genome (in particular downstream of *trkA* and within S347) where larger transcription units were identified by the tiling array analysis.

- In the case of S736 a larger transcript of ~1.9 Kb was detected. It corresponds most probably to read-through at the end of S736 where a transcriptional downshift site and an intrinsic terminator sequence were identified. It cannot be completely ruled out that S736 represents a regulatory 5'-UTR although no such element was predicted in front of SAOUHSC_01866 according to the Rfam and RegPrecise databases. In such a case the S736 segment would represent a prematurely terminated leader transcript which could still have an independent sRNA function.
- Finally, a longer transcript was detected with the S1077-specific probe, in particular under conditions of high S1077 expression (RPMI, exponential phase), indicating some read-through into the downstream operon or incomplete processing if the S1077 segment is generated by endonucleolytic cleavage from a longer transcript.

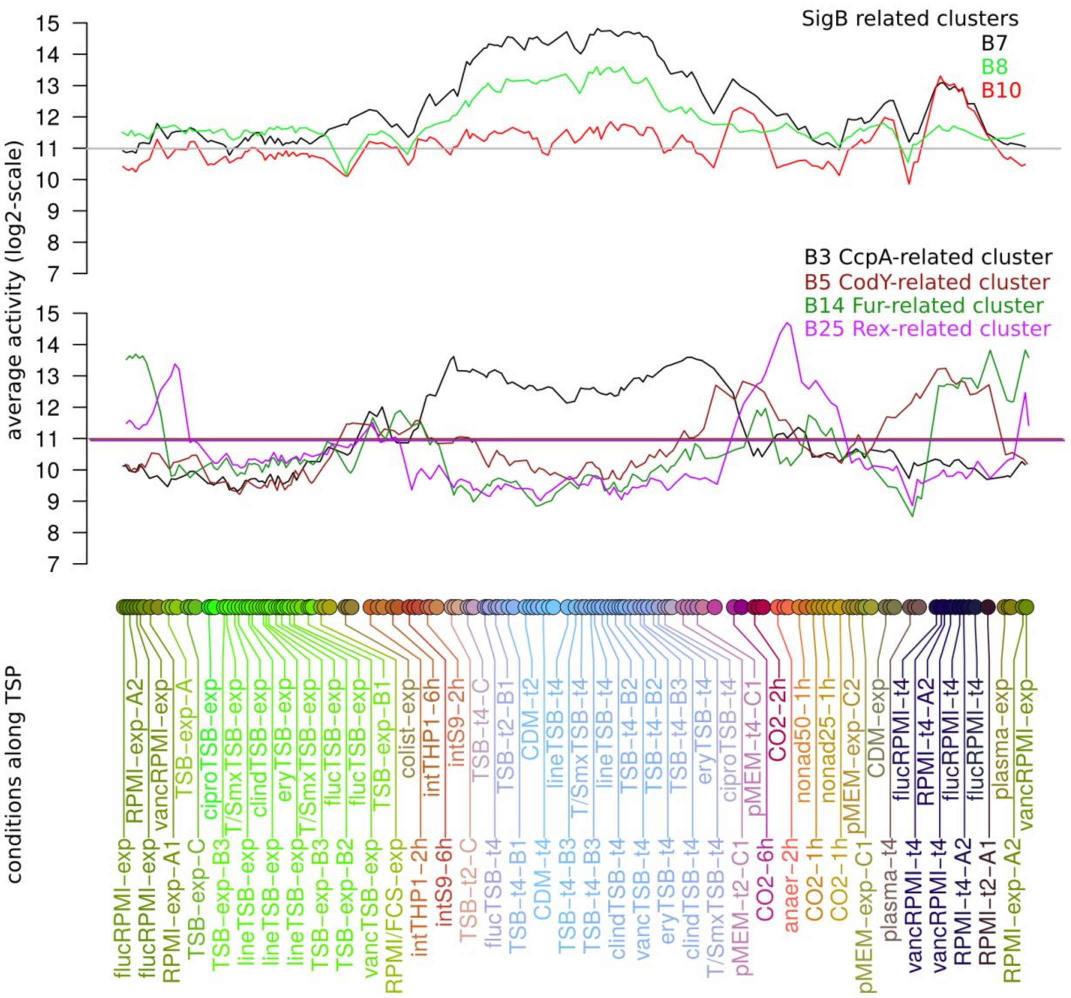

Figure F. Activity profiles of promoter clusters associated with SigB (B7, 8, and 10) and main transcription factor regulons (CcpA, CodY, Fur, and Rex). Cluster codes (B1-B25) are arbitrary numbers and correspond to those indicated in Fig 3 of the main text. Average activity of a promoter cluster is computed as the average reconstructed log2-transformed transcription signal after the corresponding up-shifts. Reconstruction of the transcriptional landscape along the chromosome was performed with our model of signal shift and drift (Nicolas *et al.*, 2009) and the whole landscapes were quantile-normalized to minimize the impact of artifacts.

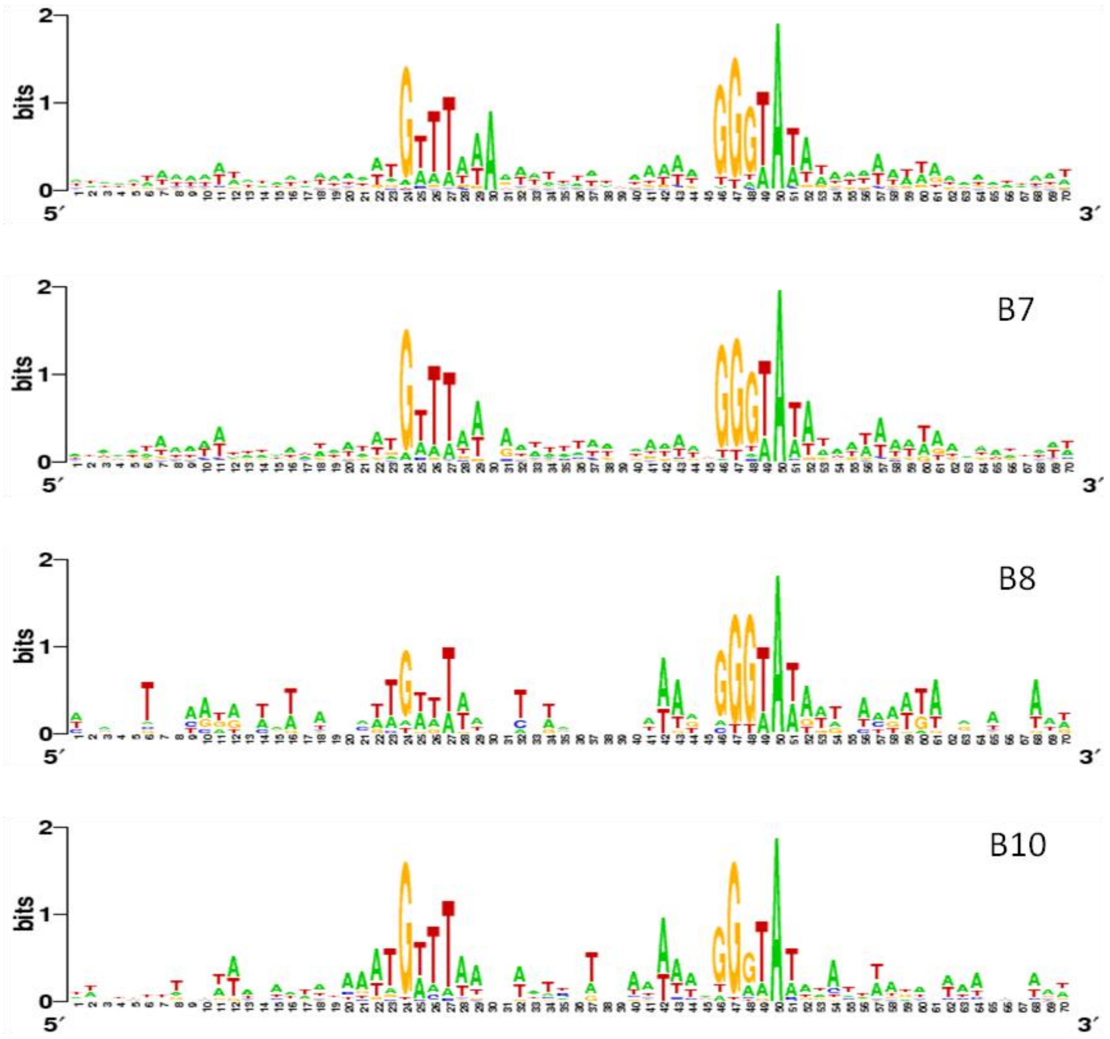

Figure G. Consensus motifs of all SigB-promoters (upper panel) and of promoter clusters B7, B8, and B10. Cluster codes (B1-B25) are arbitrary numbers and correspond to those indicated in Fig 3 of the main text. The frequency of nucleotides within the SigB promoters was visualized as Sequencelogo with the Weblogo tool (http://weblogo.berkeley.edu/logo.cgi) using default parameters.

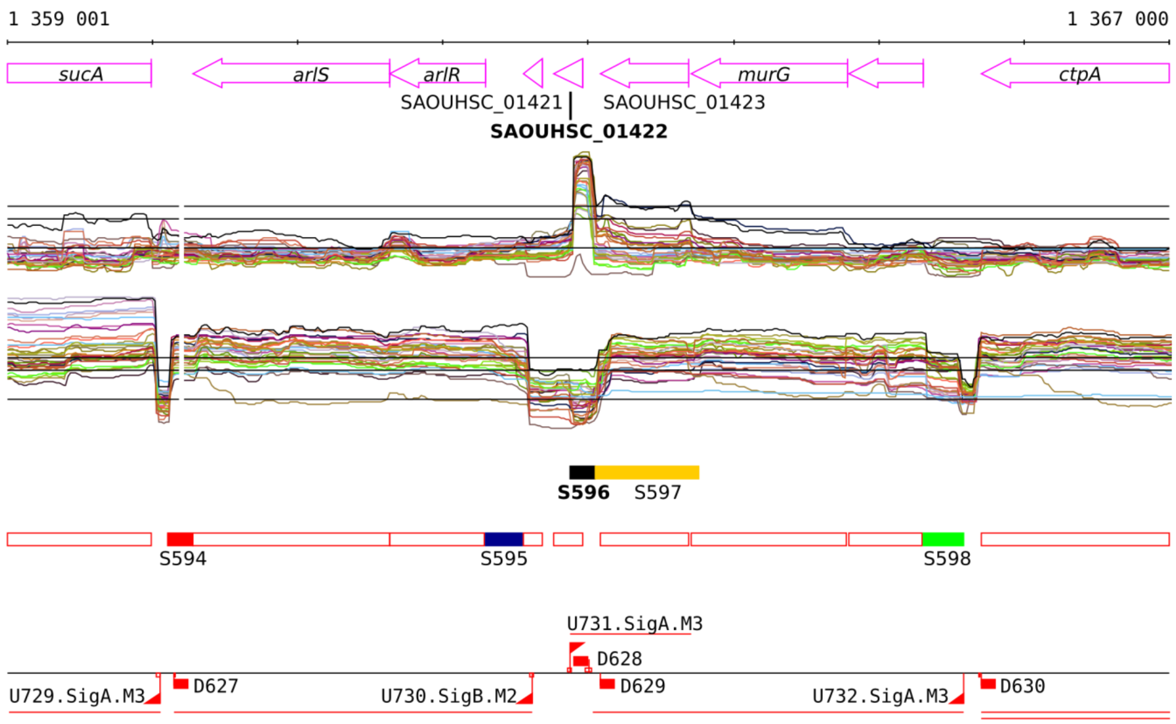

Figure H. Region of the *S. aureus* chromosome covering sRNA S596. From top-to-bottom: (i) genomic coordinates and CDS annotation from Genbank CP000253.1; (ii) a selection of 30 representative expression profiles (horizontal black lines show for each strand the chromosome median, and the associated 5-fold and 10-fold cut-offs) colored according to the position of the hybridization in 3D PCA, (iii) the detected up-shifts, the associated transcription units, and the down-shift positions, (iv) the new annotation with unannotated expressed segments colored according to the classification based on the transcriptional context. S596 overlap the antisense strand of a short annotated CDS (SAOUHSC_01422) which is not transcribed at significant level across the conditions tested (and may correspond to an erroneous annotation, see main text).

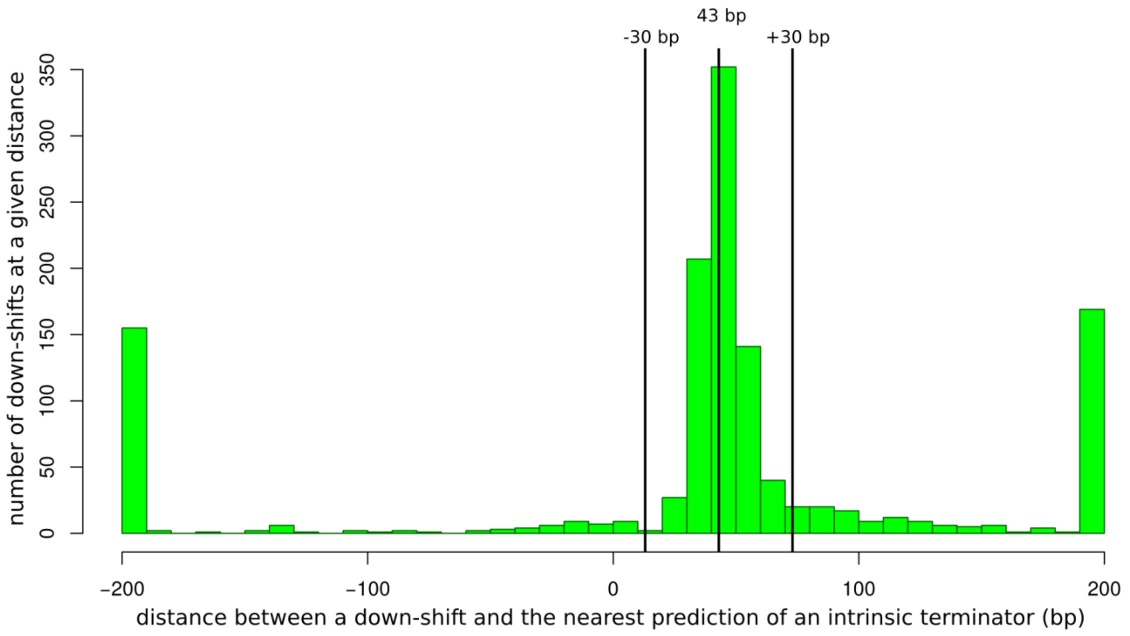

Figure I. Distance between transcription down-shifts and predicted intrinsic transcription terminators. Histogram of the distance distribution with bin size 10 bp and sample size 1261, corresponding to the total number of high-confidence down-shifts. Intrinsic transcription terminators were predicted from the sequence alone with TranstermHP (Kingsford *et al.*, 2007). The middle point of each predicted intrinsic transcription terminator served as a reference position for this comparison. Distance is positive when the reference point of the predicted intrinsic terminator is downstream the downshift (negative when upstream). Distances were truncated to 200bp for this representation. The vertical lines represent the (-30 bp,+30 bp) distance window of the histogram in which we considered a down-shift as associated to a TranstermHP prediction in the main text and in S7 Table. Of note this window is not centered on 0 but +43bp. This small shift reflects the fact that our downshift coordinates correspond to the 5'-ends of the probes whose lengths range between 45 bp and 65 bp. TranstermHP predictions were downloaded from http://transterm.cbcb.umd.edu/tt/Staphylococcus_aureus_NCTC_8325.tt.

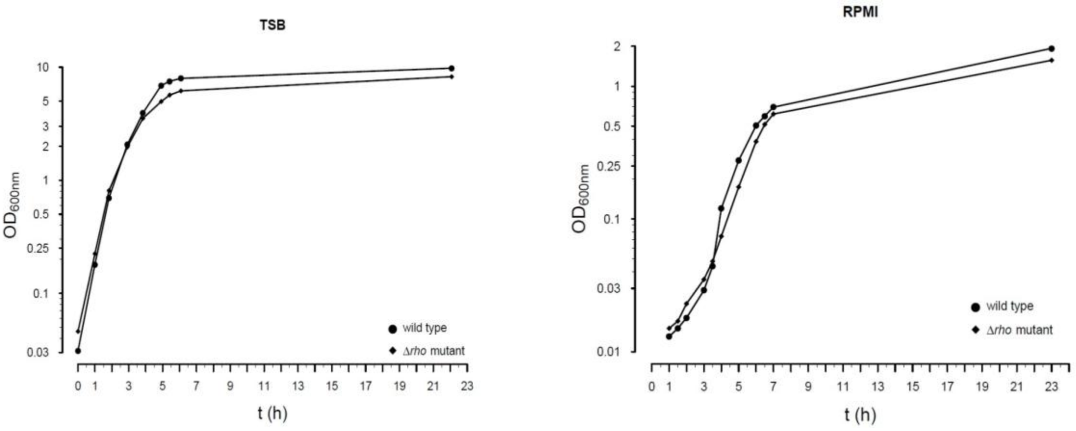

Figure J. Growth of *S. aureus* HG001 and its isogenic Δ*rho* mutant in TSB and RPMI medium.

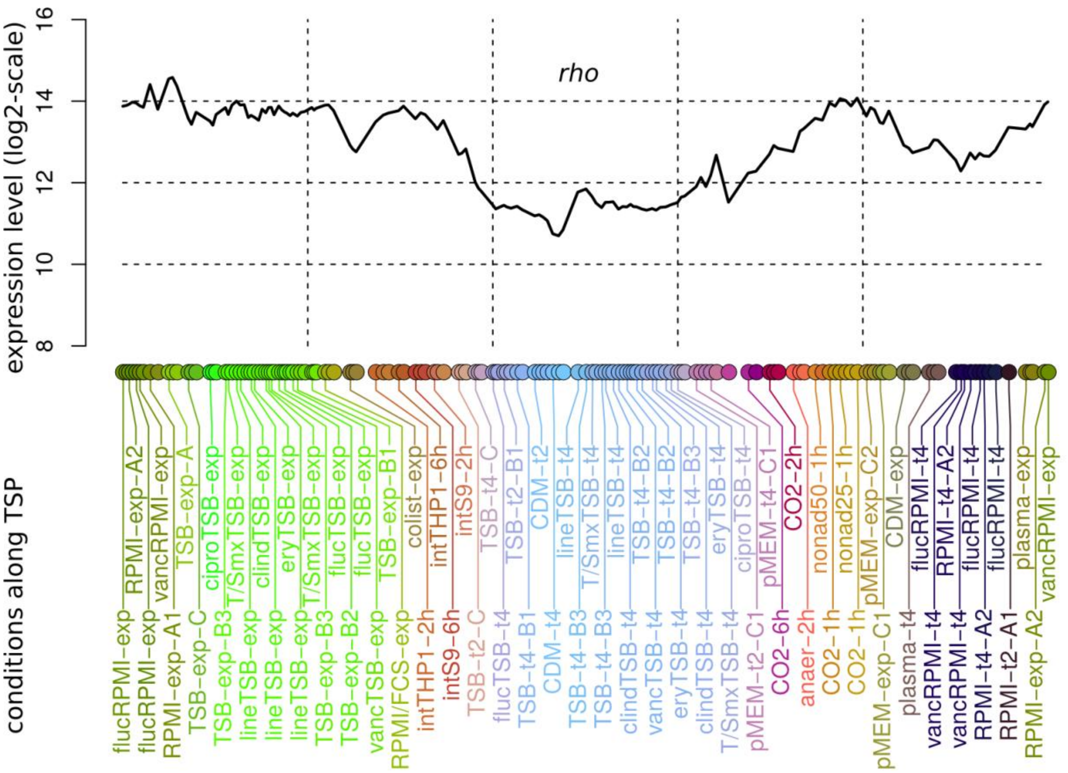

Figure K. Expression levels of *rho* under the different biological conditions.

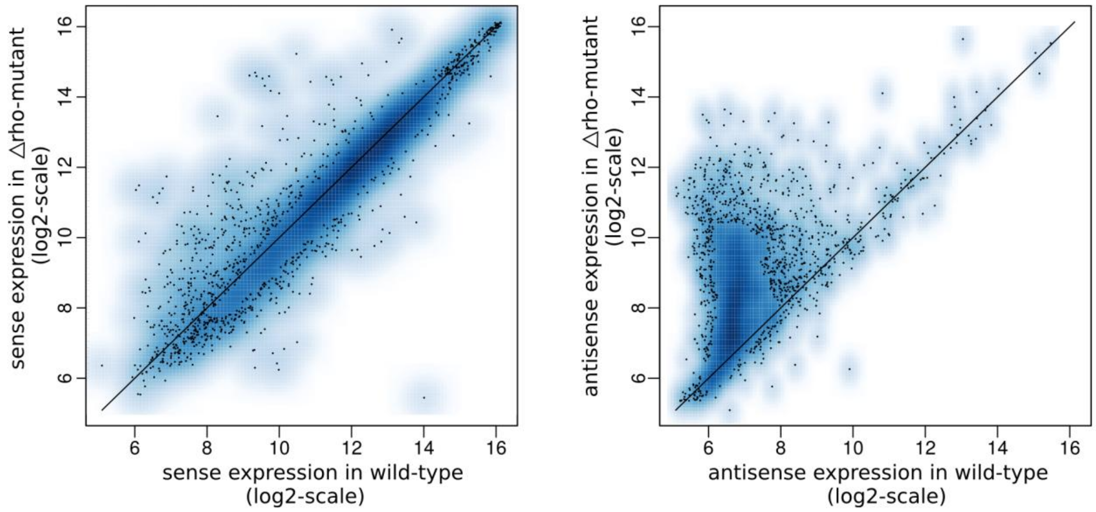

Figure L. Comparison of the impact of *rho*-deletion on the expression levels of the sense and antisense strands. Left: panel: scatter plot showing the impact of *rho*-deletion on the sense strand. Right panel: scatter plot showing the impact of *rho*-deletion on the antisense strand. The experimental condition considered here is exponential growth in RPMI medium, which is the condition - out of the four tested - showing the strongest effect of the *rho*-deletion (see main text, Table 2). Each point corresponds to the aggregated expression level of a particular gene annotated in Genbank, averaged between the two biological replicates. The density of points is represented by a blue gradient. The density was estimated with a 2D kernel smoother (R function “smoothScatter”, library “graphics”). The points in regions of highest intensity (dark blue) are not shown for clarity. Up-regulation is not detected on the sense strand, even for genes with an expression signal in the wild-type as low as of antisense regions.

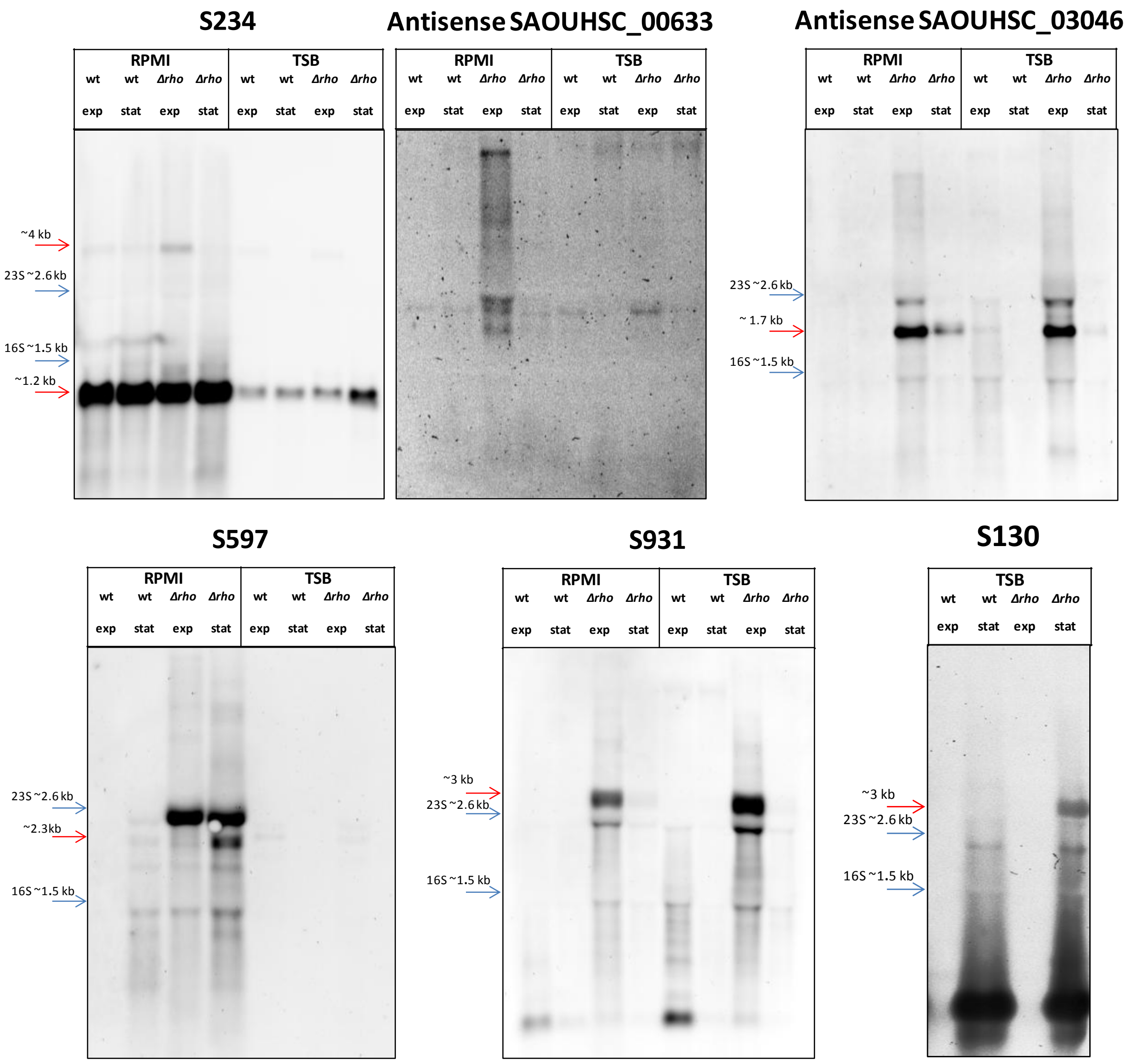

Figure M. Northern blot analysis of five antisense-generating transcribed regions. In the case of S234, the tiling array profile suggested a long (~9 Kb) 3'-extension of this potential regulatory RNA generated in the Δ*rho* mutant during exponential growth in RPMI medium. However, with the S234 probe a long transcript specific to the Δ*rho* mutant was not detected. Instead, a 4 Kb band was visible in all RPMI samples (i.e. from mutant and parental strains) which most likely corresponds to a transcript covering the upstream *mntA* operon and S234. The longer antisense transcript could only be detected with a probe complementary to the antisense strand of SAOUHSC_00633 revealing bands with different sizes larger than 3 Kb (RPMI, exponential growth). Thus, the antisense transcript downstream of S234 was obviously not associated with the S234 containing transcripts, but rather originated from a transcription start site immediately downstream of S234. Next, an antisense transcript initiating downstream of *cspB* was analyzed using a probe with specificity for the antisense strand of SAOUHSC_03046. This RNA, generated at high levels during exponential growth of the Δ*rho* mutant in both media, overlaps a tricistronic operon on the opposite strand (SAOUHSC_03048 to SAOUHSC_03046). Northern blot analysis revealed the presence of a 1.7 Kb long transcript in the Δ*rho* mutant which was particularly abundant in the exponential phase samples. In addition, two TUs extended over a long region in the absence of Rho

were analyzed, each containing a short RNA gene (S596 and S929, respectively) and a 3'PT segment resulting from inefficient termination in the wild type. Probes were directed against the 3'PT segments S597 and S931. In the case of the S596 downstream region, tiling array data of the Δ*rho* mutant showed high antisense transcript levels over a region of more than 11 Kb during exponential and stationary growth phase in RPMI medium. However, in the Northern blot analysis a relatively small specific band with a size of about 2.3 Kb was detected which was most abundant during stationary phase. In agreement with the array data showing highest expression of S597 during RPMI stationary phase, this band was also visible in the wild type stationary phase sample. Moreover, larger transcripts were detected exclusively in the Δ*rho* mutant including a strong main band most likely caused by an accumulation of degradation products, i.e. consisting of RNA fragments in the size range of the 23S rRNA whose electrophoretic mobility is disturbed due to co-migration with the highly abundant rRNA molecules. Northern blot signals obtained with a S931 specific probe revealed a 3 Kb antisense transcript in the Δ*rho* mutant generated during exponential growth in both media. Finally, the S130-S127 region containing an Indep segment (S130) and a 3'PT segment was analyzed which was extended in the Δ*rho* mutant during stationary phase in TSB. With the S130 specific probe a 3 Kb band was visible in the mutant sample corresponding to the transcript detected by the tiling array analysis (elongated up to the termination site downstream of SAOUHSC_00409).

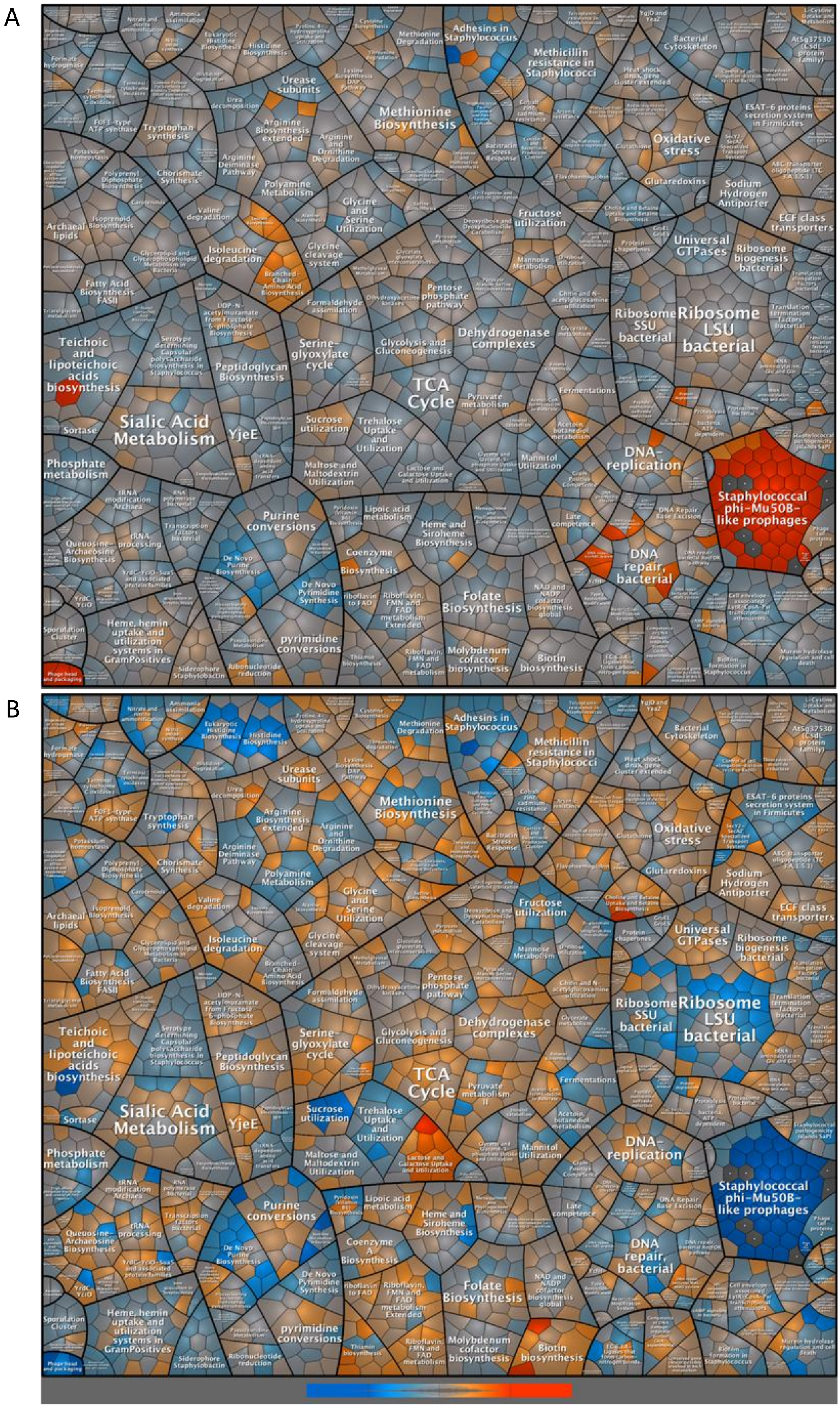

Figure N. Voronoi-treemap representation of transcriptome changes by sub-inhibitory concentrations of ciprofloxacin and flucloxacillin. Classification of genes is based on the

SEED functional categories as described in Pförtner *et al.* (2014). The treemaps display ratios of expression levels of *S. aureus* HG001 grown in TSB medium in the presence of **(A)** ciprofloxacin (exponential growth) or **(B)** flucloxacillin (stationary phase) versus the corresponding untreated conditions. Red color indicates at least a 4 times higher expression in the presence of antibiotics and blue color indicates at least 4 times lower expression in the presence of antibiotics. In addition to the effects of ciprofloxacin and flucloxacillin on the expression of phage-encoded genes (see main text), the transcription of several metabolic operons was significantly increased during stationary phase in the presence of flucloxacillin, in particular of operons involved in the biosynthesis of biotin and thiamin, metabolism of lactose and galactose, glycine betaine, and fatty acids.

Supplementary Tables

Table A. Oligonucleotides used for preparation of Northern blot probes.

Probe	Oligonucleotide	Sequence [a]
SAOUHSC_00975	SA_00975_for	ATGAAATTAGGTATGCTTTT
	SA_00975_revT7	CTAATACGACTCACTATAGGGAGATTAATAAAATTGTAGCCATT
AS_SAOUHSC_00974	SA_00974AS_for	TTAGCTATTGAAAAGATTTA
	SA_00974AS_revT7	CTAATACGACTCACTATAGGGAGAGATGTAGAAAATATTTTCAC
S234	SA_S234_for	CATCTCTTTTAACCACAGAG
	SA_S234_revT7	CTAATACGACTCACTATAGGGAGAATGGGGGGGAGGAAACCATA
AS_SAOUHSC_00633	SA_00633AS_for	CCATTTCATCATAATTGATGGC
	SA_00633AS_revT7	CTAATACGACTCACTATAGGGAGATATCATGATAAATTAGCATT
S130	SA_S130_for	GATAACTATGTACAATGAAG
	SA_S130_revT7	CTAATACGACTCACTATAGGGAGACTTAAATTATTTTGCGAAAA
S597	SA_S597_for	CTAAGTAATAACGTTGATAAAATAATG
	SA_S597_revT7	CTAATACGACTCACTATAGGGAGACGTATCTCTTTGCTCATCGT
S931	SA_S931_for	AAGAGATTAGCCACCATCTATCCA
	SA_S931_revT7	CTAATACGACTCACTATAGGGAGAATGATCGTGTATCAACGAAA
AS_SAOUHSC_03046	SA_03046AS_for	ATAGAAAGCGCATTTGTCATCGTAT
	SA_03046AS_revT7	CTAATACGACTCACTATAGGGAGAGTATGTCAAAACATTTAGTC
S596	SA_S596_for	TCACTAATGTATAATAGTAGTTG
	SA_S596_revT7	GAAATTAATACGACTCACTATAGGGAGAAGTGTCGTAAGGGTTTACTG
S35	SA_S35_for	CAATGGTAGAACCTTTTCTGGG
	SA_S35_revT7	GAAATTAATACGACTCACTATAGGGAGATATGAAAAGAATAGGCTGGGA
S204	SA_S204_for	CCTTTGCTACTCTATGGTTA
	SA_S204_revT7	GAAATTAATACGACTCACTATAGGGAGAGGTTCATCTATAAATCAACA
S736	SA_S736_for	ATAATTTAAAACGAAGCTTAAAAG
	SA_S736_revT7	GAAATTAATACGACTCACTATAGGGAGATCAAGAGCCATATATTCTAAT
S774	SA_S774_for	TAATATTATAATATAATCAATCGG
	SA_S774_revT7	GAAATTAATACGACTCACTATAGGGAGATAGTCGAATTTATTAAAAATCG
S808	SA_S808_for	AATGTGAAATGGTCATTCTTG
	SA_S808_revT7	GAAATTAATACGACTCACTATAGGGAGACATCAGTTAAGATGCGGTTATC
S1077	SA_S1077_for	ATATAGTATATAATCAAATCG
	SA_S1077_revT7	GAAATTAATACGACTCACTATAGGGAGACCGCAAAGTGGAAAGCATGAC

[a] The sequence $[N]_x$TAATACGACTCACTATAGGGAGA corresponds to the T7 promoter.

Table B. Comparison of potential *trans*-encoded small regulatory RNAs (sRNAs) with sRNAs identified by previous studies.

Segment Name[a]	RNA Name[b]	Strand	Start	End	Size (nt)	Category	Sigma Factor	Beaume[c]	Abu-Qatouseh[d]
**S35**		-1	115209	115524	315	indep	ND		
S58	RsaG	1	201740	201913	173	indep	ND	Teg93	
S109		-1	361935	362007	72	s-indep	SigA		Sau-5971
S142	4.5S RNA	1	444078	444397	319	indep	SigA	Teg42	
S201	RsaOI	-1	565660	565922	262	indep	SigA	Teg47	
**S204**		1	569584	570066	482	indep-NT	SigA		
S210	RsaA	1	575825	575970	145	s-indep	SigB	Teg88	Sau-64
S211		1	575972	576116	144	s-indep	SigB	Teg88	Sau-64
S234	RsaC	-1	623357	624469	1112	indep	ND	Teg90	
S317	RsaH	1	774283	774396	113	s-indep	SigA	Teg94	Sau-6059
S329	tmRNA	1	788284	788675	391	indep	SigA	Teg150	
S389	RsaE	1	911368	911465	97	s-indep	SigA	Teg92	Sau-20
**S414**		-1	990586	990684	98	s-indep-NT	ND		
S685	6S RNA	-1	1639007	1639262	255	indep	SigA	Teg97	
**S736**		-1	1773372	1773806	434	indep	SigA		
S771	SprC	-1	1861466	1861610	144	s-indep	SigA	Teg10	
**S774**		-1	1863829	1863923	94	s-indep	SigA		
**S808**		-1	1923624	1923878	254	indep	SigA		
S853	SprD	-1	2033653	2033770	117	5'UTR	SigA	Teg14	
S929		1	2211928	2212084	156	indep	SigA	Teg19a	
S999	RsaOG	-1	2377331	2377476	145	s-indep	SigA	Teg24	
S1036		-1	2446929	2448180	1251	indep	SigA	Teg27	
**S1077**		-1	2547824	2548037	213	indep	SigA		

[a] The sRNAs newly identified by this study are indicated in bold. RNA segment S853 corresponds to the SprD sRNA (Chabelskaya *et al.*, 2010). S853 was classified as 5'-UTR because no down-shift was detected at the 3'-end of the transcript. [b] The RNA name according to the first identification/ confirmation by Northern Blot or RT-qPCR (Pichon and Felden, 2005; Marchais *et al.*, 2009; Geissmann *et al.*, 2009; Bohn *et al.*, 2010) as given in Felden *et al.* (2011). [c] Name of the corresponding transcript of strain N315 identified by the RNA-seq based study of Beaume *et al.* (2010). [d] Name of the corresponding transcript identified by the analysis of nc-RNAs of a clinical isolate (Abu-Qatouseh *et al.*, 2010).

Table C. Known 5' *cis*-acting regions of *S. aureus*.

Regulated Gene [a]	Segment (this study)	Type [b]	Rfam [c]
serS	S4	T-box	RF00230
SAOUHSC_00341 (metI)	S106	T-box	RF00230
cysE	S175	T-box	RF00230
pheS	S450	T-box	RF00230
ileS	S476	T-box	RF00230
leuS	S740	T-box	RF00230
trpE	S576	T-box	RF00230
trpE	S577	T-box	RF00230
alaS	S678	T-box	RF00230
valS	S697	T-box	RF00230
valS	S698	T-box	RF00230
thrS	S709	T-box	RF00230
tyrS	S728	T-box	RF00230
glyS	S658	T-box	RF00230
hisS	S687	T-box	RF00230
rplJ	S183	L10_leader	RF00557
rplM	S962	L13_leader	RF00555
rplS	S500	L19_leader	RF00556
infC	S705	L20_leader	RF00558
rplU	S694	L21_leader	RF00559
SAOUHSC_00013 (metX)	S7	SAM	RF00162
SAOUHSC_00842	S349	SAM	RF00162
metK	S758	SAM	RF00162
SAOUHSC_02601	S1008	SAM	RF00162
SAOUHSC_01505	S626	FMN	RF00050
ribD	S743	FMN	RF00050
SAOUHSC_01022	S416	TPP	RF00059
SAOUHSC_02331 (tenA)	SAOUHSC_02332	TPP	RF00059
lysC	S584	Lysine	RF00168
lysP	S706	Lysine	RF00168
gcvT	S652	Glycine	RF00504
xpt	S120	Purine	RF00167
pyrR	S481	PyrR	RF00515
SAOUHSC_00721 (queC)	S279	PreQ1	RF00522
SAOUHSC_01440	S602	PreQ1	RF00522
glmS	S936	glmS	RF00234
SAOUHSC_00957	S399	yybP-ykoY	RF00080
cspC	S337	cspA	RF01766

[a] The information was derived from the Rfam database (Burge *et al.*, 2013) and RegPrecise (Novichkov *et al.*, 2013). [b] Type of 5' cis-acting structure from Rfam. [c] Rfam ID.

Table D. Results from previous transcriptome studies in comparison with the SigB regulon revealed by the promoter classification.

Locustag	Gene	Bischoff	Pané-Farré	Schult-hess
SAOUHSC_00037		yes		
SAOUHSC_00039	dus			
SAOUHSC_00057		yes	yes	yes
SAOUHSC_00058	norC	yes		
SAOUHSC_00065		yes		
SAOUHSC_00067	lctP1			
SAOUHSC_00114	capA	yes	yes	yes
SAOUHSC_00115	capB	yes	yes	yes
SAOUHSC_00116	capC	yes	yes	yes
SAOUHSC_00117	capD	yes	yes	yes
SAOUHSC_00118	capE	yes	yes	yes
SAOUHSC_00119	capF	yes	yes	
SAOUHSC_00120	capG	yes	yes	
SAOUHSC_00121	capH	yes	yes	yes
SAOUHSC_00122	capI	yes	yes	yes
SAOUHSC_00123	capJ	yes	yes	yes
SAOUHSC_00124	capK		yes	yes
SAOUHSC_00125	capL	yes	yes	
SAOUHSC_00126	capM	yes	yes	yes
SAOUHSC_00127	capN	yes	yes	
SAOUHSC_00128	capO	yes	yes	
SAOUHSC_00129	capP		yes	
SAOUHSC_00133		yes		
SAOUHSC_00134		yes		
SAOUHSC_00242		yes		
SAOUHSC_00285		yes	yes	yes
SAOUHSC_00287		yes	yes	yes
SAOUHSC_00309		yes	yes	yes
SAOUHSC_00318		yes		
SAOUHSC_00319		yes		
SAOUHSC_00320				
SAOUHSC_00337				
SAOUHSC_00338	metE			
SAOUHSC_00339	metH			
SAOUHSC_00340	metC			
SAOUHSC_00341	metI			
SAOUHSC_00356		yes	yes	yes
SAOUHSC_00358		yes	yes	
SAOUHSC_00369				
SAOUHSC_00370				
SAOUHSC_00371		yes		
SAOUHSC_00468	yabJ	yes		yes
SAOUHSC_00469	spoVG	yes		yes
SAOUHSC_00470				
SAOUHSC_00488	cysK			
SAOUHSC_00553	hxlA	yes	yes	yes
SAOUHSC_00554	hxlB	yes	yes	
SAOUHSC_00556	proP		yes	
SAOUHSC_00569		yes	yes	
SAOUHSC_00577	mvaK1	yes		
SAOUHSC_00578	mvaD	yes	yes	
SAOUHSC_00579	mvaK2	yes	yes	yes
SAOUHSC_00580				
SAOUHSC_00617				
SAOUHSC_00618				
SAOUHSC_00619		yes	yes	yes
SAOUHSC_00620	sarA	yes	yes	yes
SAOUHSC_00624		yes	yes	yes
SAOUHSC_00625	mnhA	yes	yes	yes
SAOUHSC_00626	mnhB	yes	yes	yes
SAOUHSC_00627	mnhC	yes	yes	yes
SAOUHSC_00628	mnhD	yes	yes	yes
SAOUHSC_00629	mnhE	yes		yes
SAOUHSC_00632	mnhG	yes	yes	yes
SAOUHSC_00686		yes		
SAOUHSC_00687		yes		
SAOUHSC_00688		yes		
SAOUHSC_00689		yes	yes	
SAOUHSC_00690		yes	yes	yes
SAOUHSC_00719	queE	yes		
SAOUHSC_00720	queD	yes		
SAOUHSC_00731				
SAOUHSC_00732	opuBB			
SAOUHSC_00736		yes	yes	yes
SAOUHSC_00788		yes		
SAOUHSC_00789	whiA	yes		
SAOUHSC_00790	clpP			
SAOUHSC_00792		yes		
SAOUHSC_00794	gapR			yes
SAOUHSC_00795	gapA			
SAOUHSC_00796	pgk			
SAOUHSC_00797	tpiA			
SAOUHSC_00798	pgm			
SAOUHSC_00810		yes		
SAOUHSC_00811		yes		
SAOUHSC_00812	clfA	yes	yes	yes
SAOUHSC_00820		yes		
SAOUHSC_00821		yes		yes
SAOUHSC_00823				
SAOUHSC_00824				
SAOUHSC_00826		yes	yes	
SAOUHSC_00831	ohrA	yes		yes
SAOUHSC_00840		yes		
SAOUHSC_00841		yes		
SAOUHSC_00842				
SAOUHSC_00843				
SAOUHSC_00844				
SAOUHSC_00845		yes	yes	yes
SAOUHSC_00847	sufC	yes		
SAOUHSC_00848	sufD			yes

Table D - continued

Locustag	Gene	Bischoff	Pané-Farré	Schult-hess
SAOUHSC_00849	sufS			
SAOUHSC_00850	sufU			
SAOUHSC_00851	sufB			
SAOUHSC_00854		yes		yes
SAOUHSC_00855		yes		yes
SAOUHSC_00977		yes		
SAOUHSC_00994	atl		yes	yes
SAOUHSC_01024				
SAOUHSC_01025				
SAOUHSC_01027		yes	yes	
SAOUHSC_01028	ptsH			
SAOUHSC_01029	ptsI			
SAOUHSC_01034	trkA			
SAOUHSC_01050				
SAOUHSC_01103	sdhC			
SAOUHSC_01104	sdhA			
SAOUHSC_01105	sdhB			
SAOUHSC_01106	murI			
SAOUHSC_01107				
SAOUHSC_01108				
SAOUHSC_01181		yes		
SAOUHSC_01222	topA			
SAOUHSC_01223	gid			
SAOUHSC_01248	truB	yes		yes
SAOUHSC_01249	ribC			
SAOUHSC_01250	rpsO			
SAOUHSC_01251	pnpA			
SAOUHSC_01254				
SAOUHSC_01377	opp-2F			
SAOUHSC_01419	arlS			
SAOUHSC_01420	arlR			yes
SAOUHSC_01528				
SAOUHSC_01625	efp			
SAOUHSC_01653	sodA			
SAOUHSC_01729		yes		yes
SAOUHSC_01730	csbD	yes		
SAOUHSC_01766	folC			
SAOUHSC_01784	rplT			
SAOUHSC_01785	rpmI			
SAOUHSC_01786	infC			
SAOUHSC_01787	lysP	yes		yes
SAOUHSC_01815				
SAOUHSC_01854		yes	yes	yes
SAOUHSC_01855		yes	yes	
SAOUHSC_01856	murC			
SAOUHSC_01857				
SAOUHSC_01858				
SAOUHSC_01869		yes	yes	
SAOUHSC_01890		yes		
SAOUHSC_01945	epiG	yes	yes	yes
SAOUHSC_01947	epiE	yes		yes
SAOUHSC_01948	epiF	yes		yes

Locustag	Gene	Bischoff	Pané-Farré	Schult-hess
SAOUHSC_01969		yes		
SAOUHSC_01971				
SAOUHSC_01984				
SAOUHSC_01986		yes		
SAOUHSC_01987		yes	yes	
SAOUHSC_02012	sgtB			
SAOUHSC_02013		yes	yes	yes
SAOUHSC_02095	ptpA	yes		yes
SAOUHSC_02096		yes		
SAOUHSC_02097		yes	yes	yes
SAOUHSC_02137	sdcS			
SAOUHSC_02138				
SAOUHSC_02145		yes		
SAOUHSC_02244		yes		
SAOUHSC_02248		yes		
SAOUHSC_02249		yes		
SAOUHSC_02298	sigB	yes	yes	yes
SAOUHSC_02299	rsbW	yes	yes	yes
SAOUHSC_02300	rsbV	yes	yes	yes
SAOUHSC_02320		yes		
SAOUHSC_02336	fabZ	yes	yes	
SAOUHSC_02337	murA1	yes	yes	yes
SAOUHSC_02338		yes		yes
SAOUHSC_02363		yes	yes	yes
SAOUHSC_02381	dps			
SAOUHSC_02382		yes	yes	
SAOUHSC_02384				
SAOUHSC_02385	manA			
SAOUHSC_02386				
SAOUHSC_02387		yes	yes	yes
SAOUHSC_02401		yes	yes	yes
SAOUHSC_02402	mtlA	yes	yes	yes
SAOUHSC_02403	mtlD	yes		yes
SAOUHSC_02418	mdeA			
SAOUHSC_02433		yes		yes
SAOUHSC_02434		yes		
SAOUHSC_02441	asp23	yes	yes	yes
SAOUHSC_02442	amaP	yes	yes	yes
SAOUHSC_02443		yes	yes	yes
SAOUHSC_02444	opuD2	yes	yes	yes
SAOUHSC_02460				
SAOUHSC_02466		yes	yes	yes
SAOUHSC_02547	modB			
SAOUHSC_02549	modA			
SAOUHSC_02555				
SAOUHSC_02556				yes
SAOUHSC_02569	sarY			yes
SAOUHSC_02570				yes
SAOUHSC_02572		yes	yes	
SAOUHSC_02573	nhaC		yes	
SAOUHSC_02574			yes	yes
SAOUHSC_02581		yes	yes	

Table D - continued

Locustag	Gene	Bischoff	Pané-Farré	Schult-hess
SAOUHSC_02582	fdhA	yes	yes	
SAOUHSC_02583				
SAOUHSC_02584		yes		
SAOUHSC_02604		yes	yes	yes
SAOUHSC_02610	hutG	yes	yes	yes
SAOUHSC_02635	tcaA			
SAOUHSC_02636	tcaR			
SAOUHSC_02637				
SAOUHSC_02650		yes		
SAOUHSC_02665		yes	yes	yes
SAOUHSC_02703	gpmA			
SAOUHSC_02722		yes		
SAOUHSC_02751	pnbA	yes	yes	
SAOUHSC_02753		yes	yes	yes
SAOUHSC_02754		yes	yes	yes
SAOUHSC_02771		yes		
SAOUHSC_02772		yes		
SAOUHSC_02774		yes	yes	yes
SAOUHSC_02779		yes		
SAOUHSC_02780				
SAOUHSC_02812		yes		yes
SAOUHSC_02849	cidC	yes	yes	yes
SAOUHSC_02862	clpL	yes	yes	yes
SAOUHSC_02877	crtN	yes	yes	yes

Locustag	Gene	Bischoff	Pané-Farré	Schult-hess
SAOUHSC_02879	crtM	yes	yes	yes
SAOUHSC_02880	crtQ	yes	yes	yes
SAOUHSC_02881		yes	yes	yes
SAOUHSC_02882	crtO	yes	yes	yes
SAOUHSC_02899		yes	yes	yes
SAOUHSC_02900		yes	yes	yes
SAOUHSC_02908		yes	yes	
SAOUHSC_02925		yes		
SAOUHSC_02926	fdaB			
SAOUHSC_02929				
SAOUHSC_02930		yes	yes	
SAOUHSC_02936		yes		yes
SAOUHSC_02980				
SAOUHSC_02982				
SAOUHSC_02983		yes	yes	
SAOUHSC_02984		yes	yes	yes
SAOUHSC_02991				
SAOUHSC_02994		yes	yes	yes
SAOUHSC_02995		yes		
SAOUHSC_03022		yes		
SAOUHSC_03028		yes		
SAOUHSC_03032		yes		yes
SAOUHSC_03033	nixA	yes		yes
SAOUHSC_03035		yes		

By a promoter classification approach (see main text), 249 protein-coding genes were identified whose expression is controlled by the alternative sigma factor SigB. Of these, 163 had previously been considered as SigB-regulated by at least one of the following studies: Bischoff *et al.*, 2004; Pané-Farré *et al.*, 2006; Schulthess *et al.*, 2011.

Table E. Target prediction for the potential sRNA S596

rank [a]	fdr	p-value	Locustag	Name	Annotation
1	2.57E-06	1.11E-09	SAOUHSC_01420	arlR	DNA-binding response regulator
2	0.070565	0.000214	SAOUHSC_01103	sdhC	succinate dehydrogenase cytochrome b558 subunit
3	0.070565	0.000175	SAOUHSC_01491	gpsA	NAD(P)H-dep. glycerol-3-phosphate dehydrogenase
4	0.070565	0.000214	SAOUHSC_02113	rumA	RNA methyltransferase
5	0.070565	0.000112	SAOUHSC_02582	fdhA	formate dehydrogenase subunit alpha
6	0.070565	0.000192	SAOUHSC_02651	NA	conserved hypothetical protein
7	0.087559	0.000341	SAOUHSC_02760	NA	putative glutamate synthase
8	0.093590	0.000405	SAOUHSC_01269	miaB	(dimethylallyl)adenosine tRNA methylthiotransferase
9	0.099267	0.000507	SAOUHSC_00907	NA	conserved hypothetical protein
10	0.099267	0.000558	SAOUHSC_02779	NA	conserved hypothetical protein
11	0.099267	0.000520	SAOUHSC_02943	citM	citrate transporter
12	0.122421	0.000847	SAOUHSC_01347	citB	aconitate hydratase
13	0.122421	0.000778	SAOUHSC_01802	citZ	citrate synthase
14	0.122421	0.000832	SAOUHSC_02675	nreC	conserved hypothetical protein
15	0.210293	0.001546	SAOUHSC_02303	NA	conserved hypothetical protein
16	0.251127	0.001955	SAOUHSC_01882	NA	conserved hypothetical protein
46	0.713032	0.016143	SAOUHSC_00904	addB	exonuclease
49	0.744890	0.019521	SAOUHSC_01327	katA	catalase
56	0.763364	0.020801	SAOUHSC_01065	ctaA	heme A synthase
75	0.826352	0.027986	SAOUHSC_01962	hemE	uroporphyrinogen decarboxylase
76	0.826352	0.031038	SAOUHSC_02281	ilvD	dihydroxy-acid dehydratase

[a] The rank is based on fdr values as revealed by target prediction using CopraRNA – v.2.0.1 (Wright *et al.*, 2013) with default parameters, i.e. the sRNA:mRNA interaction sites were searched in the (-200nt,+100nt) region around the 5'-ends of the annotated genes. The refseq accessions of the considered genome sequences are NC_007795, NC_004461, NC_022737 NC_014925,NC_007168, NC_017353, NC_012121, NC_007350 in which S596 homologs were identified with blastn. The predicted target genes listed here are those with an fdr <=0.25 (ranks 1-16) and in addition genes with functions related to the iron-sparing response mediated by known Fur-regulated sRNAs of other bacteria (see main text): genes encoding iron-sulfur-cluster containing proteins (*citB*, *fdhA*, *addB*, *ilvD*, *miaB*), heme biosynthesis/ containing enzymes (*ctaA*, *hemE*, *katA*) and TCA cycle enzymes (*citZ*, *sdhCA*).

REFERENCES

Arnaud M, Chastanet A, Débarbouillé M (2004) New vector for efficient allelic replacement in naturally nontransformable, low-GC-content, gram-positive bacteria. Appl Environ Microbiol 70: 6887-6891.

Abu-Qatouseh LF, Chinni SV, Seggewiss J, Proctor RA, Brosius J, et al. (2010) Identification of differentially expressed small non-protein-coding RNAs in Staphylococcus aureus displaying both the normal and the small-colony variant phenotype. J Mol Med 88: 565-575.

Beaume M, Hernandez D, Farinelli L, Deluen C, Linder P, et al. (2010) Cartography of methicillin-resistant S. aureus transcripts: detection, orientation and temporal expression during growth phase and stress conditions. PLoS One 5: e10725.

Bischoff M, Dunman P, Kormanec J, Macapagal D, Murphy E, et al. (2004) Microarray-based analysis of the Staphylococcus aureus σB regulon. J Bacteriol 186: 4085-4099.

Bohn C, Rigoulay C, Chabelskaya S, Sharma CM, Marchais A, et al. (2010) Experimental discovery of small RNAs in Staphylococcus aureus reveals a riboregulator of central metabolism. Nucleic Acids Res 38: 6620-6636.

Bolstad BM, Irizarry RA, Astrand M, Speed TP (2003) A comparison of normalization methods for high density oligonucleotide array data based on variance and bias. Bioinformatics 19: 185-193.

Burge SW, Daub J, Eberhardt R, Tate J, Barquist L, et al. (2013) Rfam 11.0: 10 years of RNA families. Nucleic Acids Res 41: D226-232.

Chabelskaya S, Gaillot O, Felden B (2010) A Staphylococcus aureus small RNA is required for bacterial virulence and regulates the expression of an immune-evasion molecule. PLoS Pathog 6: e1000927.

Felden B, Vandenesch F, Bouloc P, Romby P (2011) The Staphylococcus aureus RNome and its commitment to virulence. PLoS Pathog 7: e1002006.

Geissmann T, Chevalier C, Cros MJ, Boisset S, Fechter P, et al. (2009) A search for small noncoding RNAs in Staphylococcus aureus reveals a conserved sequence motif for regulation. Nucleic Acids Res 37: 7239-7257.

Helmann JD (2014) Specificity of metal sensing: iron and manganese homeostasis in Bacillus subtilis. J Biol Chem 289: 28112-28120.

Horton RM, Hunt HD, Ho SN, Pullen JK, Pease LR (1989) Engineering hybrid genes without the use of restriction enzymes: gene splicing by overlap extension. Gene 77: 61-68.

Kingsford CL, Ayanbule K, Salzberg SL (2007) Rapid, accurate, computational discovery of Rho-independent transcription terminators illuminates their relationship to DNA uptake. Genome Biol 8: R22.

Marchais A, Naville M, Bohn C, Bouloc P, Gautheret D (2009) Single-pass classification of all noncoding sequences in a bacterial genome using phylogenetic profiles. Genome Res 19: 1084-1092.

Miller M, Dreisbach A, Otto A, Becher D, Bernhardt J, et al. (2011) Mapping of interactions between human macrophages and Staphylococcus aureus reveals an involvement of MAP kinase signaling in the host defense. J Proteome Res 10: 4018-4032.

Nicolas P, Leduc A, Robin S, Rasmussen S, Jarmer H, Bessières P (2009) Transcriptional landscape estimation from tiling array data using a model of signal shift and drift. Bioinformatics 25: 2341-2347.

Nicolas P, Mäder U, Dervyn E, Rochat T, Leduc A, Pigeonneau N, et al. (2012) Condition-dependent transcriptome reveals high-level regulatory architecture in Bacillus subtilis. Science 335: 1103–1106.

Novichkov PS, Kazakov AE, Ravcheev DA, Leyn SA, Kovaleva GY, et al. (2013) RegPrecise 3.0 - A resource for genome-scale exploration of transcriptional regulation in bacteria. BMC Genomics 14: 745.

Pané-Farré J, Jonas B, Förstner K, Engelmann S, Hecker M (2006) The σB regulon in Staphylococcus aureus and its regulation. Int J Med Microbiol 296: 237-258.

Pförtner H, Burian MS, Michalik S, Depke M, Hildebrandt P, et al. (2014) Activation of the alternative sigma factor SigB of Staphylococcus aureus following internalization by epithelial cells - an in vivo proteomics perspective. Int J Med Microbiol 304: 177-187.

Pichon C, Felden B (2005) Small RNA genes expressed from Staphylococcus aureus genomic and pathogenicity islands with specific expression among pathogenic strains. Proc Natl Acad Sci U S A 102: 14249-14254.

Platero R, Peixoto L, O'Brian MR, Fabiano E (2004) Fur is involved in manganese-dependent regulation of mntA (sitA) expression in Sinorhizobium meliloti. Appl Environ Microbiol 70: 4349-4355.

Remy L, Carrière M, Derré-Bobillot A, Martini C, Sanguinetti M, Borezée-Durant E (2013) The Staphylococcus aureus Opp1 ABC transporter imports nickel and cobalt in zinc-depleted conditions and contributes to virulence. Mol Microbiol 87: 730-743.

Ritchie ME, Phipson B, Wu D, Hu Y, Law CW, et al. (2015) limma powers differential expression analyses for RNA-sequencing and microarray studies. Nucleic Acids Res 43: e47.

Roberts C, Anderson KL, Murphy E, Projan SJ, Mounts W, et al. (2006) Characterizing the effect of the Staphylococcus aureus virulence factor regulator, SarA, on log-phase mRNA half-lives. J Bacteriol 188: 2593-2603.

Schulthess B, Bloes DA, François P, Girard M, Schrenzel J, et al. (2011) The σB-dependent yabJ-spoVG operon is involved in the regulation of extracellular nuclease, lipase, and protease expression in Staphylococcus aureus. J Bacteriol 193: 4954-4962.

Sheldon JR, Heinrichs DE (2012) The iron-regulated staphylococcal lipoproteins. Front Cell Infect Microbiol 2: 41.

Skaar EP, Gaspar AH, Schneewind O (2004) IsdG and IsdI, heme-degrading enzymes in the cytoplasm of Staphylococcus aureus. J Biol Chem 279: 436-443.

Strimmer K (2008) A unified approach to false discovery rate estimation. BMC Bioinformatics 9: 303.

Wright PR, Richter AS, Papenfort K, Mann M, Vogel J, et al. (2013) Comparative genomics boosts target prediction for bacterial small RNAs. Proc Natl Acad Sci U S A 110: E3487-3496.
