# Supplementary material for: Staphylococcus aureus Transcriptome Architecture: From Laboratory to Infection-Mimicking Conditions
Source: PLoS Genet. 2016 Apr 1;12(4):e1005962. doi: 10.1371/journal.pgen.1005962 (PMC4818034; doi:10.1371/journal.pgen.1005962)
Supplement: S1 Fig — Gene expression levels (normalized log2 intensities) are displayed for the following conditions: exponential growth and stationary phase in different cultivation media and human plasma (blue bars); growth in the presence of sub-inhibitory concentrations of various antibiotics in TSB (red bars) and RPMI medium (orange bars); internalization of S. aureus by S9 bronchial epithelial cells or THP-1 macrophages and related conditions, i.e. 2.5 hours of anaerobic incubation in pMEM medium at 37°C, 1 hour of incubation in the infection medium at 37°C and 5% CO2 without agitation, and non-adherent bacteria retrieved from the supernatant of S9 cells after 1 hour of infection (green bars). (PDF) [file pgen.1005962.s002.pdf]

virulence associated genes

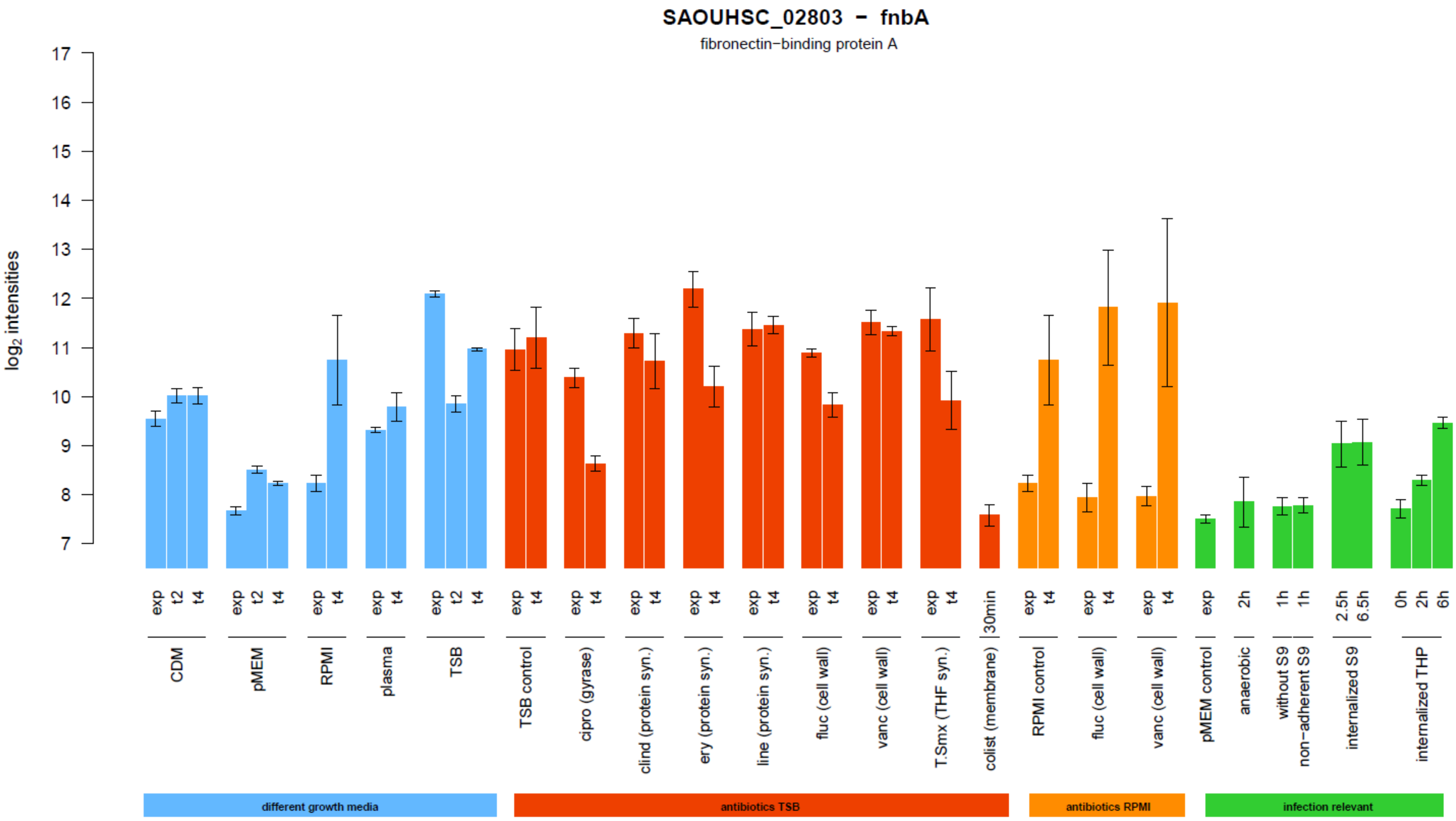
SAOUHSC_02803 – fnbA
fibronectin−binding protein A
log2 intensities
17
16
15
14
13
12
11
10
9
8
7
exp t2 t4
CDM
exp t2 t4
pMEM
exp t4
RPMI
exp t4
plasma
exp t2 t4
TSB
exp t4
TSB control
exp t4
cipro (gyrase)
exp t4
clind (protein syn.)
exp t4
ery (protein syn.)
exp t4
line (protein syn.)
exp t4
fluc (cell wall)
exp t4
vanc (cell wall)
exp t4
T.Smx (THF syn.)
30min
colist (membrane)
exp t4
RPMI control
exp t4
fluc (cell wall)
exp t4
vanc (cell wall)
exp
pMEM control
2h
anaerobic
1h
without S9
1h
non−adherent S9
2.5h 6.5h
internalized S9
0h 2h 6h
internalized THP
different growth media
antibiotics TSB
antibiotics RPMI
infection relevant

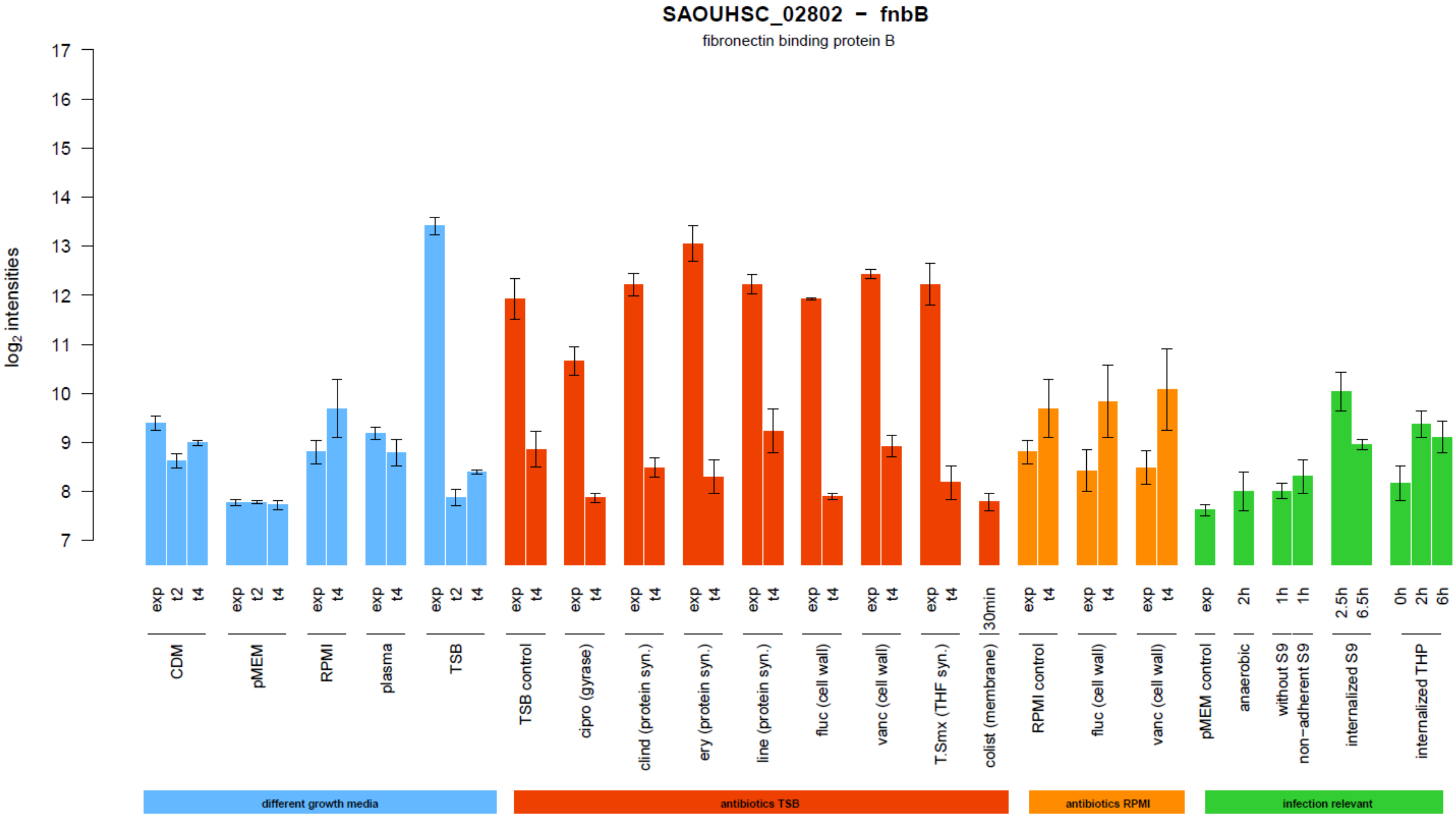

SAOUHSC_02802 – fnbB
fibronectin binding protein B
log2 intensities
17
16
15
14
13
12
11
10
9
8
7
exp
t2
t4
CDM
pMEM
RPMI
plasma
TSB
TSB control
cipro (gyrase)
clind (protein syn.)
ery (protein syn.)
line (protein syn.)
fluc (cell wall)
vanc (cell wall)
T.Smx (THF syn.)
30min
colist (membrane)
RPMI control
pMEM control
2h
anaerobic
1h
without S9
non−adherent S9
2.5h
6.5h
internalized S9
0h
6h
internalized THP
different growth media
antibiotics TSB
antibiotics RPMI
infection relevant

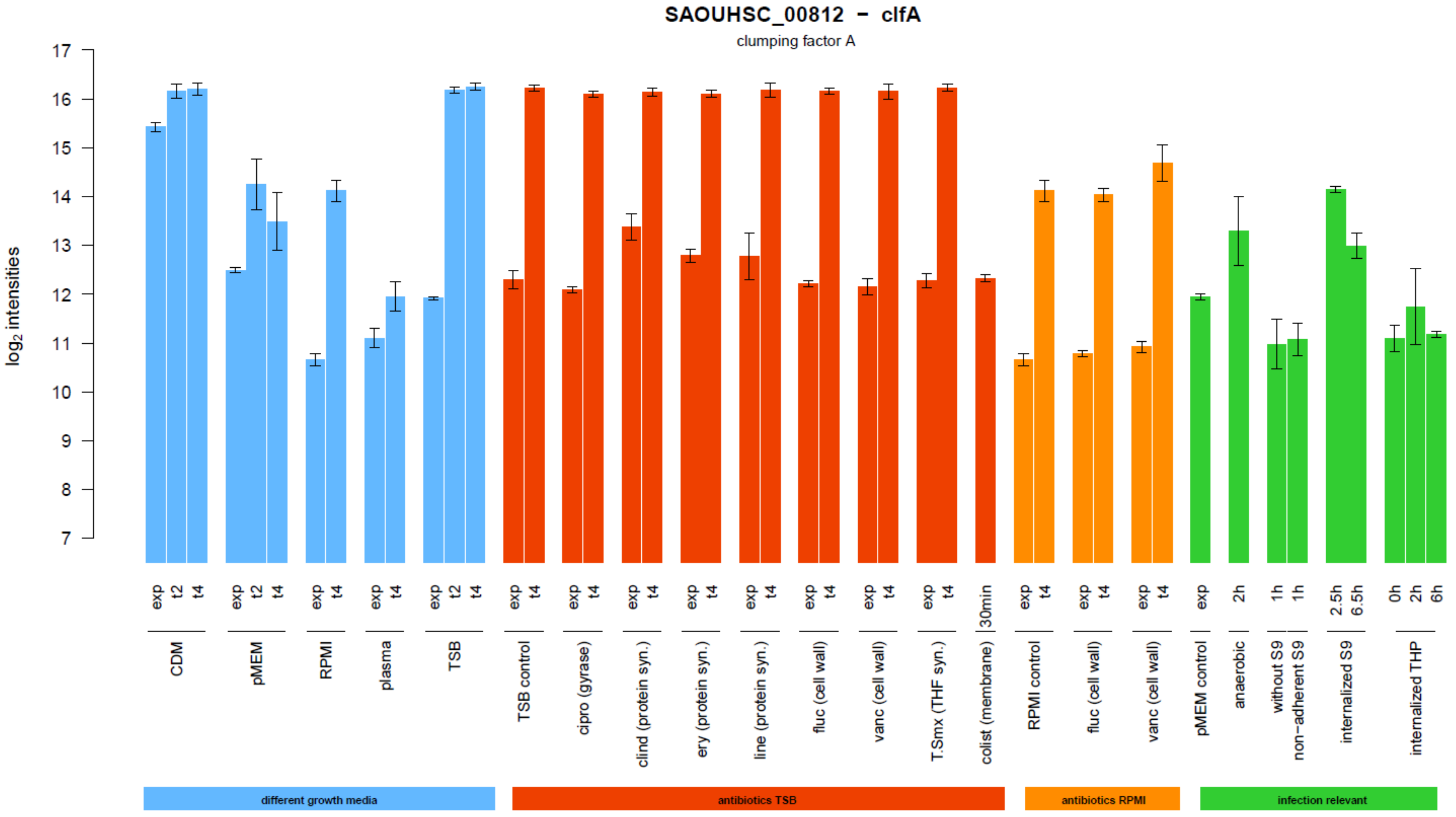
SAOUHSC_00812 - clfA
clumping factor A
log2 intensities
17
16
15
14
13
12
11
10
9
8
7
exp t2 t4
CDM
pMEM
RPMI
plasma
TSB
TSB control
cipro (gyrase)
clind (protein syn.)
ery (protein syn.)
linc (protein syn.)
fluc (cell wall)
vanc (cell wall)
T.Smx (THF syn.)
30min
colist (membrane)
RPMI control
fluc (cell wall)
vanc (cell wall)
pMEM control
anaerobic
without S9
non-adherent S9
internalized S9
internalized THP
2h
1h
2.5h
6.5h
0h
6h
different growth media
antibiotics TSB
antibiotics RPMI
infection relevant

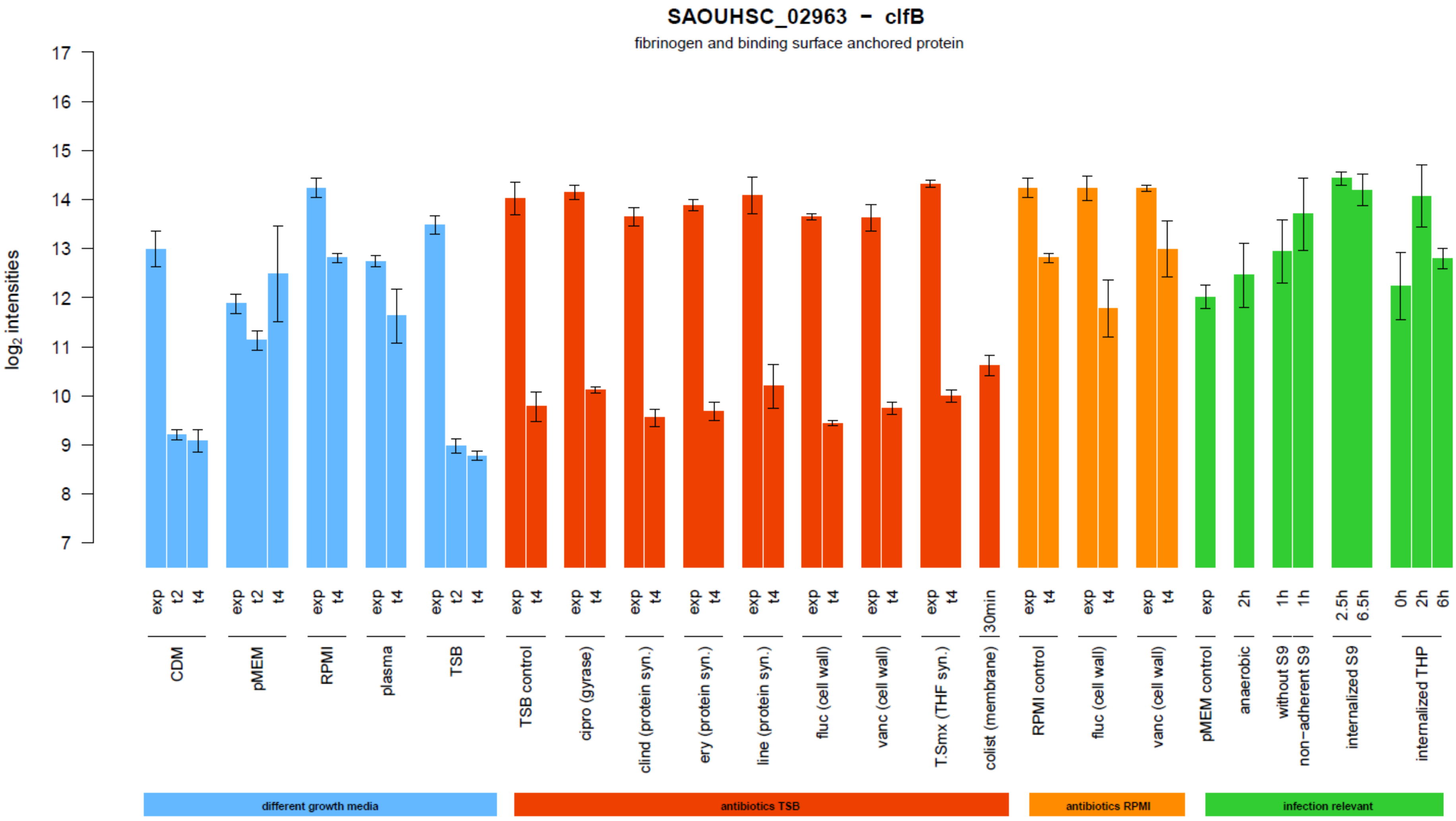
SAOUHSC_02963 – clfB
fibrinogen and binding surface anchored protein
log2 intensities
17
16
15
14
13
12
11
10
9
8
7
exp t2 t4
CDM
exp t2 t4
pMEM
exp t4
RPMI
exp t4
plasma
exp t2 t4
TSB
exp t4
TSB control
exp t4
cipro (gyrase)
exp t4
clind (protein syn.)
exp t4
ery (protein syn.)
exp t4
line (protein syn.)
exp t4
fluc (cell wall)
exp t4
vanc (cell wall)
exp t4
T.Smx (THF syn.)
30min
colist (membrane)
exp t4
RPMI control
exp t4
fluc (cell wall)
exp t4
vanc (cell wall)
exp
pMEM control
2h
anaerobic
1h
without S9
1h
non-adherent S9
2.5h 6.5h
internalized S9
0h 2h 6h
internalized THP
different growth media
antibiotics TSB
antibiotics RPMI
infection relevant

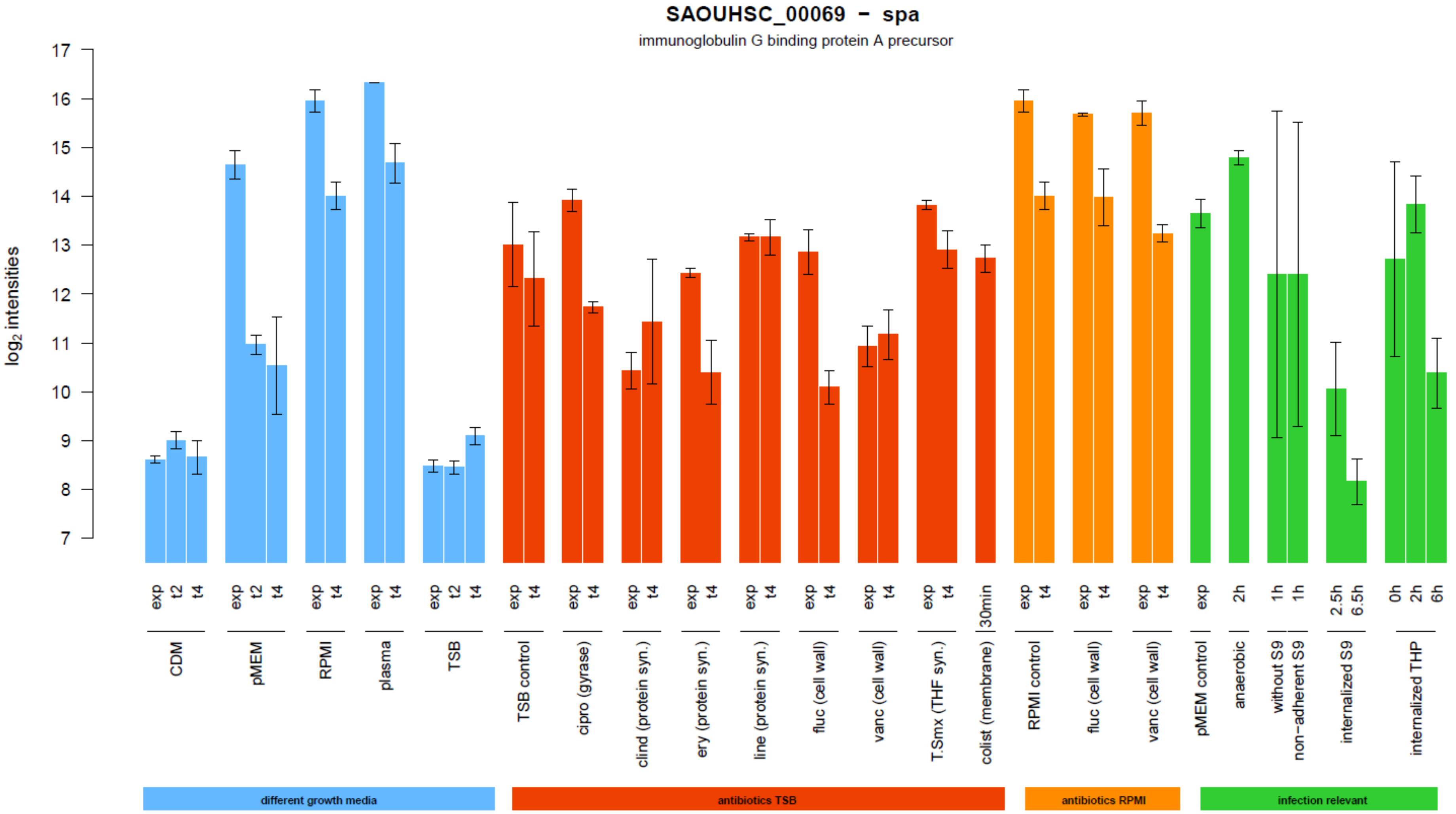
SAOUHSC_00069 – spa
immunoglobulin G binding protein A precursor
log2 intensities
17
16
15
14
13
12
11
10
9
8
7
exp t2 t4
CDM
exp t2 t4
pMEM
exp t4
RPMI
exp t4
plasma
exp t2 t4
TSB
exp t4
TSB control
exp t4
cipro (gyrase)
exp t4
clind (protein syn.)
exp t4
ery (protein syn.)
exp t4
line (protein syn.)
exp t4
fluc (cell wall)
exp t4
vanc (cell wall)
exp t4
T.Smx (THF syn.)
30min
colist (membrane)
exp t4
RPMI control
exp t4
fluc (cell wall)
exp t4
vanc (cell wall)
exp
pMEM control
2h
anaerobic
1h
without S9
1h
non−adherent S9
2.5h 6.5h
internalized S9
0h 2h 6h
internalized THP
different growth media
antibiotics TSB
antibiotics RPMI
infection relevant

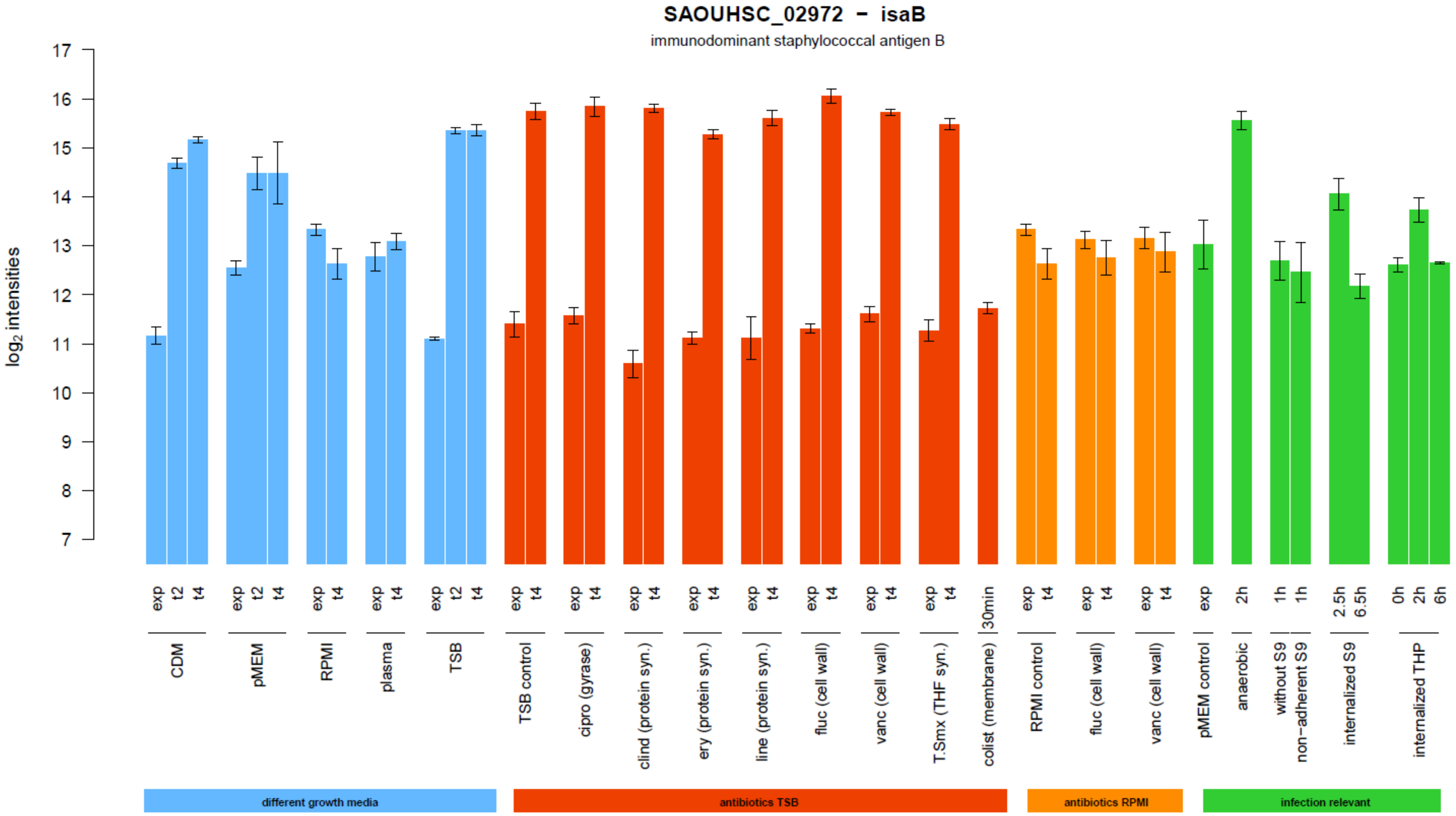

SAOUHSC_02972 - isaB
immunodominant staphylococcal antigen B
log2 intensities
17
16
15
14
13
12
11
10
9
8
7
exp t2 t4
CDM
exp t2 t4
pMEM
exp t4
RPMI
exp t4
plasma
exp t2 t4
TSB
exp t4
TSB control
exp t4
cipro (gyrase)
exp t4
clind (protein syn.)
exp t4
ery (protein syn.)
exp t4
line (protein syn.)
exp t4
fluc (cell wall)
exp t4
vanc (cell wall)
exp t4
T.Smx (THF syn.)
30min
colist (membrane)
exp t4
RPMI control
exp t4
fluc (cell wall)
exp t4
vanc (cell wall)
exp
pMEM control
2h
anaerobic
1h
without S9
1h
non-adherent S9
2.5h 6.5h
internalized S9
0h 2h 6h
internalized THP
different growth media
antibiotics TSB
antibiotics RPMI
infection relevant

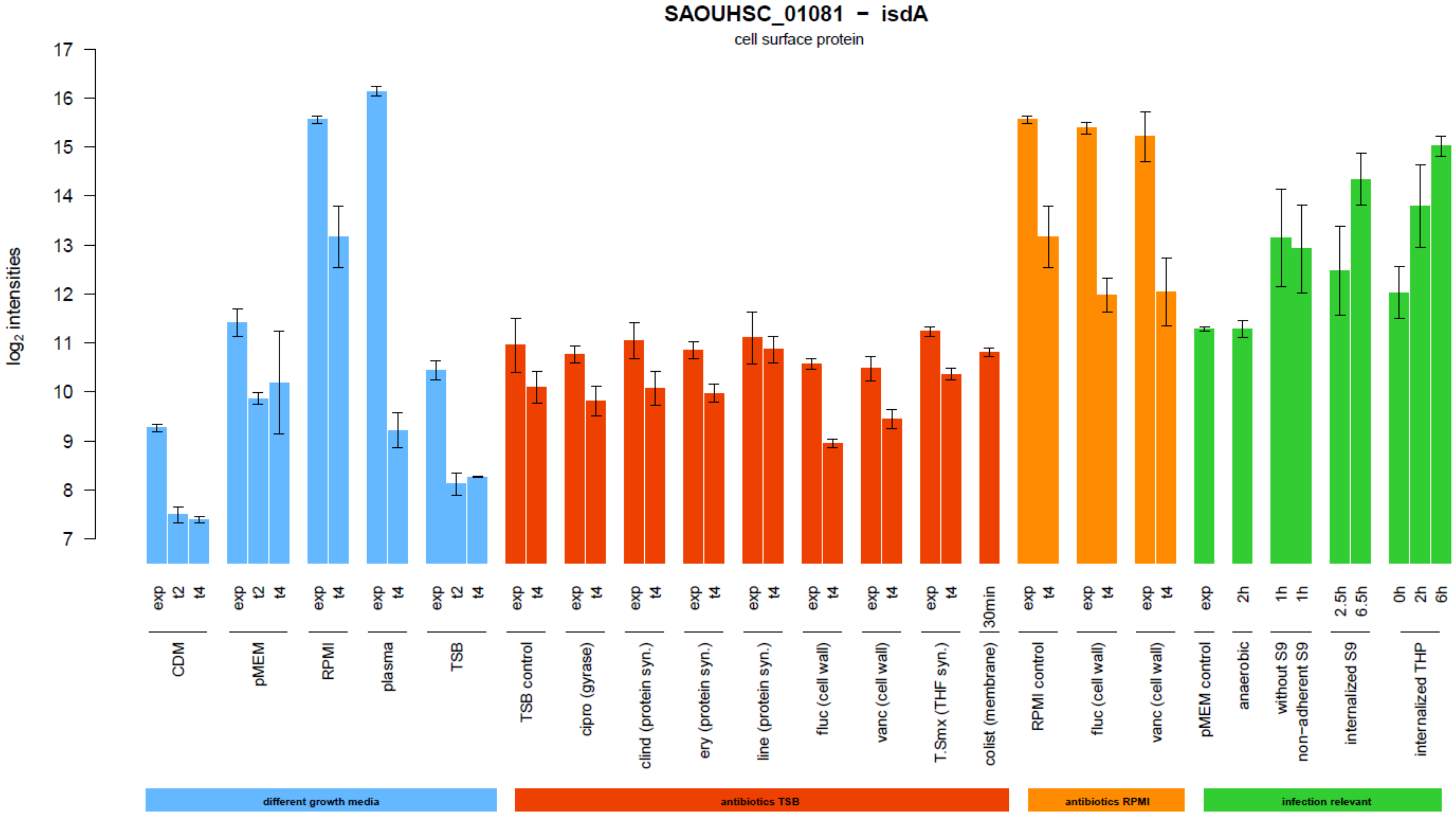
SAOUHSC_01081 – isdA
cell surface protein
log2 intensities
17
16
15
14
13
12
11
10
9
8
7
exp t2 t4
CDM
exp t2 t4
pMEM
exp t4
RPMI
exp t4
plasma
exp t2 t4
TSB
exp t4
TSB control
exp t4
cipro (gyrase)
exp t4
clind (protein syn.)
exp t4
ery (protein syn.)
exp t4
line (protein syn.)
exp t4
fluc (cell wall)
exp t4
vanc (cell wall)
exp t4
T.Smx (THF syn.)
30min
colist (membrane)
exp t4
RPMI control
exp t4
fluc (cell wall)
exp t4
vanc (cell wall)
exp
pMEM control
2h
anaerobic
1h
without S9
1h
non-adherent S9
2.5h 6.5h
internalized S9
0h 2h 6h
internalized THP
different growth media
antibiotics TSB
antibiotics RPMI
infection relevant

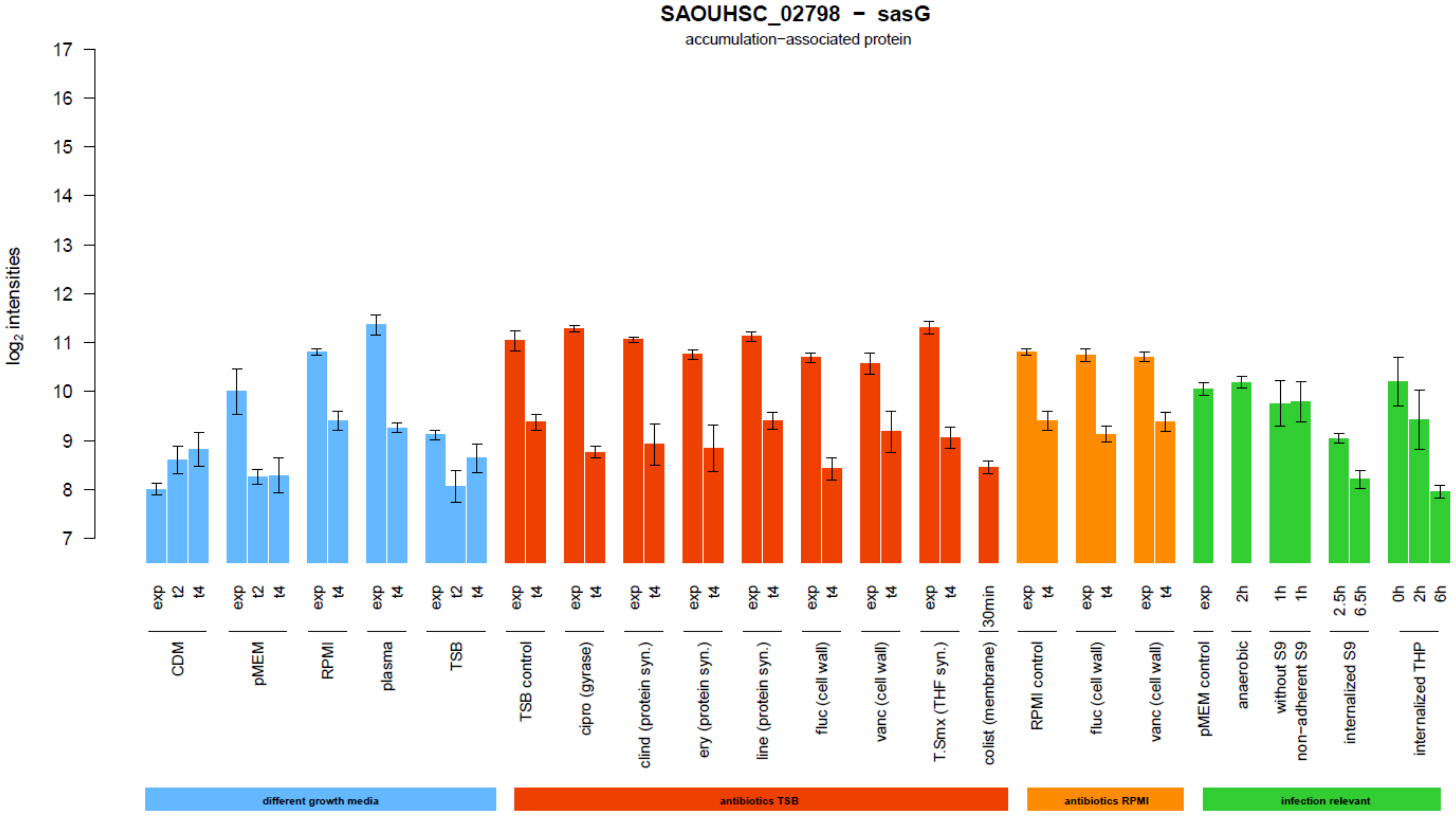
SAOUHSC_02798 - sasG
accumulation-associated protein
log2 intensities
17
16
15
14
13
12
11
10
9
8
7
exp t2 t4
CDM
exp t2 t4
pMEM
exp t4
RPMI
exp t4
plasma
exp t2 t4
TSB
exp t4
TSB control
exp t4
cipro (gyrase)
exp t4
clind (protein syn.)
exp t4
ery (protein syn.)
exp t4
line (protein syn.)
exp t4
fluc (cell wall)
exp t4
vanc (cell wall)
exp t4
T.Smx (THF syn.)
30min
colist (membrane)
exp t4
RPMI control
exp t4
fluc (cell wall)
exp t4
vanc (cell wall)
exp
pMEM control
2h
anaerobic
1h 1h
without S9
non-adherent S9
2.5h 6.5h
internalized S9
0h 2h 6h
internalized THP
different growth media
antibiotics TSB
antibiotics RPMI
infection relevant

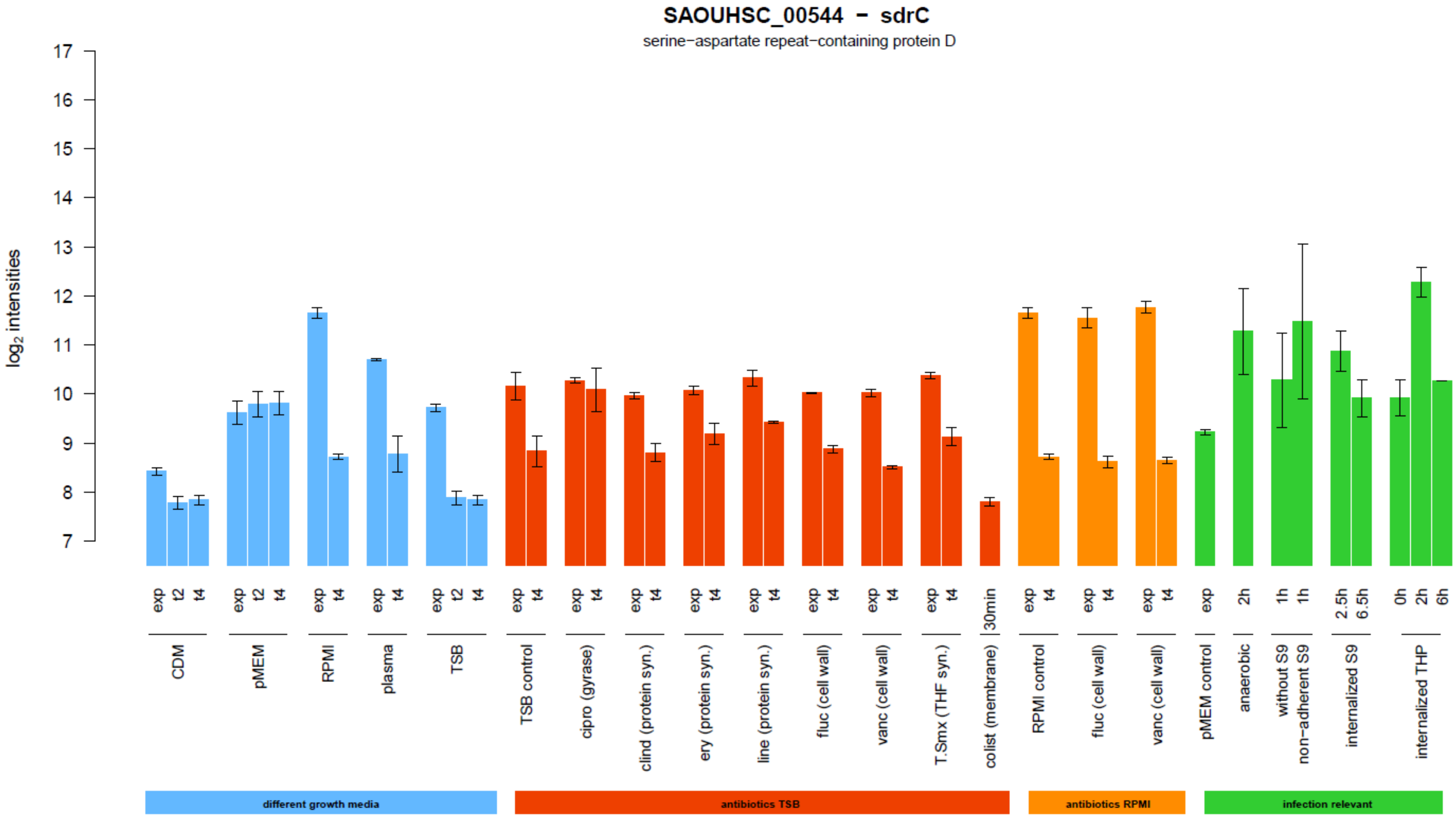

SAOUHSC_00544 - sdrC
serine-aspartate repeat-containing protein D
log2 intensities
17
16
15
14
13
12
11
10
9
8
7
exp t2 t4
CDM
exp t2 t4
pMEM
exp t4
RPMI
exp t4
plasma
exp t2 t4
TSB
exp t4
TSB control
exp t4
cipro (gyrase)
exp t4
clind (protein syn.)
exp t4
ery (protein syn.)
exp t4
line (protein syn.)
exp t4
fluc (cell wall)
exp t4
vanc (cell wall)
exp t4
T.Smx (THF syn.)
30min
colist (membrane)
exp t4
RPMI control
exp t4
fluc (cell wall)
exp t4
vanc (cell wall)
exp
pMEM control
2h
anaerobic
1h
without S9
1h
non-adherent S9
2.5h 6.5h
internalized S9
0h 2h 6h
internalized THP
different growth media
antibiotics TSB
antibiotics RPMI
infection relevant

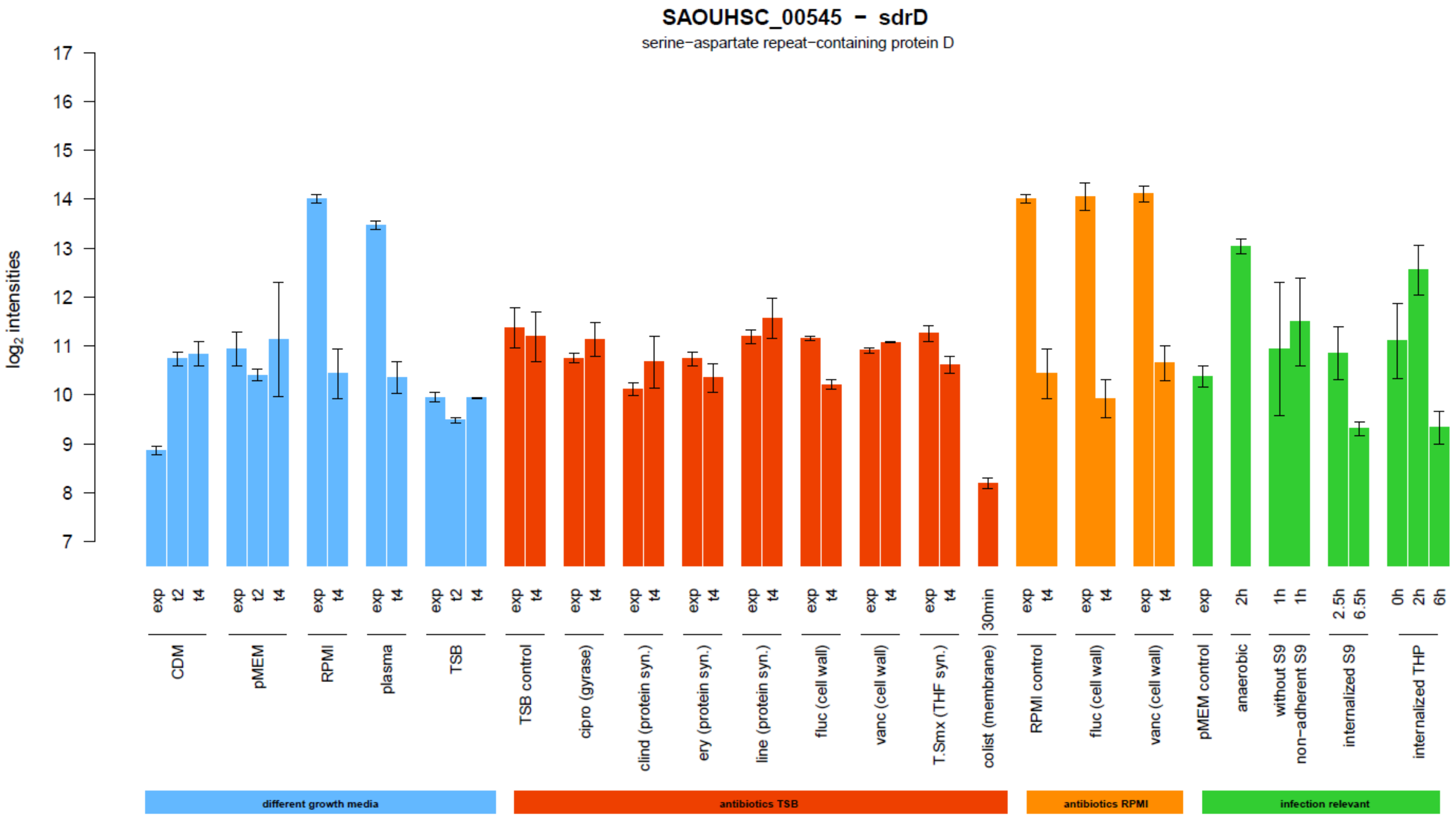
SAOUHSC_00545 – sdrD
serine−aspartate repeat−containing protein D
log2 intensities
17
16
15
14
13
12
11
10
9
8
7
exp
t2
t4
CDM
pMEM
RPMI
plasma
TSB
TSB control
cipro (gyrase)
clind (protein syn.)
ery (protein syn.)
line (protein syn.)
fluc (cell wall)
vanc (cell wall)
T.Smx (THF syn.)
30min
colist (membrane)
RPMI control
pMEM control
2h
anaerobic
1h
without S9
non−adherent S9
2.5h
6.5h
internalized S9
0h
6h
internalized THP
different growth media
antibiotics TSB
antibiotics RPMI
infection relevant

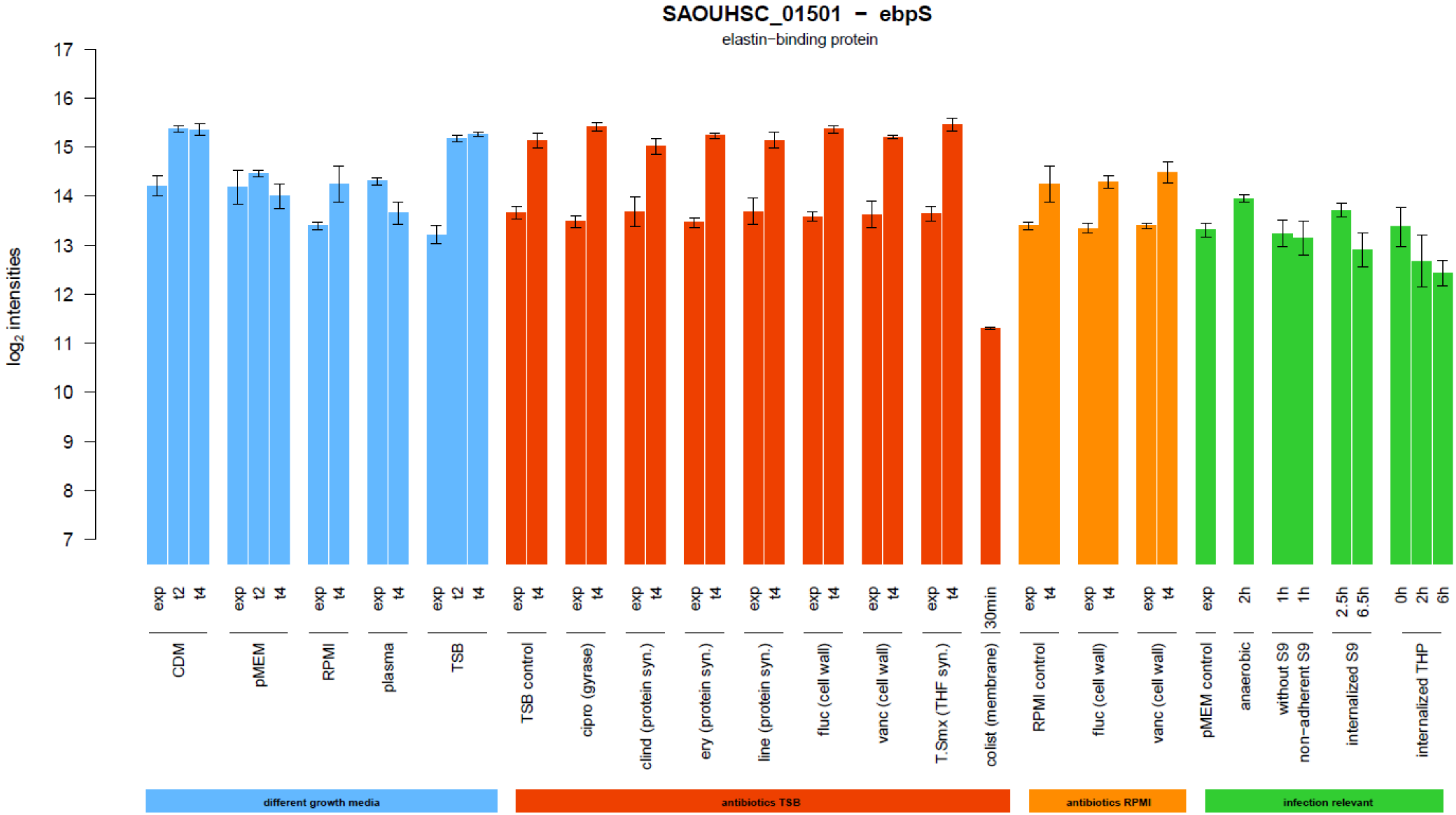
SAOUHSC_01501 – ebpS
elastin−binding protein
log2 intensities
17
16
15
14
13
12
11
10
9
8
7
exp t2 t4
CDM
exp t2 t4
pMEM
exp t4
RPMI
exp t4
plasma
exp t2 t4
TSB
exp t4
TSB control
exp t4
cipro (gyrase)
exp t4
clind (protein syn.)
exp t4
ery (protein syn.)
exp t4
line (protein syn.)
exp t4
fluc (cell wall)
exp t4
vanc (cell wall)
exp t4
T.Smx (THF syn.)
30min
colist (membrane)
exp t4
RPMI control
exp t4
fluc (cell wall)
exp t4
vanc (cell wall)
exp
pMEM control
2h
anaerobic
1h
without S9
1h
non−adherent S9
2.5h 6.5h
internalized S9
0h 2h 6h
internalized THP
different growth media
antibiotics TSB
antibiotics RPMI
infection relevant

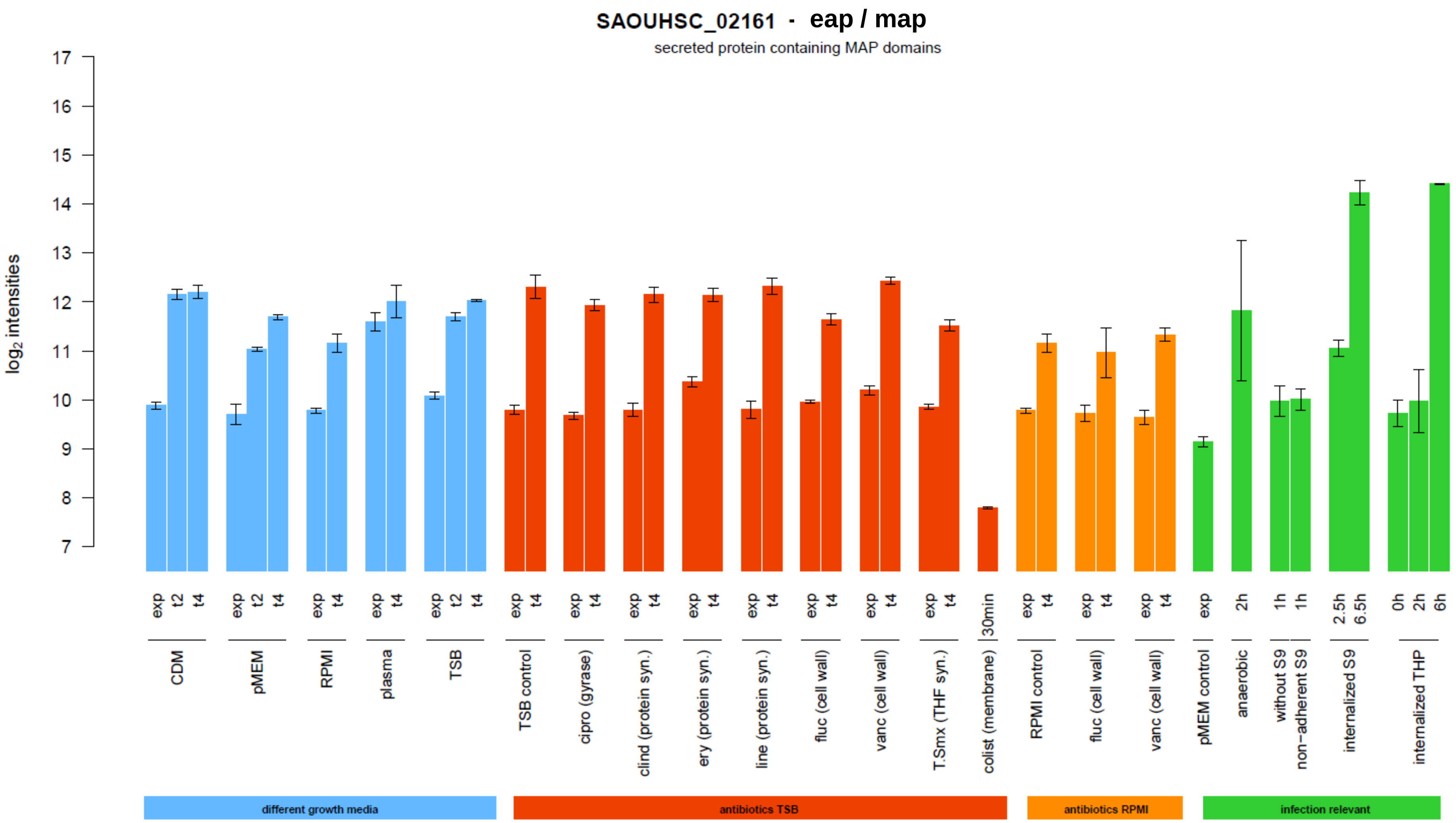
SAOUHSC_02161 · eap / map
secreted protein containing MAP domains
log2 intensities
17
16
15
14
13
12
11
10
9
8
7
exp t2 t4
CDM
exp t2 t4
pMEM
exp t4
RPMI
exp t4
plasma
exp t2 t4
TSB
exp t4
TSB control
exp t4
cipro (gyrase)
exp t4
clind (protein syn.)
exp t4
ery (protein syn.)
exp t4
line (protein syn.)
exp t4
fluc (cell wall)
exp t4
vanc (cell wall)
exp t4
T.Smx (THF syn.)
30min
colist (membrane)
exp t4
RPMI control
exp t4
fluc (cell wall)
exp t4
vanc (cell wall)
exp
pMEM control
2h
anaerobic
1h
without S9
1h
non-adherent S9
2.5h 6.5h
internalized S9
0h 2h 6h
internalized THP
different growth media
antibiotics TSB
antibiotics RPMI
infection relevant

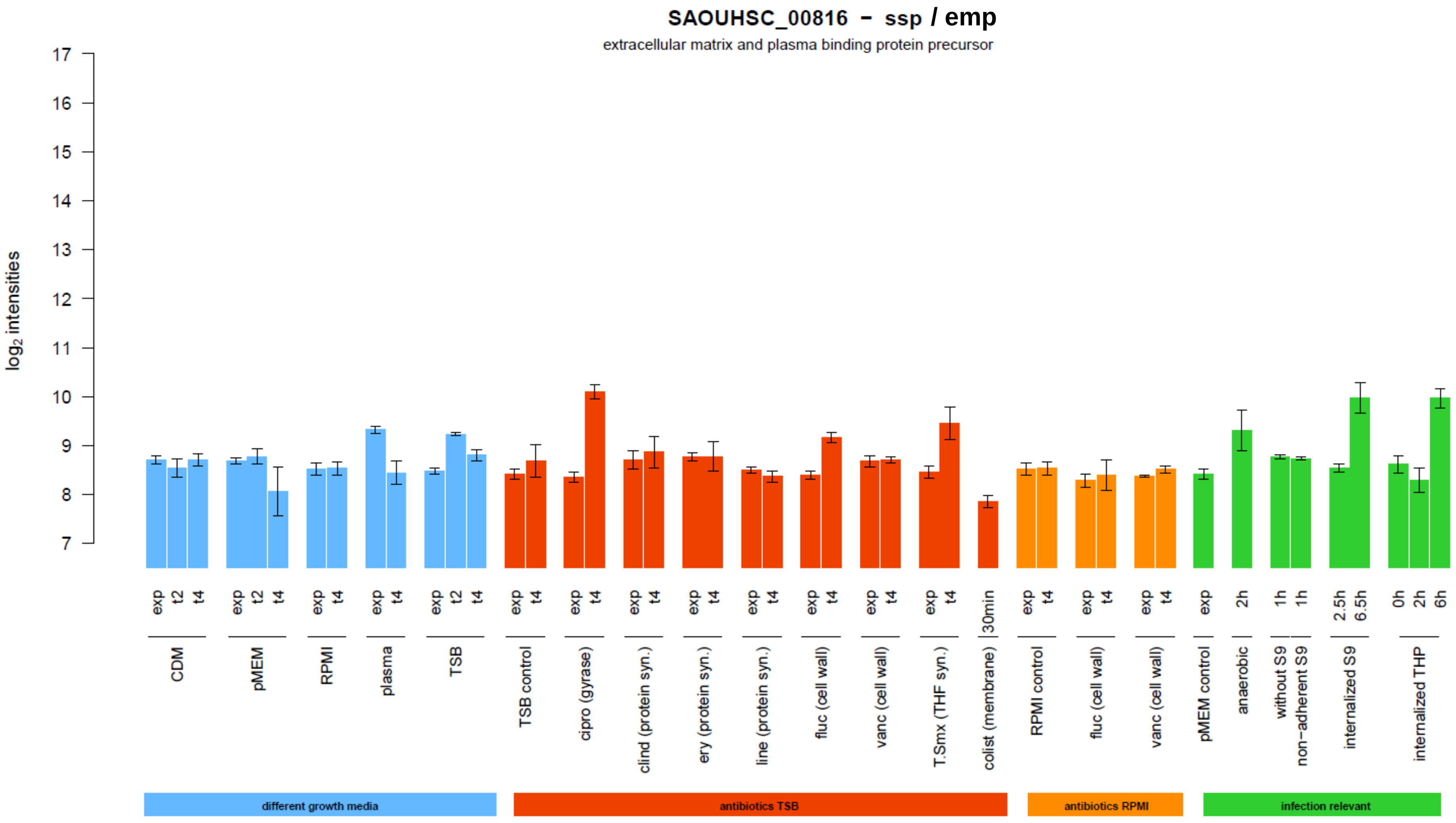
SAOUHSC_00816 – ssp / emp
extracellular matrix and plasma binding protein precursor
log2 intensities
17
16
15
14
13
12
11
10
9
8
7
exp t2 t4
CDM
exp t2 t4
pMEM
exp t4
RPMI
exp t4
plasma
exp t2 t4
TSB
exp t4
TSB control
exp t4
cipro (gyrase)
exp t4
clind (protein syn.)
exp t4
ery (protein syn.)
exp t4
line (protein syn.)
exp t4
fluc (cell wall)
exp t4
vanc (cell wall)
exp t4
T.Smx (THF syn.)
30min
colist (membrane)
exp t4
RPMI control
exp t4
fluc (cell wall)
exp t4
vanc (cell wall)
exp
pMEM control
2h
anaerobic
1h
without S9
1h
non−adherent S9
2.5h 6.5h
internalized S9
0h 2h 6h
internalized THP
different growth media
antibiotics TSB
antibiotics RPMI
infection relevant

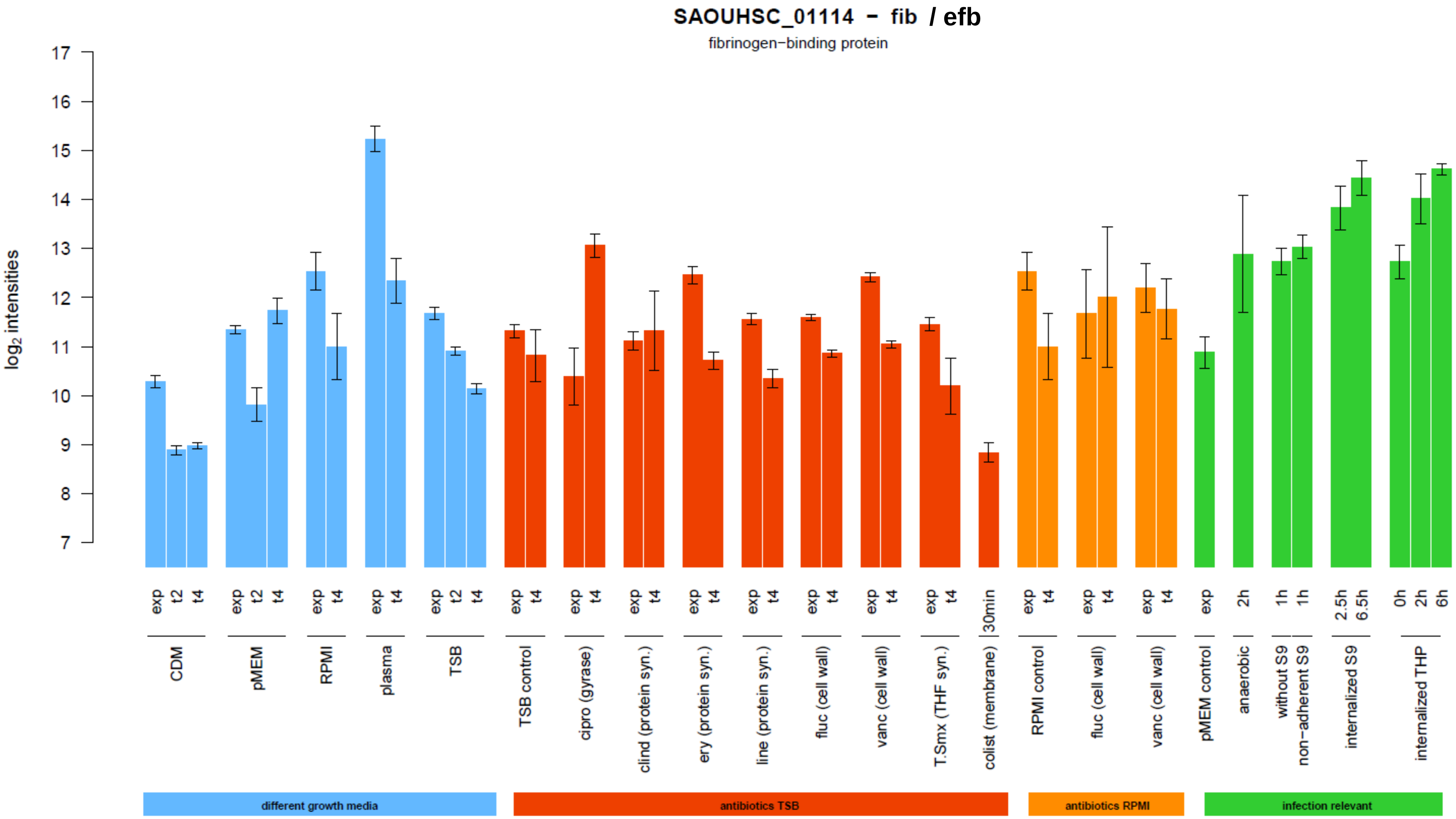
SAOUHSC_01114 – fib / efb
fibrinogen−binding protein
log2 intensities
17
16
15
14
13
12
11
10
9
8
7
exp t2 t4
CDM
exp t2 t4
pMEM
exp t4
RPMI
exp t4
plasma
exp t2 t4
TSB
exp t4
TSB control
exp t4
cipro (gyrase)
exp t4
clind (protein syn.)
exp t4
ery (protein syn.)
exp t4
line (protein syn.)
exp t4
fluc (cell wall)
exp t4
vanc (cell wall)
exp t4
T.Smx (THF syn.)
30min
colist (membrane)
exp t4
RPMI control
exp t4
fluc (cell wall)
exp t4
vanc (cell wall)
exp
pMEM control
2h
anaerobic
1h
without S9
1h
non−adherent S9
2.5h 6.5h
internalized S9
0h 2h 6h
internalized THP
different growth media
antibiotics TSB
antibiotics RPMI
infection relevant

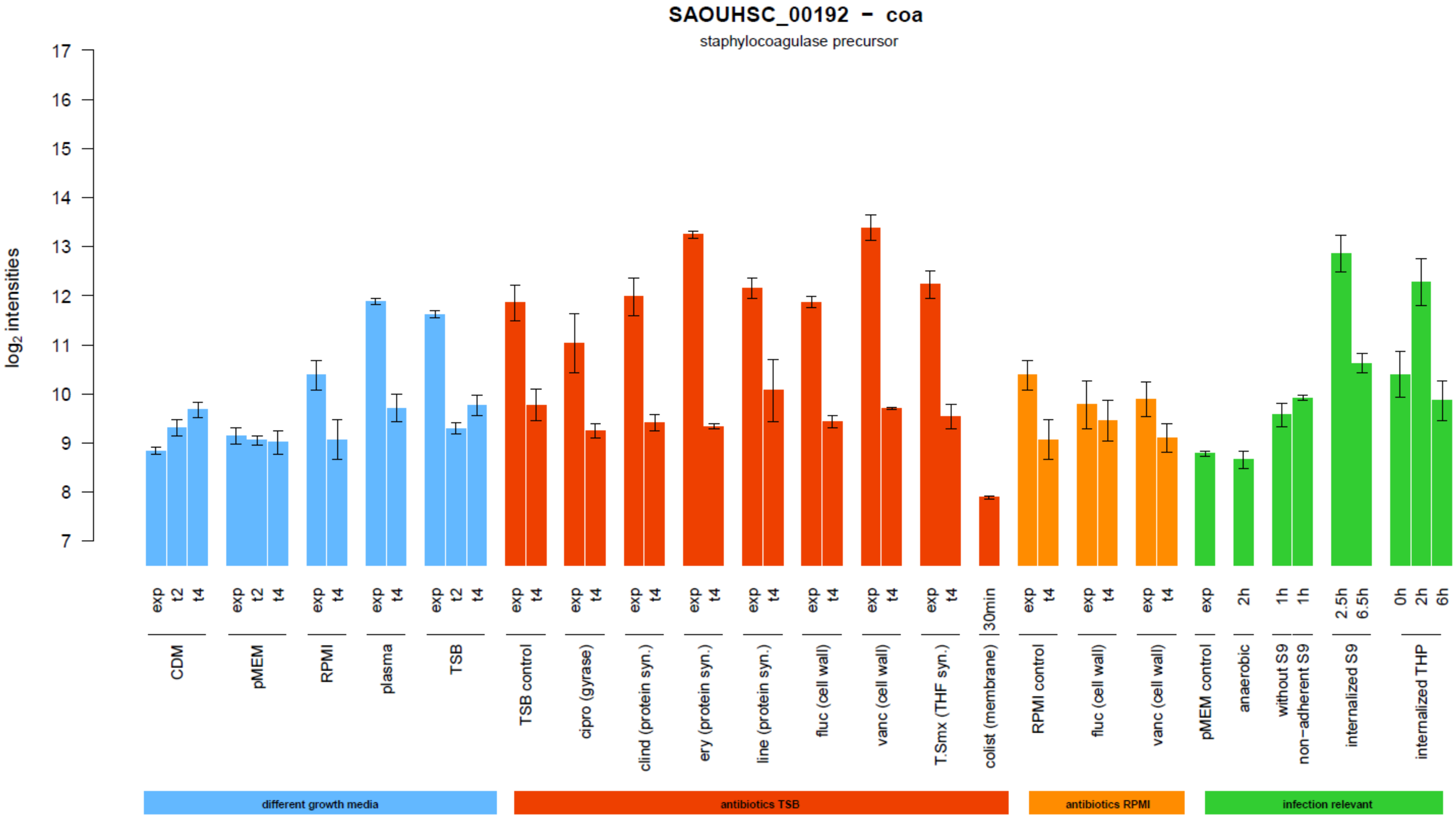

SAOUHSC_00192 – coa
staphylocoagulase precursor
log2 intensities
17
16
15
14
13
12
11
10
9
8
7
exp t2 t4
CDM
exp t2 t4
pMEM
exp t4
RPMI
exp t4
plasma
exp t2 t4
TSB
exp t4
TSB control
exp t4
cipro (gyrase)
exp t4
clind (protein syn.)
exp t4
ery (protein syn.)
exp t4
line (protein syn.)
exp t4
fluc (cell wall)
exp t4
vanc (cell wall)
exp t4
T.Smx (THF syn.)
30min
colist (membrane)
exp t4
RPMI control
exp t4
fluc (cell wall)
exp t4
vanc (cell wall)
exp
pMEM control
2h
anaerobic
1h
without S9
1h
non−adherent S9
2.5h 6.5h
internalized S9
0h 2h 6h
internalized THP
different growth media
antibiotics TSB
antibiotics RPMI
infection relevant

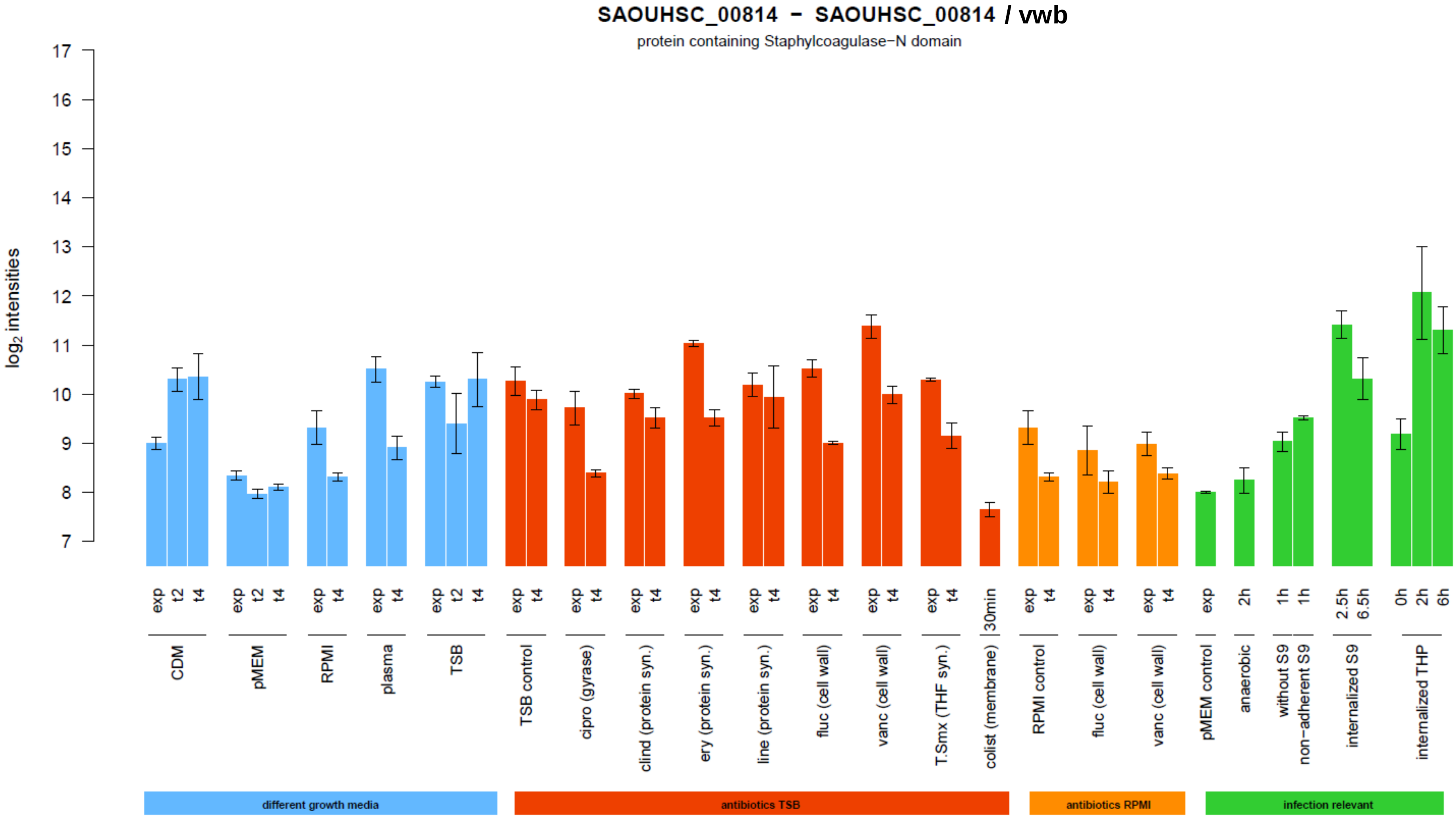

SAOUHSC_00814 – SAOUHSC_00814 / vwb
protein containing Staphylcoagulase−N domain
log2 intensities
17
16
15
14
13
12
11
10
9
8
7
exp
t2
t4
CDM
pMEM
RPMI
plasma
TSB
TSB control
cipro (gyrase)
clind (protein syn.)
ery (protein syn.)
line (protein syn.)
fluc (cell wall)
vanc (cell wall)
T.Smx (THF syn.)
30min
colist (membrane)
RPMI control
pMEM control
2h
anaerobic
1h
without S9
non−adherent S9
2.5h
6.5h
internalized S9
0h
6h
internalized THP
different growth media
antibiotics TSB
antibiotics RPMI
infection relevant

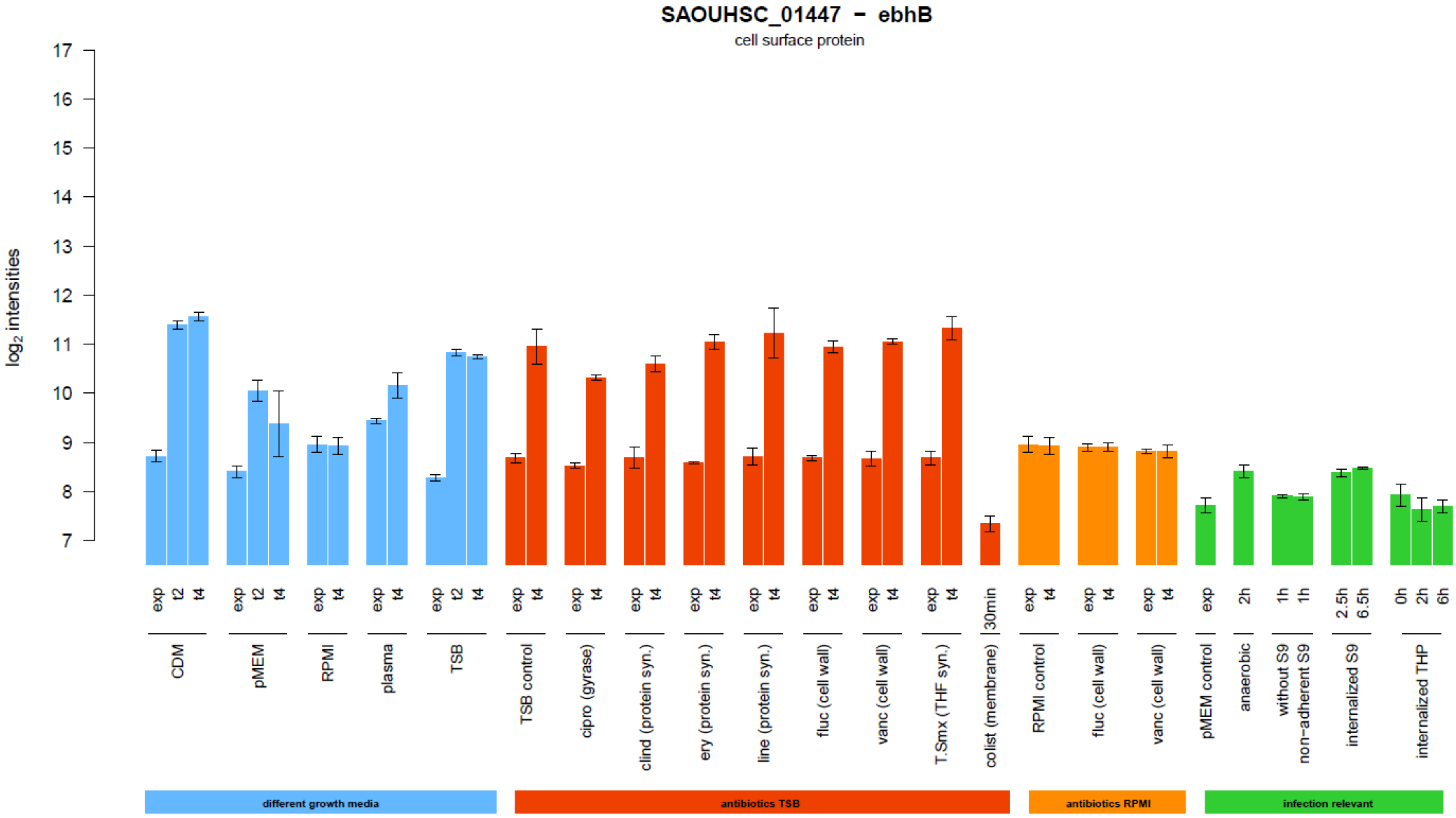

SAOUHSC_01447 – ebhB
cell surface protein
log2 intensities
17
16
15
14
13
12
11
10
9
8
7
exp t2 t4
CDM
exp t2 t4
pMEM
exp t4
RPMI
exp t4
plasma
exp t2 t4
TSB
exp t4
TSB control
exp t4
cipro (gyrase)
exp t4
clind (protein syn.)
exp t4
ery (protein syn.)
exp t4
line (protein syn.)
exp t4
fluc (cell wall)
exp t4
vanc (cell wall)
exp t4
T.Smx (THF syn.)
30min
colist (membrane)
exp t4
RPMI control
exp t4
fluc (cell wall)
exp t4
vanc (cell wall)
exp
pMEM control
2h
anaerobic
1h
without S9
1h
non−adherent S9
2.5h 6.5h
internalized S9
0h 2h 6h
internalized THP
different growth media
antibiotics TSB
antibiotics RPMI
infection relevant

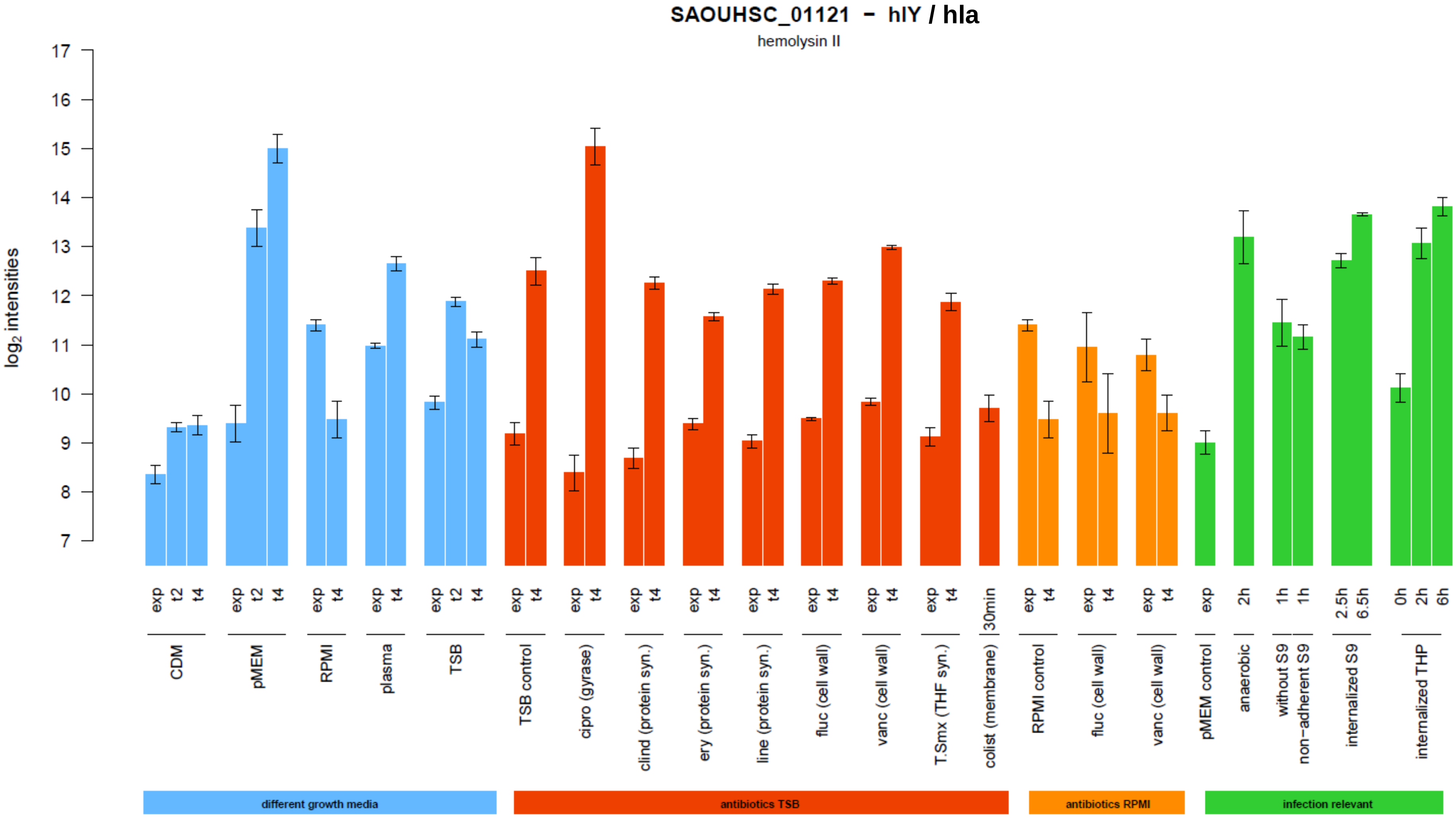

SAOUHSC_01121 – hlY / hla
hemolysin II
log2 intensities
17
16
15
14
13
12
11
10
9
8
7
exp t2 t4
CDM
exp t2 t4
pMEM
exp t4
RPMI
exp t4
plasma
exp t2 t4
TSB
exp t4
TSB control
exp t4
cipro (gyrase)
exp t4
clind (protein syn.)
exp t4
ery (protein syn.)
exp t4
line (protein syn.)
exp t4
fluc (cell wall)
exp t4
vanc (cell wall)
exp t4
T.Smx (THF syn.)
30min
colist (membrane)
exp t4
RPMI control
exp t4
fluc (cell wall)
exp t4
vanc (cell wall)
exp
pMEM control
2h
anaerobic
1h
without S9
1h
non−adherent S9
2.5h 6.5h
internalized S9
0h 2h 6h
internalized THP
different growth media
antibiotics TSB
antibiotics RPMI
infection relevant

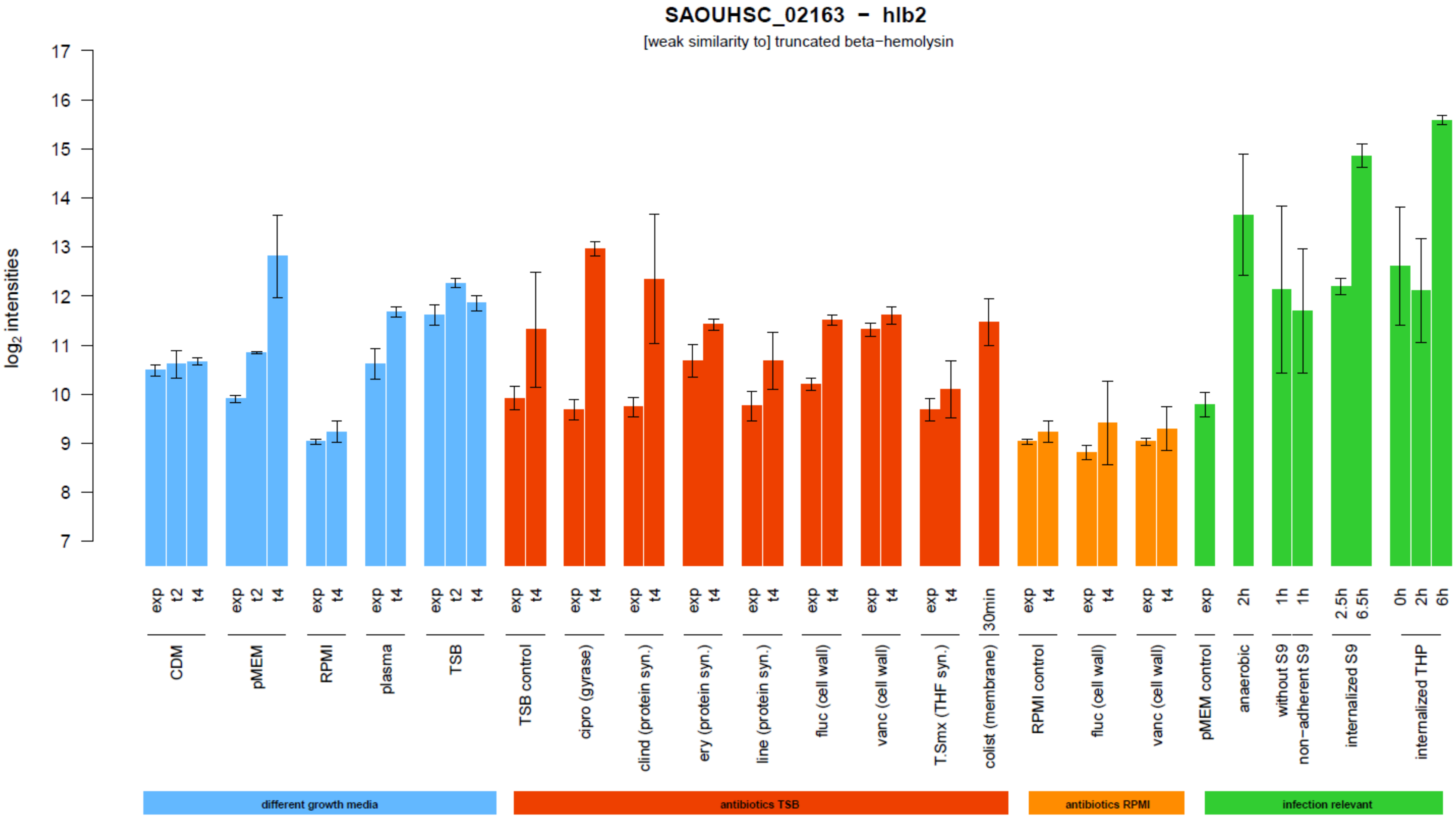

SAOUHSC_02163 - hlb2
[weak similarity to] truncated beta−hemolysin
log2 intensities
17
16
15
14
13
12
11
10
9
8
7
exp t2 t4
CDM
exp t2 t4
pMEM
exp t4
RPMI
exp t4
plasma
exp t2 t4
TSB
exp t4
TSB control
exp t4
cipro (gyrase)
exp t4
clind (protein syn.)
exp t4
ery (protein syn.)
exp t4
line (protein syn.)
exp t4
fluc (cell wall)
exp t4
vanc (cell wall)
exp t4
T.Smx (THF syn.)
30min
colist (membrane)
exp t4
RPMI control
exp t4
fluc (cell wall)
exp t4
vanc (cell wall)
exp
pMEM control
2h
anaerobic
1h
without S9
1h
non−adherent S9
2.5h 6.5h
internalized S9
0h 2h 6h
internalized THP
different growth media
antibiotics TSB
antibiotics RPMI
infection relevant

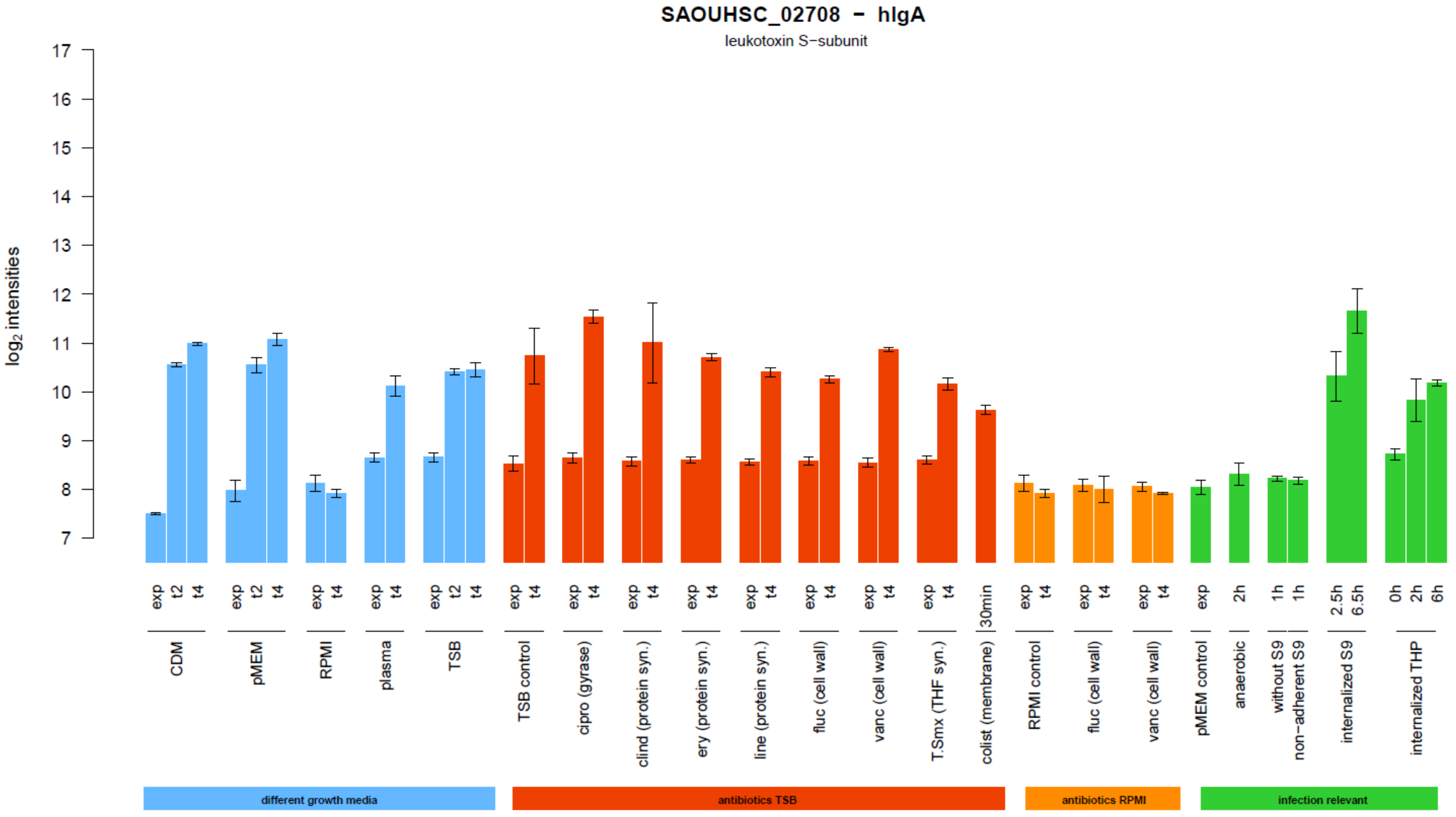
SAOUHSC_02708 – hlgA
leukotoxin S−subunit
log2 intensities
17
16
15
14
13
12
11
10
9
8
7
exp t2 t4
CDM
exp t2 t4
pMEM
exp t4
RPMI
exp t4
plasma
exp t2 t4
TSB
exp t4
TSB control
exp t4
cipro (gyrase)
exp t4
clind (protein syn.)
exp t4
ery (protein syn.)
exp t4
line (protein syn.)
exp t4
fluc (cell wall)
exp t4
vanc (cell wall)
exp t4
T.Smx (THF syn.)
30min
colist (membrane)
exp t4
RPMI control
exp t4
fluc (cell wall)
exp t4
vanc (cell wall)
exp
pMEM control
2h
anaerobic
1h
without S9
1h
non−adherent S9
2.5h 6.5h
internalized S9
0h 2h 6h
internalized THP
different growth media
antibiotics TSB
antibiotics RPMI
infection relevant

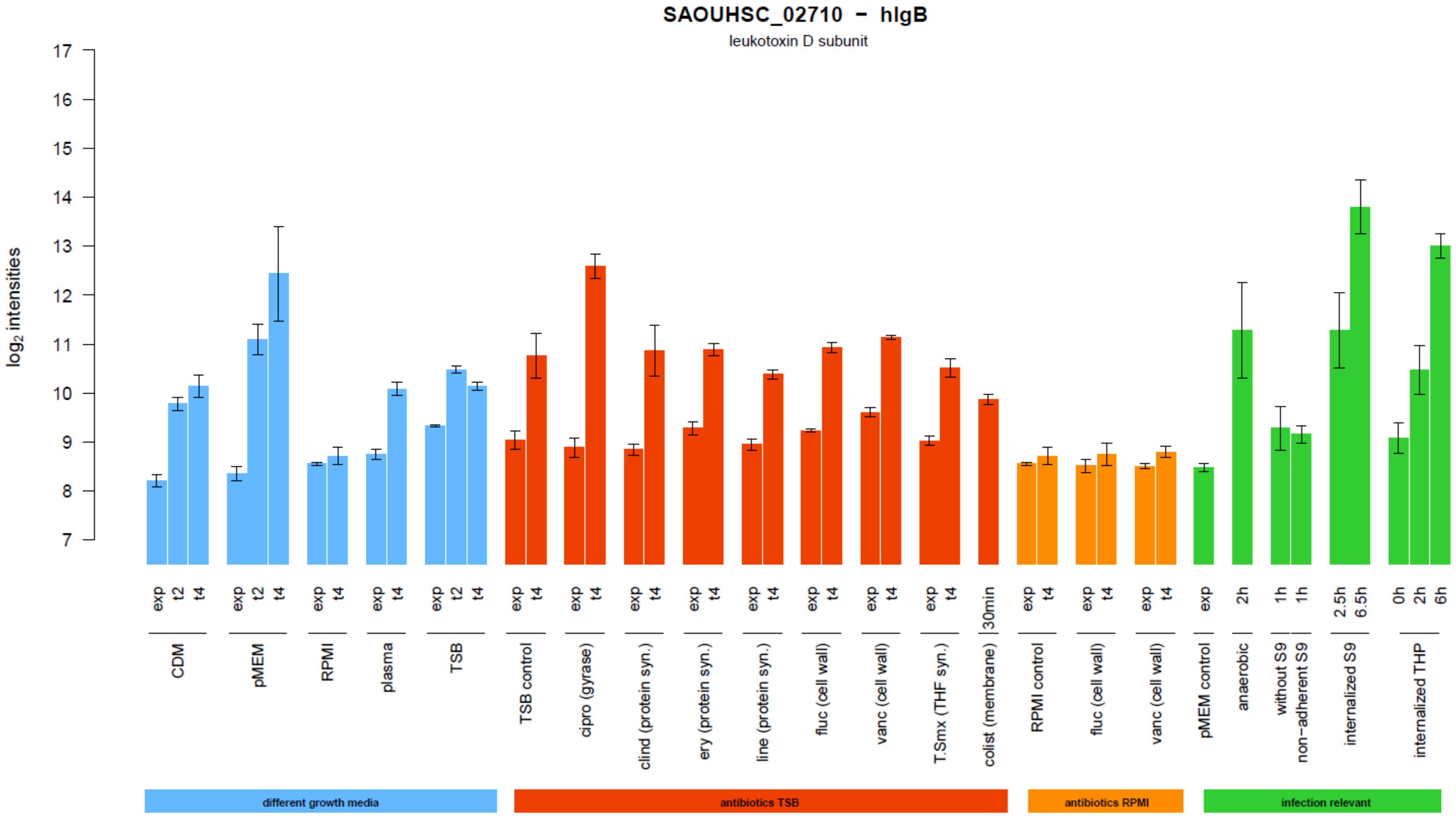
SAOUHSC_02710 – hlgB
leukotoxin D subunit
log2 intensities
17
16
15
14
13
12
11
10
9
8
7
exp t2 t4
CDM
exp t2 t4
pMEM
exp t4
RPMI
exp t4
plasma
exp t2 t4
TSB
exp t4
TSB control
exp t4
cipro (gyrase)
exp t4
clind (protein syn.)
exp t4
ery (protein syn.)
exp t4
line (protein syn.)
exp t4
fluc (cell wall)
exp t4
vanc (cell wall)
exp t4
T.Smx (THF syn.)
30min
colist (membrane)
exp t4
RPMI control
exp t4
fluc (cell wall)
exp t4
vanc (cell wall)
exp
pMEM control
2h
anaerobic
1h
without S9
1h
non−adherent S9
2.5h 6.5h
internalized S9
0h 2h 6h
internalized THP
different growth media
antibiotics TSB
antibiotics RPMI
infection relevant

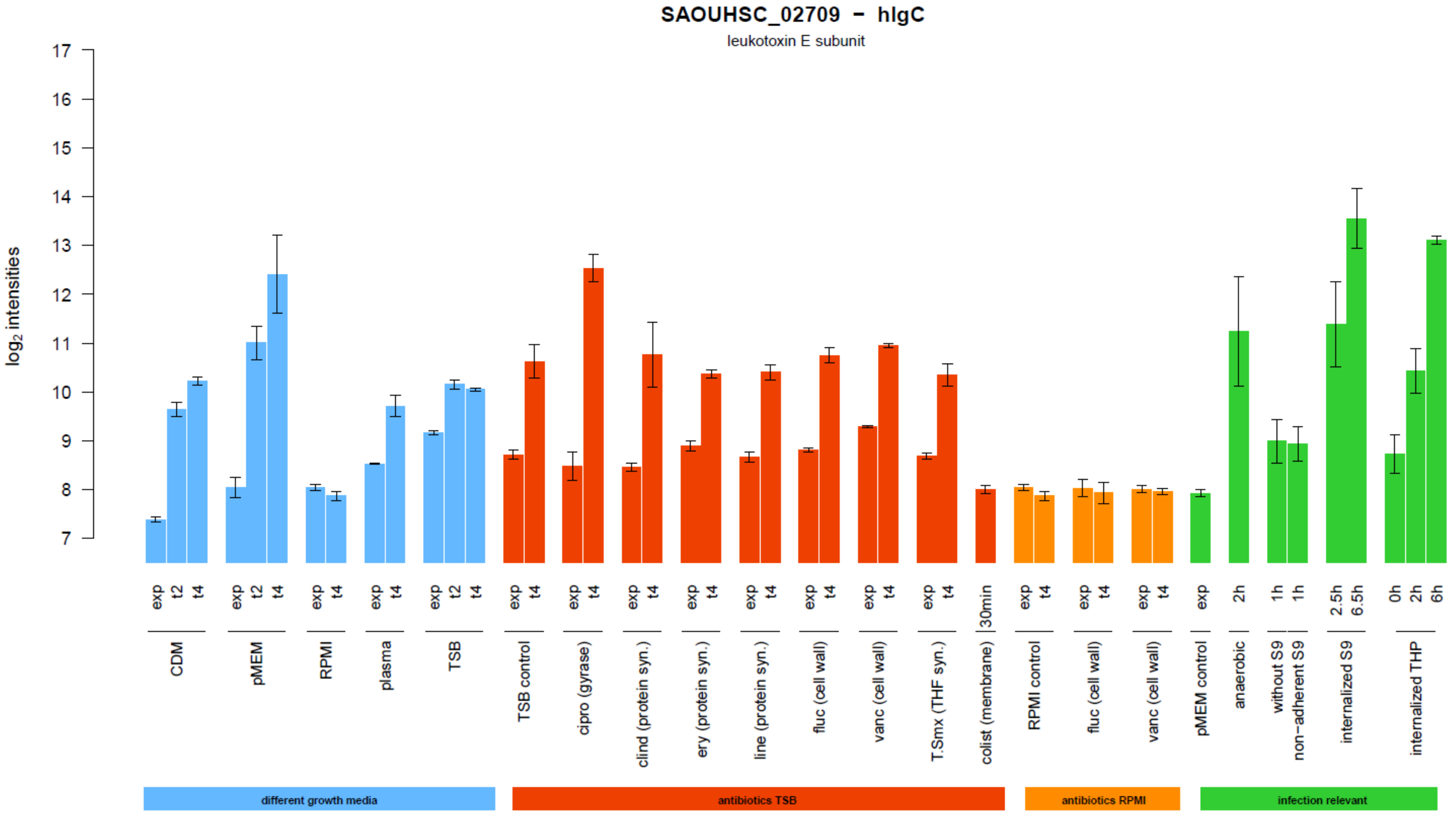
SAOUHSC_02709 – hlgC
leukotoxin E subunit
log2 intensities
17
16
15
14
13
12
11
10
9
8
7
exp t2 t4
CDM
exp t2 t4
pMEM
exp t4
RPMI
exp t4
plasma
exp t2 t4
TSB
exp t4
TSB control
exp t4
cipro (gyrase)
exp t4
clind (protein syn.)
exp t4
ery (protein syn.)
exp t4
line (protein syn.)
exp t4
fluc (cell wall)
exp t4
vanc (cell wall)
exp t4
T.Smx (THF syn.)
30min
colist (membrane)
exp t4
RPMI control
exp t4
fluc (cell wall)
exp t4
vanc (cell wall)
exp
pMEM control
2h
anaerobic
1h
without S9
1h
non-adherent S9
2.5h 6.5h
internalized S9
0h 2h 6h
internalized THP
different growth media
antibiotics TSB
antibiotics RPMI
infection relevant

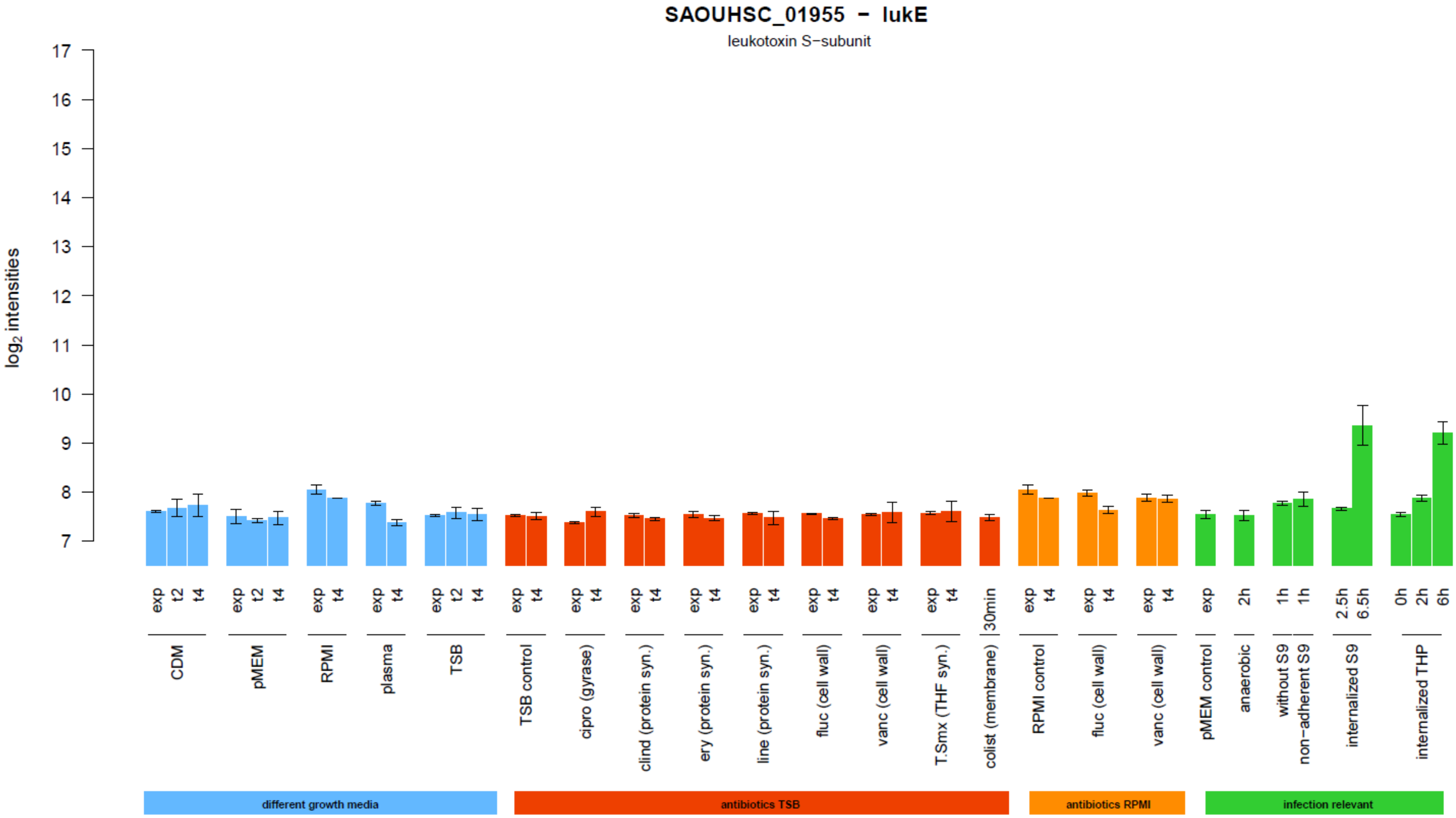
SAOUHSC_01955 – lukE
leukotoxin S–subunit
log2 intensities
17
16
15
14
13
12
11
10
9
8
7
exp t2 t4
CDM
exp t2 t4
pMEM
exp t4
RPMI
exp t4
plasma
exp t2 t4
TSB
exp t4
TSB control
exp t4
cipro (gyrase)
exp t4
clind (protein syn.)
exp t4
ery (protein syn.)
exp t4
line (protein syn.)
exp t4
fluc (cell wall)
exp t4
vanc (cell wall)
exp t4
T.Smx (THF syn.)
30min
colist (membrane)
exp t4
RPMI control
exp t4
fluc (cell wall)
exp t4
vanc (cell wall)
exp
pMEM control
2h
anaerobic
1h
without S9
1h
non–adherent S9
2.5h 6.5h
internalized S9
0h 2h 6h
internalized THP
different growth media
antibiotics TSB
antibiotics RPMI
infection relevant

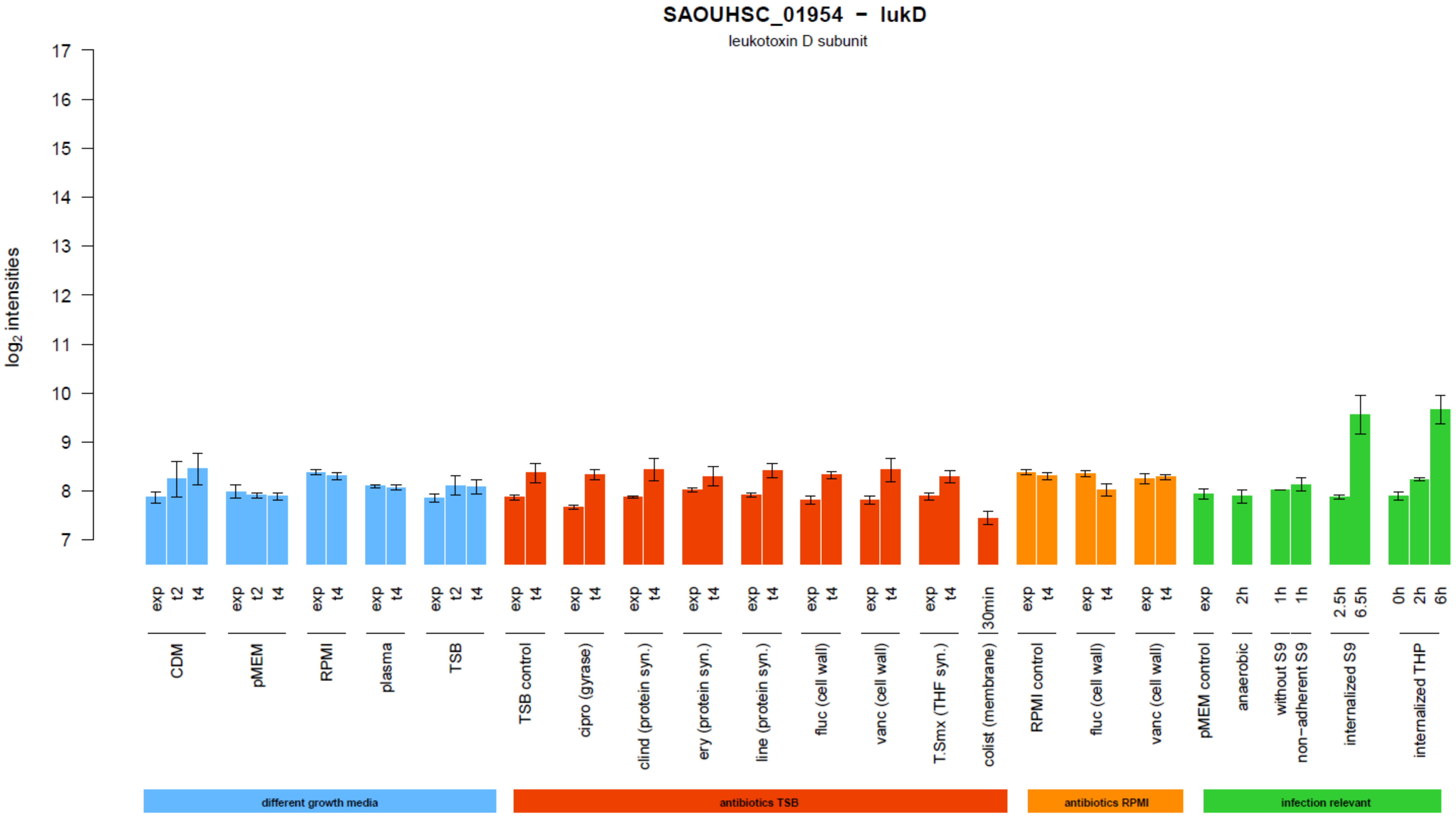
SAOUHSC_01954 – lukD
leukotoxin D subunit
log2 intensities
17
16
15
14
13
12
11
10
9
8
7
exp t2 t4
CDM
exp t2 t4
pMEM
exp t4
RPMI
exp t4
plasma
exp t2 t4
TSB
exp t4
TSB control
exp t4
cipro (gyrase)
exp t4
clind (protein syn.)
exp t4
ery (protein syn.)
exp t4
line (protein syn.)
exp t4
fluc (cell wall)
exp t4
vanc (cell wall)
exp t4
T.Smx (THF syn.)
30min
colist (membrane)
exp t4
RPMI control
exp t4
fluc (cell wall)
exp t4
vanc (cell wall)
exp
pMEM control
2h
anaerobic
1h
without S9
1h
non−adherent S9
2.5h 6.5h
internalized S9
0h 2h 6h
internalized THP
different growth media
antibiotics TSB
antibiotics RPMI
infection relevant

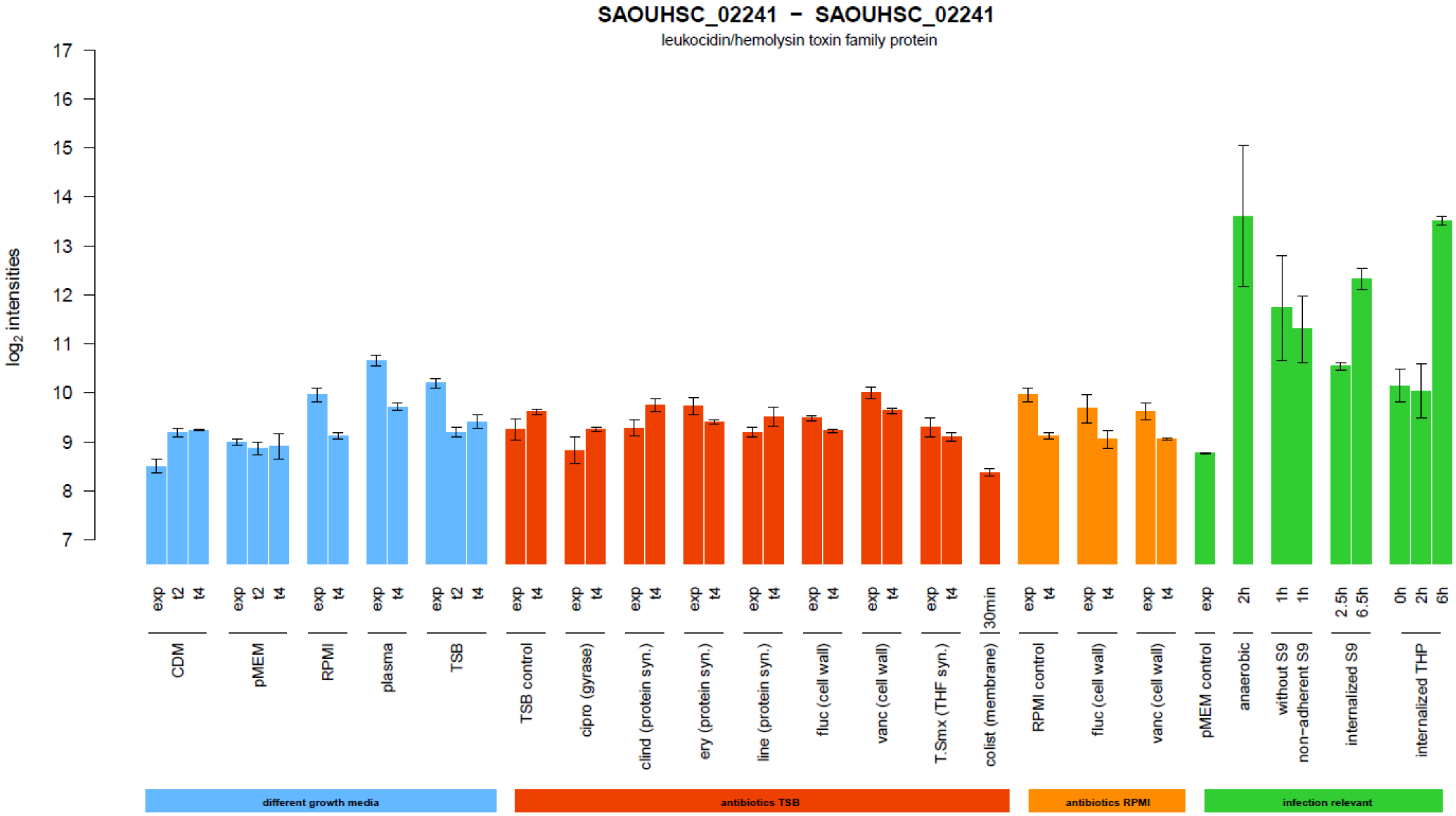
SAOUHSC_02241 – SAOUHSC_02241
leukocidin/hemolysin toxin family protein
log2 intensities
7
8
9
10
11
12
13
14
15
16
17
exp t2 t4
exp t2 t4
exp t4
exp t4
exp t2 t4
exp t4
exp t4
exp t4
exp t4
exp t4
exp t4
exp t4
exp t4
30min
exp t4
exp t4
exp t4
exp
2h
1h 1h
2.5h 6.5h
0h 2h 6h
CDM
pMEM
RPMI
plasma
TSB
TSB control
cipro (gyrase)
clind (protein syn.)
ery (protein syn.)
line (protein syn.)
fluc (cell wall)
vanc (cell wall)
T.Smx (THF syn.)
colist (membrane)
RPMI control
fluc (cell wall)
vanc (cell wall)
pMEM control
anaerobic
without S9
non−adherent S9
internalized S9
internalized THP
different growth media
antibiotics TSB
antibiotics RPMI
infection relevant

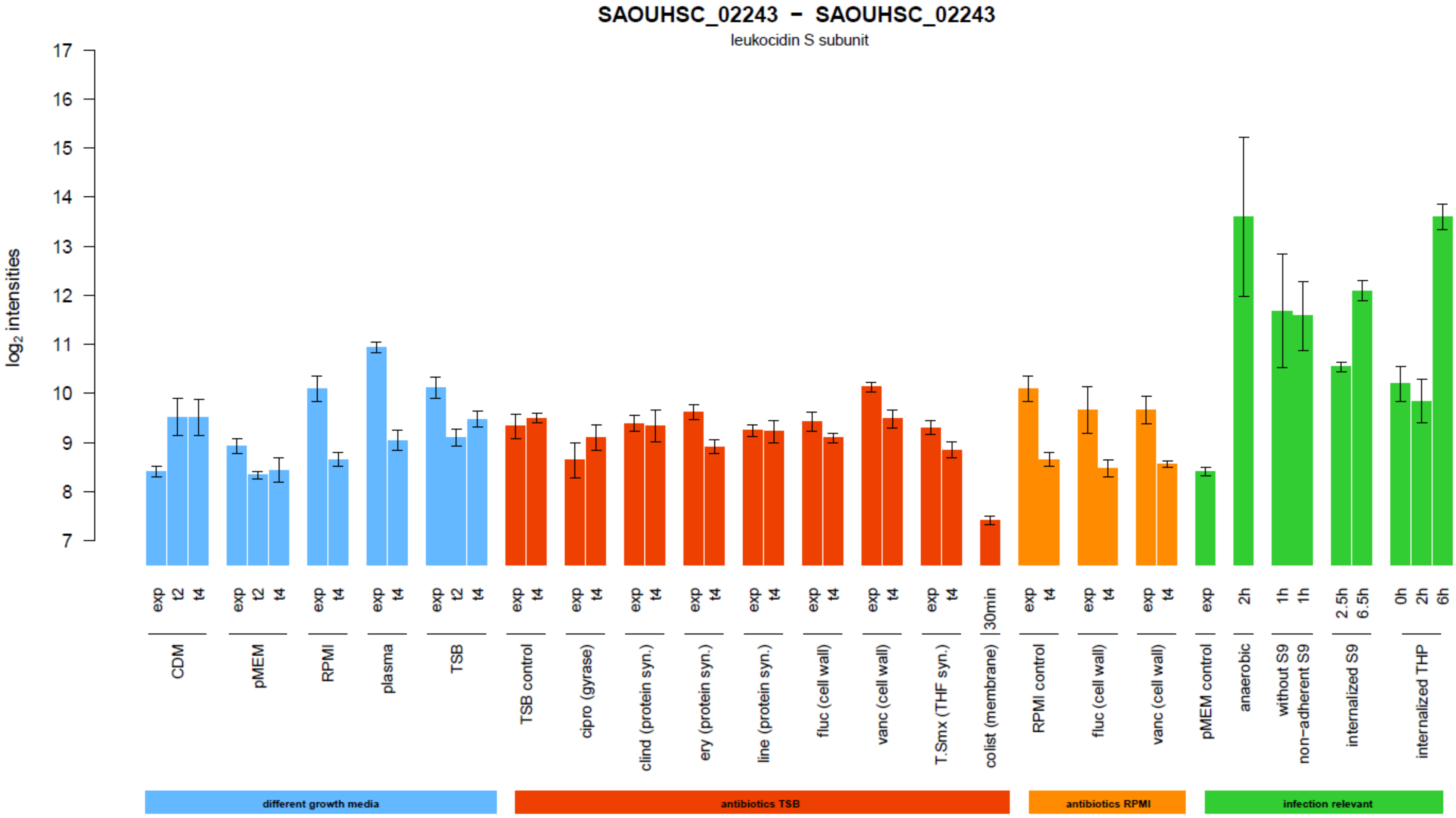
SAOUHSC_02243 – SAOUHSC_02243
leukocidin S subunit
log2 intensities
17
16
15
14
13
12
11
10
9
8
7
exp
t2
t4
CDM
pMEM
RPMI
plasma
TSB
TSB control
cipro (gyrase)
clind (protein syn.)
ery (protein syn.)
line (protein syn.)
fluc (cell wall)
vanc (cell wall)
T.Smx (THF syn.)
30min
colist (membrane)
RPMI control
pMEM control
2h
anaerobic
1h
without S9
non−adherent S9
2.5h
6.5h
internalized S9
0h
6h
internalized THP
different growth media
antibiotics TSB
antibiotics RPMI
infection relevant

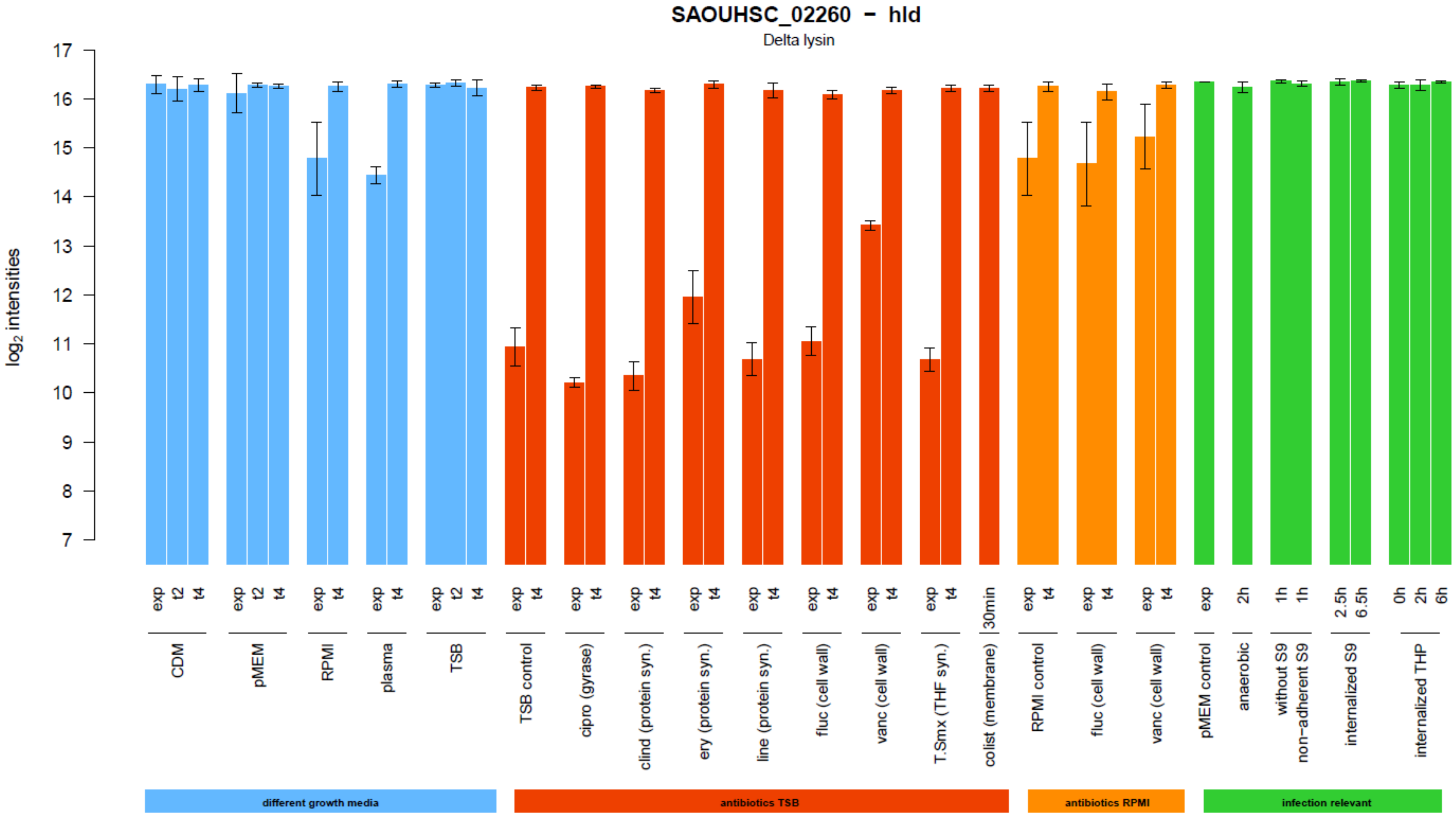

SAOUHSC_02260 – hld
Delta lysin
log2 intensities
17
16
15
14
13
12
11
10
9
8
7
exp
t2
t4
CDM
pMEM
RPMI
plasma
TSB
TSB control
cipro (gyrase)
clind (protein syn.)
ery (protein syn.)
line (protein syn.)
fluc (cell wall)
vanc (cell wall)
T.Smx (THF syn.)
30min
colist (membrane)
RPMI control
pMEM control
2h
anaerobic
1h
without S9
non−adherent S9
2.5h
6.5h
internalized S9
0h
6h
internalized THP
different growth media
antibiotics TSB
antibiotics RPMI
infection relevant

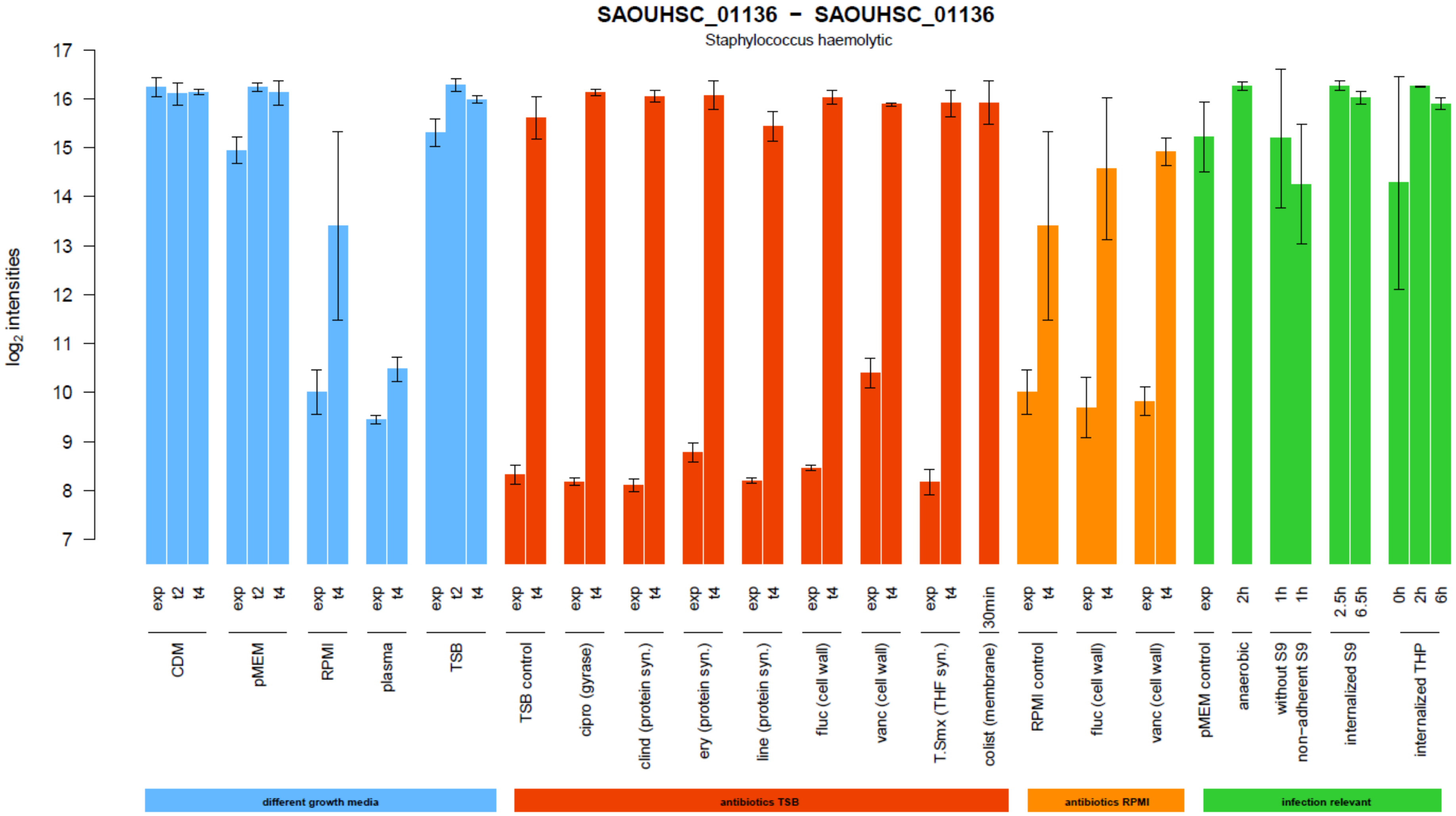

SAOUHSC_01136 – SAOUHSC_01136
Staphylococcus haemolytic
log2 intensities
17
16
15
14
13
12
11
10
9
8
7
exp t2 t4
CDM
exp t2 t4
pMEM
exp t4
RPMI
exp t4
plasma
exp t2 t4
TSB
exp t4
TSB control
exp t4
cipro (gyrase)
exp t4
clind (protein syn.)
exp t4
ery (protein syn.)
exp t4
line (protein syn.)
exp t4
fluc (cell wall)
exp t4
vanc (cell wall)
exp t4
T.Smx (THF syn.)
30min
colist (membrane)
exp t4
RPMI control
exp t4
fluc (cell wall)
exp t4
vanc (cell wall)
exp
pMEM control
2h
anaerobic
1h
without S9
1h
non−adherent S9
2.5h 6.5h
internalized S9
0h 2h 6h
internalized THP
different growth media
antibiotics TSB
antibiotics RPMI
infection relevant

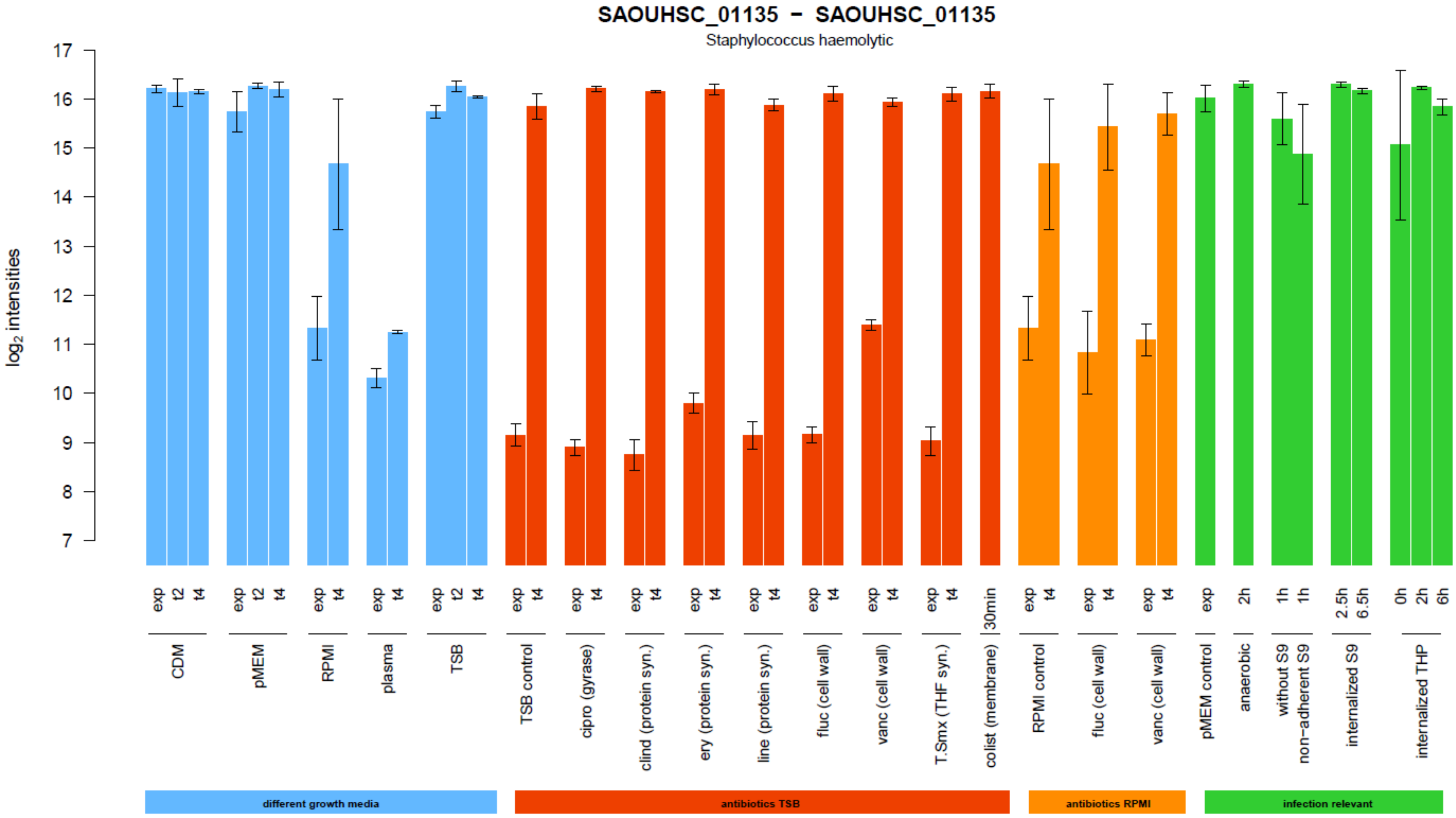

SAOUHSC_01135 – SAOUHSC_01135
Staphylococcus haemolytic
log2 intensities
17
16
15
14
13
12
11
10
9
8
7
exp t2 t4
CDM
exp t2 t4
pMEM
exp t4
RPMI
exp t4
plasma
exp t2 t4
TSB
exp t4
TSB control
exp t4
cipro (gyrase)
exp t4
clind (protein syn.)
exp t4
ery (protein syn.)
exp t4
line (protein syn.)
exp t4
fluc (cell wall)
exp t4
vanc (cell wall)
exp t4
T.Smx (THF syn.)
30min
colist (membrane)
exp t4
RPMI control
exp t4
fluc (cell wall)
exp t4
vanc (cell wall)
exp
pMEM control
2h
anaerobic
1h
without S9
1h
non-adherent S9
2.5h 6.5h
internalized S9
0h 2h 6h
internalized THP
different growth media
antibiotics TSB
antibiotics RPMI
infection relevant

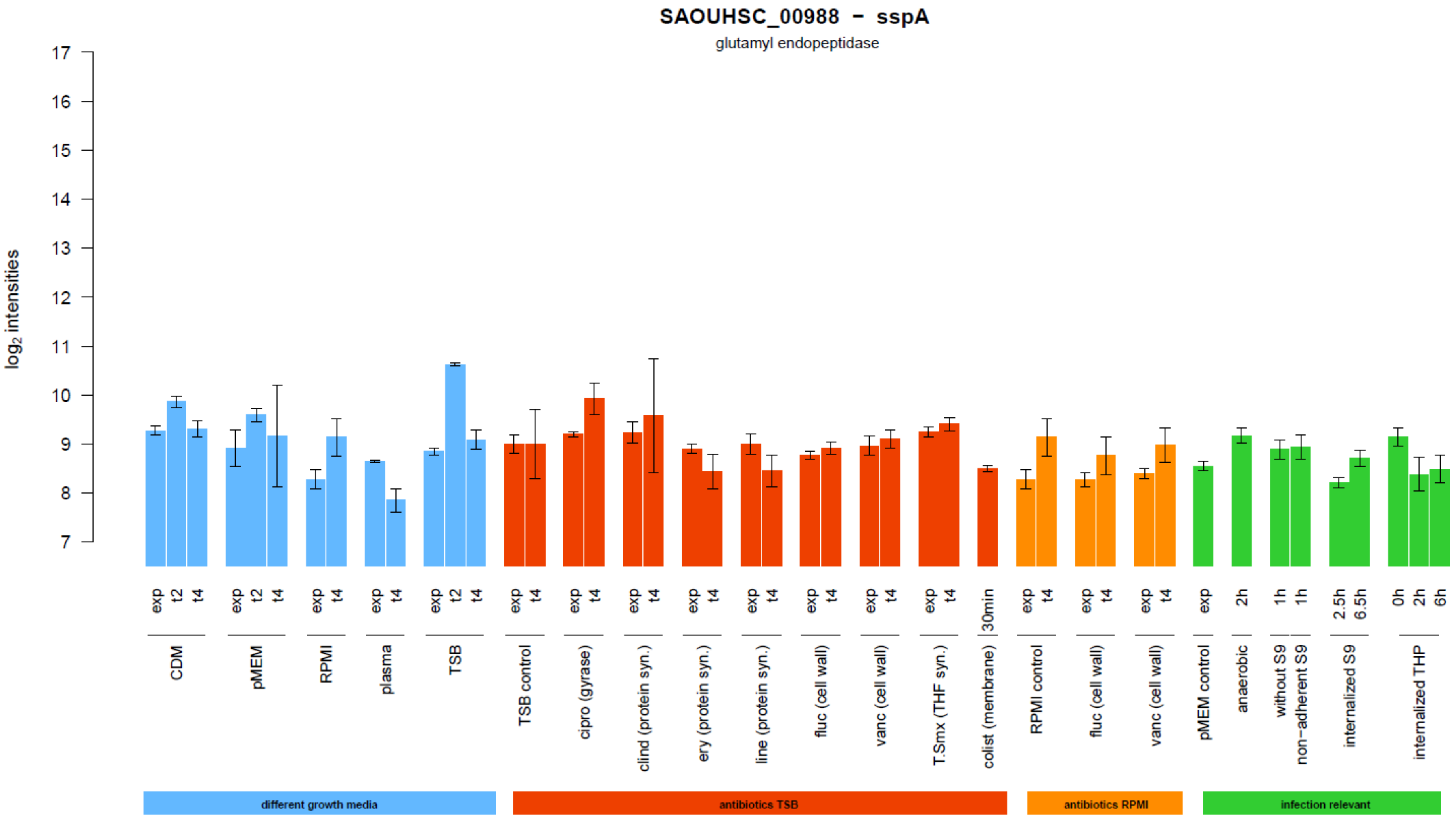
SAOUHSC_00988 - sspA
glutamyl endopeptidase
log2 intensities
17
16
15
14
13
12
11
10
9
8
7
exp t2 t4
CDM
exp t2 t4
pMEM
exp t4
RPMI
exp t4
plasma
exp t2 t4
TSB
exp t4
TSB control
exp t4
cipro (gyrase)
exp t4
clind (protein syn.)
exp t4
ery (protein syn.)
exp t4
line (protein syn.)
exp t4
fluc (cell wall)
exp t4
vanc (cell wall)
exp t4
T.Smx (THF syn.)
30min
colist (membrane)
exp t4
RPMI control
exp t4
fluc (cell wall)
exp t4
vanc (cell wall)
exp
pMEM control
2h
anaerobic
1h
without S9
1h
non-adherent S9
2.5h 6.5h
internalized S9
0h 2h 6h
internalized THP
different growth media
antibiotics TSB
antibiotics RPMI
infection relevant

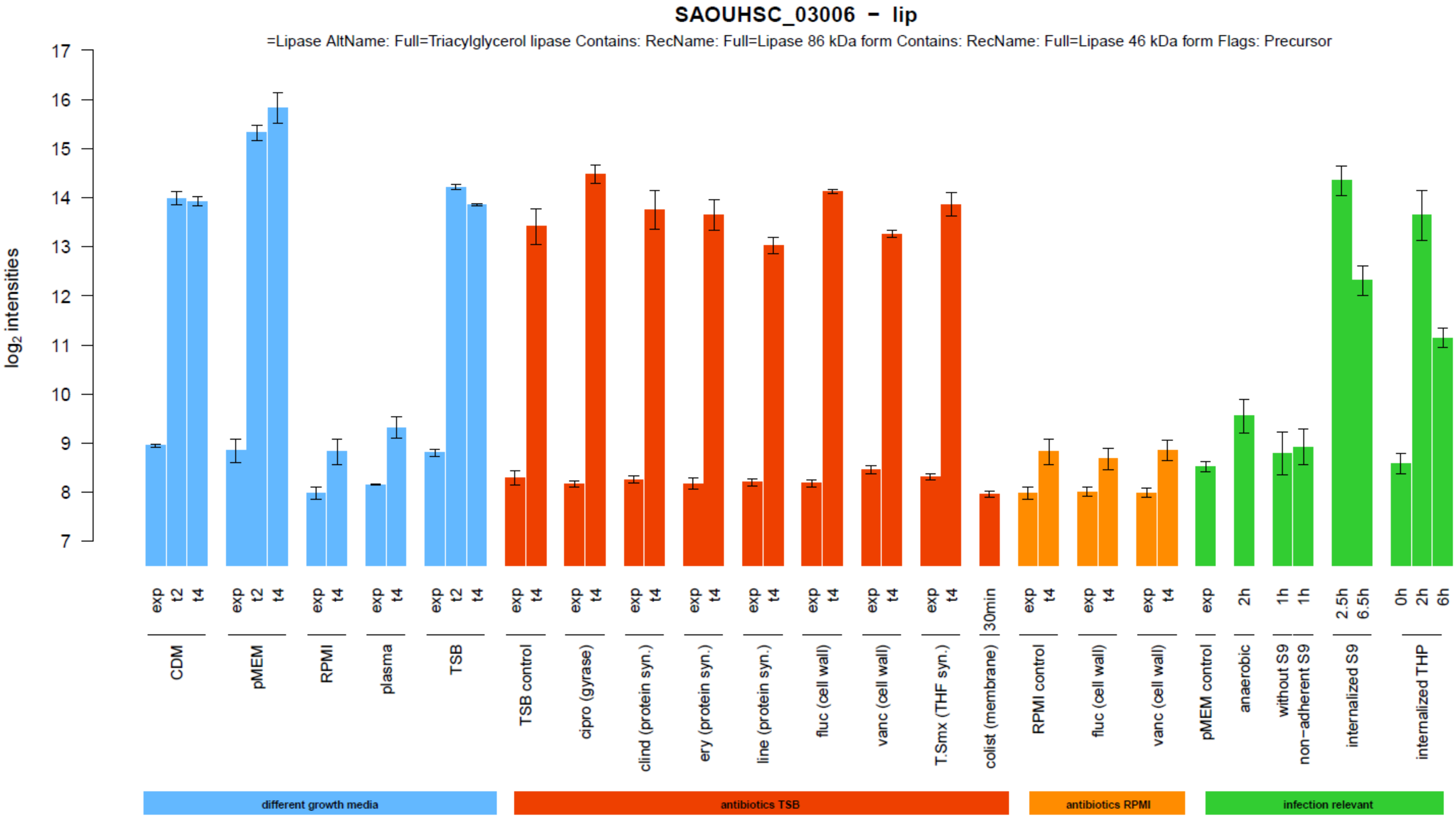
SAOUHSC_03006 – lip
=Lipase AltName: Full=Triacylglycerol lipase Contains: RecName: Full=Lipase 86 kDa form Contains: RecName: Full=Lipase 46 kDa form Flags: Precursor
log2 intensities
CDM
pMEM
RPMI
plasma
TSB
TSB control
cipro (gyrase)
clind (protein syn.)
ery (protein syn.)
line (protein syn.)
fluc (cell wall)
vanc (cell wall)
T.Smx (THF syn.)
colist (membrane)
RPMI control
fluc (cell wall)
vanc (cell wall)
pMEM control
anaerobic
without S9
non-adherent S9
internalized S9
internalized THP
different growth media
antibiotics TSB
antibiotics RPMI
infection relevant

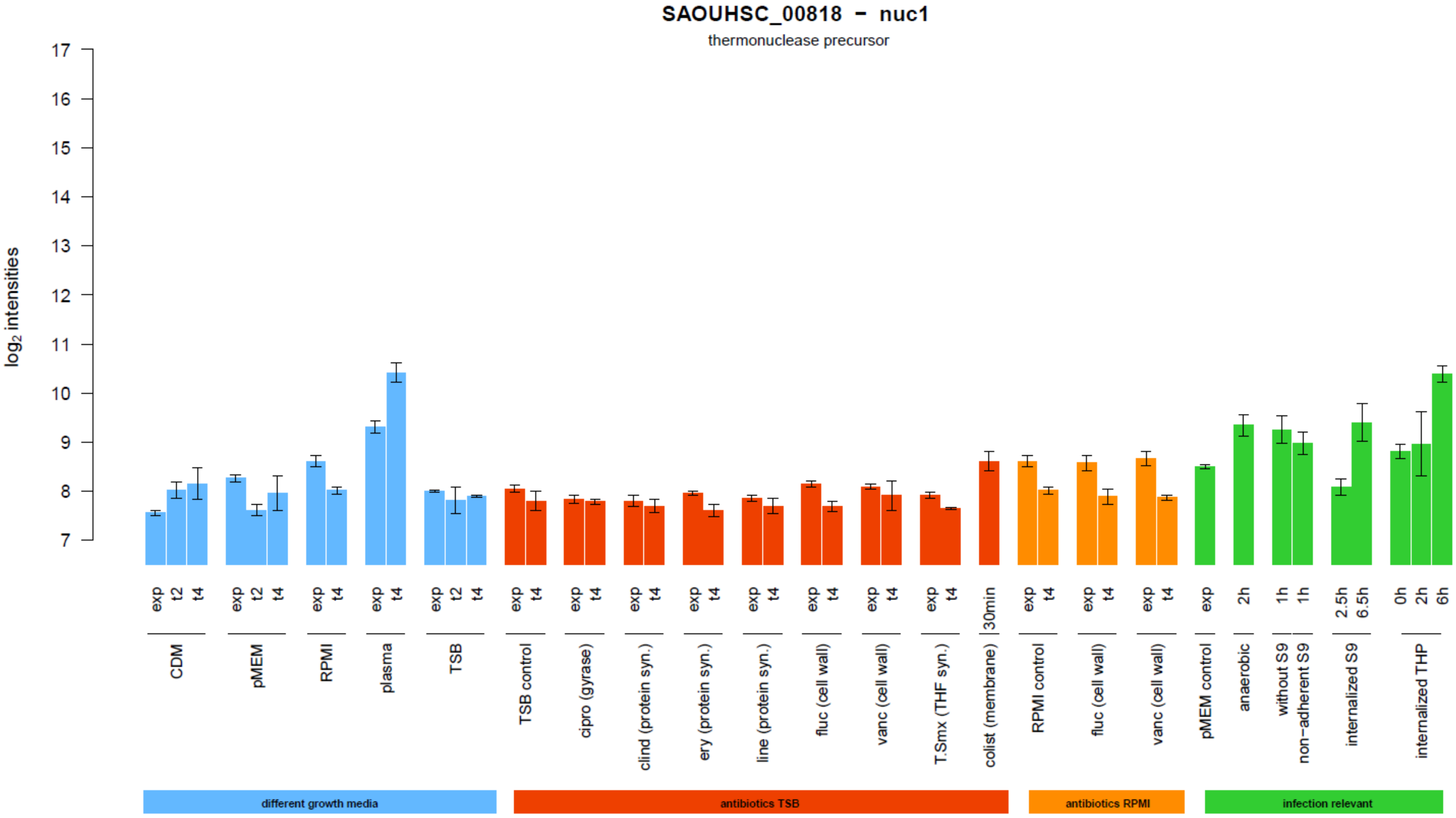

SAOUHSC_00818 – nuc1
thermonuclease precursor
log2 intensities
17
16
15
14
13
12
11
10
9
8
7
exp
t2
t4
CDM
pMEM
RPMI
plasma
TSB
TSB control
cipro (gyrase)
clind (protein syn.)
ery (protein syn.)
linе (protein syn.)
fluc (cell wall)
vanc (cell wall)
T.Smx (THF syn.)
30min
colist (membrane)
RPMI control
pMEM control
2h
anaerobic
1h
without S9
non−adherent S9
2.5h
6.5h
internalized S9
0h
6h
internalized THP
different growth media
antibiotics TSB
antibiotics RPMI
infection relevant

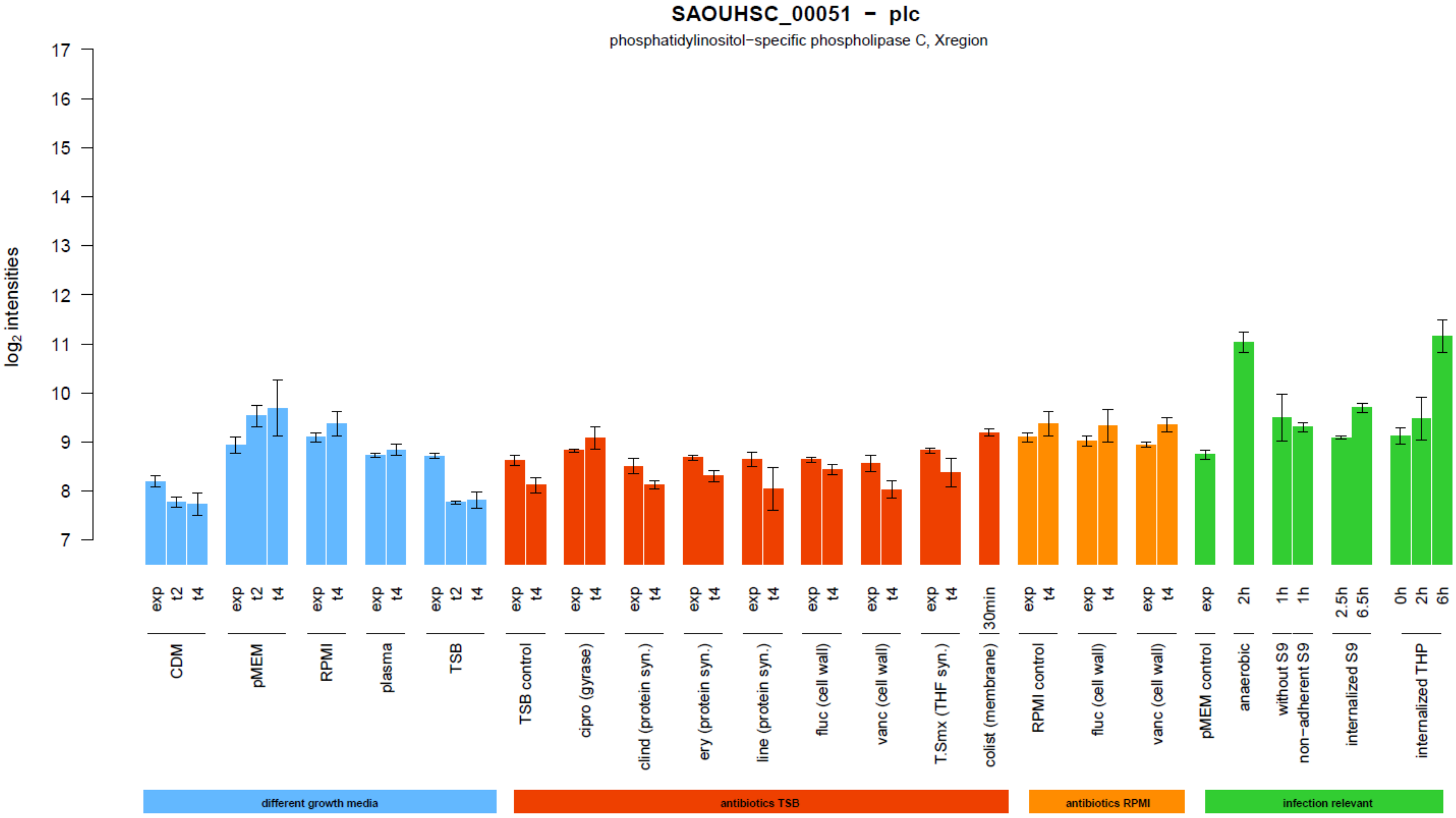
SAOUHSC_00051 – plc
phosphatidylinositol−specific phospholipase C, Xregion
log2 intensities
17
16
15
14
13
12
11
10
9
8
7
exp
t2
t4
CDM
pMEM
RPMI
plasma
TSB
TSB control
cipro (gyrase)
clind (protein syn.)
ery (protein syn.)
line (protein syn.)
fluc (cell wall)
vanc (cell wall)
T.Smx (THF syn.)
30min
colist (membrane)
RPMI control
fluc (cell wall)
vanc (cell wall)
pMEM control
2h
anaerobic
1h
without S9
non−adherent S9
2.5h
6.5h
internalized S9
0h
6h
internalized THP
different growth media
antibiotics TSB
antibiotics RPMI
infection relevant

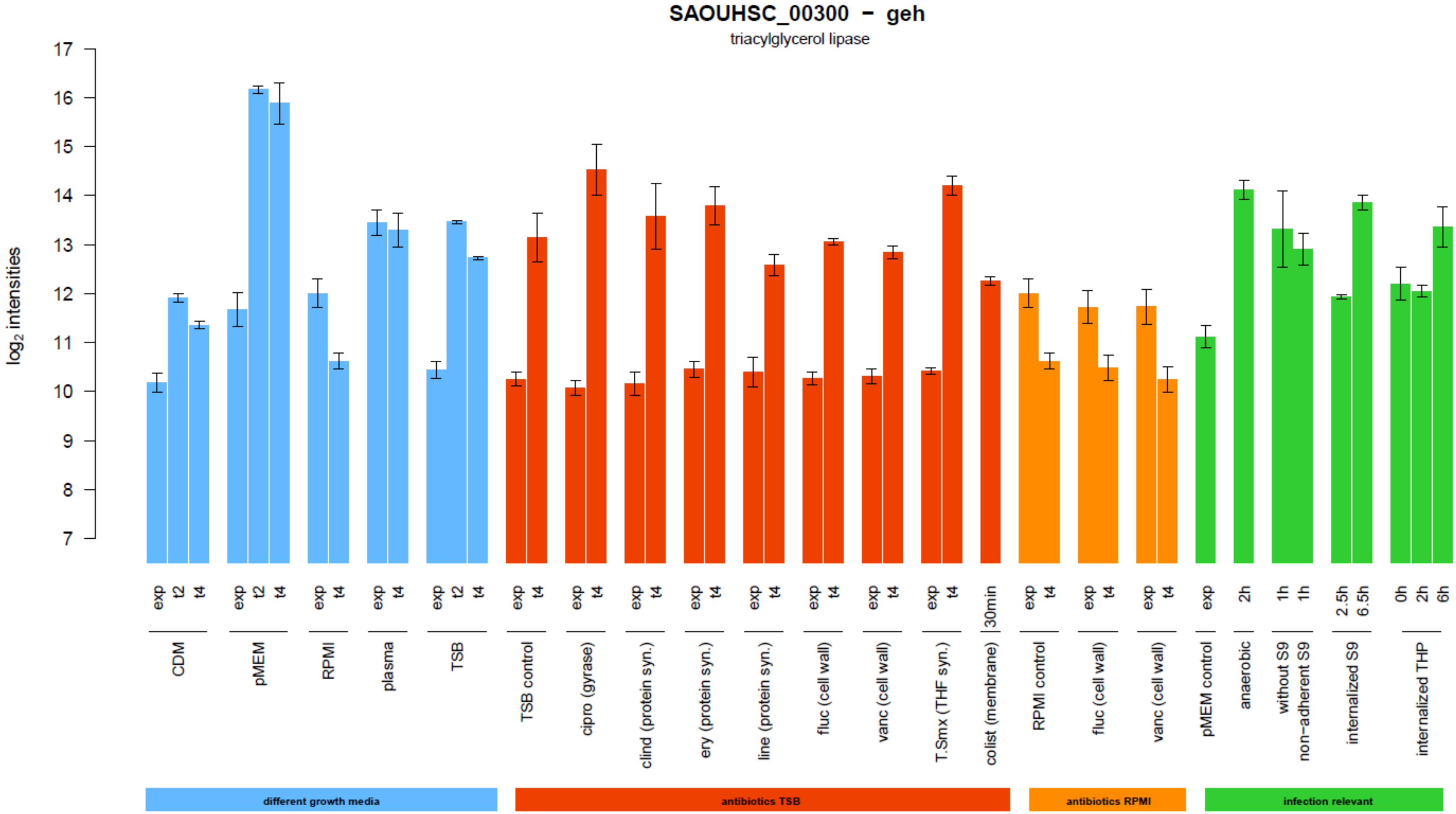

SAOUHSC_00300 – geh
triacylglycerol lipase
log2 intensities
17
16
15
14
13
12
11
10
9
8
7
exp
t2
t4
CDM
pMEM
RPMI
plasma
TSB
TSB control
cipro (gyrase)
clind (protein syn.)
ery (protein syn.)
line (protein syn.)
fluc (cell wall)
vanc (cell wall)
T.Smx (THF syn.)
30min
colist (membrane)
RPMI control
pMEM control
2h
anaerobic
1h
without S9
non−adherent S9
2.5h
6.5h
internalized S9
0h
6h
internalized THP
different growth media
antibiotics TSB
antibiotics RPMI
infection relevant

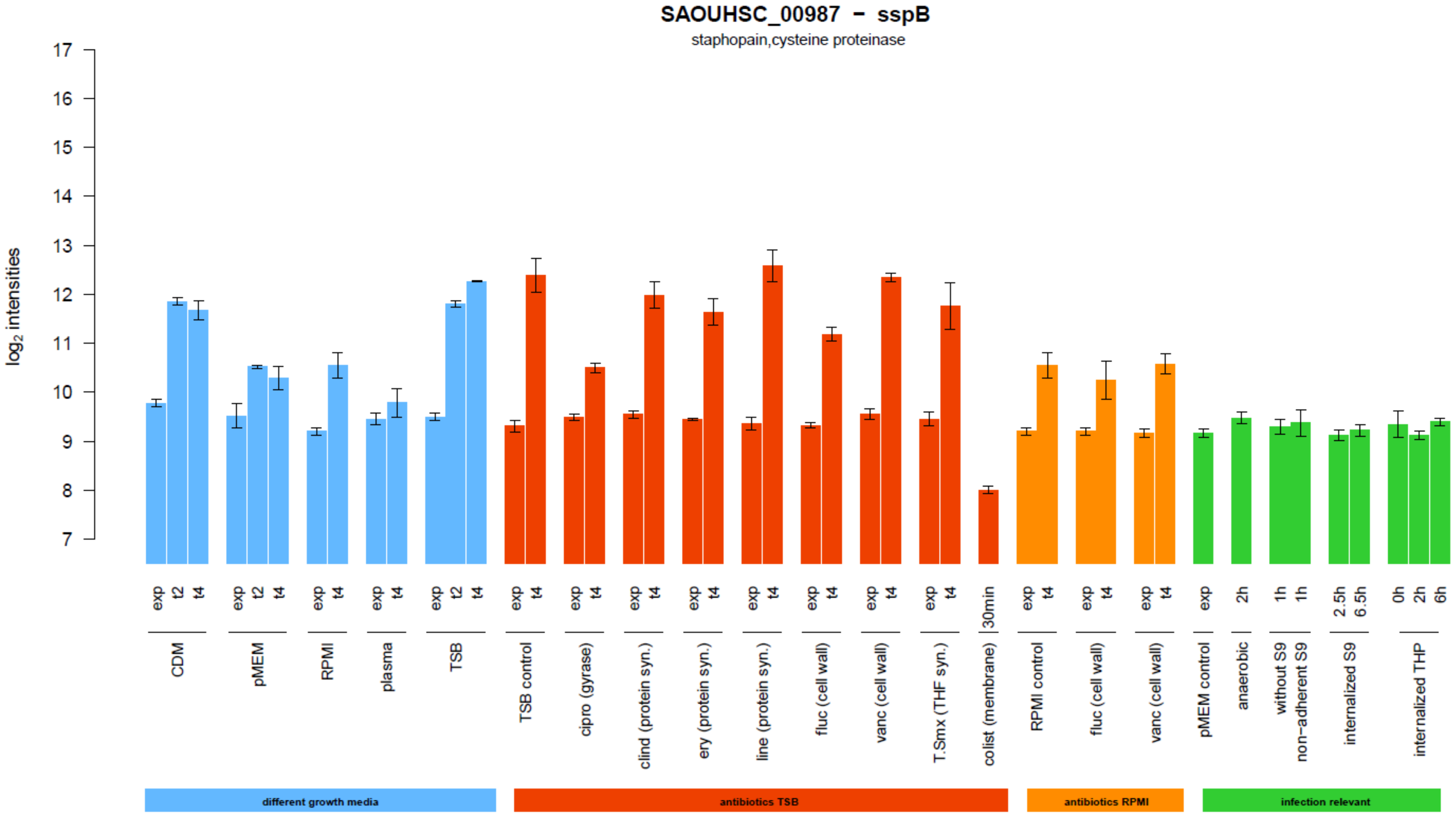
SAOUHSC_00987 – sspB
staphopain,cysteine proteinase
log2 intensities
17
16
15
14
13
12
11
10
9
8
7
exp t2 t4
CDM
exp t2 t4
pMEM
exp t4
RPMI
exp t4
plasma
exp t2 t4
TSB
exp t4
TSB control
exp t4
cipro (gyrase)
exp t4
clind (protein syn.)
exp t4
ery (protein syn.)
exp t4
line (protein syn.)
exp t4
fluc (cell wall)
exp t4
vanc (cell wall)
exp t4
T.Smx (THF syn.)
30min
colist (membrane)
exp t4
RPMI control
exp t4
fluc (cell wall)
exp t4
vanc (cell wall)
exp
pMEM control
2h
anaerobic
1h
without S9
1h
non-adherent S9
2.5h 6.5h
internalized S9
0h 2h 6h
internalized THP
different growth media
antibiotics TSB
antibiotics RPMI
infection relevant

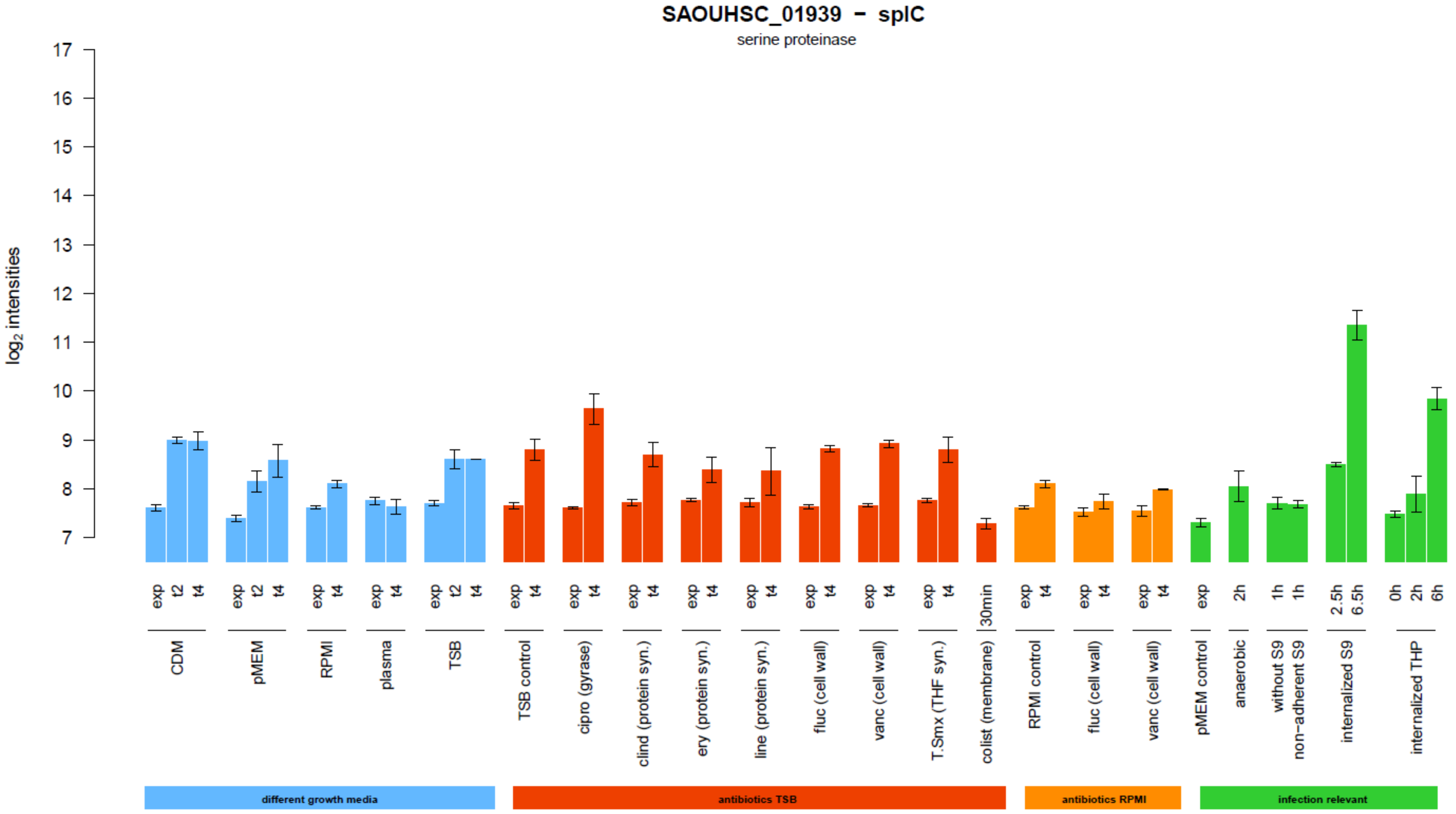
SAOUHSC_01939 – splC
serine proteinase
log2 intensities
17
16
15
14
13
12
11
10
9
8
7
exp t2 t4
CDM
exp t2 t4
pMEM
exp t4
RPMI
exp t4
plasma
exp t2 t4
TSB
exp t4
TSB control
exp t4
cipro (gyrase)
exp t4
clind (protein syn.)
exp t4
ery (protein syn.)
exp t4
line (protein syn.)
exp t4
fluc (cell wall)
exp t4
vanc (cell wall)
exp t4
T.Smx (THF syn.)
30min
colist (membrane)
exp t4
RPMI control
exp t4
fluc (cell wall)
exp t4
vanc (cell wall)
exp
pMEM control
2h
anaerobic
1h
without S9
1h
non-adherent S9
2.5h 6.5h
internalized S9
0h 2h 6h
internalized THP
different growth media
antibiotics TSB
antibiotics RPMI
infection relevant

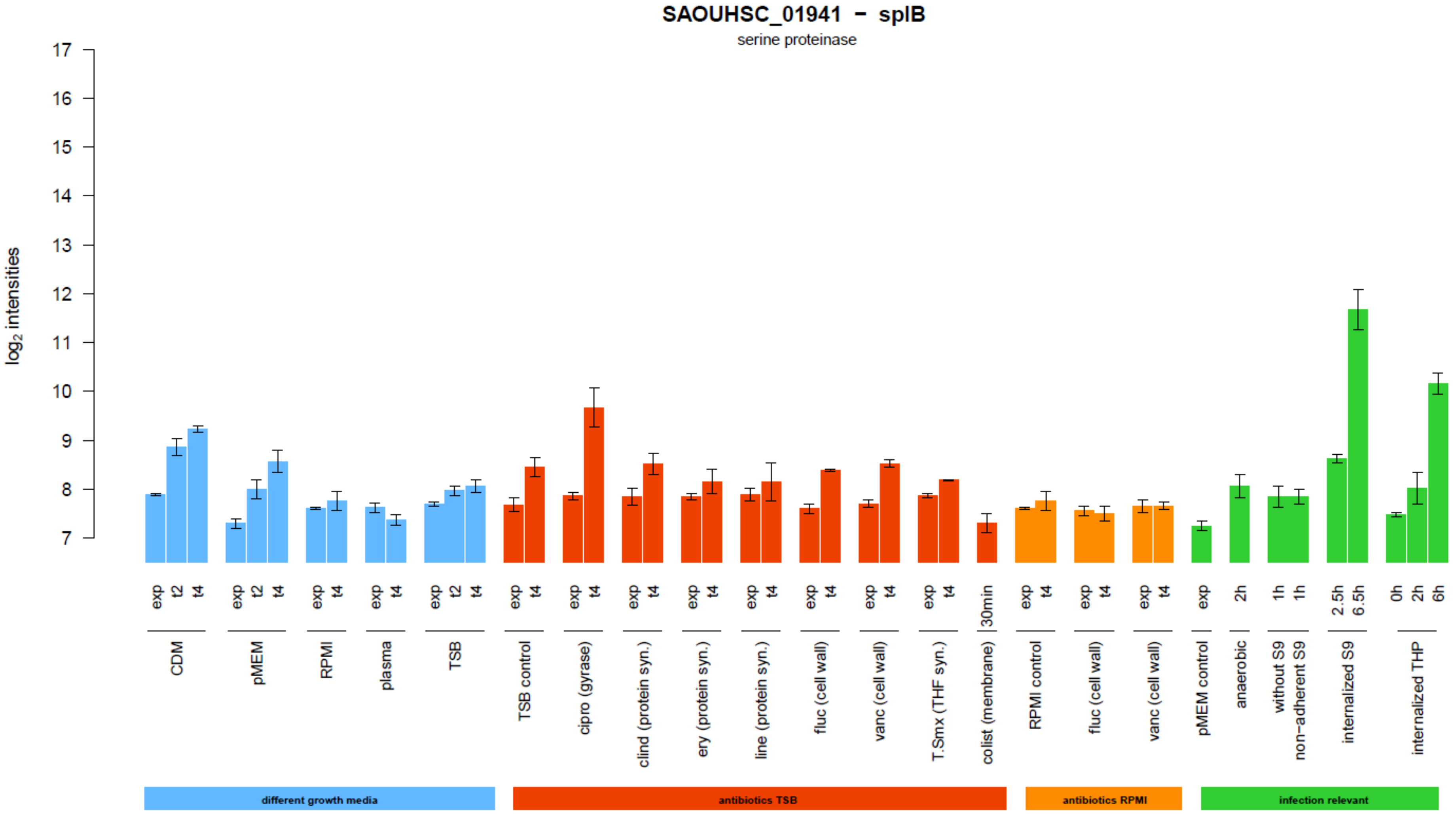
SAOUHSC_01941 – splB
serine proteinase
log2 intensities
17
16
15
14
13
12
11
10
9
8
7
exp t2 t4
CDM
exp t2 t4
pMEM
exp t4
RPMI
exp t4
plasma
exp t2 t4
TSB
exp t4
TSB control
exp t4
cipro (gyrase)
exp t4
clind (protein syn.)
exp t4
ery (protein syn.)
exp t4
line (protein syn.)
exp t4
fluc (cell wall)
exp t4
vanc (cell wall)
exp t4
T.Smx (THF syn.)
30min
colist (membrane)
exp t4
RPMI control
exp t4
fluc (cell wall)
exp t4
vanc (cell wall)
exp
pMEM control
2h
anaerobic
1h
without S9
1h
non-adherent S9
2.5h 6.5h
internalized S9
0h 2h 6h
internalized THP
different growth media
antibiotics TSB
antibiotics RPMI
infection relevant

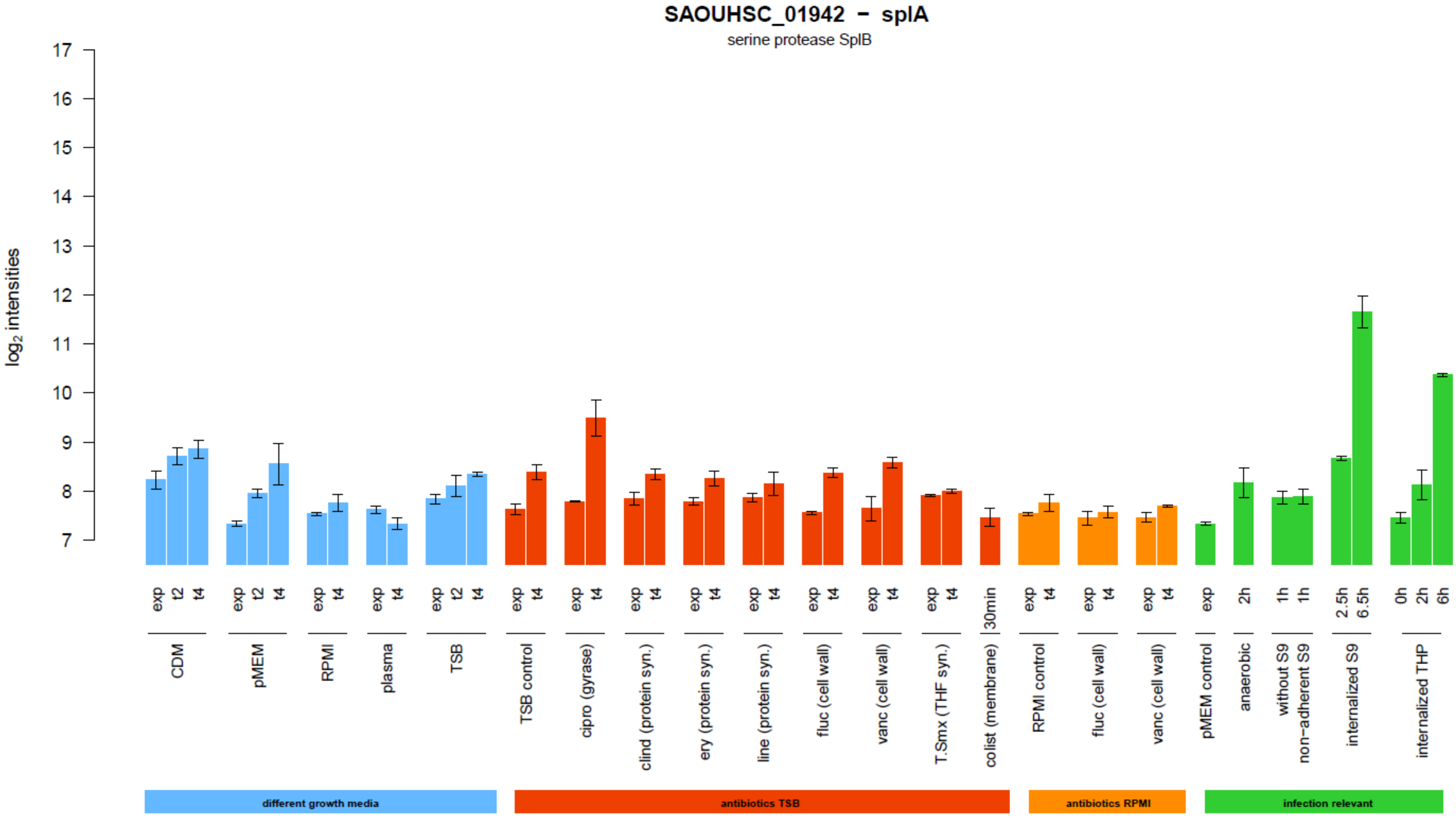

SAOUHSC_01942 – splA
serine protease SplB
log2 intensities
17
16
15
14
13
12
11
10
9
8
7
exp t2 t4
CDM
exp t2 t4
pMEM
exp t4
RPMI
exp t4
plasma
exp t2 t4
TSB
exp t4
TSB control
exp t4
cipro (gyrase)
exp t4
clind (protein syn.)
exp t4
ery (protein syn.)
exp t4
line (protein syn.)
exp t4
fluc (cell wall)
exp t4
vanc (cell wall)
exp t4
T.Smx (THF syn.)
30min
colist (membrane)
exp t4
RPMI control
exp t4
fluc (cell wall)
exp t4
vanc (cell wall)
exp
pMEM control
2h
anaerobic
1h
without S9
1h
non-adherent S9
2.5h 6.5h
internalized S9
0h 2h 6h
internalized THP
different growth media
antibiotics TSB
antibiotics RPMI
infection relevant

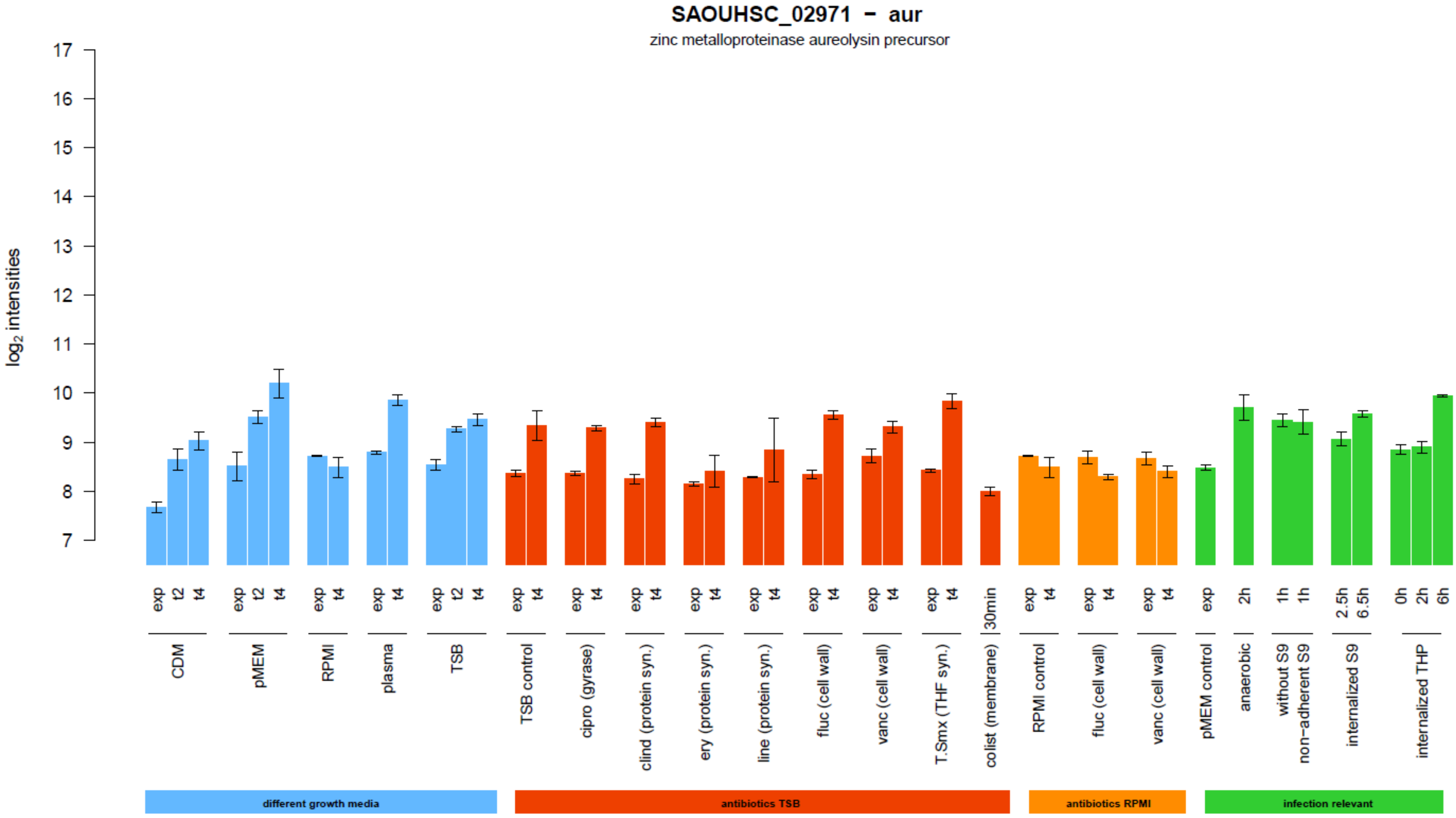
SAOUHSC_02971 – aur
zinc metalloproteinase aureolysin precursor
log2 intensities
17
16
15
14
13
12
11
10
9
8
7
exp t2 t4
CDM
exp t2 t4
pMEM
exp t4
RPMI
exp t4
plasma
exp t2 t4
TSB
exp t4
TSB control
exp t4
cipro (gyrase)
exp t4
clind (protein syn.)
exp t4
ery (protein syn.)
exp t4
line (protein syn.)
exp t4
fluc (cell wall)
exp t4
vanc (cell wall)
exp t4
T.Smx (THF syn.)
30min
colist (membrane)
exp t4
RPMI control
exp t4
fluc (cell wall)
exp t4
vanc (cell wall)
exp
pMEM control
2h
anaerobic
1h
without S9
1h
non−adherent S9
2.5h 6.5h
internalized S9
0h 2h 6h
internalized THP
different growth media
antibiotics TSB
antibiotics RPMI
infection relevant

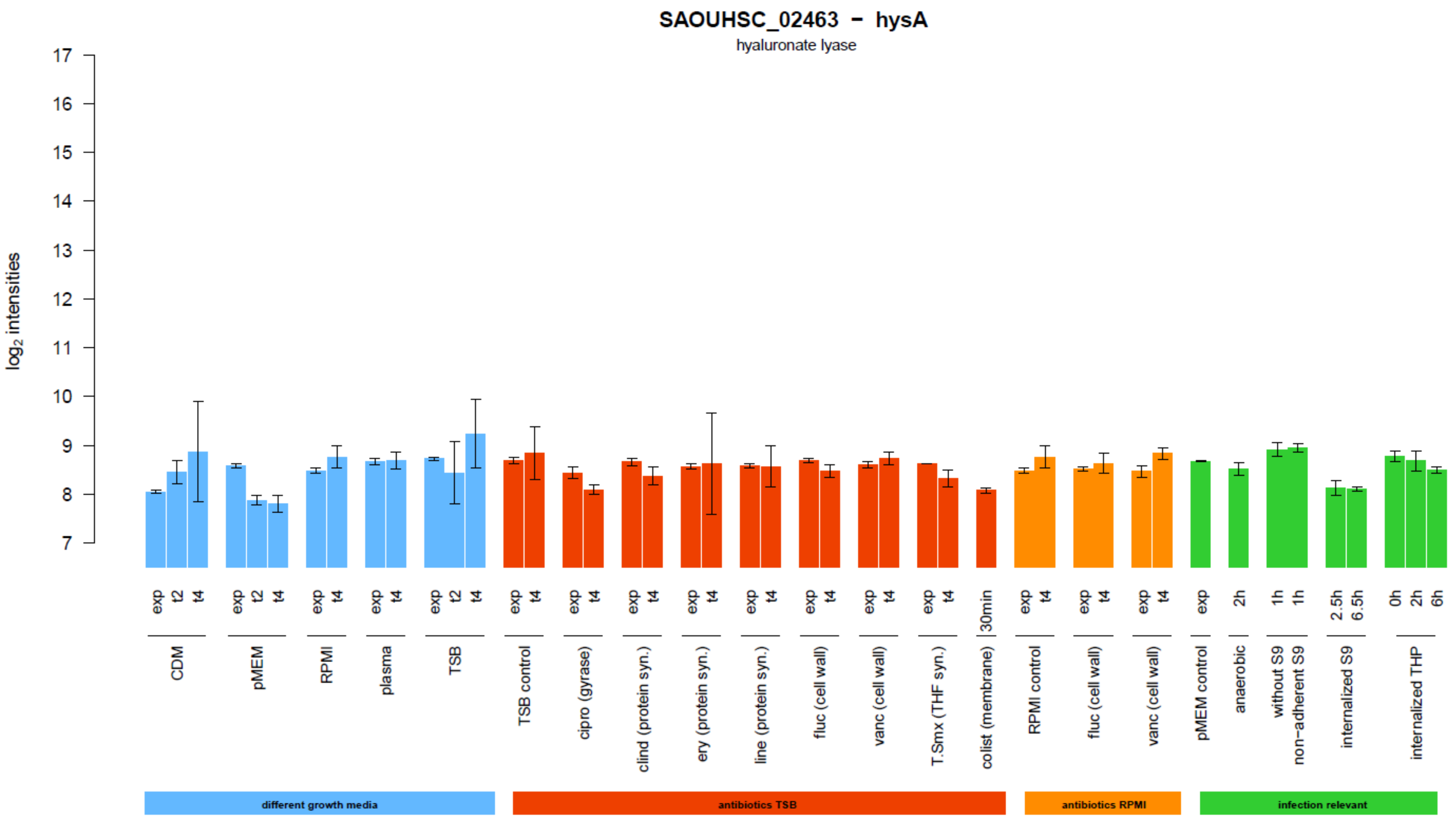

SAOUHSC_02463 – hysA
hyaluronate lyase
log2 intensities
17
16
15
14
13
12
11
10
9
8
7
exp t2 t4
CDM
exp t2 t4
pMEM
exp t4
RPMI
exp t4
plasma
exp t2 t4
TSB
exp t4
TSB control
exp t4
cipro (gyrase)
exp t4
clind (protein syn.)
exp t4
ery (protein syn.)
exp t4
line (protein syn.)
exp t4
fluc (cell wall)
exp t4
vanc (cell wall)
exp t4
T.Smx (THF syn.)
30min
colist (membrane)
exp t4
RPMI control
exp t4
fluc (cell wall)
exp t4
vanc (cell wall)
exp
pMEM control
2h
anaerobic
1h
without S9
1h
non−adherent S9
2.5h 6.5h
internalized S9
0h 2h 6h
internalized THP
different growth media
antibiotics TSB
antibiotics RPMI
infection relevant

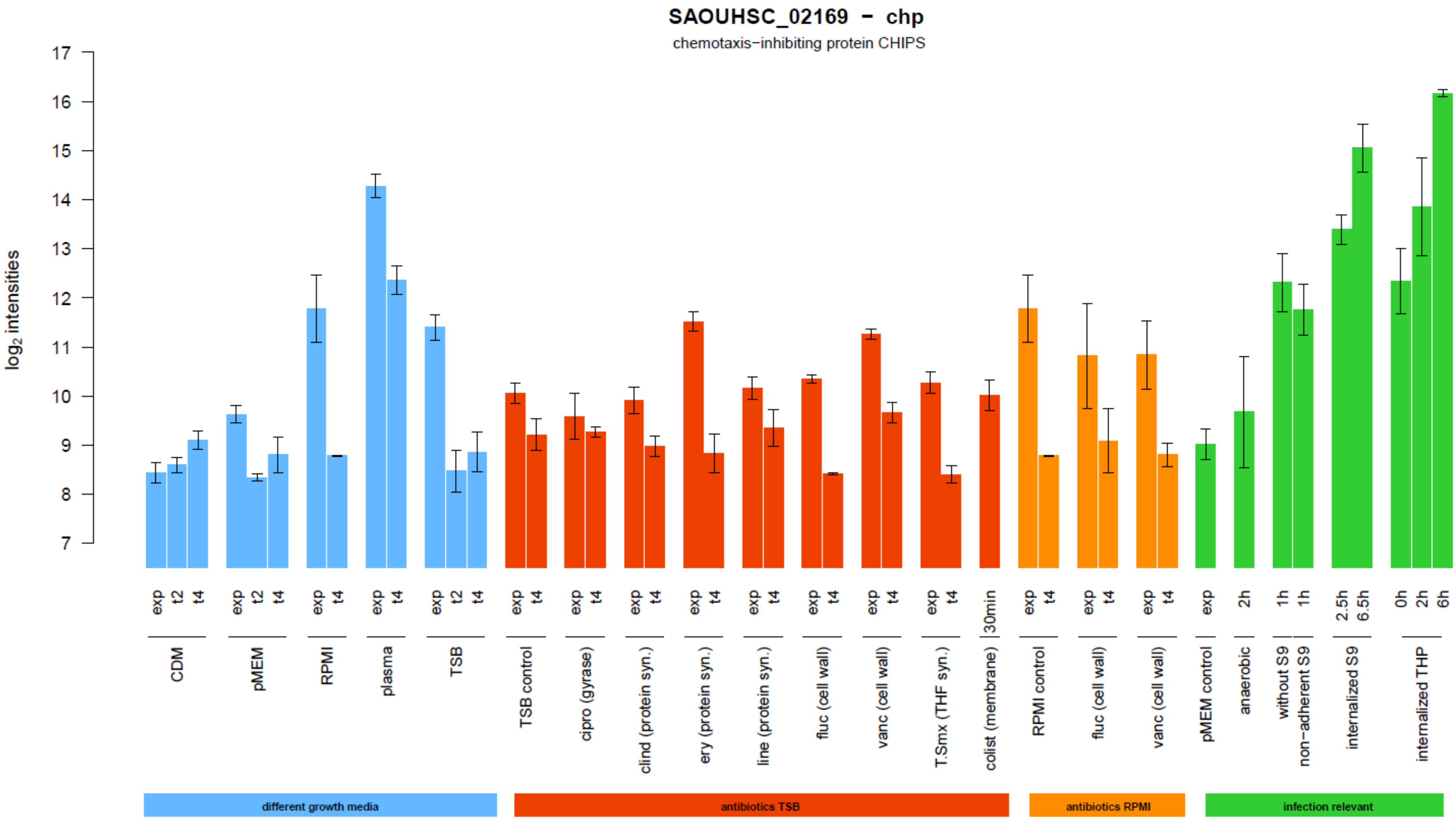
SAOUHSC_02169 – chp
chemotaxis–inhibiting protein CHIPS
log2 intensities
17
16
15
14
13
12
11
10
9
8
7
exp t2 t4
CDM
exp t2 t4
pMEM
exp t4
RPMI
exp t4
plasma
exp t2 t4
TSB
exp t4
TSB control
exp t4
cipro (gyrase)
exp t4
clind (protein syn.)
exp t4
ery (protein syn.)
exp t4
line (protein syn.)
exp t4
fluc (cell wall)
exp t4
vanc (cell wall)
exp t4
T.Smx (THF syn.)
30min
colist (membrane)
exp t4
RPMI control
exp t4
fluc (cell wall)
exp t4
vanc (cell wall)
exp
pMEM control
2h
anaerobic
1h
without S9
1h
non–adherent S9
2.5h 6.5h
internalized S9
0h 2h 6h
internalized THP
different growth media
antibiotics TSB
antibiotics RPMI
infection relevant

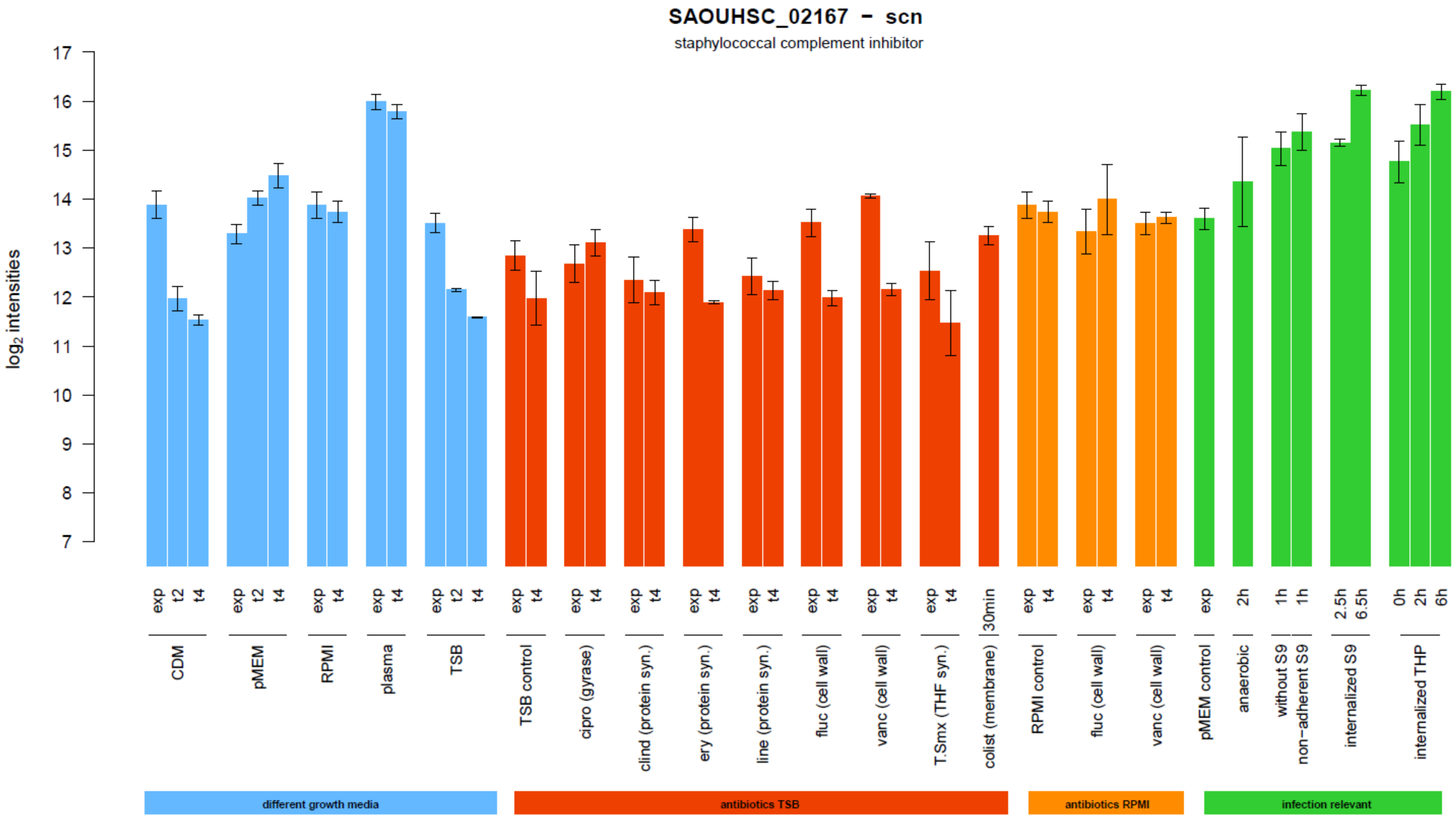
SAOUHSC_02167 – scn
staphylococcal complement inhibitor
log2 intensities
17
16
15
14
13
12
11
10
9
8
7
exp t2 t4
CDM
exp t2 t4
pMEM
exp t4
RPMI
exp t4
plasma
exp t2 t4
TSB
exp t4
TSB control
exp t4
cipro (gyrase)
exp t4
clind (protein syn.)
exp t4
ery (protein syn.)
exp t4
line (protein syn.)
exp t4
fluc (cell wall)
exp t4
vanc (cell wall)
exp t4
T.Smx (THF syn.)
30min
colist (membrane)
exp t4
RPMI control
exp t4
fluc (cell wall)
exp t4
vanc (cell wall)
exp
pMEM control
2h
anaerobic
1h
without S9
1h
non−adherent S9
2.5h 6.5h
internalized S9
0h 2h 6h
internalized THP
different growth media
antibiotics TSB
antibiotics RPMI
infection relevant

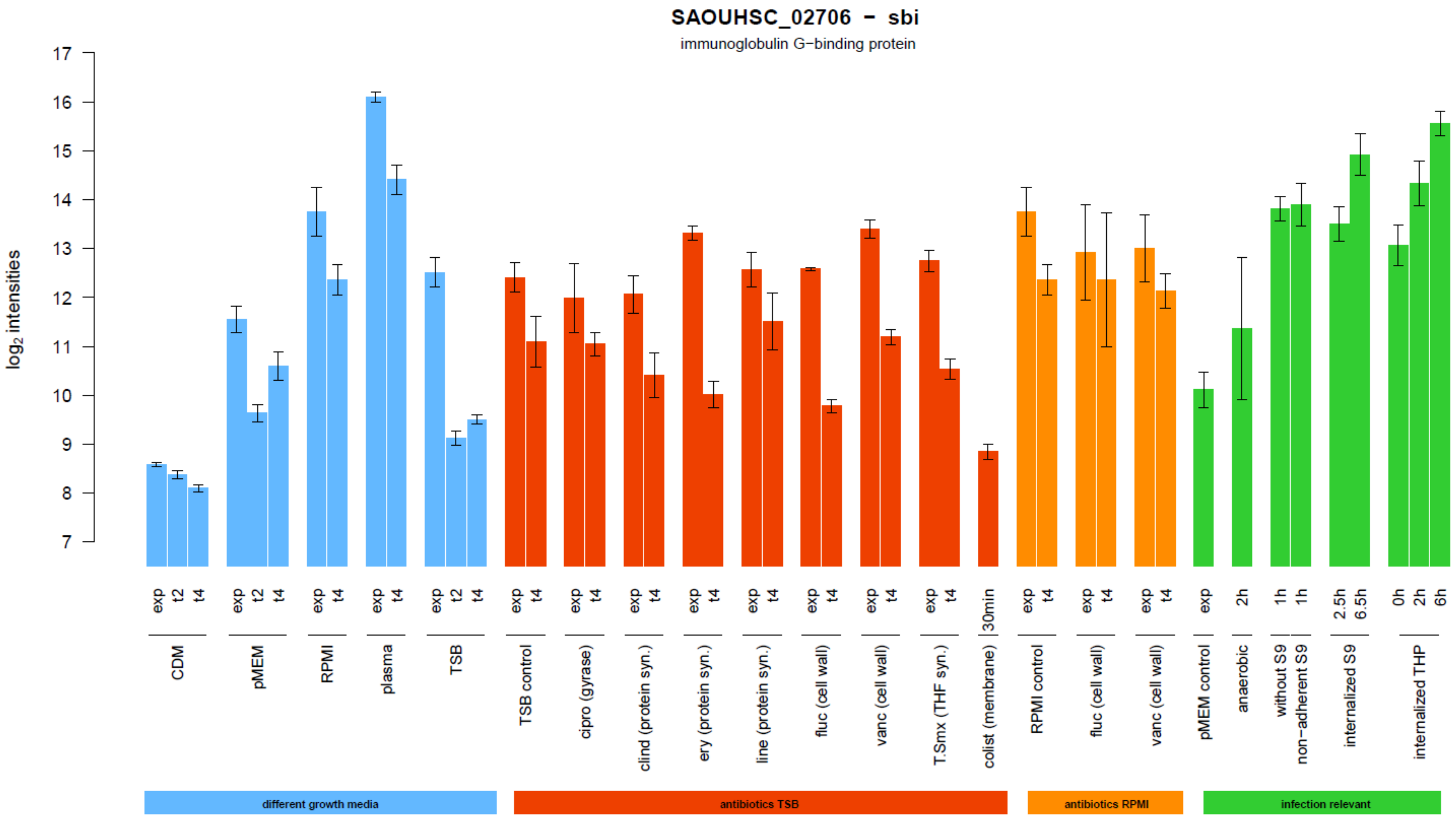

SAOUHSC_02706 – sbi
immunoglobulin G–binding protein
log2 intensities
17
16
15
14
13
12
11
10
9
8
7
exp t2 t4
CDM
exp t2 t4
pMEM
exp t4
RPMI
exp t4
plasma
exp t2 t4
TSB
exp t4
TSB control
exp t4
cipro (gyrase)
exp t4
clind (protein syn.)
exp t4
ery (protein syn.)
exp t4
line (protein syn.)
exp t4
fluc (cell wall)
exp t4
vanc (cell wall)
exp t4
T.Smx (THF syn.)
30min
colist (membrane)
exp t4
RPMI control
exp t4
fluc (cell wall)
exp t4
vanc (cell wall)
exp
pMEM control
2h
anaerobic
1h
without S9
1h
non–adherent S9
2.5h 6.5h
internalized S9
0h 2h 6h
internalized THP
different growth media
antibiotics TSB
antibiotics RPMI
infection relevant

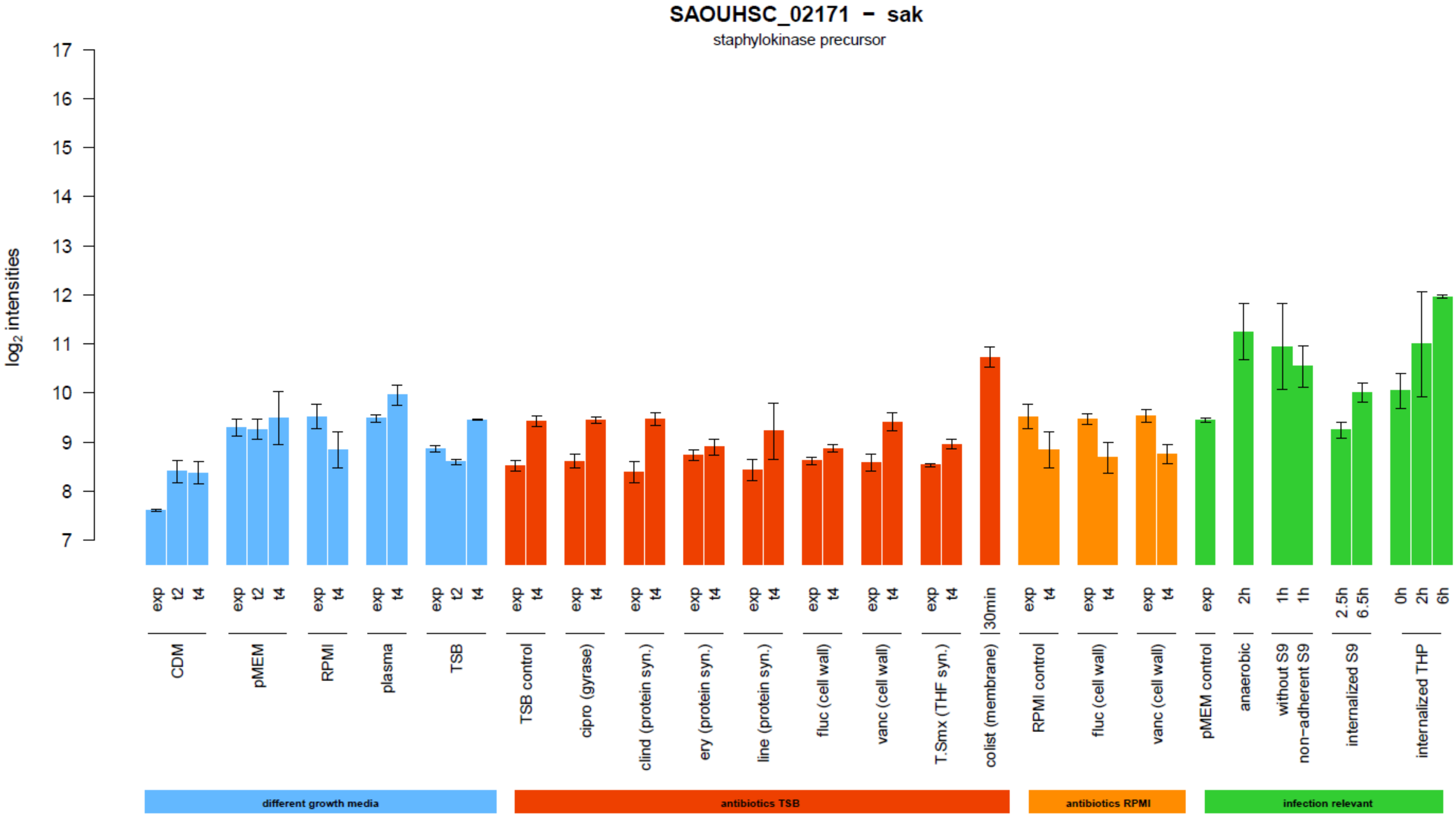

SAOUHSC_02171 – sak
staphylokinase precursor
log2 intensities
17
16
15
14
13
12
11
10
9
8
7
exp t2 t4
CDM
exp t2 t4
pMEM
exp t4
RPMI
exp t4
plasma
exp t2 t4
TSB
exp t4
TSB control
exp t4
cipro (gyrase)
exp t4
clind (protein syn.)
exp t4
ery (protein syn.)
exp t4
line (protein syn.)
exp t4
fluc (cell wall)
exp t4
vanc (cell wall)
exp t4
T.Smx (THF syn.)
30min
colist (membrane)
exp t4
RPMI control
exp t4
fluc (cell wall)
exp t4
vanc (cell wall)
exp
pMEM control
2h
anaerobic
1h
without S9
1h
non-adherent S9
2.5h 6.5h
internalized S9
0h 2h 6h
internalized THP
different growth media
antibiotics TSB
antibiotics RPMI
infection relevant

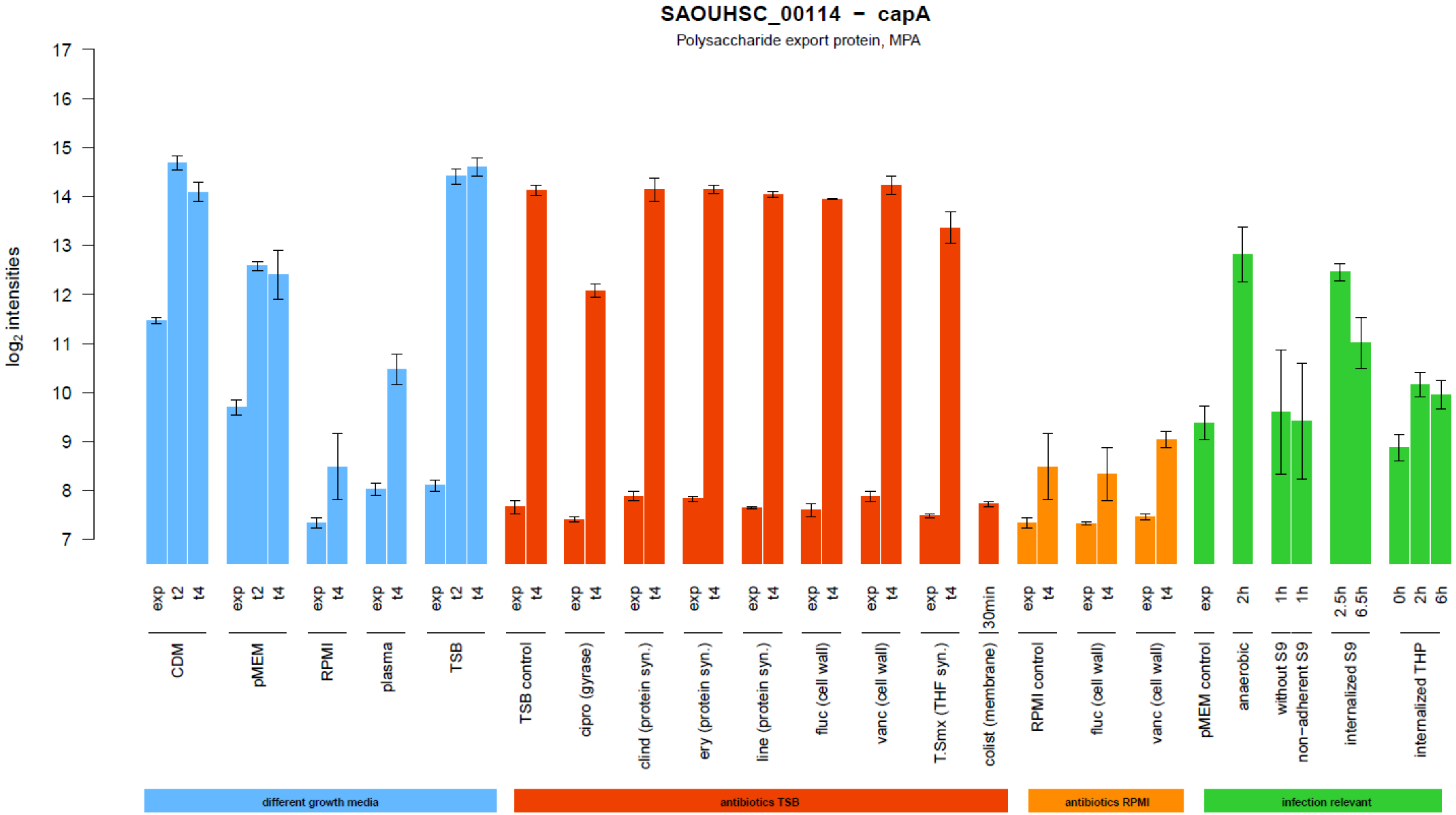
SAOUHSC_00114 – capA
Polysaccharide export protein, MPA
log2 intensities
17
16
15
14
13
12
11
10
9
8
7
exp
t2
t4
CDM
pMEM
RPMI
plasma
TSB
TSB control
cipro (gyrase)
clind (protein syn.)
ery (protein syn.)
line (protein syn.)
fluc (cell wall)
vanc (cell wall)
T.Smx (THF syn.)
colist (membrane)
30min
RPMI control
pMEM control
anaerobic
2h
without S9
non-adherent S9
1h
internalized S9
2.5h
6.5h
internalized THP
0h
6h
different growth media
antibiotics TSB
antibiotics RPMI
infection relevant

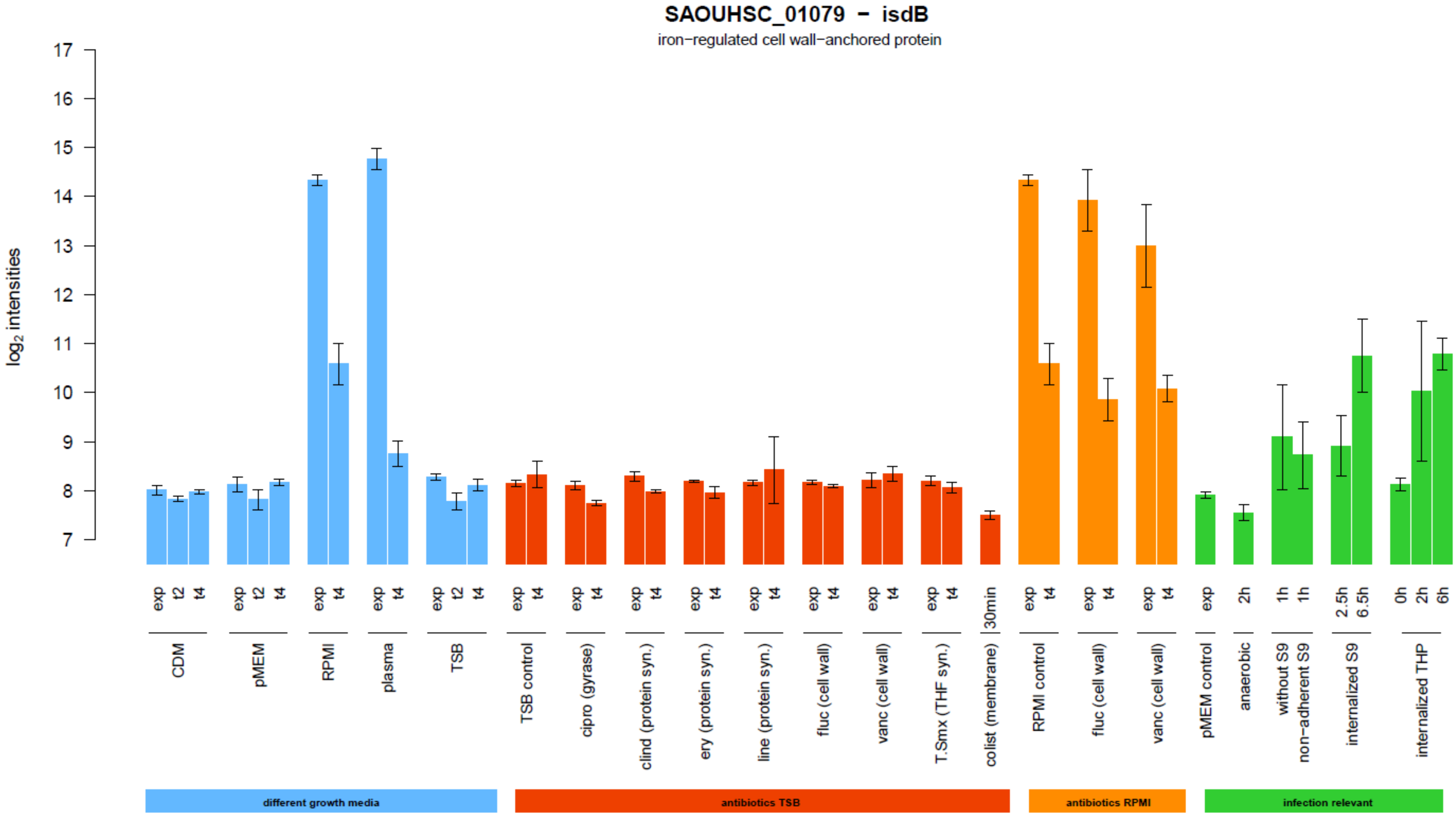
SAOUHSC_01079 – isdB
iron−regulated cell wall−anchored protein
log2 intensities
17
16
15
14
13
12
11
10
9
8
7
exp t2 t4
CDM
exp t2 t4
pMEM
exp t4
RPMI
exp t4
plasma
exp t2 t4
TSB
exp t4
TSB control
exp t4
cipro (gyrase)
exp t4
clind (protein syn.)
exp t4
ery (protein syn.)
exp t4
line (protein syn.)
exp t4
fluc (cell wall)
exp t4
vanc (cell wall)
exp t4
T.Smx (THF syn.)
30min
colist (membrane)
exp t4
RPMI control
exp t4
fluc (cell wall)
exp t4
vanc (cell wall)
exp
pMEM control
2h
anaerobic
1h
without S9
1h
non−adherent S9
2.5h 6.5h
internalized S9
0h 2h 6h
internalized THP
different growth media
antibiotics TSB
antibiotics RPMI
infection relevant

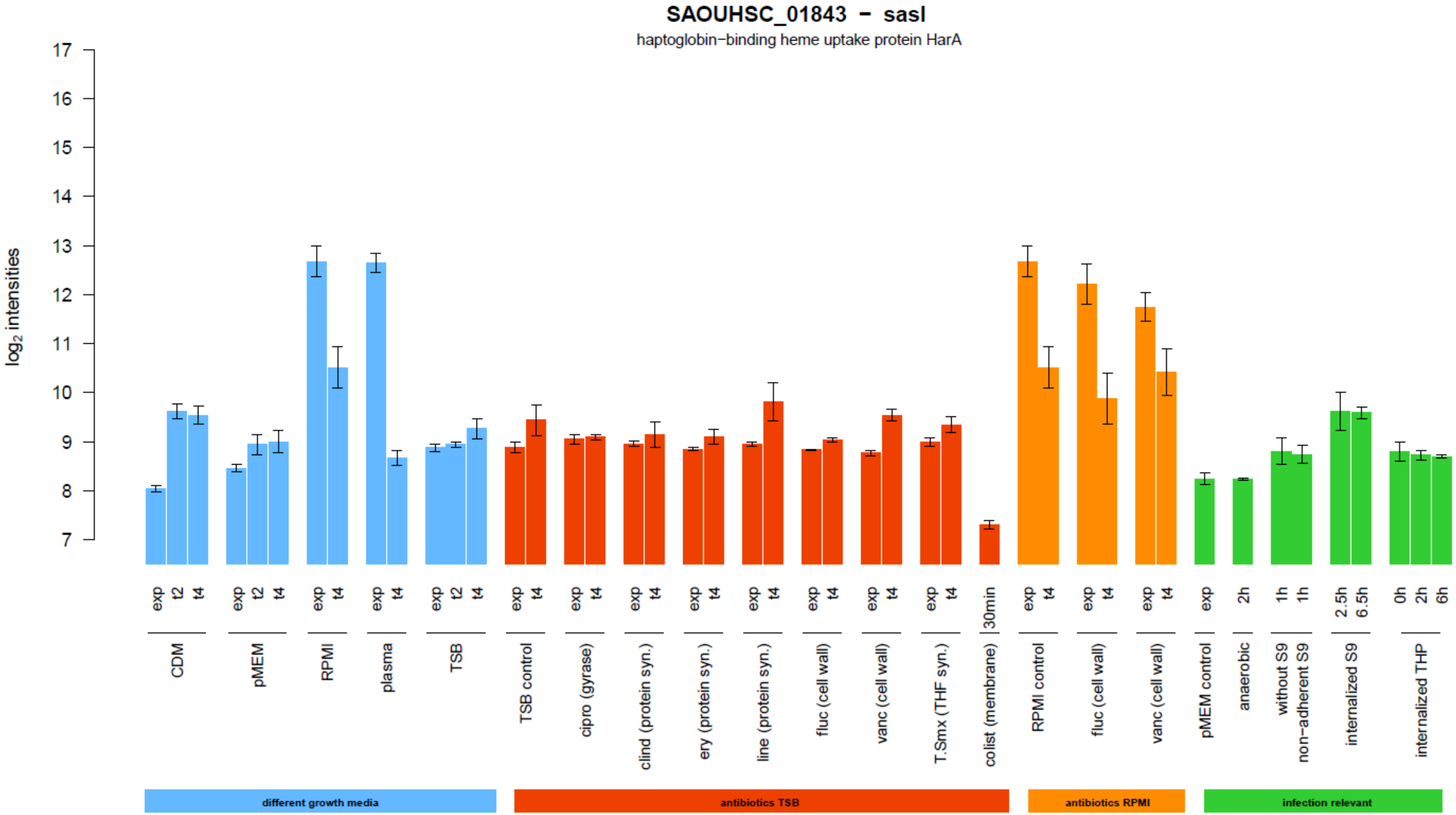
SAOUHSC_01843 - sasI
haptoglobin−binding heme uptake protein HarA
log2 intensities
17
16
15
14
13
12
11
10
9
8
7
exp t2 t4
CDM
exp t2 t4
pMEM
exp t4
RPMI
exp t4
plasma
exp t2 t4
TSB
exp t4
TSB control
exp t4
cipro (gyrase)
exp t4
clind (protein syn.)
exp t4
ery (protein syn.)
exp t4
line (protein syn.)
exp t4
fluc (cell wall)
exp t4
vanc (cell wall)
exp t4
T.Smx (THF syn.)
30min
colist (membrane)
exp t4
RPMI control
exp t4
fluc (cell wall)
exp t4
vanc (cell wall)
exp
pMEM control
2h
anaerobic
1h
without S9
1h
non−adherent S9
2.5h 6.5h
internalized S9
0h 2h 6h
internalized THP
different growth media
antibiotics TSB
antibiotics RPMI
infection relevant

reference genes

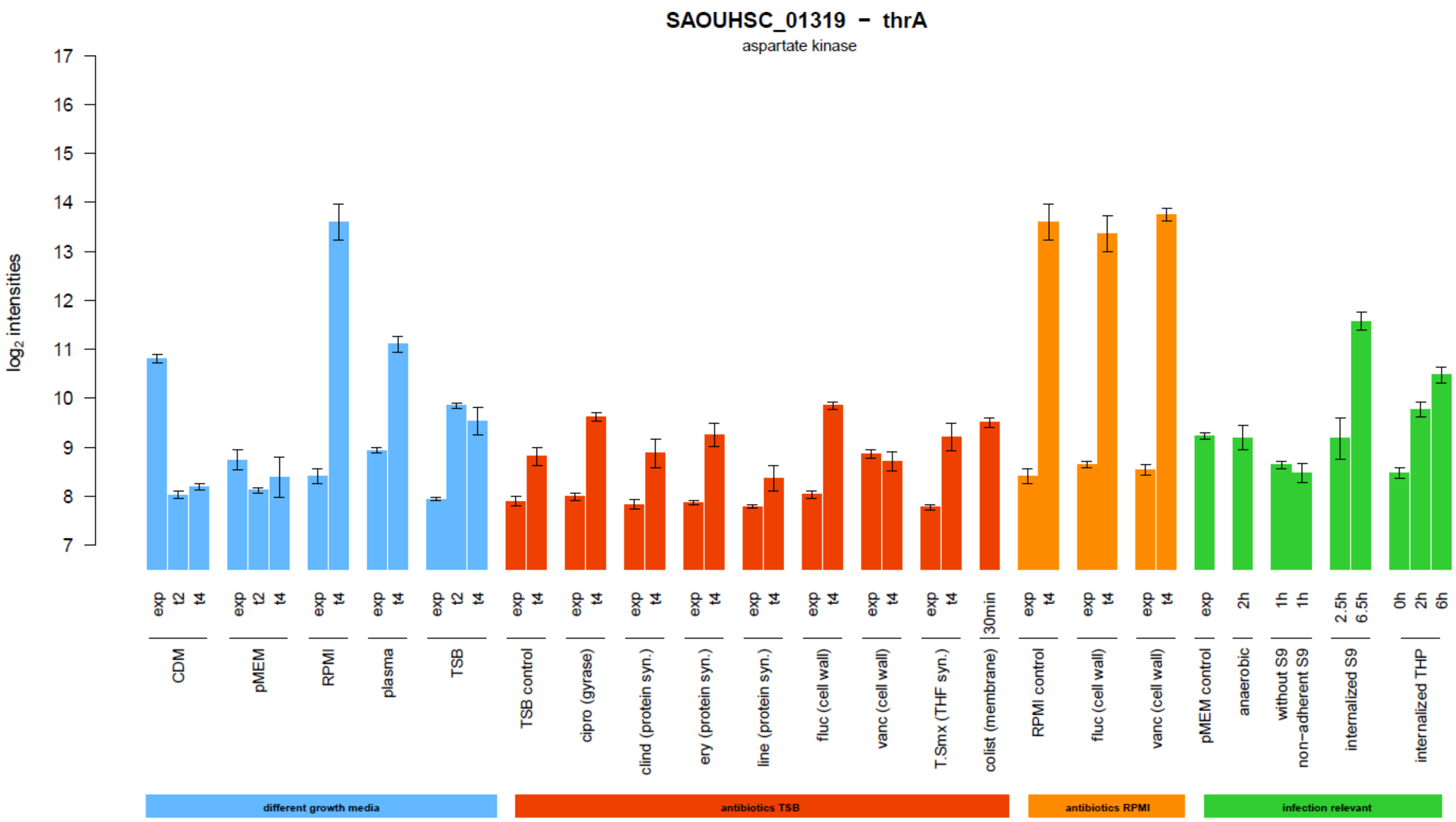

SAOUHSC_01319 – thrA
aspartate kinase
log2 intensities
17
16
15
14
13
12
11
10
9
8
7
exp t2 t4
CDM
exp t2 t4
pMEM
exp t4
RPMI
exp t4
plasma
exp t2 t4
TSB
exp t4
TSB control
exp t4
cipro (gyrase)
exp t4
clind (protein syn.)
exp t4
ery (protein syn.)
exp t4
line (protein syn.)
exp t4
fluc (cell wall)
exp t4
vanc (cell wall)
exp t4
T.Smx (THF syn.)
30min
colist (membrane)
exp t4
RPMI control
exp t4
fluc (cell wall)
exp t4
vanc (cell wall)
exp
pMEM control
2h
anaerobic
1h 1h
without S9
non−adherent S9
2.5h 6.5h
internalized S9
0h 2h 6h
internalized THP
different growth media
antibiotics TSB
antibiotics RPMI
infection relevant

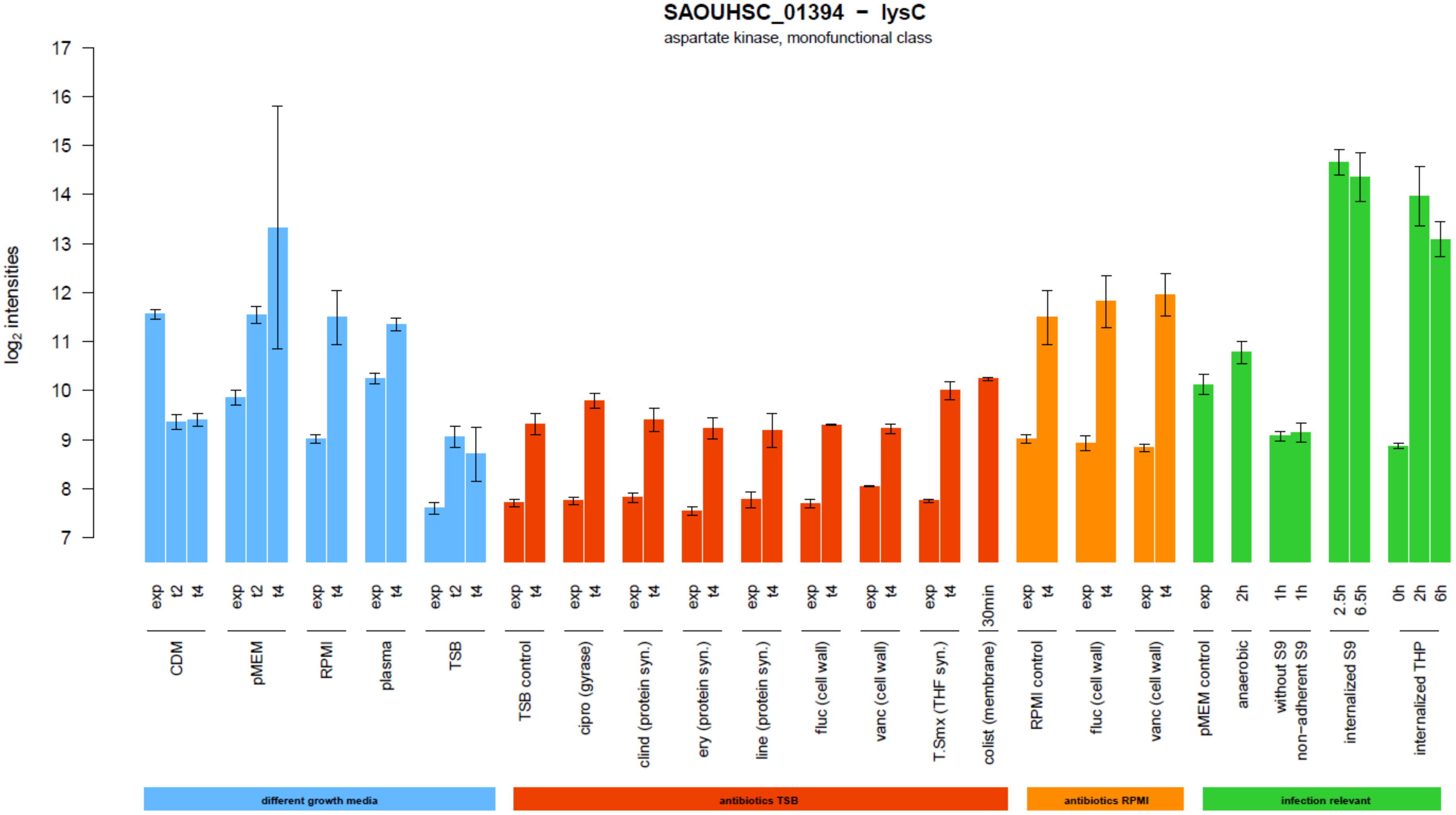
SAOUHSC_01394 – lysC
aspartate kinase, monofunctional class
log2 intensities
17
16
15
14
13
12
11
10
9
8
7
exp t2 t4
CDM
exp t2 t4
pMEM
exp t4
RPMI
exp t4
plasma
exp t2 t4
TSB
exp t4
TSB control
exp t4
cipro (gyrase)
exp t4
clind (protein syn.)
exp t4
ery (protein syn.)
exp t4
line (protein syn.)
exp t4
fluc (cell wall)
exp t4
vanc (cell wall)
exp t4
T.Smx (THF syn.)
30min
colist (membrane)
exp t4
RPMI control
exp t4
fluc (cell wall)
exp t4
vanc (cell wall)
exp
pMEM control
2h
anaerobic
1h
without S9
1h
non−adherent S9
2.5h 6.5h
internalized S9
0h 2h 6h
internalized THP
different growth media
antibiotics TSB
antibiotics RPMI
infection relevant

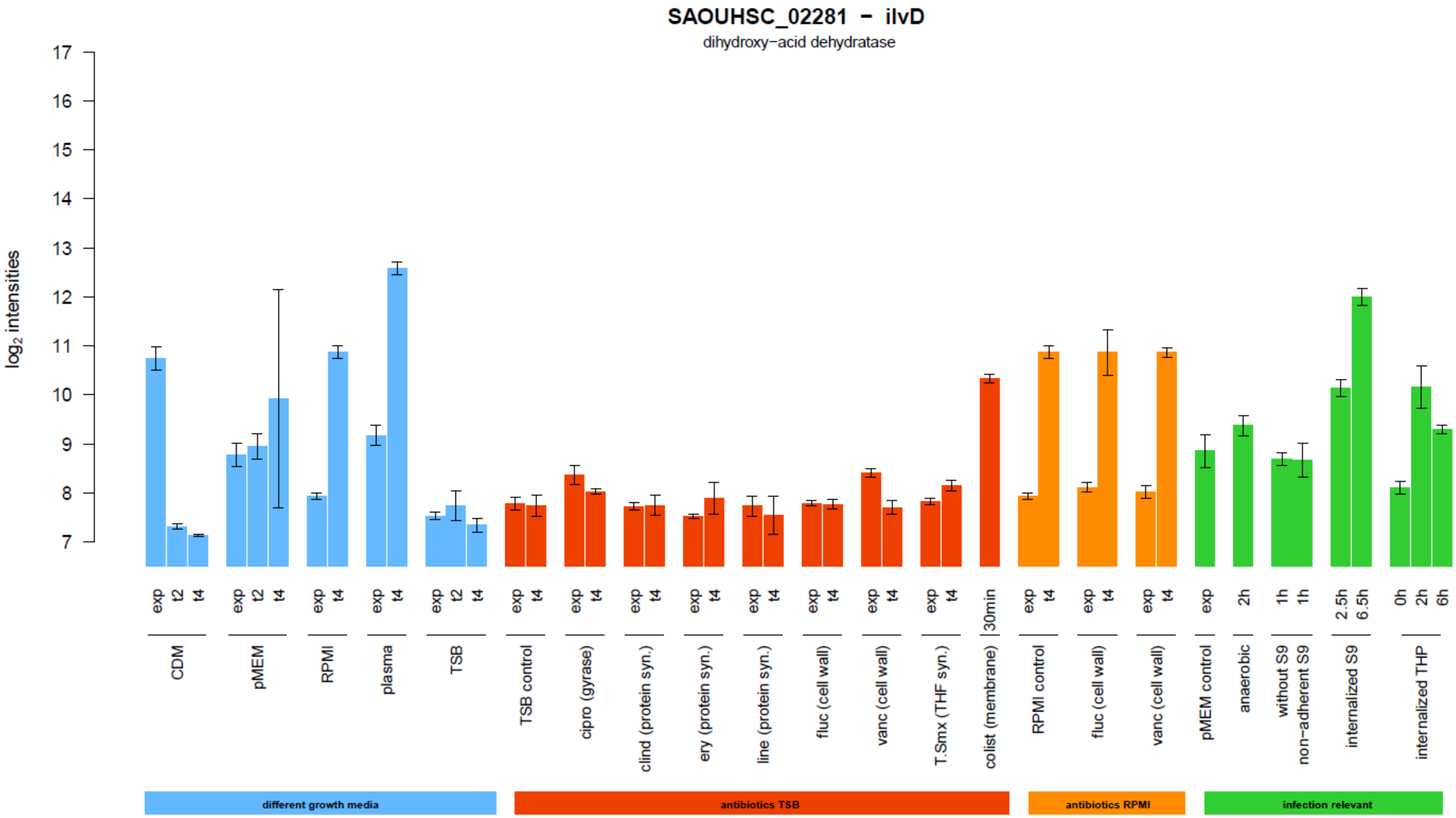

SAOUHSC_02281 – ilvD
dihydroxy−acid dehydratase
log2 intensities
17
16
15
14
13
12
11
10
9
8
7
exp
t2
t4
CDM
pMEM
RPMI
plasma
TSB
TSB control
cipro (gyrase)
clind (protein syn.)
ery (protein syn.)
line (protein syn.)
fluc (cell wall)
vanc (cell wall)
T.Smx (THF syn.)
30min
colist (membrane)
RPMI control
fluc (cell wall)
vanc (cell wall)
pMEM control
2h
anaerobic
1h
without S9
non−adherent S9
2.5h
6.5h
internalized S9
0h
2h
6h
internalized THP
different growth media
antibiotics TSB
antibiotics RPMI
infection relevant

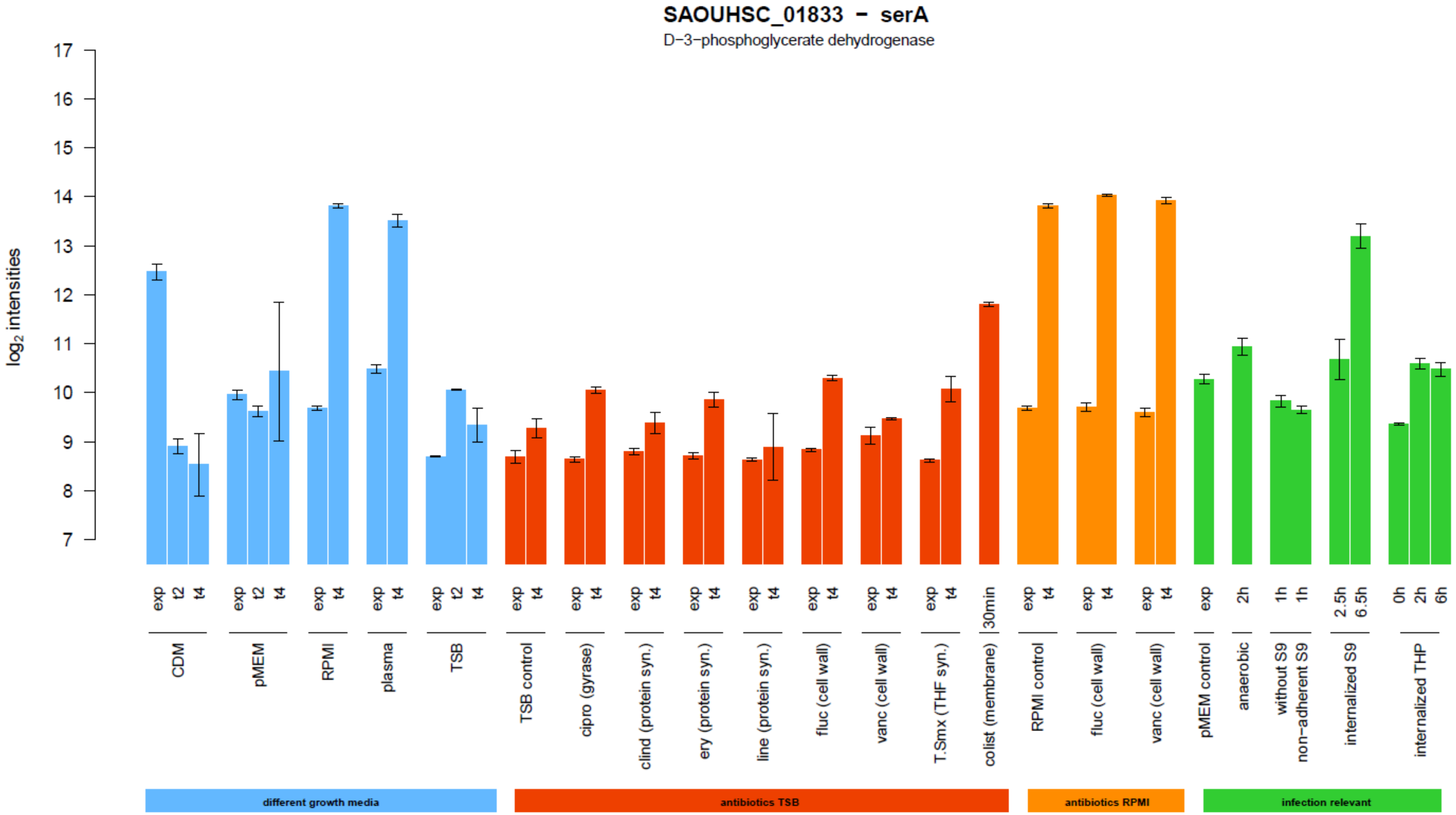
SAOUHSC_01833 – serA
D−3−phosphoglycerate dehydrogenase
log2 intensities
17
16
15
14
13
12
11
10
9
8
7
exp t2 t4
exp t2 t4
exp t4
exp t4
exp t2 t4
exp t4
exp t4
exp t4
exp t4
exp t4
exp t4
exp t4
exp t4
30min
exp t4
exp t4
exp t4
exp
2h
1h 1h
2.5h 6.5h
0h 2h 6h
CDM
pMEM
RPMI
plasma
TSB
TSB control
cipro (gyrase)
clind (protein syn.)
ery (protein syn.)
line (protein syn.)
fluc (cell wall)
vanc (cell wall)
T.Smx (THF syn.)
colist (membrane)
RPMI control
fluc (cell wall)
vanc (cell wall)
pMEM control
anaerobic
without S9
non−adherent S9
internalized S9
internalized THP
different growth media
antibiotics TSB
antibiotics RPMI
infection relevant

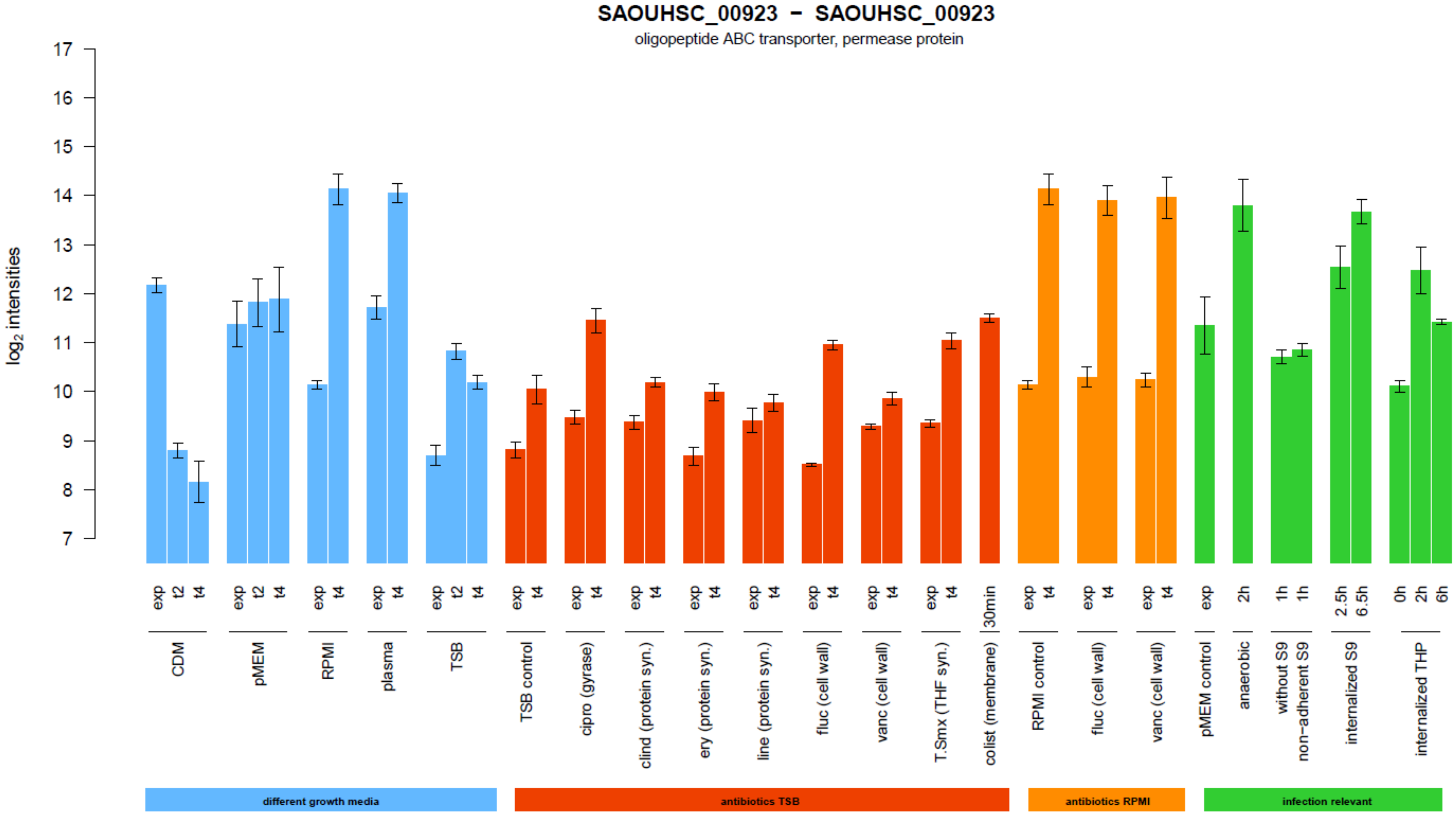

SAOUHSC_00923 – SAOUHSC_00923
oligopeptide ABC transporter, permease protein
log2 intensities
17
16
15
14
13
12
11
10
9
8
7
exp t2 t4
CDM
exp t2 t4
pMEM
exp t4
RPMI
exp t4
plasma
exp t2 t4
TSB
exp t4
TSB control
exp t4
cipro (gyrase)
exp t4
clind (protein syn.)
exp t4
ery (protein syn.)
exp t4
line (protein syn.)
exp t4
fluc (cell wall)
exp t4
vanc (cell wall)
exp t4
T.Smx (THF syn.)
30min
colist (membrane)
exp t4
RPMI control
exp t4
fluc (cell wall)
exp t4
vanc (cell wall)
exp
pMEM control
2h
anaerobic
1h 1h
without S9
non−adherent S9
2.5h 6.5h
internalized S9
0h 2h 6h
internalized THP
different growth media
antibiotics TSB
antibiotics RPMI
infection relevant

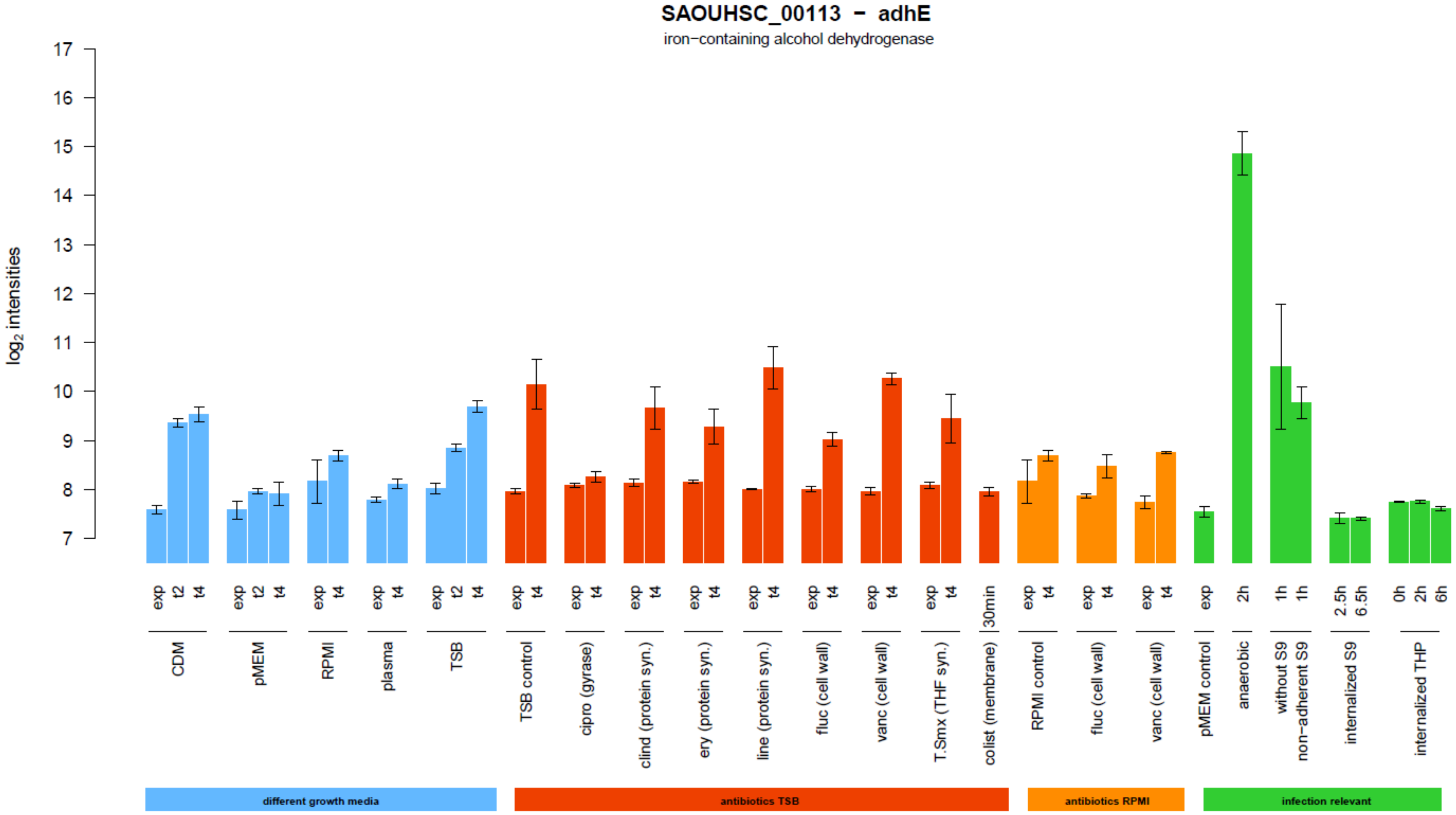
SAOUHSC_00113 – adhE
iron−containing alcohol dehydrogenase
log2 intensities
17
16
15
14
13
12
11
10
9
8
7
exp t2 t4
CDM
exp t2 t4
pMEM
exp t4
RPMI
exp t4
plasma
exp t2 t4
TSB
exp t4
TSB control
exp t4
cipro (gyrase)
exp t4
clind (protein syn.)
exp t4
ery (protein syn.)
exp t4
line (protein syn.)
exp t4
fluc (cell wall)
exp t4
vanc (cell wall)
exp t4
T.Smx (THF syn.)
30min
colist (membrane)
exp t4
RPMI control
exp t4
fluc (cell wall)
exp t4
vanc (cell wall)
exp
pMEM control
2h
anaerobic
1h
without S9
1h
non−adherent S9
2.5h 6.5h
internalized S9
0h 2h 6h
internalized THP
different growth media
antibiotics TSB
antibiotics RPMI
infection relevant

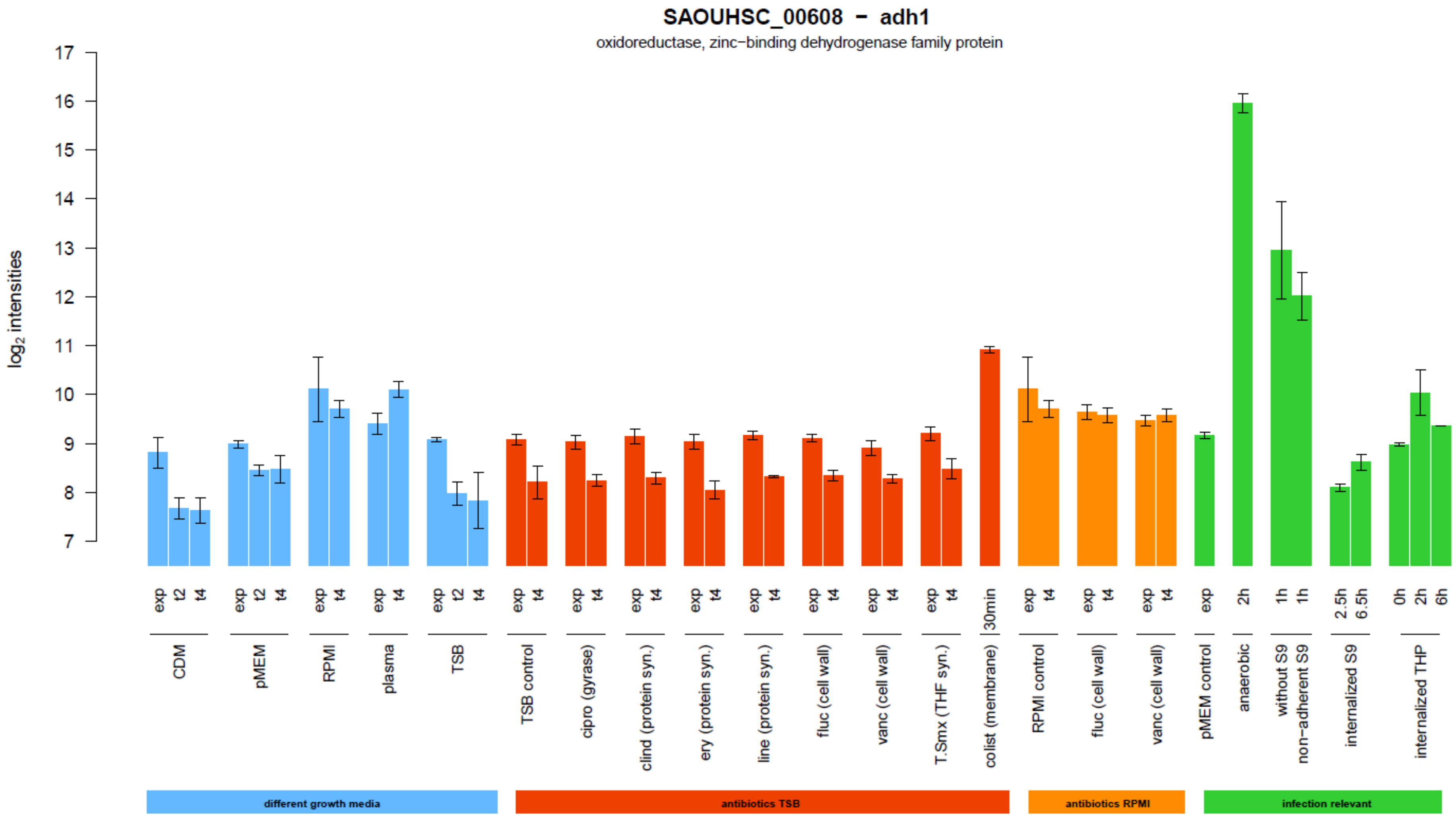

SAOUHSC_00608 – adh1
oxidoreductase, zinc−binding dehydrogenase family protein
log2 intensities
17
16
15
14
13
12
11
10
9
8
7
exp t2 t4
CDM
exp t2 t4
pMEM
exp t4
RPMI
exp t4
plasma
exp t2 t4
TSB
exp t4
TSB control
exp t4
cipro (gyrase)
exp t4
clind (protein syn.)
exp t4
ery (protein syn.)
exp t4
line (protein syn.)
exp t4
fluc (cell wall)
exp t4
vanc (cell wall)
exp t4
T.Smx (THF syn.)
30min
colist (membrane)
exp t4
RPMI control
exp t4
fluc (cell wall)
exp t4
vanc (cell wall)
exp
pMEM control
2h
anaerobic
1h 1h
without S9
non−adherent S9
2.5h 6.5h
internalized S9
0h 2h 6h
internalized THP
different growth media
antibiotics TSB
antibiotics RPMI
infection relevant

SAOUHSC_00187 – pflB

formate acetyltransferase

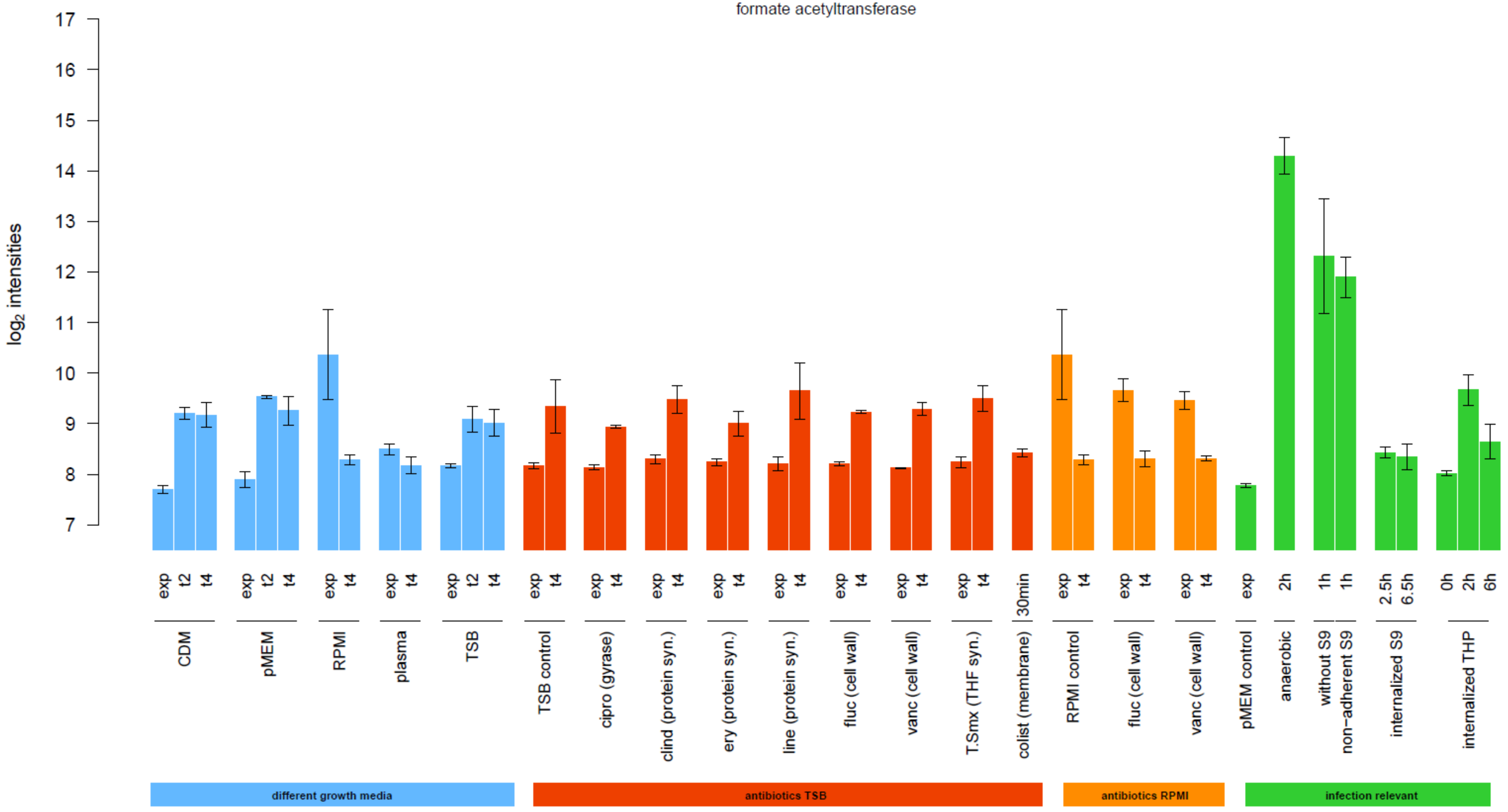

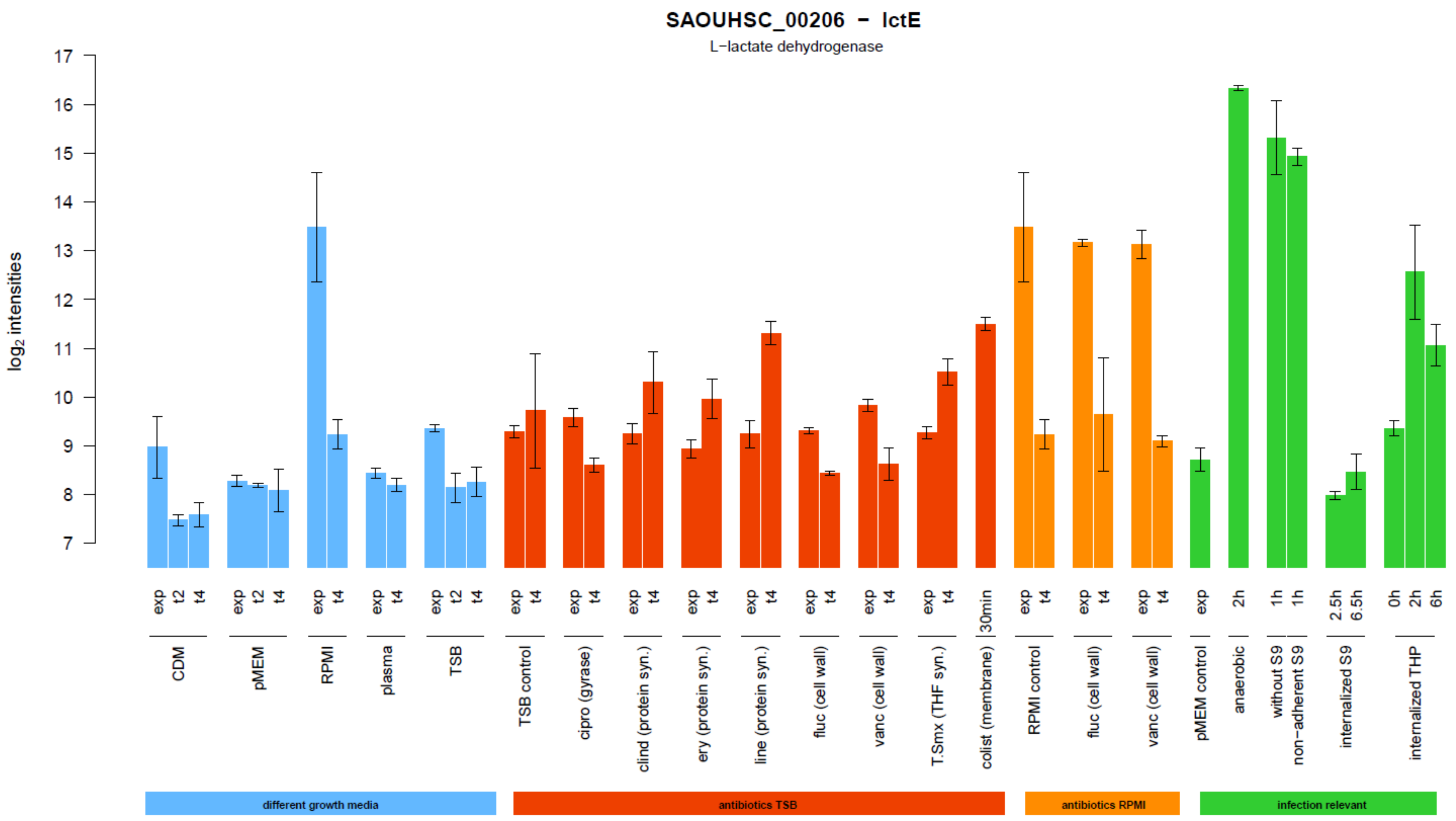
SAOUHSC_00206 – lctE
L−lactate dehydrogenase
log2 intensities
17
16
15
14
13
12
11
10
9
8
7
exp t2 t4
CDM
exp t2 t4
pMEM
exp t4
RPMI
exp t4
plasma
exp t2 t4
TSB
exp t4
TSB control
exp t4
cipro (gyrase)
exp t4
clind (protein syn.)
exp t4
ery (protein syn.)
exp t4
line (protein syn.)
exp t4
fluc (cell wall)
exp t4
vanc (cell wall)
exp t4
T.Smx (THF syn.)
30min
colist (membrane)
exp t4
RPMI control
exp t4
fluc (cell wall)
exp t4
vanc (cell wall)
exp
pMEM control
2h
anaerobic
1h
without S9
1h
non−adherent S9
2.5h 6.5h
internalized S9
0h 2h 6h
internalized THP
different growth media
antibiotics TSB
antibiotics RPMI
infection relevant

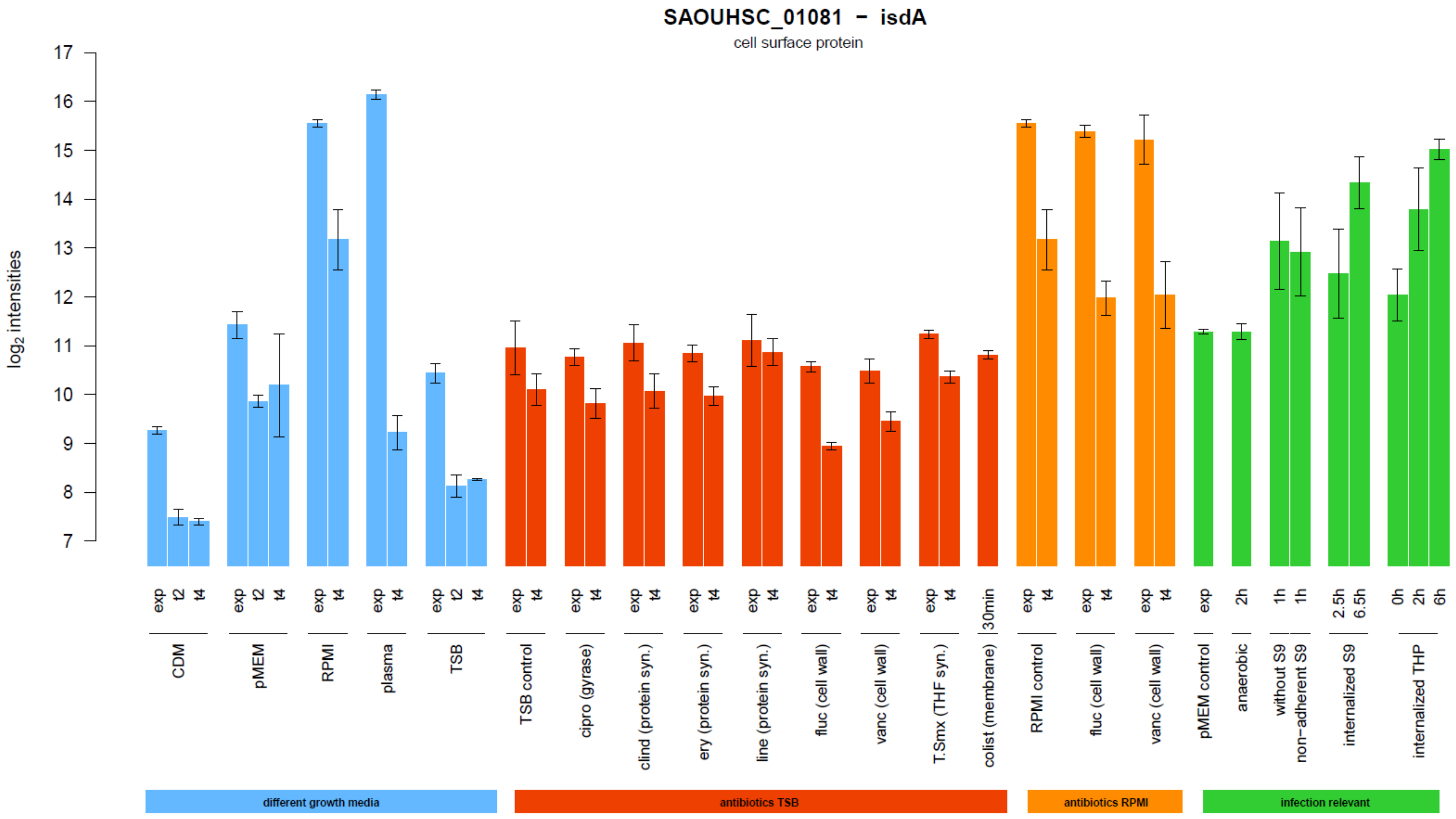

SAOUHSC_01081 – isdA
cell surface protein
log2 intensities
17
16
15
14
13
12
11
10
9
8
7
exp
t2
t4
CDM
pMEM
RPMI
plasma
TSB
TSB control
cipro (gyrase)
clind (protein syn.)
ery (protein syn.)
line (protein syn.)
fluc (cell wall)
vanc (cell wall)
T.Smx (THF syn.)
30min
colist (membrane)
RPMI control
pMEM control
2h
anaerobic
1h
without S9
non−adherent S9
2.5h
6.5h
internalized S9
0h
6h
internalized THP
different growth media
antibiotics TSB
antibiotics RPMI
infection relevant

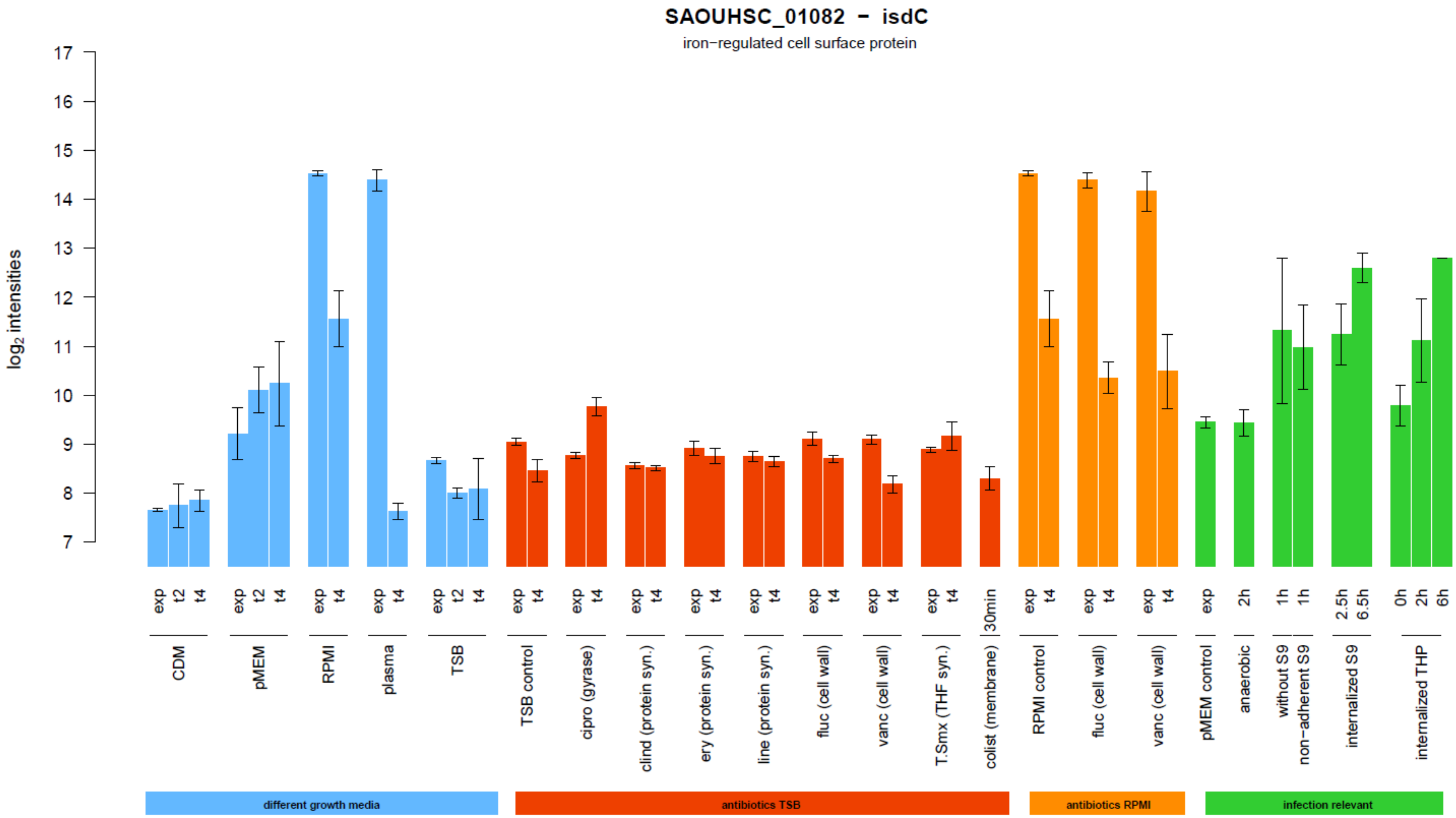
SAOUHSC_01082 – isdC
iron−regulated cell surface protein
log2 intensities
17
16
15
14
13
12
11
10
9
8
7
exp t2 t4
CDM
exp t2 t4
pMEM
exp t4
RPMI
exp t4
plasma
exp t2 t4
TSB
exp t4
TSB control
exp t4
cipro (gyrase)
exp t4
clind (protein syn.)
exp t4
ery (protein syn.)
exp t4
line (protein syn.)
exp t4
fluc (cell wall)
exp t4
vanc (cell wall)
exp t4
T.Smx (THF syn.)
30min
colist (membrane)
exp t4
RPMI control
exp t4
fluc (cell wall)
exp t4
vanc (cell wall)
exp
pMEM control
2h
anaerobic
1h
without S9
1h
non−adherent S9
2.5h 6.5h
internalized S9
0h 2h 6h
internalized THP
different growth media
antibiotics TSB
antibiotics RPMI
infection relevant

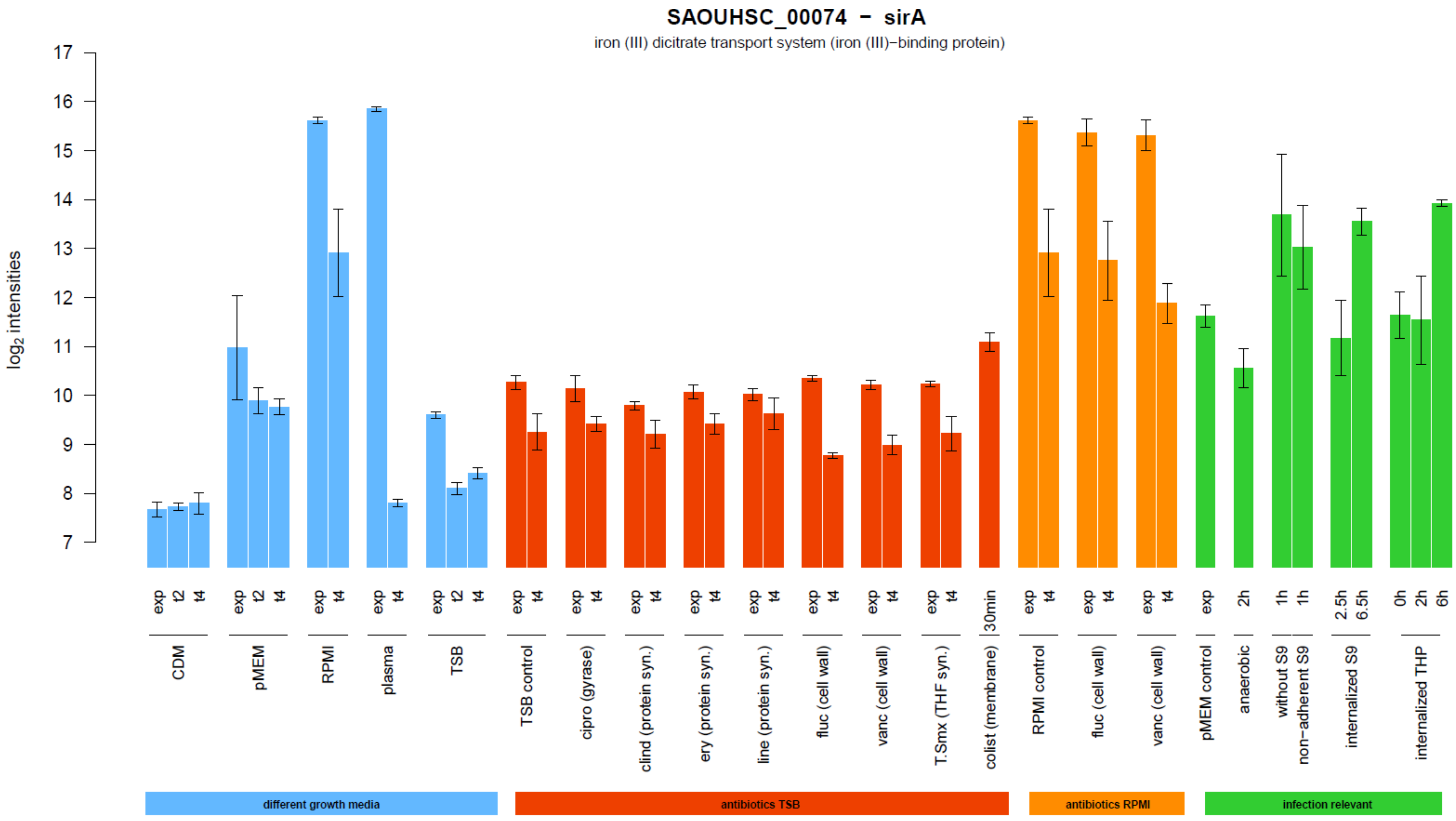

SAOUHSC_00074 – sirA
iron (III) dicitrate transport system (iron (III)–binding protein)
log2 intensities
17
16
15
14
13
12
11
10
9
8
7
exp t2 t4
CDM
exp t2 t4
pMEM
exp t4
RPMI
exp t4
plasma
exp t2 t4
TSB
exp t4
TSB control
exp t4
cipro (gyrase)
exp t4
clind (protein syn.)
exp t4
ery (protein syn.)
exp t4
line (protein syn.)
exp t4
fluc (cell wall)
exp t4
vanc (cell wall)
exp t4
T.Smx (THF syn.)
30min
colist (membrane)
exp t4
RPMI control
exp t4
fluc (cell wall)
exp t4
vanc (cell wall)
exp
pMEM control
2h
anaerobic
1h
without S9
1h
non–adherent S9
2.5h 6.5h
internalized S9
0h 2h 6h
internalized THP
different growth media
antibiotics TSB
antibiotics RPMI
infection relevant

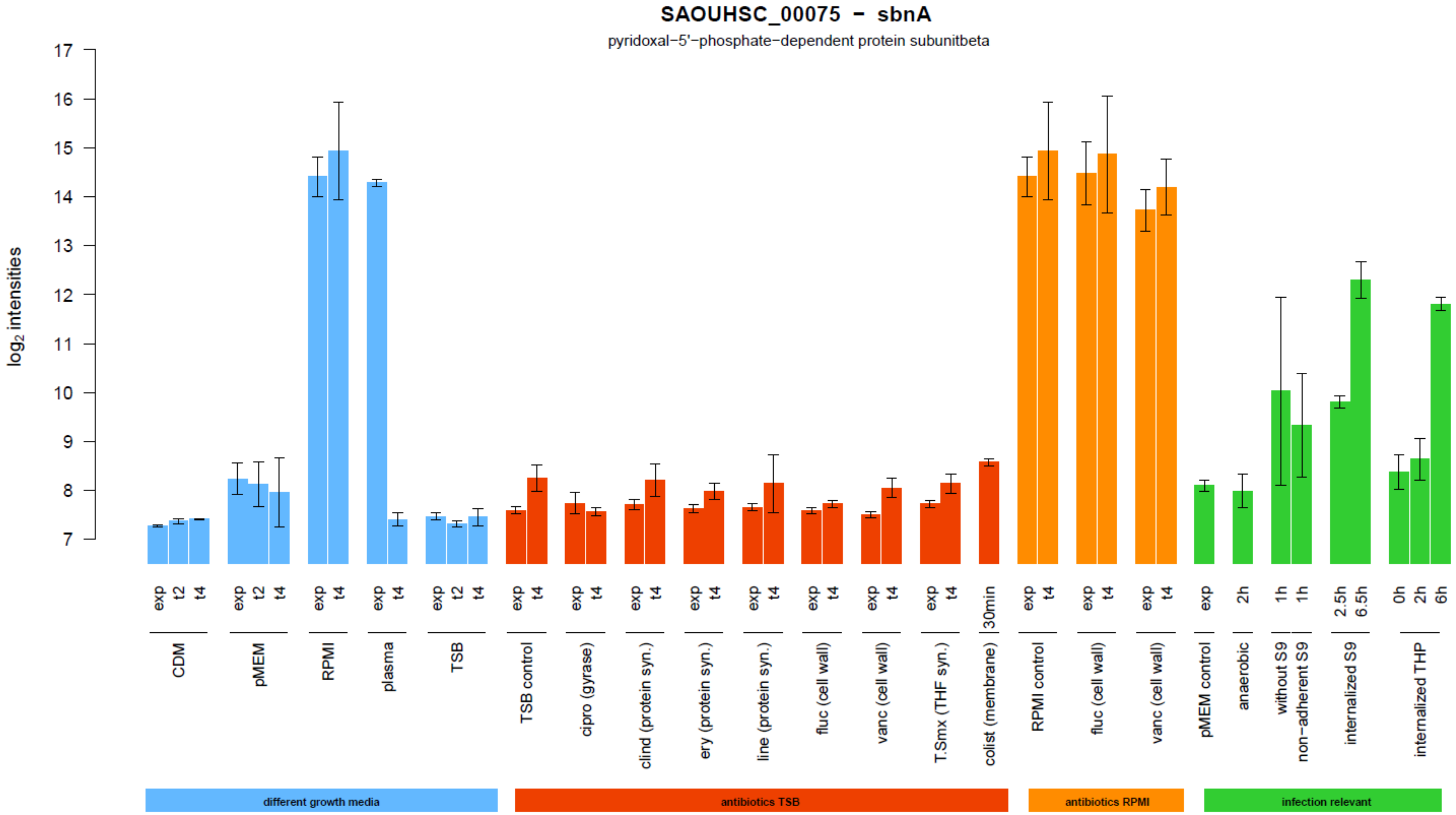
SAOUHSC_00075 – sbnA
pyridoxal−5'−phosphate−dependent protein subunitbeta
log2 intensities
17
16
15
14
13
12
11
10
9
8
7
exp t2 t4 exp t2 t4 exp t4 exp t4 exp t2 t4 exp t4 exp t4 exp t4 exp t4 exp t4 exp t4 exp t4 exp t4 30min exp t4 exp t4 exp t4 exp 2h 1h 1h 2.5h 6.5h 0h 2h 6h
CDM
pMEM
RPMI
plasma
TSB
TSB control
cipro (gyrase)
clind (protein syn.)
ery (protein syn.)
line (protein syn.)
fluc (cell wall)
vanc (cell wall)
T.Smx (THF syn.)
colist (membrane)
RPMI control
fluc (cell wall)
vanc (cell wall)
pMEM control
anaerobic
without S9
non−adherent S9
internalized S9
internalized THP
different growth media
antibiotics TSB
antibiotics RPMI
infection relevant
